# C8-substituted pyrido[3,4-*d*]pyrimidin-4(3*H*)-ones: Studies towards the identification of potent, cell penetrant Jumonji C domain containing histone lysine demethylase 4 subfamily (KDM4) inhibitors, compound profiling in cell-based target engagement assays

**DOI:** 10.1016/j.ejmech.2019.05.041

**Published:** 2019-09-01

**Authors:** Yann-Vaï Le Bihan, Rachel M. Lanigan, Butrus Atrash, Mark G. McLaughlin, Srikannathasan Velupillai, Andrew G. Malcolm, Katherine S. England, Gian Filippo Ruda, N. Yi Mok, Anthony Tumber, Kathy Tomlin, Harry Saville, Erald Shehu, Craig McAndrew, LeAnne Carmichael, James M. Bennett, Fiona Jeganathan, Paul Eve, Adam Donovan, Angela Hayes, Francesca Wood, Florence I. Raynaud, Oleg Fedorov, Paul E. Brennan, Rosemary Burke, Rob L.M. van Montfort, Olivia W. Rossanese, Julian Blagg, Vassilios Bavetsias

**Affiliations:** a Cancer Research UK Cancer Therapeutics Unit, The Institute of Cancer Research, London, UK; b Structural Genomics Consortium (SGC), University of Oxford, ORCRB Roosevelt Drive, Oxford, OX3 7DQ, UK; c Target Discovery Institute (TDI), Nuffield Department of Medicine, University of Oxford, NDMRB, Roosevelt Drive, Oxford, OX3 7FZ, UK

**Keywords:** Pyridopyrimidinones, KDM4 subfamily, KDM5 subfamily, KDM inhibitors, Histone demethylases

## Abstract

Residues in the histone substrate binding sites that differ between the KDM4 and KDM5 subfamilies were identified. Subsequently, a C8-substituted pyrido[3,4-*d*]pyrimidin-4(3*H*)-one series was designed to rationally exploit these residue differences between the histone substrate binding sites in order to improve affinity for the KDM4-subfamily over KDM5-subfamily enzymes. In particular, residues E169 and V313 (KDM4A numbering) were targeted. Additionally, conformational restriction of the flexible pyridopyrimidinone C8-substituent was investigated. These approaches yielded potent and cell-penetrant dual KDM4/5-subfamily inhibitors including **19a** (KDM4A and KDM5B Ki = 0.004 and 0.007 μM, respectively). Compound cellular profiling in two orthogonal target engagement assays revealed a significant reduction from biochemical to cell-based activity across multiple analogues; this decrease was shown to be consistent with 2OG competition, and suggests that sub-nanomolar biochemical potency will be required with C8-substituted pyrido[3,4-*d*]pyrimidin-4(3*H*)-one compounds to achieve sub-micromolar target inhibition in cells.

## Highlights

• Targeting of KDM4A residues E169 and V313 to improve affinity for the KDM4-subfamily.
• Potent and cell-penetrant KDM4/5-subfamily inhibitors such as **19a** were identified.
• Significant drop from biochemical to cell-based activity was observed.
• Lower potency in cells is linked to competition with the 2OG co-substrate.

## Graphical abstract

**Figure:**
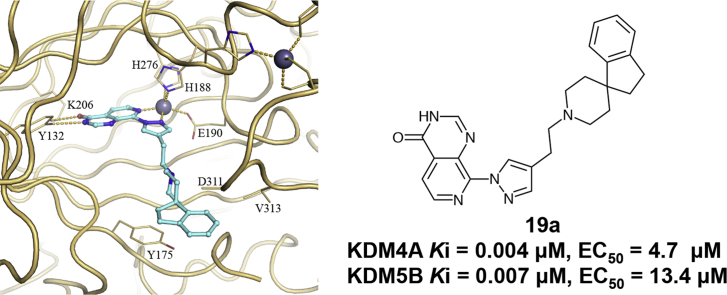

## Abbreviations list

Boc: *tert*-butoxycarbonyl
DME: 1,2-dimethoxyethane
DIBAL: diisobutylaluminium hydride
ESI: electrospray ionization
HPLC: high performance liquid chromatography
HRMS: high resolution mass spectrometry
JmjC: Jumonji C
LC: liquid chromatography
2OG: 2-oxoglutarate
PDB: protein data bank
SAR: structure-activity relationship
SEM: 2-(trimethylsilyl)ethoxymethyl
TFA: trifluoroacetic acid

## 1 Introduction

Human Jumonji C-domain (JmjC) histone lysine demethylases (KDMs) are Fe(II) and 2-oxoglutarate (2OG)-dependent oxygenases that demethylate mono-, di-, and trimethyl histone lysine substrates [1,2]. They comprise five subfamilies varying in domain architecture but sharing a well conserved catalytic site [3]. Since the reporting of the first JmjC histone lysine demethylase in 2006 [4,5], the JmjC KDMs have emerged as important players in maintaining chromatin architecture and regulating transcription [6].

The KDM4 subfamily consists of six members (A-F) that demethylate histone tail substrates, most notably di-, and tri-methylated lysine 9 (H3K9Me_3_/Me_2_) and lysine 36 (H3K36Me_3_/Me_2_) on histone 3 [6,7]. As well as the JmjC catalytic domain, KDM4 subfamily enzymes contain a JmjN domain [6]; KDM4A-C also possess PHD and Tudor methyl-lysine reader domains. It has been suggested that PHD and Tudor domains could stimulate KDM-driven demethylation *via* the recognition of histone substrates [8,9]. KDM4 subfamily members have been implicated in a number of human cancers, including promoting growth and cell survival in acute myeloid leukemia [10] and as regulators of ER-dependent breast cancer cell growth [11,12]. High expression of KDM4B in *MYCN*-amplified neuroblastomas is associated with poor prognosis [13], and overexpression of KDM4A is reported to promote site-specific copy number gain (e.g. 1q12) [14]. In addition, KDM4A is reported to promote prostate cancer initiation through the transcription factor ETV1 [15].

The human KDM5 subfamily, which consists of four members (A-D), is structurally the most closely related to the KDM4 subfamily and also contains a JmjN domain. KDM5 enzymes catalyse demethylation of H3K4Me_3_/Me_2_ histone lysine substrates and are implicated in various types of human cancers [[16], [17], [18]]. A requirement for KDM5A has been reported in drug tolerant human tumor cells [19], while KDM5B and KDM5C have been implicated in breast [20,21] and prostate cancers [22], respectively. Also Dalvi et al. recently reported that taxane-platin resistant lung cancer cells showed hypersensitivity to JIB-04 (a pan-selective JmjC KDM inhibitor) and the KDM5/KDM6 subfamily inhibitor GSK-J4 [23].

**Fig. 1:**
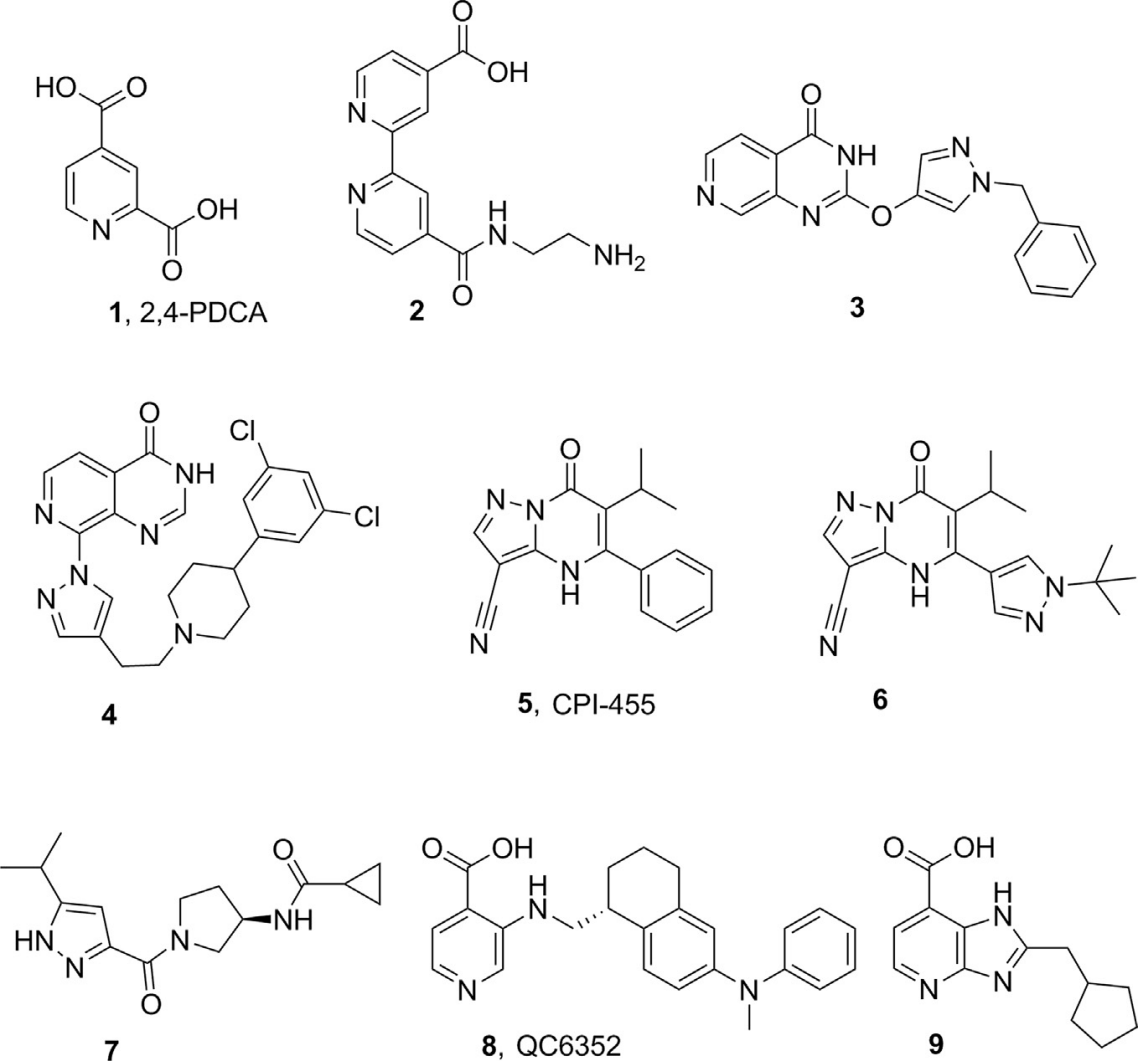
Reported KDM inhibitors.

These findings have generated considerable interest in small-molecule inhibitors of KDM demethylase functions [24]. Early inhibitors of JmjC KDMs, such as the pyridine carboxylic acid-based analogues **1** and **2** (Fig. 1) [25,26] display pan - JmjC histone demethylase inhibitory activity, and feature a carboxylic acid which may hinder cell permeability [27]. More recently, a range of structurally diverse JmjC KDM inhibitors has been reported with evidence of selective inhibition of specific KDM subfamilies. For example, the pyridopyrimidinone derivatives **3** and **4** (Fig. 1) show selective inhibition of both KDM4 and KDM5 subfamilies over members of KDM2, 3, or 6 demethylases [28,29]. Compounds **5**, **6**, and **7** (Fig. 1) selectively inhibit KDM5 subfamily members [[30], [31], [32]], and compound **8** (Fig. 1) has recently been reported as a potent KDM4 subfamily inhibitor with promising selectivity [33]. In addition, a chemoproteomics approach revealed compound **9** as a selective KDM4 subfamily inhibitor [34]. Covalent inhibitors of KDM5 subfamily have also been recently reported [35,36]. In addition, a rhodium(III) complex has been reported as a selective inhibitor of KDM5A [37].

We previously reported the discovery of C8-substituted pyridopyrimidinones (e.g. **4**) as a potent, cell penetrant dual KDM4 and KDM5 inhibitors [29]. Determination of the crystal structures of the mono *m*-Cl counterpart of **4** in complex with both KDM4A and KDM5B allowed us to identify residue differences in the histone substrate binding site that could be exploited for selective KDM4 subfamily inhibition. Herein, we report our efforts to gain selectivity for KDM4 over KDM5 subfamily inhibition by targeting residues V313 (KDM4A) and E169 (KDM4A). We also report our attempts to reduce the conformational flexibility of the C8-substituent to improve KDM inhibitory activity and investigate the impact of such changes on KDM4 *versus* KDM5 selectivity. Finally, we demonstrate cell-based target engagement for exemplar compounds from the 2-OG-competitive pyridopyrimidinone series and reveal a consistent drop off from *in vitro* biochemical to cell-based activity.

## 2 Chemistry

**Scheme 1:**
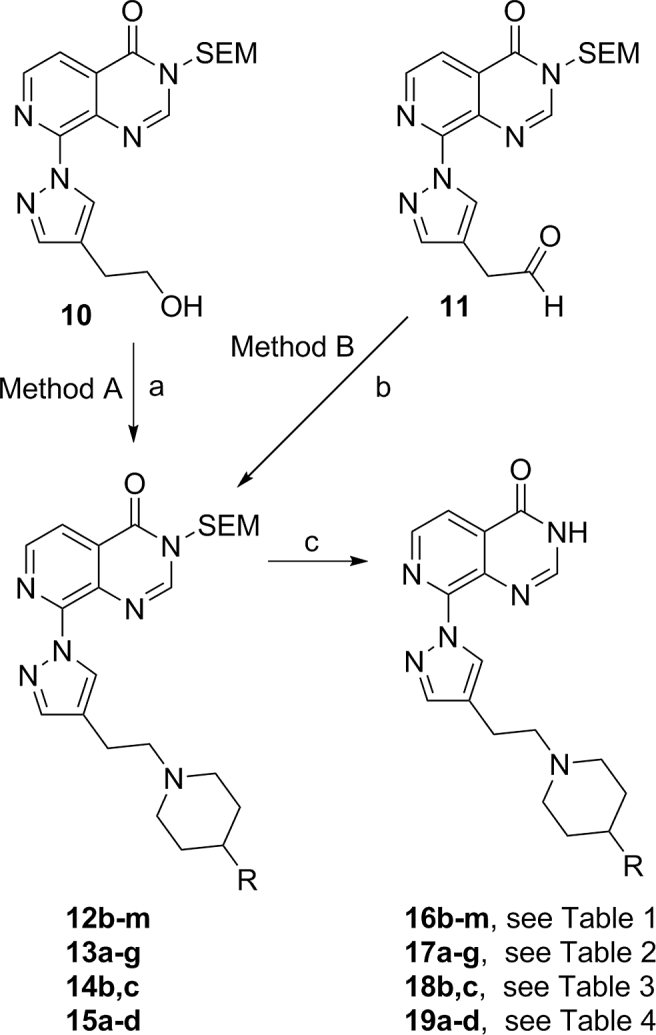
^a^Reagents and conditions: (a) (i) methanesulfonic anhydride, anhydrous CH_2_Cl_2_, 0 °C, 15 min, work-up, (ii) triethylamine, C4-substituted piperidine, anhydrous DMF, 50 °C, 15 h; (b) C4-substituted piperidine, anhydrous 1,2-dichloroethane or CH_2_Cl_2_, NaBH(OAc)_3_, room temp.; (c) 6 M HCl, THF, 50–60 °C, 3–8 h or 4 M HCl in dioxane, H_2_O, 50 °C.

Compounds **16b-m** (Table 1), **17a-g** (Table 2), **18b-c** (Table 3), and **19a-d** (Table 4) were prepared from key intermediates **10** and **11** *via* methods A and B, respectively as previously described for the synthesis of closely related analogues (Scheme 1) [29].

**Table 1:**
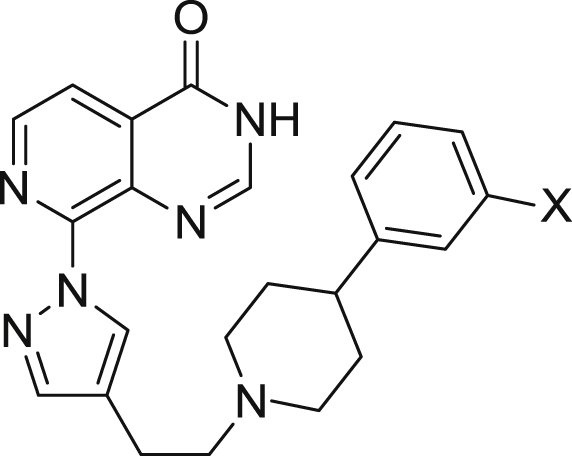
4-Phenylpiperidine derivatisation: *m*-Phenyl substitution^a^.

Compound	X	KDM4A IC_50_ (μM)	KDM4B IC_50_ (μM)	KDM5B IC_50_ (μM)	KDM5C IC_50_ (μM)	Caco-2 (x10^−6^ cm/s)
**16a**^b^	Cl	0.102 ± 0.058	0.031 ± 0.012	0.023	0.065	11.80
**16b**	F	0.084± 0.019	0.032± 0.016	0.029	0.096	11.63
**16c**	^*t*^Bu	0.141	0.049± 0.041	0.025^c^	0.063^c^	4.67
**16d**	OMe	0.212± 0.013	0.067± 0.036	0.023	0.086	not tested
**16e**		0.178	0.101	0.051^c^	0.156^c^	10.77
**16f**		0.282	0.145	0.066^c^	0.213^c^	not tested
**16g**		0.136	0.033± 0.012	0.042^c^	0.079^c^	not tested
**16h**		0.056	0.027± 0.014	0.017^c^	0.059^c^	<1.73
**16i**		0.088± 0.046	0.047± 0.024	0.021^c^	0.054^c^	<1.52
**16j**		0.083± 0.049	0.019± 0.013	0.012	0.030	<0.76
**16k**		0.259	0.063± 0.023	0.048^c^	0.113^c^	not tested
**16l**		0.059	0.015	0.012^c^	0.035^c^	<0.92
**16m**		0.038± 0.006	0.008	0.006^c^	0.014^c^	<1.52

a Results are mean values of two independent determinations or mean (±SD) for n > 2 unless specified otherwise.

b Data taken from reference 29.

c Results are from a single experiment.

**Table 2:**
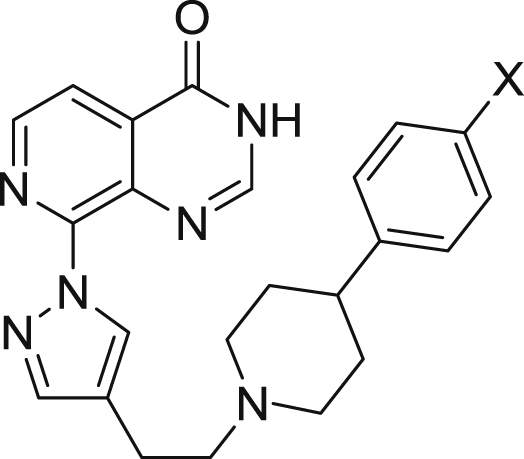
4-Phenylpiperidine derivatisation: *p*-Phenyl substitution^a^.

Compound	X	KDM4A IC_50_ (μM)	KDM4B IC_50_ (μM)	KDM5B IC_50_ (μM)	KDM5C IC_50_ (μM)	Caco-2 (x10^−6^ cm/s)
**17a**		0.218	0.153	0.146	0.896^b^	not tested
**17b**		0.131	0.024± 0.018	0.080	0.246	2.29
**17c**		0.068	0.032	0.127^b^	0.549^b^	<1.81
**17d**		0.188± 0.063	0.079± 0.033	0.081^b^	0.313^b^	not tested
**17e**		0.077± 0.007	0.018± 0.006	0.026^b^	0.053^b^	<0.55
**17f**		0.093	0.020± 0.011	0.012	0.048	<0.76
**17g**		0.103	0.020± 0.009	0.041	0.108	<1.52

a Results are mean values of two independent determinations or mean (±SD) for n > 2 unless specified otherwise.

b Results are from a single experiment.

**Table 3:**
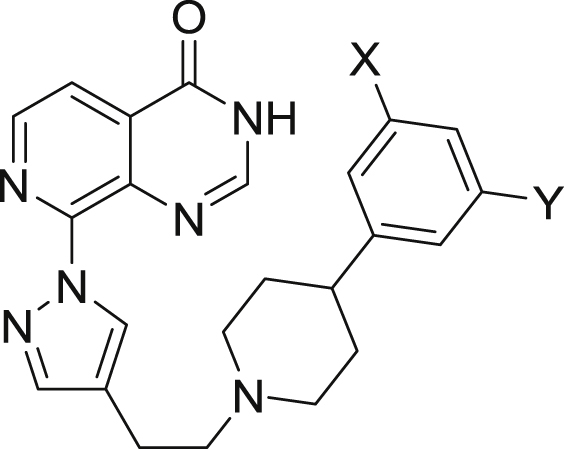
4-Phenylpiperidine derivatisation: 3,5-phenyl substitution^a^.

Compound	X	Y	KDM4A IC_50_ (μM)	KDM4B IC_50_ (μM)	KDM5B IC_50_ (μM)	KDM5C IC_50_ (μM)	Caco-2 (x10^−6^ cm/s)
**18a**^b^	CF_3_	H	0.128	0.039	0.018	0.070	15.31
**18b**	CF_3_		0.071	0.033± 0.015	0.017	0.038	<0.76
**18c**	CF_3_		0.246± 0.146	0.102± 0.062	0.032^c^	0.096^c^	<0.76

a Results are mean values of two independent determinations or mean (±SD) for n > 2 unless specified otherwise.

b Data taken from reference 29.

c Results are from a single experiment.

**Table 4:**
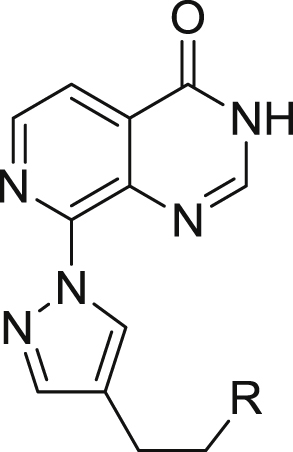
Pyrazole C4-substituent: Conformational restriction^a^.

Compound	R	KDM4A IC_50_ (μM)	KDM4B IC_50_ (μM)	KDM5B IC_50_ (μM)	KDM5C IC_50_ (μM)	Caco-2 (x10^−6^ cm/s)
**19a**		0.100± 0.041	0.043± 0.021	0.038	0.123	11.64
**19b**		0.143± 0.056	0.045	0.114	0.214	18.06
**19c**		0.184± 0.069	0.057	0.123^b^	0.470^b^	4.25
**19d**		0.107± 0.020	0.029± 0.006	0.014^b^	not tested	13.33

a Results are mean values of two independent determinations or mean (±SD) for n > 2 unless specified otherwise.

b Results are from a single experiment.

**Scheme 2:**
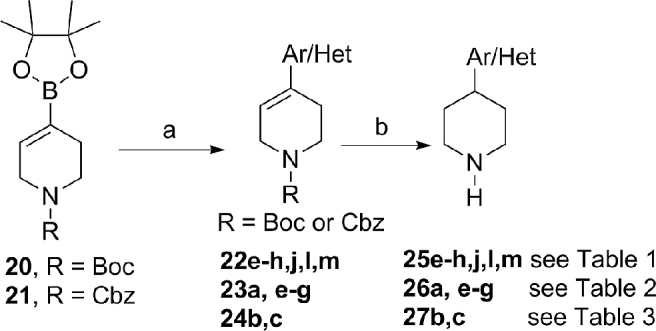
^a^Reagents and conditions: (a) aryl/heteroaryl halide, Pd(dppf)Cl_2_, DME, 1 M aqueous Na_2_CO_3_, microwave, 120 °C, 45 min; (b) (i) Pd(OH)_2_ on carbon, EtOH, 1 M HCl, H_2_, room temp 1 h or 10% Pd/C, EtOH, H_2_, room temp, 2 h, (ii) when R = Boc: 4 M HCl in dioxane, room temp, 2 h or when R = Cbz: AcOH, 10% Pd/C, H_2_, room temp, 4–6 h.

The key C4-substituted piperidine intermediates required for the S_N_2 displacement or reductive amination reactions to prepare **12b-m**, **13a-g**, **14b-c**, and **15a-d** (see Scheme 1) were commercially available or obtained as shown in Scheme 2. *N*-Protected tetrahydropyridine-4-boronic acid pinacol ester was reacted with appropriately substituted aryl/heteroaryl halides under Pd-catalysed conditions to provide intermediates **22e-h, 22j, 22l,m, 23a, 23e-g** and **24b,c** (Scheme 2). Appropriately substituted aryl/heteroaryl halides were commercially available or readily synthesised *via* standard transformations as detailed in the experimental procedure (see Supporting Information). Reduction of the double bond followed by the removal of the *N*-protecting group afforded the desired C4-substituted piperidines (Scheme 2).

**Scheme 3:**
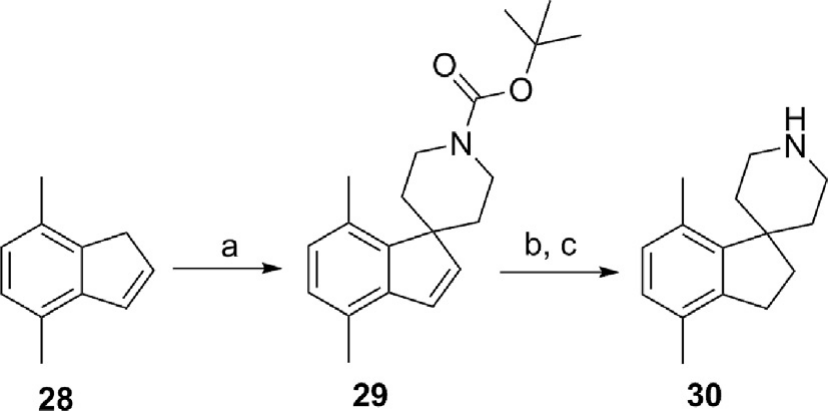
^a^Reagents and conditions: (a) (i) LiN(SiMe_3_)_2_ (1 M solution in THF), THF, 0 °C, 45 min, (ii) *tert*-butyl bis(2-chloroethyl)carbamate, 0 °C, 2 h; (b) 10% Pd/C, EtOH, H_2_, room temp, 2 h; (c) 4 M HCl in dioxane, room temp, 2 h.

The spirocyclopiperidine **30** required for the synthesis of **19b** (Table 4) was accessed by reacting 4,7-dimethyl-1*H*-indene with lithium bis(trimethylsilyl)amide and *tert*-butyl bis(2-chloroethyl)carbamate followed by the hydrogenation of the double bond in **29**, and finally, removal of the Boc protecting group under acidic conditions (Scheme 3).

**Scheme 4:**
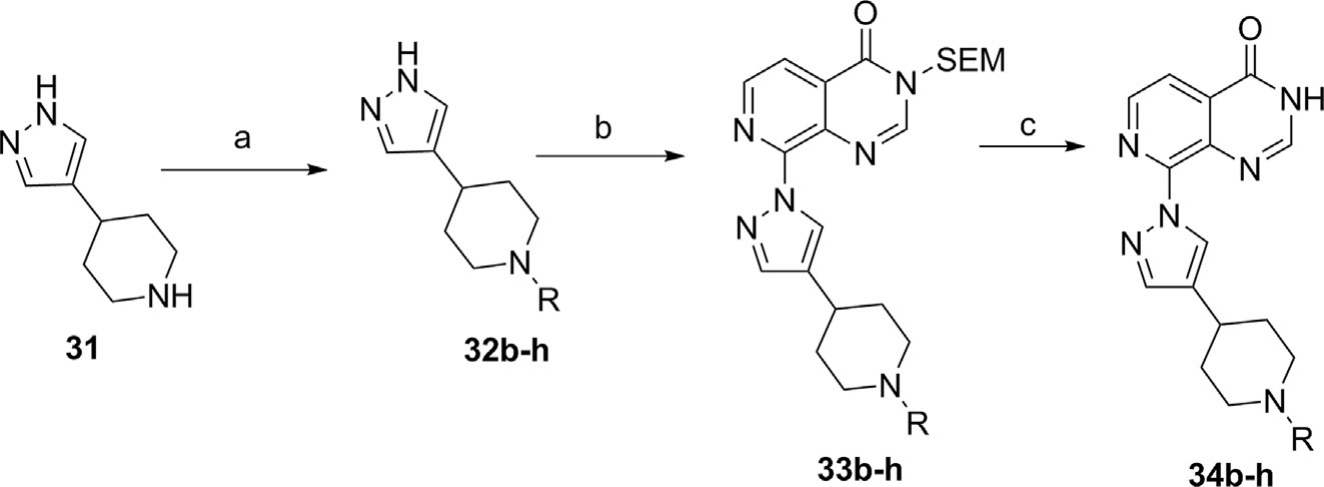
^a^Reagents and conditions: (a) aldehyde, NaBH(OAc)_3_, DMF, room temp, stirring up to 6 h; (b) Cs_2_CO_3_, anhydrous MeCN, 8-chloro-3-((2-(trimethylsilyl)ethoxy)methyl)pyrido[3,4-*d*]pyrimidin-4(3*H*)-one, reflux, 18 h; (c) 6 M HCl, THF, 50–60 °C, 3–8 h.

Access to conformationally constrained pyrazole-C4 compounds **34b-h** (Scheme 4, Table 5) was achieved by reductive amination of the relevant aldehyde by piperidine derivative **31** followed by the introduction of the substituted pyrazole moiety **32** at the C8-pyridopyrimidinone position *via* a S_N_Ar displacement reaction, and removal of the SEM protecting group under acidic conditions (Scheme 4).

**Table 5:**
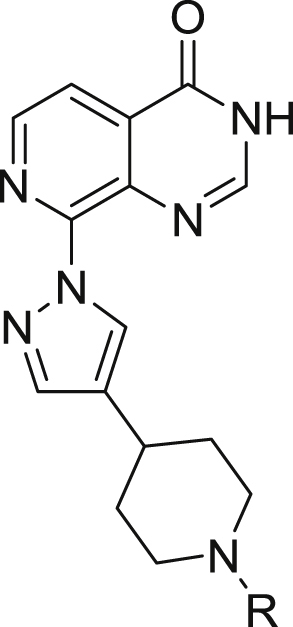
Pyrazole C4-substituent: Conformational restriction^a^.

Compound	R	KDM4A IC_50_ (μM)	KDM4B IC_50_ (μM)	KDM5B IC_50_ (μM)	KDM5C IC_50_ (μM)	Caco-2 (x10^−6^ cm/s)
**34a**	Me	2.06	0.82	0.156	0.163	<0.76
**34b**	Et	0.693± 0.231	0.455± 0.037	0.149	0.081^b^	<0.76
**34c**		0.848± 0.234	0.504± 0.072	0.439	0.566	not tested
**34d**		1.06	0.497	0.601^b^	0.856^b^	not tested
**34e**		0.756	0.372	0.447^b^	0.571^b^	<0.76
**34f**		0.559± 0.152	0.248± 0.048	0.134	0.202^b^	5.46
**34g**		0.613	0.302	0.068^b^	0.132^b^	2.12
**34h**		2.41	0.573	0.131^b^	0.377^b^	not tested

a Results are mean values of two independent determinations or mean (±SD) for n > 2 unless specified otherwise.

b Results are from a single experiment.

**Scheme 5:**
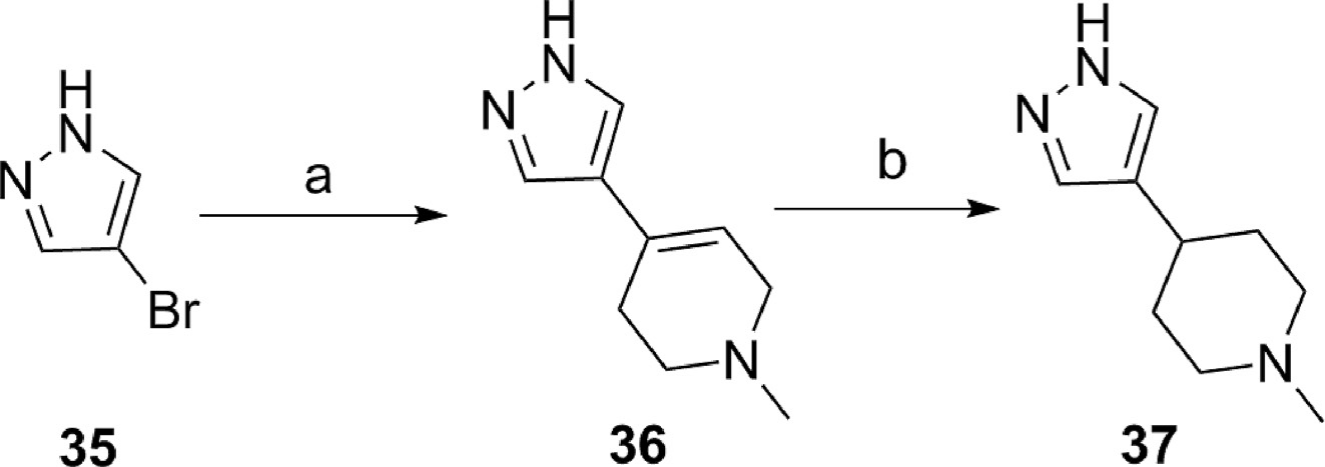
^a^Reagents and conditions: (a) (i) 2.5 M *n*BuLi in hexane, THF, −78 °C to room temp (1.5 h) then cool to −78 °C, (ii) 1-methylpiperidin-4-one, warm up to room temp, 1.5 h, then AcOH/TFA, 90 °C, 4 h; (b) 10% Pd/C, EtOH, 1 M HCl, H_2_, 16 h.

Preparation of 1-methyl-4-(1*H*-pyrazol-4-yl)piperidine (**37**), required for the synthesis of **34a** (Table 5) was carried out using an alternative approach whereby 4-bromopyrazole (**35**) was reacted with *n*BuLi followed by the addition of 1-methylpiperidin-4-one. Subsequent dehydration of the addition product under acidic conditions followed by hydrogenation of the double bond in **36** afforded **37** (Scheme 5). The incorporation of the piperidine **37** at the C8-pyridopyrimidinone position was achieved *via* a S_N_Ar displacement reaction as described for analogues **33b-h** (Scheme 4), and the SEM protecting group was removed by treatment with TBAF in THF.

**Scheme 6:**
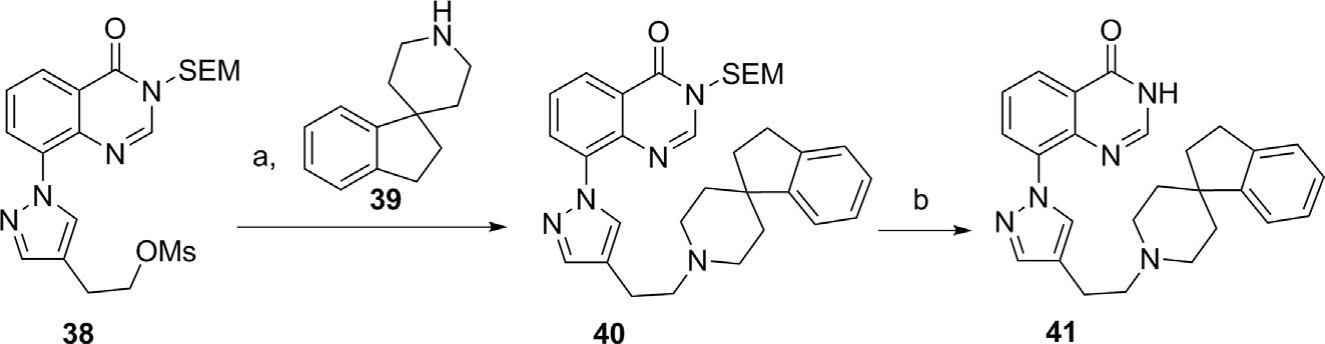
^a^Reagents and conditions: (a) triethylamine, anhydrous DMF, 50 °C, 15 h; (b) 6 M HCl, THF, 50 °C, 3 h.

Access to **41**, a matched-pair inactive control for **19a,** was achieved upon treatment of the mesylate **38**, prepared as previously described [38], with spirocyclopiperidine **39** and triethylamine in DMF followed by the removal of the SEM protecting group of **40** under acidic conditions (Scheme 6).

## 3 Results and discussion

We recently reported a series of C8-substituted pyridopyrimidinone derivatives, with the most advanced analogues, such as **4** (Fig. 1) and **16a** (Table 1), demonstrating selective and equipotent inhibition of KDM4 and KDM5 over the KDM2, KDM3, and KDM6 subfamilies [29].

**Fig. 2:**
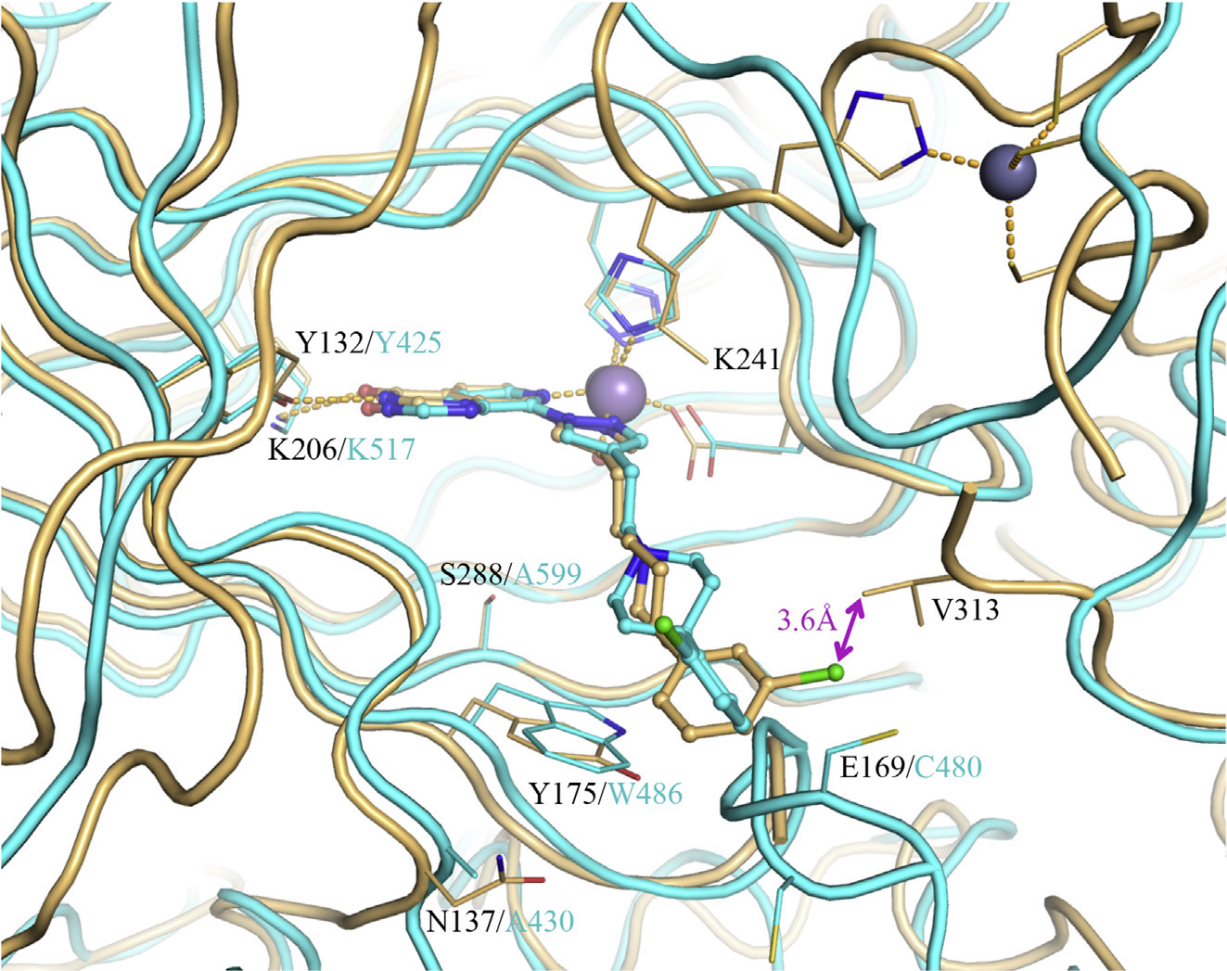
Overlay of crystal structures of **16a** bound to KDM4A (light orange), and KDM5B (cyan). Compound **16a** is shown in ball and stick representation and protein backbone chains are represented as cartoon tubes, with key amino acid side-chains displayed in line representation. Metal ions are shown as spheres. Labels highlight residues that are different between KDM4A (black) and KDM5B (cyan). KDM5B loop 91–100 is not displayed due to its construct-derived artefactual position as discussed in Fig. S1. (For interpretation of the references to colour in this figure legend, the reader is referred to the Web version of this article.)

To identify potential interactions that could increase KDM4 subfamily potency and selectivity over KDM5 subfamily enzymes, we determined the crystal structures of **16a** in complex with KDM4A and KDM5B (Fig. 2). In the KDM4A-bound structure, the C4-pyrazole substituent of **16a** extends into the histone substrate binding site and towards the surface of the enzyme (Fig. S2); the *m*-Cl substituent on the phenyl group makes hydrophobic contact with V313 of KDM4A (Fig. 2), a residue that is conserved across all KDM4 subfamily members (Fig. S3). We postulated that this favourable hydrophobic interaction with V313 contributes to the increased KDM4 inhibitory profile of **16a** compared to the KDM5-preferring profile of earlier analogues in this series [29]. In addition, **16a** is a more potent inhibitor of both KDM4A and KDM4B than its unsubstituted phenyl counterpart (compound **54g** in reference 29) consistent with the observed hydrophobic interaction of the *m*-Cl substituent. Furthermore, the crystal structure of **16a** bound to KDM5B indicated no residue equivalent to KDM4A V313 due to a different protein fold (Fig. 2). This observation further supports the hypothesis that the hydrophobic contact of the *m*-Cl substituent in **16a** with V313 increases KDM4 inhibitory activity leading to a balanced KDM4/5 inhibitory profile.

A more detailed study of the **16a**-bound KDM5B crystal structure revealed additional residues close to the *m*-chlorophenyl moiety of the bound inhibitor that could be exploited to enhance KDM4-subfamiliy selectivity (Fig. 2). For example, C480 is a serine residue in all other KDM5 subfamily members, and a glutamate in the KDM4 subfamily (E169 in KDM4A, Fig. 2, S3 and S4). This suggests a potential for differential KDM4 versus KDM5 targeting although we recognised that exploiting selective interaction with the side chain of E169 (KDM4A numbering) might be challenging due to its solvent exposed location and high mobility; for example, the side chain of E169 could not be associated to any electron density in the **16a-**bound KDM4A crystal structure. W486 in KDM5B is conserved across the KDM5 subfamily whilst the equivalent residue in the KDM4 subfamily is a tyrosine (Y175 in KDM4A, Fig. 2, S3 and S4). However, the potential to exploit this difference is limited due to the similarity of tryptophan and tyrosine residues. We also observed that A430 in KDM5B is an asparagine residue in KDM4A (N137, Fig. 2). However, these residues are not conserved within their respective subfamilies. N137 in KDM4A corresponds to S138 in KDM4B, S141 in KDM4D, and N139 in KDM4C (Fig. S3), whilst A430 in KDM5B corresponds to S414 in KDM5A, and to H445/H435 in KDM5C and KDM5D, respectively (Fig. S4), rendering exploitation of such differences for KDM4 over KDM5 subfamily selective inhibition challenging.

**Fig. 3:**
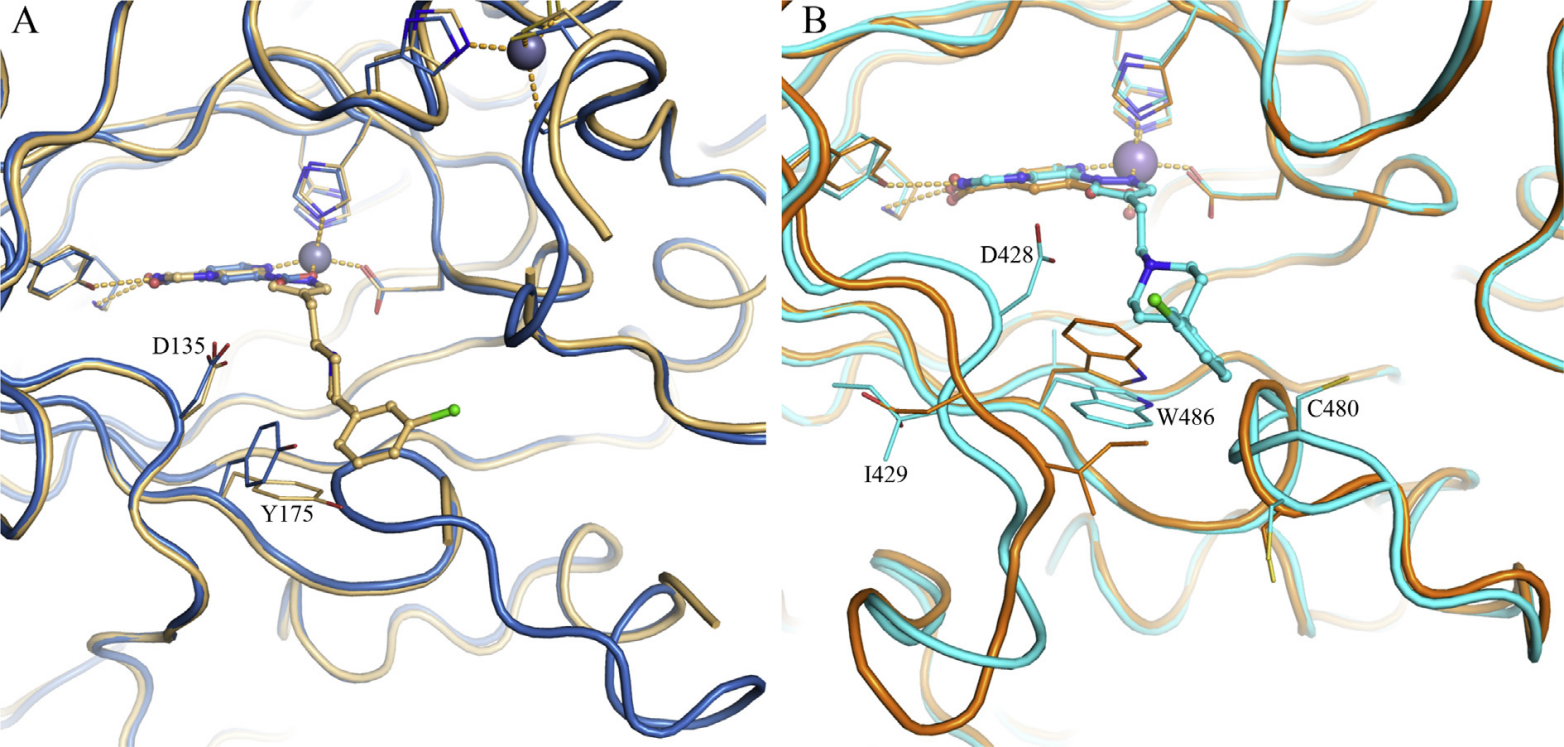
**A**) Overlay of crystal structures of **1** (2,4-PDCA, blue, PDB 2VD7) and **16a** (beige) bound to KDM4A. **B**) Overlay of crystal structures of **1** (2,4-PDCA, orange, PDB 5A3W) and **16a** (cyan) bound to KDM5B. Metal ions are shown as spheres. Protein backbone chains are represented as cartoon tubes, key residues are displayed in line representation. Compounds **1** (2,4-PDCA) and **16a** are shown in ball and stick representation. KDM5B loop 91–100 is not displayed due to its construct-derived artefactual position as discussed in Fig. S1. (For interpretation of the references to colour in this figure legend, the reader is referred to the Web version of this article.)

Based on these structural insights, we decided to further explore the favourable hydrophobic contact with V313 (KDM4A) to increase affinity and selectivity for the KDM4 over KDM5 subfamily. As an alternative approach, we also considered the introduction of a basic substituent at either the *m*- or *p*-position of the phenyl ring in **16a** to target E169 (KDM4A). We recognised that achieving selective KDM4-over KDM5-subfamily inhibition may be challenging due to protein flexibility in the histone substrate binding site, as exemplified by induced-fit movement observed in C8-substituted pyrido[3,4-*d*]pyrimidin-4-one-bound KDM4A and KDM5B crystal structures compared with small 2OG-competitive inhibitors such as **1** (Fig. 3). For example, the pyrazole C4-substituent in **16a** induces movement of Y175 and nearby loop 160–171 of KDM4A, such that residues 162–169 become too mobile to be observed in the electron density map. In the KDM5B-**16a** crystal structure, W486 and nearby loop 426–435 are displaced relative to the corresponding compound **1**-bound structure, and an alternative conformation is observed for residues 479–482. We also recognised that conformational flexibility of the ligand pyrazole C4-substituent may adversely impact KDM inhibition and subfamily selectivity.

Both fluoro and *tert*-butyl substitutions (compounds **16b** and **16c**, respectively) resulted in similar KDM inhibitory activities to those observed for the *m*-Cl counterpart **16a** (Table 1). The methoxy and isopropyl derivatives **16d** and **16e** proved marginally inferior KDM4 subfamily inhibitors compared to **16a**, and a similar trend was also observed with the introduction of a bulkier alkoxy substituent (**16f**, Table 1).

**Fig. 4:**
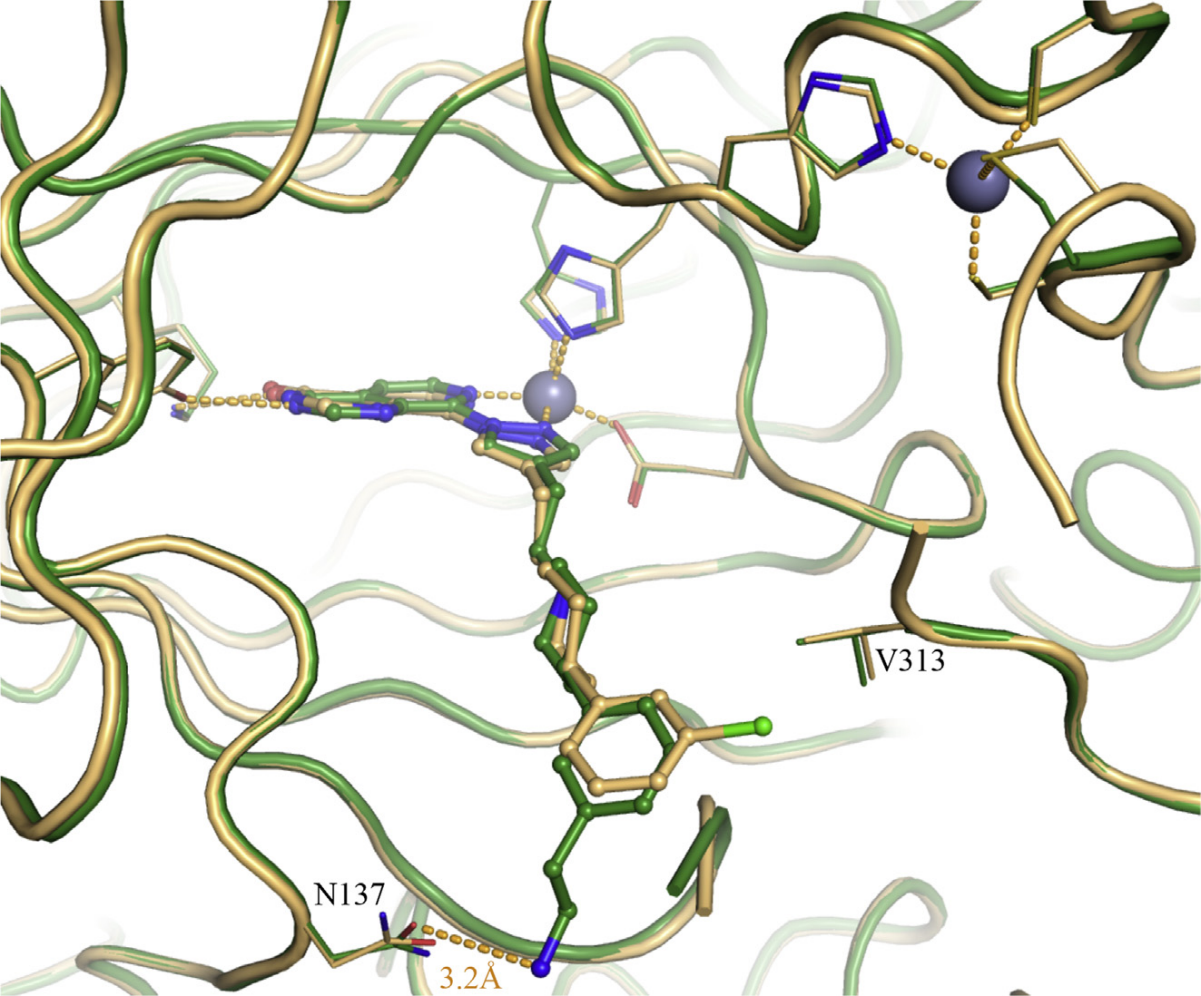
Overlay of crystal structures of **16m** (green) and **16a** (beige) bound to KDM4A. Protein backbone chains are represented as cartoon tubes, key residues are displayed in line representation. Compounds **16m** and **16a** are shown in ball and stick representation. The two methyl groups in **16m** have not been modelled due to too weak density linked to the high mobility of the corresponding atoms. Metal ions are shown as spheres. (For interpretation of the references to colour in this figure legend, the reader is referred to the Web version of this article.)

The introduction of 6-membered heterocyclic substituents (compounds **16g-i**) was tolerated but with no evidence for selective KDM4 subfamily inhibition (Table 1). Some of the most active inhibitors incorporated a basic substituent at the *m*-position of the phenyl ring; notably pyrrolidine and dimethylamino derivatives **16j** and **16m**, respectively (Table 1). Consistent with previous findings [29], these *m*-substituted analogues retained selectivity over other KDM subfamilies; for example, **16j** displayed weaker inhibition of KDM2A (IC_50_ = 1.97 μM), KDM3A (IC_50_ = 1.85 μM), and KDM6B (IC_50_ = 32.26 μM). Likewise, **16b** displayed selectivity over KDM2A (IC_50_ = 4.68 μM), KDM3A (IC_50_ = 6.42 μM), and KDM6B (IC_50_ > 100 μM). The crystal structure of **16m** bound to KDM4A confirmed a direct but suboptimal interaction between the amino group of the *m*-substituent and the side chain of residue N137 (distance of 3.2 Å, Fig. 4), consistent with the improved KDM4A potency of **16m** (0.038 μM) compared to the unsubstituted parent (**54g** in reference 29; KDM4A IC_50_ = 0.456 μM). Overall, **16m** shows a balanced KDM4/KDM5 subfamily inhibitory profile whereas the unsubstituted parent compound (**54g** in reference 29; KDM5B IC_50_ = 0.058 μM) is KDM5B preferring. However, direct interaction of the basic substituent with KDM4A E169 was not observed by X-ray crystallography, likely due to the high mobility of the E169 side chain observed in all crystal structures (no electron density detected). Compounds bearing a lipophilic piperidine 4-substituent (**16b, 16c,** and **16e**) displayed moderate cell permeability (Caco-2 assay, A to B flux) similar to that of **16a** (Table 1). However, the introduction of a heteroaromatic ring (**16h** and **16i**), or the inclusion of an additional basic centre (**16j, 16l,** and **16m)** had a detrimental effect on cell permeability (Table 1).

We subsequently investigated the effect of selected *p*-substituents on the 4-phenylpiperidine moiety. The presence of a 6-membered heterocycle (**17a-c**, Table 2) or alkyl chains bearing a basic centre (**17e** and **17f**, Table 2) elicited a KDM4/5 inhibitory profile similar to their *m*-substituted counterparts with no evidence of clear selective KDM4 over KDM5 inhibition (Table 1, Table 2). However, selectivity for dual KDM4/5 inhibition over other KDM subfamilies was maintained; for example **17f** displayed a weaker inhibitory activity against KDM2A (IC_50_ = 1.42 μM), KDM3A (IC_50_ = 2.02 μM), and KDM6B (IC_50_ = 52.41 μM).

**Fig. 5:**
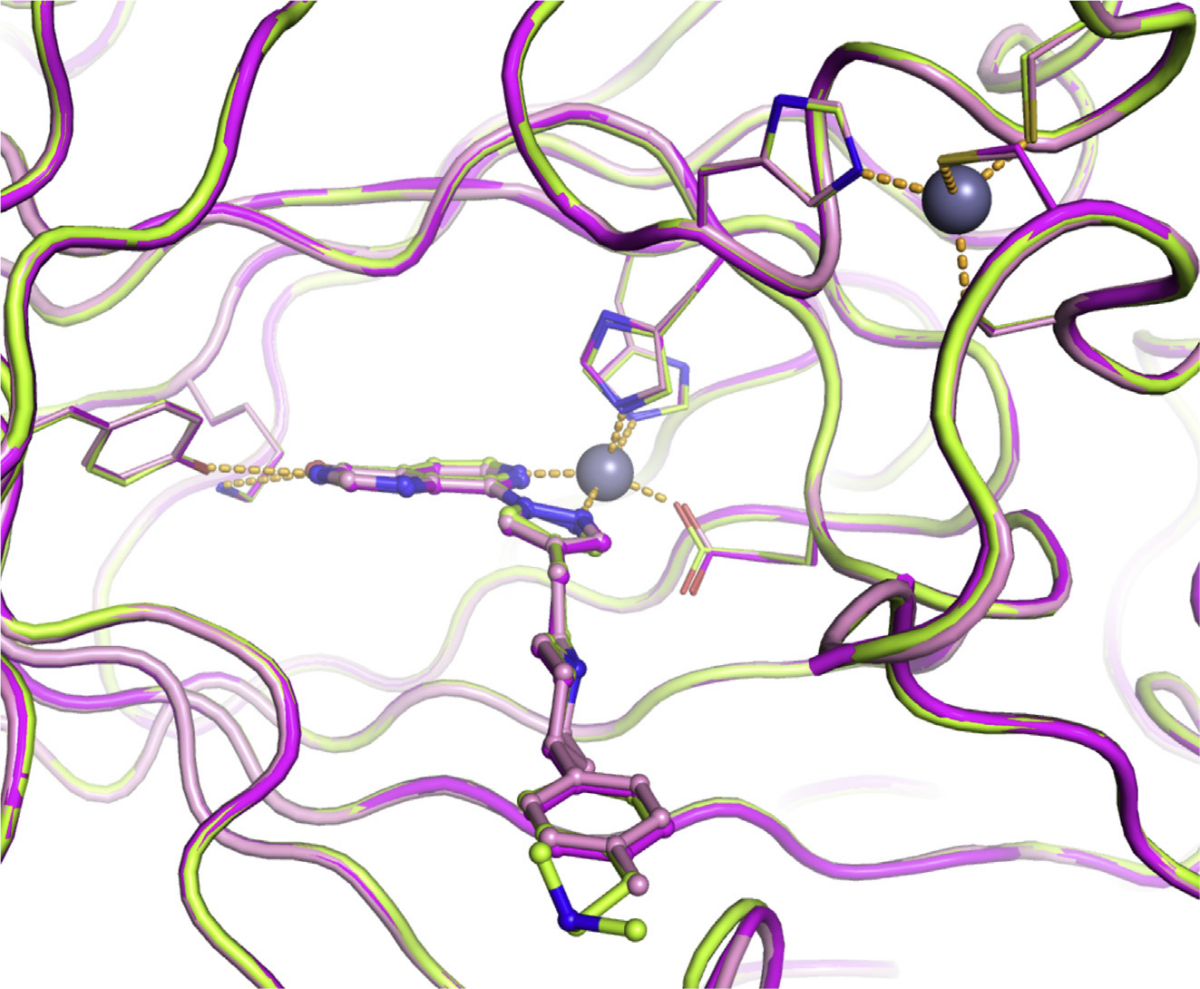
Overlay of crystal structures of **17b** (pink), **17e** (magenta) and **17f** (light-green) bound to KDM4A. Zn(II) atoms are shown as grey spheres. Protein backbone chains are represented as cartoon tubes, key residues are displayed in line representation. The pyridyl ring in **17b** has not been modelled due to too weak density of the corresponding atoms. Compounds **17b**, **17e** and **17f** are shown in ball and stick representation. (For interpretation of the references to colour in this figure legend, the reader is referred to the Web version of this article.)

Crystal structures of **17b**, **17e** and **17f** bound to KDM4A confirmed that the phenyl *p*-substituent points towards the solvent accessible region without making any specific contact with the protein including the targeted residue E169 (Fig. 5). More precisely, the *p*-substituents of both **17b** and **17e** could not be associated with any electron density, suggesting high mobility and lack of stabilisation by the protein. In the **17f**-bound KDM4A structure, the *p*-dimethylamino moiety points towards the solvent accessible region, but without clear interactions with the protein (Fig. 5). Similarly to the *m*-substituted analogues (Table 1), *p*-substituted compounds displayed significantly lower permeability in the Caco-2 assay (A to B flux) relative to that of **16a** (Table 2). This drop in cellular permeability is consistent with increased polarity and/or the presence of a basic nitrogen.

**Fig. 6:**
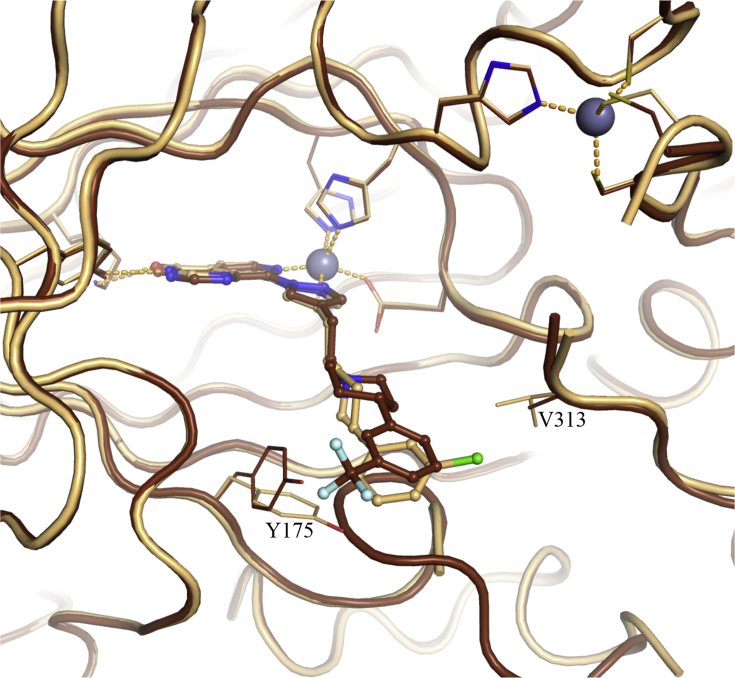
Overlay of crystal structures of **18a** (brown) and **16a** (beige) bound to KDM4A. Zn(II) atoms are shown as grey spheres. Proteins backbone chains are represented as cartoon tubes, key residues are displayed in line representation. Compounds **18a** and **16a** are shown in ball and stick representation. (For interpretation of the references to colour in this figure legend, the reader is referred to the Web version of this article.)

Noting that both *m*-chloro, -fluoro, -trifluoromethyl [29] substituents, and alkyl chains bearing basic centres at the *m-*position of the distal phenyl ring (Table 1) impart potent and balanced KDM4/5 subfamily inhibition, and also that their binding modes are potentially complementary (see overlay of **16a** and **16m**, Fig. 4), we combined these two substituents in a small series of 3,5-disubstituted phenyl derivatives (Table 3). Synthesis of the most desired compounds with both *m*-Cl and *m*-alkylamino side chains proved challenging due to competing dehalogenation upon reduction of the piperidine double bond (Scheme 2). Nevertheless, we were able to combine a *m*-CF_3_ substituent with basic functionality in the remaining *m*-position (**18b,c**; Table 3). This approach led to potent dual KDM4 and KDM5 inhibition but no additive inhibitory effect was observed (Table 3), and the KDM4/5 inhibitory profile was broadly similar to that observed with the corresponding mono-substituted analogues (Table 1, Table 3). We subsequently solved the structure of **18a** bound to KDM4A (Fig. 6). This structure indicated that the pendant phenyl ring is rotated such that the *m*-CF_3_ substituent does not form an interaction with V313 in contrast to its *m*-Cl counterpart (compare Fig. 2, Fig. 4, Fig. 6). This observation could partly explain the lack of additive SAR in compounds **18b** and **18c,** assuming that the *m*-CF_3_ substituent in these 3,5-disubstituted phenyl derivatives occupies the same position as observed for **18a**. Compounds **18b** and **18c** maintained a selective inhibition profile *versus* other KDM subfamily members; for example, **18b** displayed weaker inhibitory activity against KDM2A (IC_50_ = 3.77 μM), KDM3A (IC_50_ = 5.68 μM), and KDM6B (IC_50_ = 23.97 μM). However, both **18b** and **18c** displayed low Caco-2 permeability (A to B flux) in line with previous results obtained with compounds bearing a basic substituent on the phenyl ring (Table 1, Table 2).

We next turned our attention to constraining the C4-pyrazole substituent in order to maintain contact with V313 whilst minimising the entropic cost associated with ligand binding. We envisaged that the lipophilic contact with V313 (KDM4A) (Fig. 2) could be optimised by conformational restriction directing the phenyl ring towards V313. This hypothesis led to synthesis of the spirocyclic analogue **19a** which inhibited KDM4 and KDM5 subfamily members with IC_50_ values similar to those observed with **16a** (Table 1, Table 4). **19a** also displayed selective inhibition of KDM4/5 over other KDM subfamilies, inhibiting KDM2A, KDM3A, and KDM6B with IC_50_ values of 4.50, 5.78 and 90.22 μM, respectively.

**Fig. 7:**
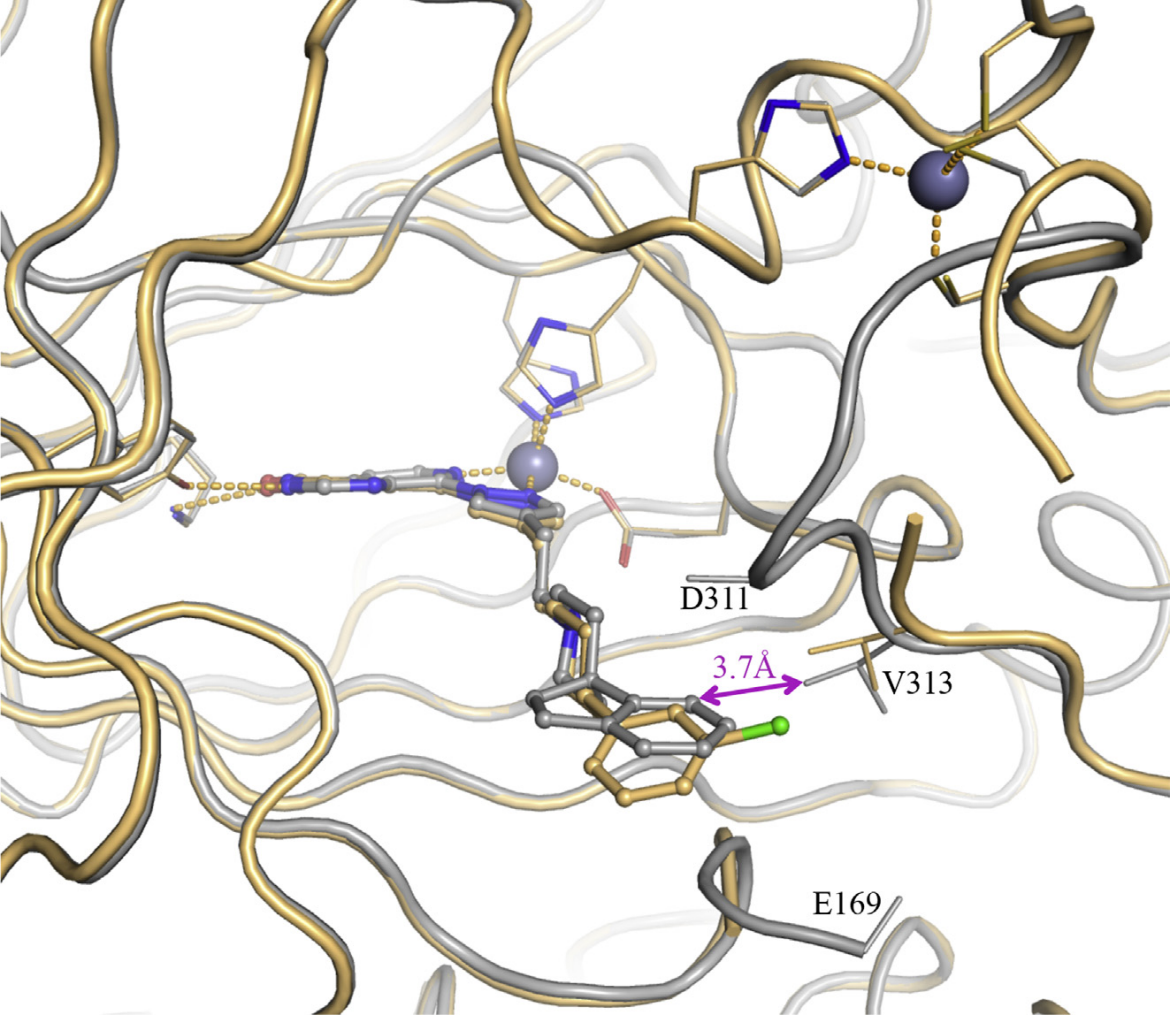
Overlay of crystal structures of **19a** (grey) and **16a** (beige) bound to KDM4A. Zn(II) atoms are shown as spheres. Protein backbone chains are represented as cartoon tubes, key residues are displayed in line representation. Compounds **19a** and **16a** are shown in ball and stick representation.

**Fig. 8:**
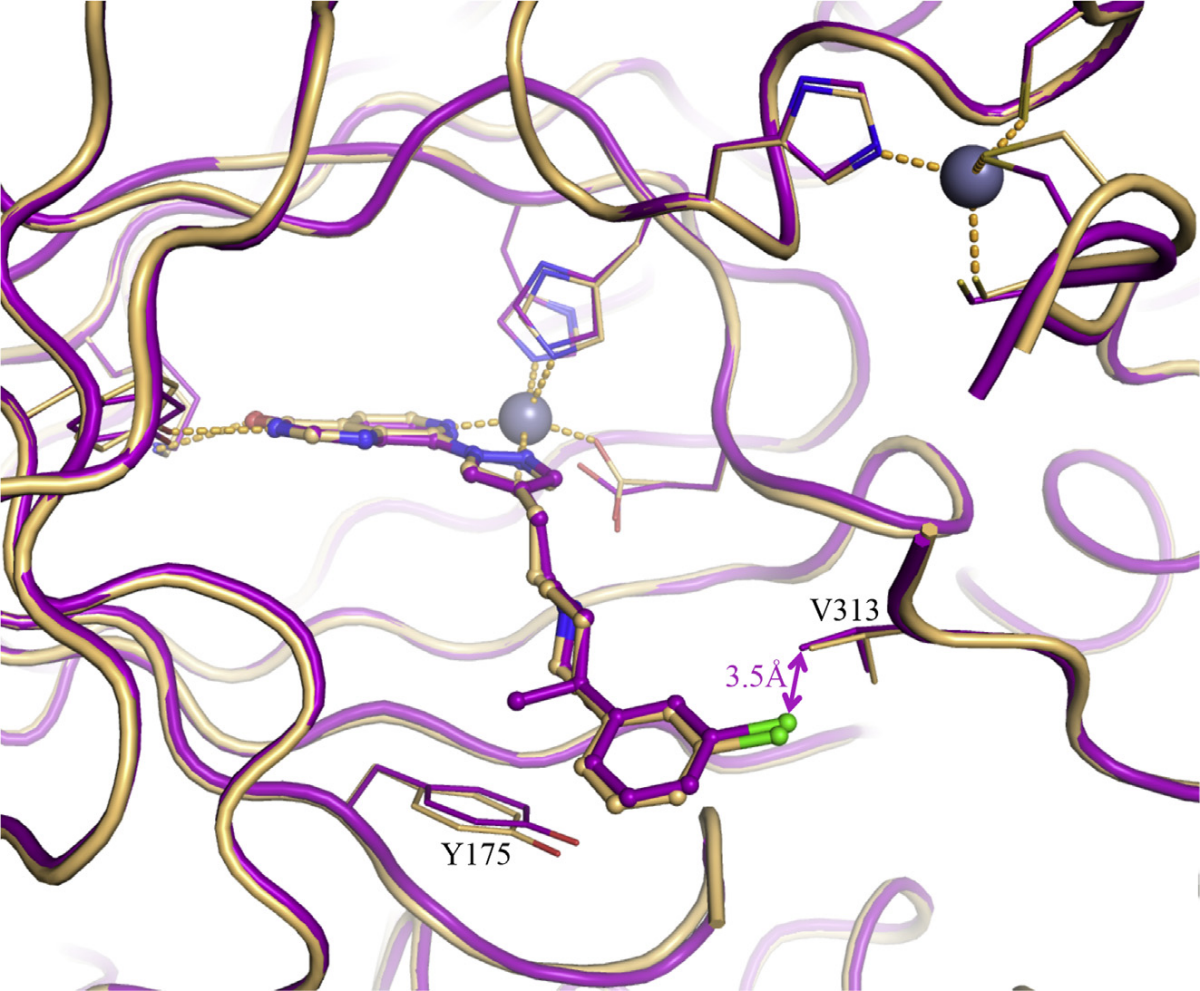
Overlay of crystal structures of **19d** (pink) and **16a** (beige) bound to KDM4A. Zn(II) are shown as spheres. Proteins backbone chains are represented as a cartoon tubes, key residues are displayed in line representation. Compounds **19d** and **16a** are shown in ball and stick representation. (For interpretation of the references to colour in this figure legend, the reader is referred to the Web version of this article.)

The crystal structure of **19a** bound to KDM4A (Fig. 7) revealed a binding mode similar to that of **16a**, with the phenyl ring of the spirocyclic system slightly closer to the side chain of V313 than in **16a** (closest phenyl carbon atom is 3.7 Å from the side chain of V313 for **19a**, versus 4 Å for the corresponding carbon in **16a**). In the **19a**-bound KDM4A crystal structure, we also observed that a loop comprising KDM4A residues 308–313 folds over the conformationally restricted spirocyclic phenyl ring to elicit favourable hydrophobic stacking interactions with both Cα and Cβ atoms of D311. In addition, we also observed electron density for the main chain and Cβ of E169 below the spirocyclic phenyl ring of **19a** − interestingly, E169 is not commonly visible due to both main chain and side chain flexibility. Further, the pyrazole C4-substituent in **19a** is associated with a stronger electron density than for the corresponding **16a** structure, and is well defined in all four chains of the asymmetric unit with B factors significantly lower than for the corresponding atoms in **16a** (average B factors of the terminal phenyl in **19a** is 0.8 times the average B factor for the whole structure, while it was 1.3 times for **16a**). These observations suggest that **19a** is more stably bound in the active site of KDM4A than **16a**. Compounds **19b** and **19c** gave no improvement to KDM4/5 inhibitory profiles relative to **19a** (Table 4); however, comparison of the structures of **19a** and **16a** bound to KDM4A (Fig. 7) prompted us to introduce a methyl group at the piperidine C4-position in **16a** to restrict the conformation without a spirocyclic ring system. Pleasingly, **19d** (Table 4) exhibited a KDM4/5 inhibitory profile similar to that observed with **19a** and the crystal structure of **19d** bound to KDM4A revealed the *m*-Cl phenyl substituent in an equatorial position with the Cl atom making contact with V313 (distance of 3.5 Å, Fig. 8). However, the pyrazole C4-substituent appeared less well stabilised than its equivalent in **19a** (the full substituent is seen only in one chain for **19d**, and with B factors up to 1.3 times higher than the average of the structure). This may arise from freedom of rotation around the piperidine C4-bond in **19d** and **16a** compared to the constrained spirocyclic compound **19a**. Satisfyingly, all analogues in this subseries displayed good permeability in the Caco-2 assay (A to B flux; Table 4).

**Fig. 9:**
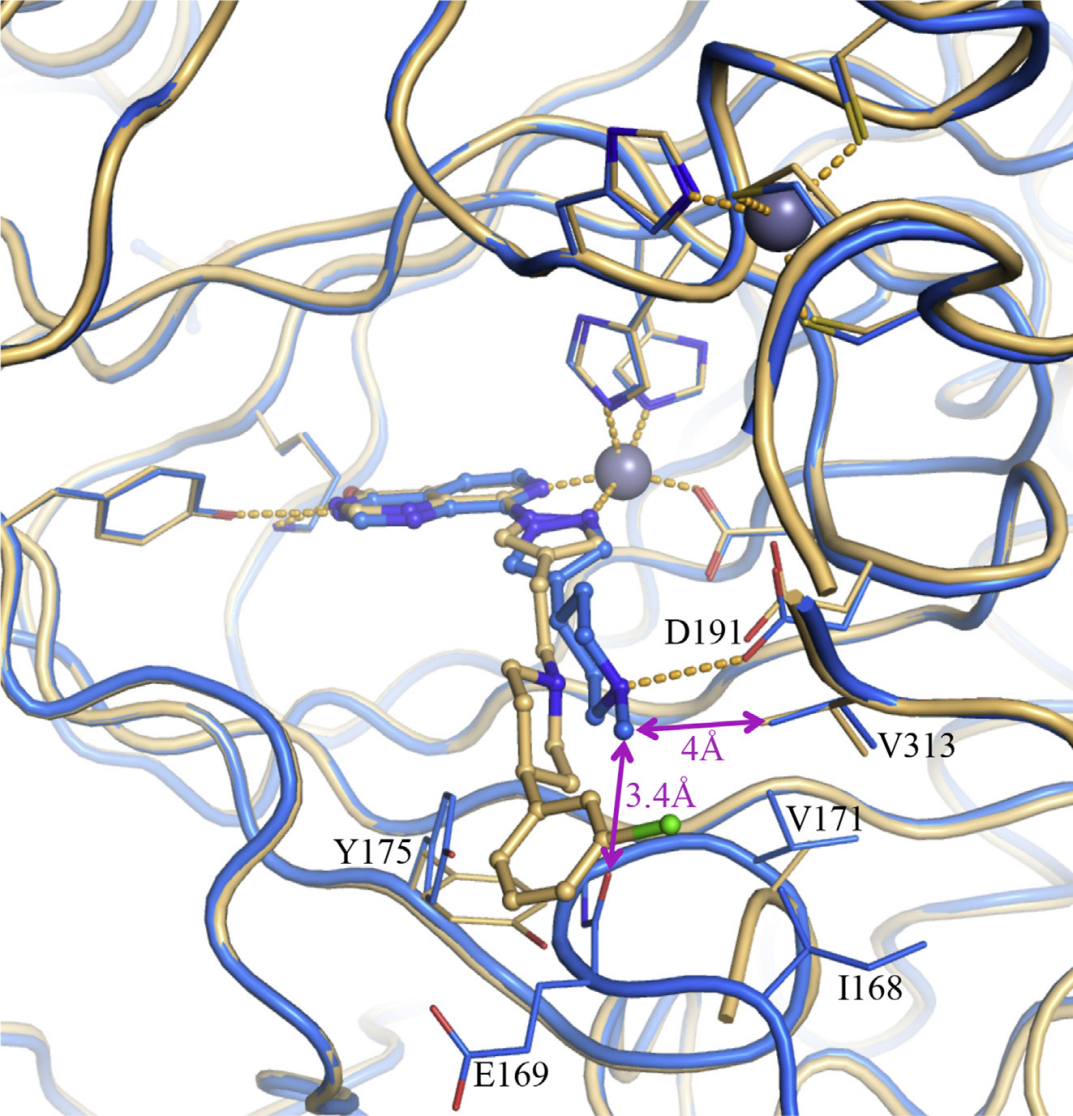
Overlay of crystal structures of **34a** (blue) and **16a** (beige) bound to KDM4A. Zn(II) atoms are shown as spheres. Protein backbone chains are represented as cartoon tubes, key residues are displayed in line representation. Compounds **34a** and **16a** are shown in ball and stick representation. (For interpretation of the references to colour in this figure legend, the reader is referred to the Web version of this article.)

In a further attempt to restrict the conformation of the C4-pyrazole substituent, we directly attached the *N*-methylpiperidin-4-yl moiety to C4 of the pyrazole to give **34a**, a more potent inhibitor of KDM5 relative to KDM4 subfamily enzymes by at least 5-fold (Table 5). A crystal structure of **34a** in complex with KDM4A (Fig. 9) revealed electron density for the pyrazole substituent in all four chains of the asymmetric unit, with the piperidine nitrogen closer to the side chain carboxylate of D191 than with **16a** (2.9 Å versus 4.5 Å for **16a**). In contrast to the **16a-**KDM4A structure, the loop comprising residues 160–171 remained in the conformation observed in the apoprotein structure and no movement was observed for Y175. Interestingly, in the **34a**-bound structure, the piperidine *N*-Me is located in close proximity to V313 (Fig. 9). In an attempt to further understand the KDM5B-preferring activity of **34a** (Table 5), we also solved the crystal structure of **34a** bound to KDM5B (Fig. S5). Given the higher potency of **34a** against KDM5B relative to KDM4A, we were surprised to find that the electron density corresponding to the C4-pyrazole substituent was less well resolved in the **34a**-KDM5B structure, with no interpretable density for the piperidine *N*-Me group (Fig. S5). Furthermore, no interaction was observed with the carboxylic acid of residue D502 (D191 in KDM4A). Analogous to the **34a**-KDM4A structure, residue W486 in KDM5B (Y175 in KDM4A) and surrounding loops adopt the Apo conformation (Fig. 3 and Fig. S5). However, the piperidine ring of **34a** makes a hydrophobic interaction with W486 in KDM5B but does not form productive interactions with the equivalent Y175 residue in the **34a**-KDM4A crystal structure − this may account for the KDM5B-preferring activity profile of **34a**.

Recognising the KDM5B-preferring inhibitory profile of **34a** (Table 5), the close proximity of the *N*-Me group of **34a** to V313, V171 and I168 in KDM4A (Fig. 9) prompted us to introduce bulkier *N*-alkyl substituents to improve lipophilic contact with these residues, aiming for an enhancement in KDM4 potency. This hypothesis led to the synthesis of compounds **34b-h (**Table 5**);** however, most of these analogues displayed only a modest gain in KDM4 inhibitory activity relative to **34a**, with KDM5 inhibition also maintained (Table 5). Weaker inhibition of the other KDM subfamilies was observed; for example, **34f** displayed inhibition of KDM2A (IC_50_ = 12.54 μM), KDM3A (IC_50_ = 6.13 μM), and KDM6B (<60% inhibition at 100 μM).

**Fig. 10:**
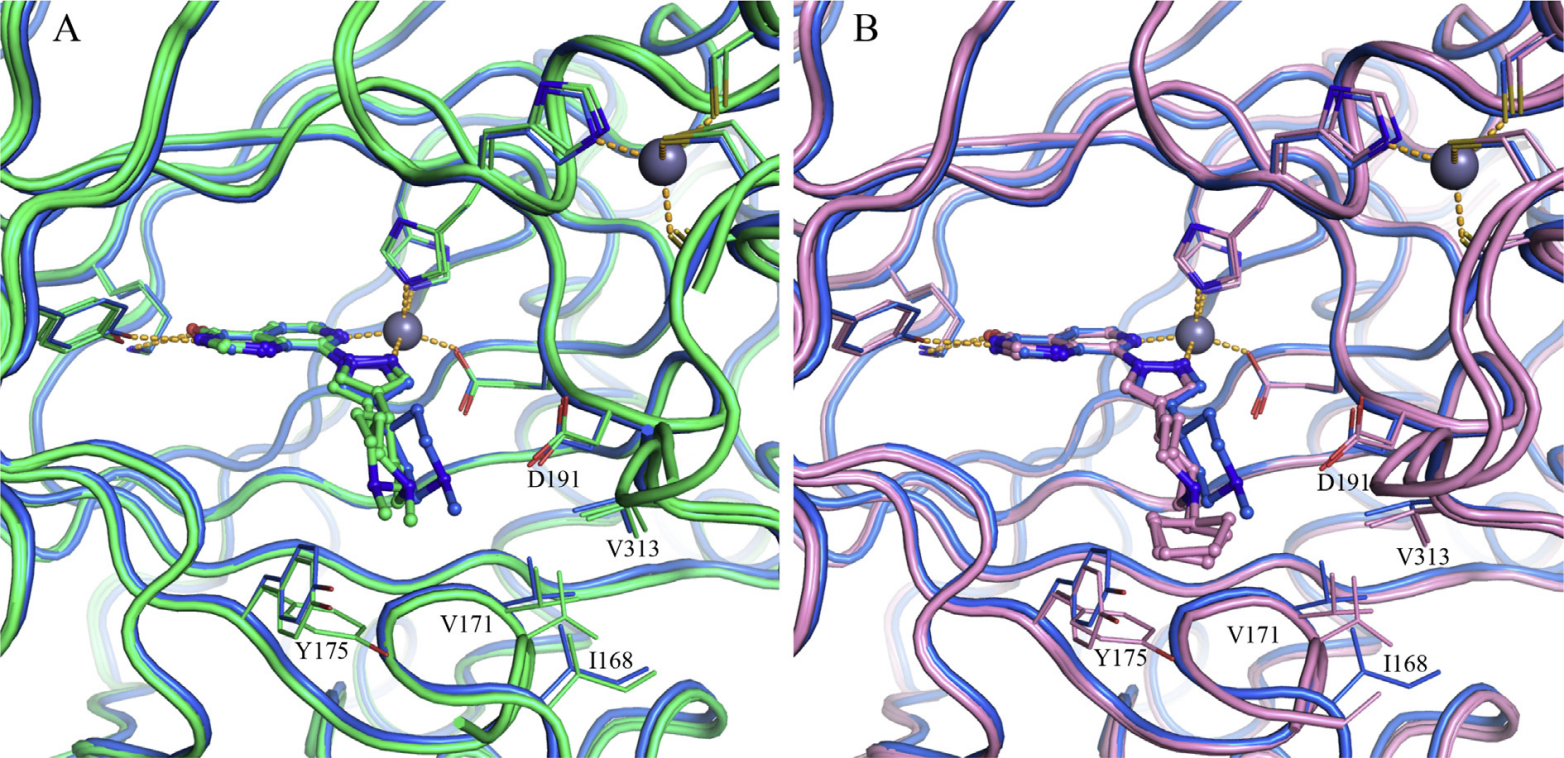
A: Overlay of crystal structures of **34a** (blue) and **34b** (green) bound to KDM4A. Chains A and B of the asymmetric unit are shown for **34b**-KDM4A structure, as they do not show exactly the same conformation for the ligand and its neighbouring protein residues. B: Overlay of crystal structures of **34a** (blue) and **34g** (pink) bound to KDM4A. Chains B and D of the asymmetric unit are shown for **34g**-KDM4A structure, as they do not show exactly the same conformation of the protein. Zn(II) atoms are shown as grey spheres. Protein backbone chains are represented as cartoon tubes, key residues are displayed in line representation. Compounds are shown in ball and stick representation. The distal methyl group in **34b** has not been modelled due to too weak density linked to the high mobility of the corresponding atoms. (For interpretation of the references to colour in this figure legend, the reader is referred to the Web version of this article.)

In an attempt to rationalise the KDM4/5 structure activity relationship (SAR) observations in this subseries, we determined the crystal structures of **34b** and **34g** in complex with KDM4A (Fig. 10A and B, respectively). In both cases, the C4-pyrazole substituent is shifted from the position observed in the **34a**-bound KDM4A structure and the interaction between the piperidine nitrogen and D191 is no longer present. Notably, the pyrazole C4-substituents of both **34b** and **34g** are closer to Y175 and we observe both apo and ligand-induced shifts in the positions of Y175 and nearby loop 161–170. The terminal carbon of the *N*-ethyl substituent of **34b** is not associated with any electron density in the four chains of the asymmetric unit, and the *N*-cyclopentane substituent in **34g** is seen in only two chains; furthermore, interaction with the three targeted residues V313, V171 and I168 is not observed. Thus, a piperidine *N*-substituent bigger than the methyl is sufficient to disrupt the interaction with D191, likely because of steric clashes with the side chain of V313 or the main chain carbonyl of E169 (both of which lie in close proximity to the *N*-Me of **34a**, Fig. 9). These factors may explain the modest potency improvement for compounds **34b-h** compared to **34a**. We also solved the structures of **34f** and **34g** bound to KDM5B, which were nearly identical to the KDM5B-**34a** structure (Fig. S6), with the exception of an extra carbon seen for the piperidine *N*-substituent of **34f**. These structures indicate that the piperidine *N*-substituents of **34f** and **34g** are mobile, and not making specific interactions with the protein, which likely explains the relatively flat SAR for this series of compounds against KDM5B. Based on the above observations, this subseries was not pursued further.

**Fig. 11:**
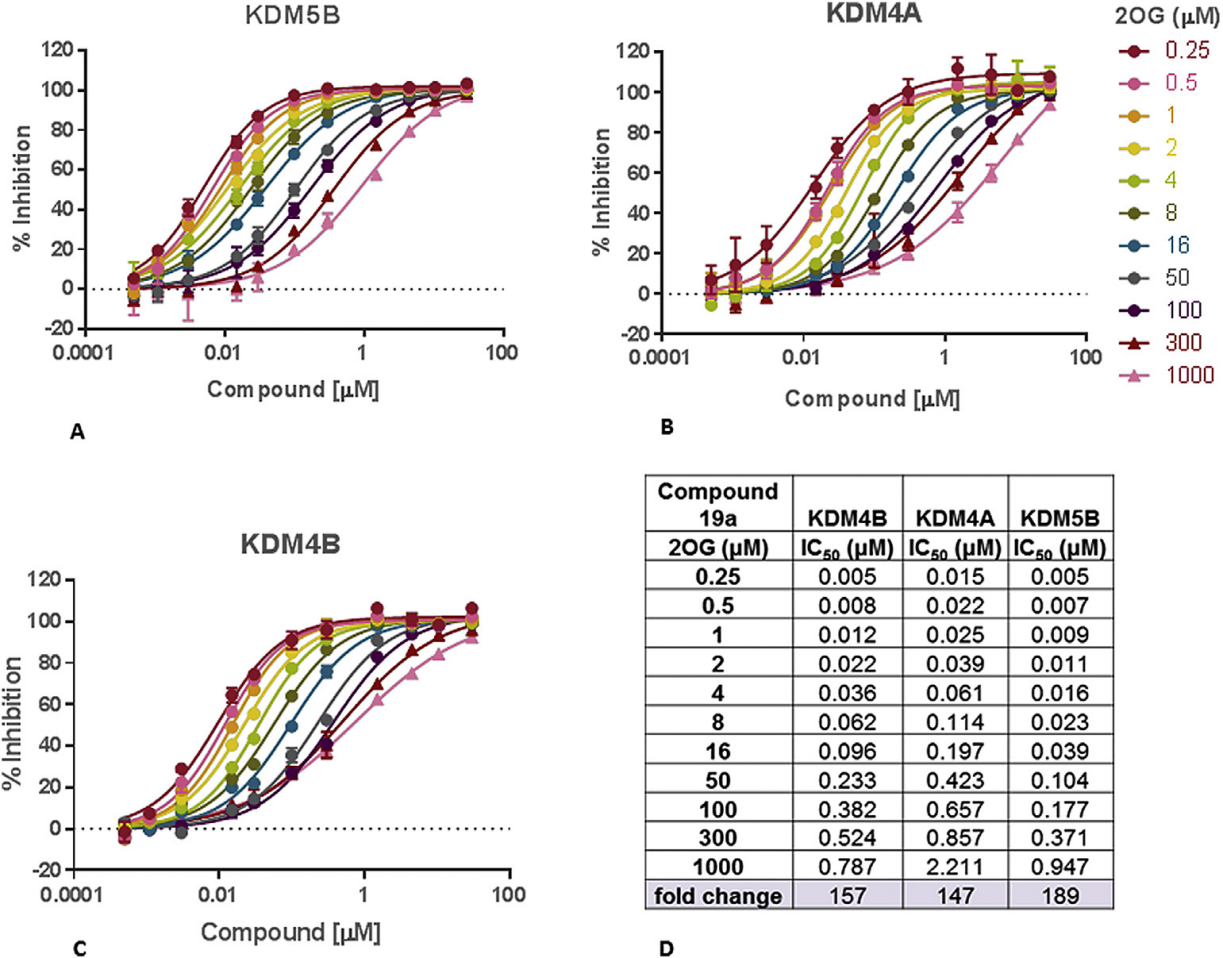
2OG co-substrate competition studies for **19a** in KDM4A, KDM4B and KDM5B biochemical assays.

Overall, our investigations towards selective KDM4 over KDM5 inhibition by targeting residue differences between the histone substrate binding sites afforded potent dual inhibitors of both KDM4 and KDM5 subfamilies in biochemical assays with selectivity *versus* KDM2A, KDM3A, and KDM6B exemplars of other histone demethylase subfamilies. We have previously reported the KDM cellular profiling of **16a**, and that the KDM inhibitory activity of **16a** is dependent upon the 2OG co-substrate concentration in a biochemical assay [38]. We therefore assessed the 2OG-dependence of KDM inhibitory activity for exemplar compounds **16m** (Fig. S7), **19a** (Fig. 11), and **34f** (Fig. S7). For **19a**, we observe a 147-fold drop in KDM4A inhibition with increasing 2OG concentration from 0.25 μM to a physiologically relevant concentration of 1 mM (Fig. 11) [[39], [40], [41]]. Calculated *K*i values [42] (Table 6) demonstrate similar affinity for KDM4A and KDM5B (**16a**, *K*i = 0.003 and 0.002 μM, respectively; **19a**, *K*i = 0.004 and 0.007 μM, respectively).

**Table 6:** KDM4A and KDM5B *K*i values; KDM4A/B and KDM5B target engagement in cells (InCELL Hunter™ and IF assays)^a^.

Compd	*K*i, (μM)		KDM4, EC_50_ (μM)		KDM5B, EC_50_ (μM)	
	KDM4A	KDM5B	KDM4B, InCELL Hunter™	KDM4A, IF assay	InCELL Hunter™	IF assay
**16a**	0.003	0.002	1.0 ± 0.7	8.5 ± 6.1	0.6 ± 0.2	24.2 ± 16.4
**16b**	0.004	0.003	2.0 ± 0.2	not tested	2.9 ± 1.0	not tested
**16h**	0.003	0.001	1.4 ± 0.2	10.4^b^	1.1 ± 0.3	26.0^b^
**16m**	0.002	0.001	3.0 ± 1.6	30.3 ± 23.8	1.4 ± 0.3	3.77^b^
**17f**	0.004	0.004	3.5 ± 0.6	not tested	1.9 ± 0.6	not tested
**19a**	0.004	0.007	1.3 ± 0.3	4.7 ± 3.6	4.0 ± 3.5	13.4 ± 8.5
**19d**	0.005	0.002	1.7 ± 0.6	5.9^b^	1.1 ± 0.5	20.8^b^
**34f**	0.026	0.016	9.1 ± 2.3	38% at 200 μM	6.6 ± 5.0	26.4 ± 4.4

a EC_50_ data are mean values of at least two independent determinations unless specified otherwise.

b Results from a single experiment.

In addition to co-substrate competition, the translation of biochemical inhibitory activity into cellular target engagement can also be influenced by compound cell permeability and residence time. Furthermore, biochemical KDM-subfamily selectivity trends may not be maintained in a cellular environment due to differences in the affinity of KDM subfamilies for 2OG [28,34], and differences in the KDM expression levels in cells [28]. We therefore investigated the cellular target engagement of selected compounds against KDM4A, KDM4B, and KDM5B using two orthogonal assay formats. Firstly, an InCELL Hunter^™^ assay based upon measuring compound-mediated protein stabilisation [43]; secondly, a previously reported immunofluorescence-based (IF) assay whereby cellular KDM4A and KDM5B inhibition is determined by monitoring the levels of H3K9Me_3_ and H3K4Me_3_ histone marks, respectively [38]. Testing of the known selective KDM5-subfamily inhibitor **6** in both the InCELL Hunter™ and IF assays provided KDM5B EC_50_ values of 0.3 and 0.6 μM, respectively, entirely consistent with previously reported KDM5 EC_50_ value of 0.34 μM, based on assessment of H3K4Me_3_ levels in PC9 cells [31].

**Fig. 12:**
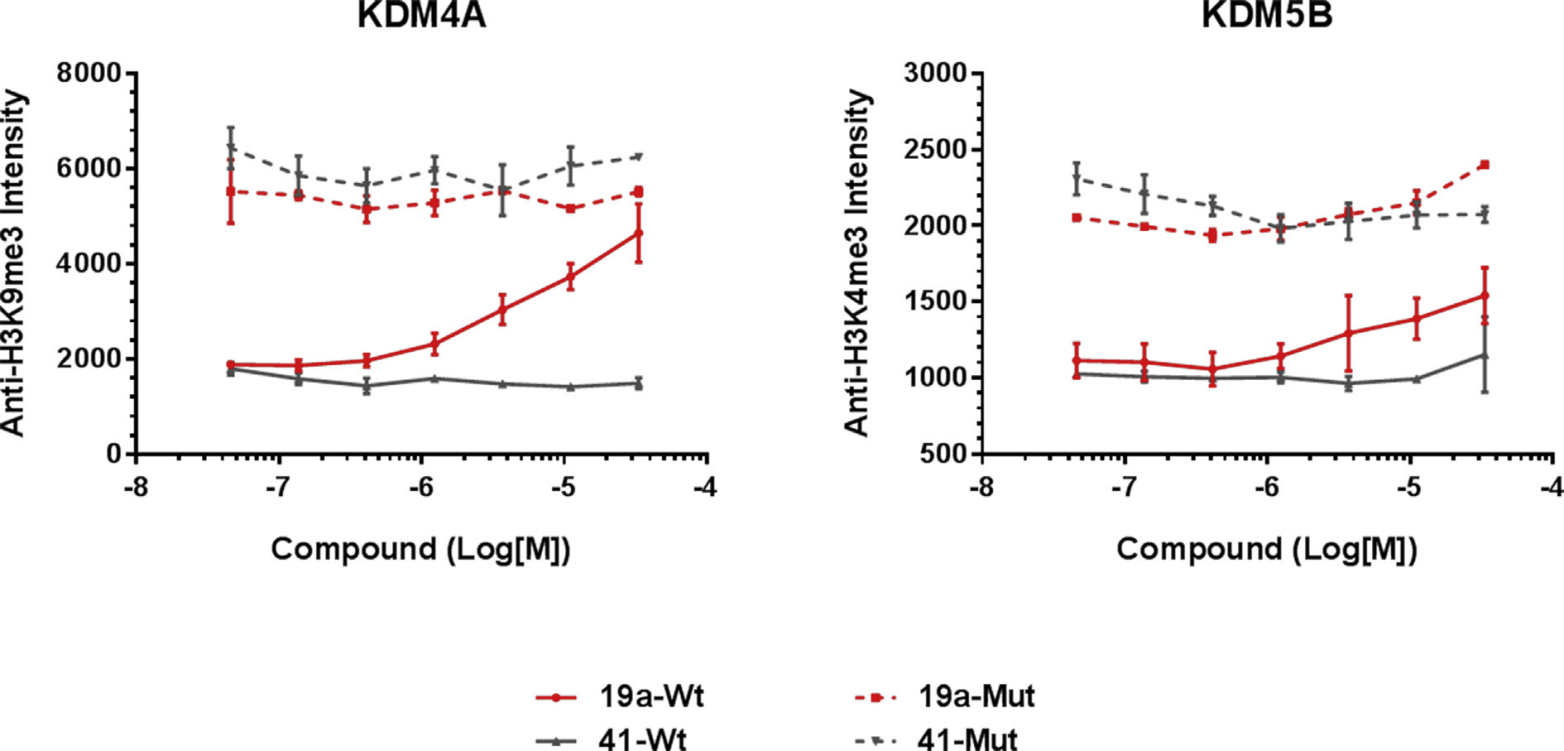
KDM4A and KDM5B target engagement in HeLa cells by **19a** and its matched pair inactive control **41** based on modulation of the relevant histone methyl mark (IF assay). Constructs encoding FLAG-tagged wild type KDM4A or catalytically inactive mutant (H188A/E190A) KDM4A, and wild type KDM5B or catalytically inactive mutant (H499A/E501A) KDM5B were used.

We initially tested compounds in the InCELL Hunter™ assay for KDM4B and KDM5B cellular target engagement with several compounds demonstrating EC_50_ values close to 1 μM (Table 6). Interestingly, compounds **16h**, **16m** and **17f** (Table 1, Table 2), that display low permeability in the Caco-2 assay, showed cellular target engagement suggesting cellular internalisation under the assay conditions. We also profiled compounds in the cellular IF assay (Table 6). Both **19a** and **19d** inhibited KDM4A demethylase activity in cells (EC_50_ = 4.7 and 5.9 μM, respectively), slightly more potently in comparison to KDM5B inhibition (EC_50_ = 13.4 and 20.8 μM, respectively; Table 6). Consistent with our *in vitro* biochemical 2OG competition experiments, we observe a 1175-fold drop in KDM4A biochemical potency to IF cell-based activity for **19a**, a trend that is replicated across all compounds tested (Table 6). Of note, the cellular KDM4A and KDM4B EC_50_ values for **19a** (Table 6) are similar to the KDM4A and KDM4B biochemical IC_50_ values obtained at a physiologically relevant 2OG concentration (approximately 1 mM) [[39], [40], [41]] (IC_50_ = 2.21 μM, and 0.79 μM, respectively, Fig. 11). As a further confirmation of cell-based activity, **19a** was tested in the IF assay alongside its matched pair inactive control **41** (KDM4A, KDM4B and KDM5B *in vitro* biochemical IC_50_ > 15 μM) (Scheme 6, Fig. 12). In this assay format, we created an assay window through overexpression of the catalytically active protein since we were unable to measure changes in endogenous methyl marks. The mutant protein used in the assay is catalytically dead; as such, addition of an inhibitor should have no effect on the methyl marks providing that no modulation of endogenous methyl marks occurs. Pleasingly, compound **41** did not alter levels of H3K9Me_3_ nor H3K4Me_3_ histone methyl marks (Fig. 12) suggesting that elevation of these marks upon incubation of the cells with **19a** is due to cell-based inhibition of KDM4 or KDM5 subfamily enzymes by compound **19a**.

Taken together, these studies suggest that the 2-OG competitive inhibition mode of the C8-substituted pyrido[3,4-*d*]pyrimidin-4(3*H*)-one series is the major contributing factor to the drop off between *in vitro* biochemical and cell-based potency, and that a greater than 10-fold improvement in biochemical KDM4 inhibitory activity would likely be required to achieve sub-micromolar cellular KDM4 inhibition. In addition, if the observed tentative selectivity trends regarding cellular target inhibition are maintained, then a 10-fold KDM4 over KDM5 *in vitro* biochemical selectivity window may be sufficient to provide a 30-fold KDM4 selective inhibitor in cells.

We have previously reported that aldehyde oxidase (AO)-mediated metabolism at C2 of the pyridopyrimidinone scaffold leads to high *in vivo* clearance for this class of compounds, precluding their use in *in vivo* settings [44]. Given these findings, we investigated the stability of the pyridopyrimidinone derivatives in cellular cultures *in vitro*, and found no evidence of cell-based metabolism. For example, incubation of **19a** and its inactive control **41** in human prostate carcinoma LNCaP cells for up to 120 h showed uptake of the parent compounds in a time-dependent manner and stable concentrations in media with no metabolites detected in cells or media culture. We further characterised **19a** by measuring its kinetic aqueous solubility (93.3 μM), human plasma protein binding (89%), and *in vitro* intrinsic microsomal clearance in mouse, rat, and human (7.5, 19.8, and 24.7 μL/min/mg, respectively).

## 4 Conclusion

Following our discovery of C8-pyridopyrimidinone-based analogues **4** and **16a** as selective, dual KDM4 and KDM5 subfamily inhibitors [29], we determined the crystal structures of **16a** in complex with both KDM4A and KDM5B. Both structures reveal that the C8-substituent extends towards the histone substrate binding site and the solvent exposed surface of the enzymes. We identified residues in close proximity to the *m*-Cl phenyl moiety of **16a** that are conserved within, but differ between the KDM4 and KDM5 subfamilies. Targeting these residues, in particular E169 and V313 (KDM4A numbering), did not elicit the desired KDM4 over KDM5 selectivity profile in *in vitro* biochemical assays; likely due to mobility of the histone peptide binding region of both the KDM4A and KDM5B proteins, and conformational flexibility of the synthesised ligands. Nevertheless, many compounds displayed potent and selective KDM4/KDM5 dual inhibition. Conformational constraint of the pyrazole C4-substituent by direct attachment of an *N*-methylpiperidin-4-yl moiety provided **34a**, a KDM5-subfamily preferring inhibitor. Subsequent structure-based design to increase KDM4 potency for this subseries *via* optimising lipophilic interactions with V313 (KDM4A numbering) proved unsuccessful. In an alternative approach, we rigidified the distal part of the flexible pyrazole C4-substituent, and optimised interactions with V313 by introducing a spirocyclic ring system which led to **19a**, a potent, cell-penetrant and selective KDM4/KDM5 dual inhibitor. A crystal structure of **19a** bound to KDM4A confirmed a binding mode broadly similar to that of **16a** and revealed distinct features including an induced-fit movement of loop 308–313 of KDM4A which folds over the phenyl ring of the spirocyclic system to create a hydrophobic stacking interaction with both the main chain and Cβ of D311. Compound cellular profiling in two orthogonal target-engagement assays revealed a significant drop from biochemical to cell-based activity across multiple analogues. Compounds **19a** and **19d** showed single digit μM KDM4A/KDM4B cellular inhibitory activity in the two orthogonal cell-based assay formats. Detailed characterisation of **19a** and additional analogues suggests that the significant drop in potency from *in vitro* biochemical to cell-based target engagement assays can be mainly attributed to the 2OG-competitive mode of inhibition. Taken together, our data suggests that sub-1nM *in vitro* biochemical affinity will be required with this C8-substituted pyridopyrimidinone series in order to achieve sub-μM target inhibition in cells. Achieving sub-1nM KDM4 biochemical potency, together with KDM5-subfamily selectivity *via* pyridopyrimidinone C8-derivatisation, is likely to be challenging. The lessons learned may be applied to a different scaffold to achieve potent and selective KDM4 inhibition.

## 5 Experimental section

**KDM4A and KDM4B AlphaScreen™ biochemical assays.** KDM4A and KDM4B IC_50_ values were determined as previously described [29]. In these assays 2OG co-substrate concentrations were as follows: KDM4A: 10 μM; KDM4B 2 μM.

**KDM5B and KDM5C AlphaScreen™ biochemical assays**: KDM5B and KDM5C IC_50_ values were determined as previously described [29]. In these assays 2OG co-substrate concentrations were as follows: KDM5B: 5 μM; KDM5C: 5 μM.

**KDM2A, KDM3A and KDM6B AlphaScreen™ biochemical assays.** IC_50_ values were determined as previously described [29] and references cited therein. In these assays 2OG co-substrate concentrations were as follows: KDM2A: 10 μM; KDM3A: 5 μM; KDM6B: 10 μM.

### 5.1 Cell-based assays for KDM4A/B and KDM5B target engagement

**IF assay:** Mammalian overexpression constructs encoding full length 1*FLAG-tagged wild type, or catalytically inactive mutant (H188A/E190A), KDM4A were obtained from the Structural Genomics Consortium. Constructs encoding wild type or catalytically inactive mutant (H499A/E501A) KDM5B have been previously described [38].

HeLa cells (ATCC) were routinely cultured at 37 °C, 5% CO_2_ in DMEM (Sigma-Aldrich, UK) supplemented with 10% FBS (Gibco, UK), 1% l-glutamine (Gibco, UK) and 1% non-essential amino acids (Sigma-Aldrich, UK), and passaged regularly using 0.25% Trypsin-EDTA (Sigma-Aldrich, UK) before reaching confluence.

For IF, 6000 cells/well were seeded into a 96-well clear bottom ViewPlate (PerkinElmer, UK) and incubated overnight. DNA overexpression vectors were transfected into cells using HeLaFect transfection reagent (OZBIOSCIENCES, France). Briefly, 0.1 μg/well plasmid DNA and 0.15 μL/well HeLaFect were diluted in separate tubes containing 25 μL/well OptiMEM (Gibco, UK). The two solutions were gently mixed in a 1:1 ratio and incubated for 20 min at room temperature. Following aspiration of the culture medium, the DNA-lipid complex was added to cells and incubated for 4 h at 37 °C, 5% CO_2_. Compounds were prepared in culture medium supplemented with 20% FBS and an appropriate volume of DMSO (Sigma-Aldrich, UK) to maintain solvent concentration. Compounds were serially diluted at a 1:3 to 1:5 dilution ratio to 5X final concentration in a 96-well plate (ThermoFisher Scientific, UK). Compound was then added to cells and incubated for a further 24 h at 37 °C, 5% CO_2_. After 24 h compound incubation, cells were stained for immunofluorescence and analysed as previously described [38]. Immunofluorescence images of 10 fields of view per well were captured through a 20× objective on the PerkinElmer IN Cell 2200 instrument. High-content analysis was performed using INCell Analyzer software (GE Healthcare, UK), and concentration-response curves generated in GraphPad Prism 6 (GraphPad, USA) and Dotmatics (Dotmatics, UK).

**InCELL Hunter™ assay:** KDM4B and KDM5B cellular target engagement assays were set up using the InCell Hunter Target Engagement Kit from DiscoverX (DiscoverX 2018) [43]. For the KDM4B assay, the expressed protein comprised the N-terminal region of KDM4B (aa's 1–348) fused upstream of the F24 mutant (E31G-R71G-K105E) of FKBP1A [45] with an ePL tag (enhanced ProLabel, DiscoverX) appended at the C-terminus. In the KDM5B assay, the N-terminal region (aa's 26–772) of the protein fused with a C-terminal ePL tag was used. Constructs were transfected over 24 h in HEK293T cells using Lipofectamine 3000 (ThermoFisher Scientific). 2.5 × 10^3^ transfected cells/well were re-plated in 384 assay plates before treating with compounds over 6 h. To complete the assay, cells were incubated over 30 min at RT with the InCell Hunter assay reagents mix following the manufacturer's instructions before reading the chemiluminescent signal.

### 5.2 Crystal structure determinations

**KDM4A structures.** A previously established 6His-TEV-KDM4A construct (residues 1–359) [46] was produced in *Escherichia coli* and purified by nickel affinity chromatography, followed by tag removal with TEV protease, reverse nickel affinity, and gel filtration. The protein was stored at −80 °C at 10 mg/mL in a buffer containing 10 mM HEPES pH 7.5 and 200 mM NaCl. Purified KDM4A was crystallized in the apo form at 18 °C using the hanging-drop vapor-diffusion method. The crystallization drops were composed of 1.5 μL of protein (7 mg/mL) and 1.5 μL of reservoir solution containing 14% (w/v) PEG4000 and 0.1 M BTP pH 7.5, placed over 800 μL of reservoir solution. Plate-like crystals typically grew in a week at 18 °C and were soaked by addition of 0.75 μL of compound at 50–200 mM in DMSO directly to the drops, followed by 4–48 h incubation at 18 °C. Crystals were briefly transferred to cryoprotectant solution containing 14% (w/v) PEG4000, 75 mM BTP pH 7.5, and 25% (v/v) glycerol prior to freezing in liquid nitrogen.

**KDM5B structures.** A previously established 6His-TEV-KDM5B construct (residues 26-771Δ101−374) [47] was produced in Sf9 cells and purified by nickel affinity chromatography, followed by tag removal with TEV protease, gel filtration and reverse nickel affinity. Protein was concentrated to 8 mg/mL crystallized at 4 °C using the sitting drop vapor diffusion method. KDM5B was preincubated with 4 mM MnCl_2_ before the protein was transferred to crystallization plates. Crystals were obtained in drops consisting of 100 nL of protein (8 mg/mL), 200 nL of a precipitant consisting of 1.6 M Na/K phosphate, 0.1 M HEPES pH 7.5, and 20 nL of KDM5B seeds from crystals obtained from the same condition. Crystals were grown at 4 °C, then compounds were soaked into apo crystals for 5 min at a final concentration of 5 mM. Crystals were cryoprotected with mother liquor supplemented with 25% ethylene glycol prior to freezing in liquid nitrogen.

**Data collection, processing and refinement:** X-ray data were collected in-house at the Institute of Cancer Research, London, UK, on a Rigaku FRX-AFC11-VariMax Cu-VHF-Pilatus300K, and at Diamond Light Source, Oxfordshire, UK, on beamlines I02, I03, and I04-1. Crystals of KDM4A belonged to the space group P 1 2_1_ 1 and diffracted to a resolution between 2.14 and 2.81 Å. KDM5B crystals belonged to the space group P 6_5_ 2 2 and diffracted to a resolution between of 2.14 and 2.3 Å. Datasets were integrated with XDS [48] and scaled and merged with AIMLESS [49]. Structures were solved by molecular replacement using PHASER [50,51]with the publicly available KDM4A and KDM5B structures (PDB codes 2OQ7 and 5A1F, respectively) [46,47] with ligand and water molecules removed used as molecular replacement models. All protein−ligand structures were manually corrected and rebuilt in COOT [52] and refined with BUSTER [53] in iterative cycles. Ligand restraints were generated with GRADE [54] and MOGUL [55]. The quality of the structures was assessed with MOLPROBITY [56,57]. The data collection and refinement statistics are presented in Supporting Information Table S1.

**Caco-2 permeability:** Caco-2 cellular permeability was determined as previously described [29].

### 5.3 Cell uptake experiment and stability of 19a in cellular culture

LNCaP cells (ATCC) were routinely cultured at 37 °C, 5% CO_2_ in RPMI-1640 (Sigma-Aldrich, UK) supplemented with 10% FBS (Gibco, UK), and passaged regularly using 0.25% Trypsin-EDTA (Sigma-Aldrich, UK) before reaching confluence.

For cell uptake and stability experiments, 1.5 × 10^5^ cells/well were seeded into 6-well plates (Corning, UK) and incubated for 48 h at 37 °C, 5% CO_2_, before treatment with either 10 μM compound or 0.1% DMSO (Sigma-Aldrich, UK) as vehicle control. Following 1, 6, 24, 48, 72 and 120 h incubation, media was collected from cells and immediately frozen at −80 °C. Cells were trypsinised, pelleted and then frozen at −80 °C until analysis.

Cell and tissue culture media extracts were analysed using a Dionex Ultimate 3000 UHPLC system coupled to a Thermo Scientific Q Exactive Plus orbitrap mass spectrometer (Thermo Fisher Scientific Inc., Waltham, USA). Separation of analytes was achieved using an Acquity UPLC HSS T3 column (1.8 μm, 100 × 2.1 mm) (Waters, Elstree, UK) at a temperature of 50 °C and a binary mobile phase gradient at a flow rate of 0.4 mL/min. Initial LC conditions comprised 10% solvent A (0.1% formic acid in water), 90% solvent B (methanol); this was ramped to 95% A at 12 min, immediately returned to initial conditions and held for the remaining 3 min of the method. Sample analysis was by electrospray atmospheric pressure ionization combined with full scan acquisition in positive ion mode. The capillary voltage was 3.5 kV; desolvation gas and capillary temperatures were 450 °C and 275 °C respectively; sheath, aux and sweep gas flow rates were 55, 15 and 3 respectively. Full MS/dd-MS^2^ (full MS scan followed by data dependent MS/MS) and Full MS/AIF (full MS scan followed by all ion fragmentation) workflows were used in combination. Identification/presence of metabolites was undertaken with the aid of the software Compound Discoverer (v2.0.0.303, Thermo Fisher Scientific Inc., Waltham, USA). Raw data files of the incubation time course generated in the acquisition software Chromeleon (v7.2 SR3 (7553)), Thermo Fisher Scientific Inc., Waltham, USA) were input into the targeted workflow.

**Chemistry:** Commercially available starting materials, reagents and anhydrous solvents were used as supplied. Flash column chromatography was performed using Merck silica gel 60 (0.025–0.04 mm). Thin layer chromatography was performed using Merck Millipore TLC silica gel 60 F_254_ aluminium sheets and visualised by UV (254 & 280 nm), iodine and KMnO_4_. Column chromatography was also performed on a FlashMaster personal unit using isolute Flash silica columns or a Biotage Isolera purification system using Biotage KP-SNAP cartridges. Ion exchange chromatography was performed using acidic Isolute Flash SCX-II cartridges or basic Isolute Flash NH_2_ cartridges. ^1^H NMR spectra were recorded on either a Bruker Avance-500 or Bruker Avance-400 NMR machine. Samples were prepared as solutions in a deuterated solvent and referenced to the appropriate internal non-deuterated solvent peak or tetramethylsilane. Chemical shifts were recorded in ppm (*δ*) downfield of tetramethylsilane.

**LC-MS Analysis.** Analysis was performed using an Agilent 1200 series HPLC and diode array detector coupled to a 6210 time of flight mass spectrometer with dual multimode APCI/ESI source.

**Method A:** Analytical separation was carried out at 40 °C on a Merck Chromolith Flash column (RP-18e, 25 × 2 mm) using a flow rate of 1.5 mL/min in a 2 min gradient elution with detection at 254 nm. The mobile phase was a mixture of methanol (solvent A) and water (solvent B), both containing formic acid at 0.1%. Gradient elution was as follows: 5:95 (A/B) to 100:0 (A/B) over 1.25 min, 100:0 (A/B) for 0.5 min, and then reversion back to 5:95 (A/B) over 0.05 min, finally 5:95 (A/B) for 0.2 min.

**Method B:** Analytical separation was carried out at 30 °C on a Merck Chromolith Flash column (RP-18e, 25 × 2 mm) using a flow rate of 0.75 mL/min in a 4 min gradient elution with detection at 254 nm. The mobile phase was a mixture of methanol (solvent A) and water (solvent B), both containing formic acid at 0.1%. Gradient elution was as follows: 5:95 (A/B) to 100:0 (A/B) over 2.5 min, 100:0 (A/B) for 1 min, and then reversion back to 5:95 (A/B) over 0.1 min, finally 5:95 (A/B) for 0.4 min.

Analysis was also performed on a Waters Acquity UPLC and diode array detector coupled to a Waters G2 QToF mass spectrometer fitted with a multimode ESI/APCI source.

**Method C:** Analytical separation was carried out at 30 °C on a Phenomenex Kinetex C18 column (30 × 2.1 mm, 2.6u, 100 A) using a flow rate of 0.5 mL/min in a 2 min gradient elution with detection at 254 nm. The mobile phase was a mixture of methanol (solvent A) and water (solvent B), both containing formic acid at 0.1%. Gradient elution was as follows: 10:90 (A/B) to 90:10 (A/B) over 1.25 min, 90:10 (A/B) for 0.5 min, and then reversion back to 10:90 (A/B) over 0.15 min, finally 10:90 (A/B) for 0.1 min.

**Method D:** Analytical separation was carried out at 30 °C on a Phenomenex Kinetex C18 column (30 × 2.1 mm, 2.6u, 100 A) using a flow rate of 0.3 mL/min in a 4 min gradient elution with detection at 254 nm. The mobile phase was a mixture of methanol (solvent A) and water (solvent B), both containing formic acid at 0.1%. Gradient elution was as follows: 10:90 (A/B) to 90:10 (A/B) over 3 min, 90:10 (A/B) for 0.5 min, and then reversion back to 10:90 (A/B) over 0.3 min, finally 10:90 (A/B) for 0.2 min.

Analysis was also performed on a Waters system equipped with a Waters 2545 binary gradient module, a Waters SQ Detector 2, Waters 2489 UV/visible detector, and a Waters 2424 ELS detector.

**Method E**: Analytical separation was carried out on a Kinetex 5u EVO C18 column (100 mm × 3.0 mm, 100 A) using a flow rate of 2 mL/min in a 3 min gradient elution. The mobile phase was a mixture of 93% H_2_O, 5% acetonitrile, and 2% of 0.5 M ammonium acetate adjusted to pH 6 with glacial acetic acid (solvent A) and 18% H_2_O, 80% acetonitrile, and 2% of 0.5 M ammonium acetate adjusted to pH 6 with glacial acetic acid (solvent B). Gradient elution was as follows: 95:5 (A/B) 0.35 min, 95:5 (A/B) to 5:95 (A/B) over 1 min, 5:95 (A/B) over 0.75 min, and then reversion back to 95:5 (A/B) over 0.1 min and 95:5 (A/B) over 0.8 min.

**LC-HRMS analysis** was performed using either an Agilent 1200 series HPLC and diode array detector coupled to a 6210 time of flight mass spectrometer with dual multimode APCI/ESI source (method B) or a Waters Acquity UPLC and diode array detector coupled to a Waters G2 QToF mass spectrometer fitted with a multimode ESI/APCI source (method D). LC-HRMS method **B** referenced to caffeine [M+H]^+^ 195.087652 or hexakis (2,2-difluroethoxy)phosphazene [M+H]^+^ 622.02896 or hexakis(1*H*,1*H*,3*H*-tetrafluoropentoxy)phosphazene [M+H]^+^ 922.009798. LC-HRMS method D referenced to Leucine Enkephalin fragment ion [M+H]^+^ 397.1876.

Preparative HPLC (for the purification of **34a**) purification was carried out using a Merck Chromolith column (RP18-e 10 × 100 mm) at ambient temperature using a gradient program; detection was by UV–MS; the flow rate was 10 mL/min. The mobile phase used was A: acetonitrile/water = 5/95 with 0.1% NH_4_HCO_3_, B: acetonitrile/water = 80/20 with 0.1% NH_4_HCO_3_.

**General Procedure 1 – Suzuki coupling:** The boronic acid pinacol ester (1 equiv.), aryl halide (1 equiv.) and Pd(dppf)Cl_2_·CH_2_Cl_2_ adduct (0.1 equiv.) were dissolved in a mixture of DME and aqueous sodium carbonate (1 M) in a microwave vial. The vial was sealed, evacuated and backfilled with N_2_. The reaction mixture was heated in the microwave at 120 °C for 45 min and monitored by LCMS. The reaction mixture was concentrated *in vacuo* to give the crude material which was purified by Biotage column chromatography (see individual compounds for details of the eluent used).

**General Procedure 2 – Dihydropyridine hydrogenation and Boc deprotection:** Pd(OH)_2_ on carbon (0.4 equiv.) was added to a solution of dihydropyridine (1 equiv.) in EtOH and hydrochloric acid (1 M) under an atmosphere of N_2_. The reaction mixture was then flushed with H_2_ and stirred at room temperature under an atmosphere of H_2_ (2 atm) for 1 h. The reaction mixture was monitored by LCMS. On completion of the reduction, the reaction mixture was filtered through celite to remove catalyst and concentrated *in vacuo* to obtain the crude product. The crude material was taken up in THF and hydrochloric acid (1 M), stirred at 50 °C for 2 h and monitored by LCMS. On completion of the reaction, the reaction mixture was concentrated *in vacuo* and the residue redissolved in MeOH/CH_2_Cl_2_. The crude material was passed through an SCX-2 cartridge eluting with 1 M NH_3_ in MeOH/CH_2_Cl_2_. The ammoniacal solution was concentrated *in vacuo* to yield the product.

**General Procedure 3 – Amine displacement of mesylate using trimethylamine:** Triethylamine (2 equiv.) was added to a solution of freshly made (from compound **10**) mesylate intermediate (1 equiv.) and amine (1.2 equiv.) in anhydrous DMF under N_2_. The reaction mixture was heated at 50 °C for 15 h and monitored by LCMS. When the reaction had reached completion, the reaction mixture was diluted in H_2_O and extracted three times with EtOAc. The combined organic layers were washed with saturated LiCl solution, saturated brine solution, dried over MgSO_4_ and concentrated *in vacuo* to give the crude material which was purified by Biotage column chromatography (see individual compounds for details of the eluent used).

**General Procedure 4 – SEM Deprotection with 6 M HCl/THF:** Hydrochloric acid (6 M, 60–90 equiv.) was added to a solution of SEM protected material (1 equiv.) in THF (0.1 M). The reaction mixture was stirred at 50–60 °C for between 3 and 8 h and monitored by LCMS. Following completion of the reaction, the reaction mixture was concentrated *in vacuo* and purified by Biotage column chromatography on a KP-NH snap column unless otherwise stated (see individual compounds for details of the eluent used). The product obtained from column chromatography was triturated with Et_2_O to give the pure product.

**General Procedure 5 – Reductive amination:** To a microwave vial was added the required aldehyde (1 equiv.) and corresponding amine (1.5 equiv.) and the flask purged with argon. Anhydrous 1,2-dichloroethane (4 mL) or dichloromethane was then added, the mixture stirred to allow dissolution followed by the addition of sodium triacetoxyborohydride (1.6 equiv.), the vial was capped and the mixture stirred at room temperature. Once the reaction was deemed complete by LCMS analysis, the mixture was directly absorbed onto silica gel and purified by flash column chromatography to afford the requisite amine (workup procedure A). Alternatively, the mixture was concentrated to afford an amorphous solid and passed through a plug of silica eluting with 40% MeOH/CH_2_Cl_2_ to afford an oil which was used without further purification (workup procedure B).

**General Procedure 6 – SEM deprotection with HCl/1,4-dioxane:** To a microwave vial was added the SEM protected starting material (1 equiv), 1,4-dioxane (3 mL) and distilled water (1 mL) followed by the dropwise addition of HCl in dioxane (4 M, 25 equiv.). The vial was capped and the mixture stirred at 50 ^°^C until analysis by LCMS indicated complete conversion to the product. The mixture was then concentrated and passed through an SCX-2 cartridge washing initially with MeOH and then NH_3_/MeOH. The basic wash was concentrated and triturated with Et_2_O to afford a solid. If necessary the solid was further purified by flash column chromatography on a SNAP KP-NH column eluting with 0–40% EtOH/CH_2_Cl_2_.

**General Procedure 7 – Synthesis of pyrazole-piperidines:** 4-(1*H*-pyrazol-4-yl)piperidine (1 equiv.) was dissolved in NMP or DMF over 4 Å mol sieve in an oven-dried flask under a flow of N_2_. Appropriate aldehyde (3–4 equiv.) was added to the solution in one portion and the reaction mixture stirred for 10 min - 6 h (see individual compounds for reaction time) at room temperature. Sodium triacetoxyborohydride (2–4 equiv.) was then added and the reaction mixture was stirred at room temperature under N_2_ for 2 h and monitored by LCMS. On completion of the reaction, the reaction mixture was concentrated *in vacuo* and the residue redissolved in MeOH/CH_2_Cl_2_. The crude material was passed through an SCX-2 cartridge eluting with 1 M NH_3_ in MeOH/CH_2_Cl_2_. The ammoniacal solution was concentrated *in vacuo* to yield the product.

**General Procedure 8 – S**_**N**_**Ar displacement:** Cesium carbonate (1.5 equiv.) and the requisite pyrazole (1.1 equiv.) were added to a microwave vial equipped with a stirrer bar. This was sealed, evacuated and flushed with N_2_. Anhydrous MeCN was added to the vial and the reaction mixture stirred for 20 min under N_2_ at room temperature. The cap was then removed, 8-chloro-3-((2-(trimethylsilyl)ethoxy)methyl)pyrido[3,4-*d*]pyrimidin-4(3*H*)-one (1 equiv.) was added, the vial resealed, evacuated and flushed with N_2_. The reaction mixture was then stirred at reflux for 18 h. Solids were removed by filtration and rinsed with CH_2_Cl_2_ three times. The filtrate was concentrated *in vacuo* to give the crude material which was purified by Biotage column chromatography (see individual compounds for details of the eluent used).

### 5.4 *tert*-Butyl 4-(3-(pyridin-3-yl)phenyl)-3,6-dihydropyridine-1(2*H*)-carboxylate (**22h**)

According to General Procedure 1, *tert*-butyl 4-(4,4,5,5-tetramethyl-1,3,2-dioxaborolan-2-yl)-5,6-dihydropyridine-1(2*H*)-carboxylate (300 mg, 0.970 mmol), 3-(3-bromophenyl)pyridine (227 mg, 0.970 mmol) and Pd(dppf)Cl_2_·CH_2_Cl_2_ adduct (79 mg, 0.097 mmol) were reacted together in DME (3 mL) and aqueous sodium carbonate (1 M, 2 mL). Purification on a KP-Sil snap cartridge (9% [0.2 M NH_3_ in MeOH] in CH_2_Cl_2_) gave the product as a brown oil (324 mg, quant.); ^1^H NMR (500 MHz, CDCl_3_) 1.48 (s, 9H), 2.55 (br s, 2H), 3.64 (t, *J* = 5.3 Hz, 2H), 4.08 (s, 2H), 6.08 (br s, 1H), 7.33 (dd, *J* = 7.9, 4.9 Hz, 1H), 7.36–7.45 (m, 3H), 7.53 (s, 1H), 7.84 (dt, *J* = 7.9, 1.9 Hz, 1H), 8.57 (dd, *J* = 4.8, 1.3 Hz, 1H), 8.82 (br d, *J* = 2.0 Hz, 1H); LC - MS (method C; ESI, *m/z*) *t*_*R*_ = 1.40 min–337 [(M + H)^+^]; HRMS (method D): found 337.1917; calculated for C_21_H_25_N_2_O_2_ (M + H)^+^ 337.1916.

### 5.5 3-(3-(Piperidin-4-yl)phenyl)pyridine (**25h**)

According to General Procedure 2, Pd(OH)_2_ on carbon (134 mg, 0.191 mmol) and *tert*-butyl 4-(3-(pyridin-3-yl)phenyl)-5,6-dihydropyridine-1(2*H*)-carboxylate (161 mg, 0.479 mmol) were reacted together in EtOH (3 mL) and hydrochloric acid (1 M, 1 mL). The crude material from this reaction was then stirred in THF (3 mL) and hydrochloric acid (1 M, 3 mL) and purified by passing through an SCX-2 cartridge eluting with 1 M NH_3_ in MeOH/DCM. The ammoniacal solution was concentrated *in vacuo* to yield the product as an off white solid (61.6 mg, 54%); ^1^H NMR (500 MHz, CDCl_3_) 1.69 (qd, *J* = 12.4, 3.7 Hz, 2H), 1.87 (br d, *J* = 12.7 Hz, 2H), 2.08 (br s, 1H), 2.69 (tt, *J* = 12.4, 3.7 Hz, 1H), 2.76 (td, *J* = 12.2, 2.2 Hz, 2H), 3.20 (br d, *J* = 12.3 Hz, 2H), 7.24–7.29 (m, 1H), 7.34 (ddd, *J* = 7.9, 4.9, 0.8 Hz, 1H), 7.39–7.44 (m, 3H), 7.85 (ddd, *J* = 7.9, 2.2, 1.7 Hz, 1H), 8.57 (dd, *J* = 4.9, 1.7 Hz, 1H), 8.83 (dd, *J* = 2.2, 0.8 Hz, 1H); LC - MS (method C; ESI, *m/z*) *t*_*R*_ = 0.46 min–239 [(M + H)^+^]; HRMS (method D): found 239.1555; calculated for C_16_H_19_N_2_ (M + H)^+^ 239.1548.

### 5.6 8-(4-(2-(4-(3-(Pyridin-3-yl)phenyl)piperidin-1-yl)ethyl)-1*H*-pyrazol-1-yl)-3-((2-(trimethylsilyl)ethoxy)methyl)pyrido[3,4-*d*]pyrimidin-4(3*H*)-one (**12h**)

According to General Procedure 3, triethylamine (0.03 mL, 0.215 mmol), 2-(1-(4-oxo-3-((2-(trimethylsilyl)ethoxy)methyl)-3,4-dihydropyrido[3,4-*d*]pyrimidin-8-yl)-1*H*-pyrazol-4-yl)- ethyl methanesulfonate (50 mg, 0.107 mmol) and 3-(3-(piperidin-4-yl)phenyl)pyridine (38.4 mg, 0.161 mmol) were reacted together in anhydrous DMF (1 mL). Purification on a KP-Sil snap cartridge (9% [0.2 M NH_3_ in MeOH] in CH_2_Cl_2_) gave the product as a pale yellow oil (25.4 mg, 39%); ^1^H NMR (500 MHz, CDCl_3_) 0.01 (s, 9H), 0.95–1.01 (m, 2H), 1.86–1.97 (m, 4H), 2.17–2.26 (m, 2H), 2.59–2.67 (m, 1H), 2.69–2.76 (m, 2H), 2.82–2.89 (m, 2H), 3.19 (br d, *J* = 11.2 Hz, 2H), 3.67–3.72 (m, 2H), 5.46 (s, 2H), 7.29–7.32 (m, 1H), 7.36 (dd, *J* = 4.7, 0.7 Hz, 1H), 7.41–7.45 (m, 2H), 7.46 (br s, 1H), 7.81 (br s, 1H), 7.87 (ddd, *J* = 7.9, 2.2, 1.7 Hz, 1H), 8.07 (d, *J* = 5.1 Hz, 1H), 8.31 (s, 1H), 8.57–8.60 (m, 2H), 8.63 (d, *J* = 5.1 Hz, 1H), 8.84 (br d, *J* = 2.5 Hz, 1H); LC - MS (method C; ESI, *m/z*) *t*_*R*_ = 1.14 min–608 [(M + H)^+^]; HRMS (method D): found 608.3162; calculated for C_34_H_42_N_7_O_2_Si (M + H)^+^ 608.3169.

### 5.7 8-(4-(2-(4-(3-(Pyridin-3-yl)phenyl)piperidin-1-yl)ethyl)-1*H*-pyrazol-1-yl)pyrido[3,4-*d*]pyrimidin-4(3*H*)-one (**16h**)

According to General Procedure 4, 8-(4-(2-(4-(3-(pyridin-3-yl)phenyl)piperidin-1-yl)ethyl)-1*H*-pyrazol-1-yl)-3-((2-(trimethylsilyl)ethoxy)methyl)pyrido[3,4-*d*]pyrimidin-4(3*H*)-one (16 mg, 0.026 mmol) and hydrochloric acid (6 M, 1 mL) were reacted together in THF (1 mL). Purification on a KP-NH snap cartridge (40% EtOH in CH_2_Cl_2_) gave the title product as a white solid (7.8 mg, 62%); ^1^H NMR (500 MHz, DMSO‑*d*_6_) 1.72–1.86 (m, 4H), 2.10 (td, *J* = 11.5, 2.5 Hz, 2H), 2.57–2.65 (m, 3H), 2.73 (t, *J* = 7.3 Hz, 2H), 3.10 (br d, *J* = 11.5 Hz, 2H), 7.33 (br d, *J* = 7.7 Hz, 1H), 7.43 (t, *J* = 7.7 Hz, 1H), 7.47 (ddd, *J* = 7.9, 4.7, 0.8 Hz, 1H), 7.53–7.56 (m, 1H), 7.60 (br t, *J* = 1.6 Hz, 1H), 7.73 (s, 1H), 7.97 (d, *J* = 5.1 Hz, 1H), 8.08 (ddd, *J* = 7.9, 2.3, 1.6 Hz, 1H), 8.29 (s, 1H), 8.44 (s, 1H), 8.54 (d, *J* = 5.1 Hz, 1H), 8.56 (dd, *J* = 4.8, 1.6 Hz, 1H), 8.89 (dd, *J* = 2.3, 0.8 Hz, 1H), 12.80 (br s, 1H); LC - MS (method C; ESI, *m/z*) *t*_*R*_ = 0.69 min (purity: 100%) – 478 [(M + H)^+^]; HRMS (method D): found 478.2373; calculated for C_28_H_28_N_7_O (M + H)^+^ 478.2355.

### 5.8 *tert*-Butyl 4-(4-(pyridin-2-yl)phenyl)-3,6-dihydropyridine-1(2*H*)-carboxylate (**23a**)

According to General Procedure 1, *tert*-butyl 4-(4,4,5,5-tetramethyl-1,3,2-dioxaborolan-2-yl)-5,6-dihydropyridine-1(2*H*)-carboxylate (300 mg, 0.970 mmol), 2-(4-bromophenyl)pyridine (227 mg, 0.970 mmol) and Pd(dppf)Cl_2_·CH_2_Cl_2_ adduct (79 mg, 0.097 mmol) were reacted together in DME (3 mL) and aqueous sodium carbonate (1 M, 2 mL). Purification on a KP-Sil snap cartridge (5% [0.2 M NH_3_ in MeOH] in CH_2_Cl_2_) gave the product as a pale brown solid (96 mg, 29%); ^1^H NMR (500 MHz, CDCl_3_) 1.50 (s, 9H), 2.57 (br s, 2H), 3.66 (br t, *J* = 4.9 Hz, 2H), 4.10 (br s, 2H), 6.13 (br s, 1H), 7.19–7.23 (m, 1H), 7.48 (d, *J* = 8.3 Hz, 2H), 7.71–7.74 (m, 2H), 7.96–7.99 (m, 2H), 8.69 (dt, *J* = 4.9, 1.3 Hz, 1H); LC - MS (method C; ESI, *m/z*) *t*_*R*_ = 1.44 min–337 [(M + H)^+^]; HRMS (method D): found 337.1916; calculated for C_21_H_25_N_2_O_2_ (M + H)^+^ 337.1916.

### 5.9 2-(4-(Piperidin-4-yl)phenyl)pyridine (**26a**)

According to General Procedure 2, Pd(OH)_2_ on carbon (15.2 mg, 0.108 mmol) and *tert*-butyl 4-(4-(pyridin-2-yl)phenyl)-5,6-dihydropyridine-1(2*H*)-carboxylate (91 mg, 0.271 mmol) were reacted together in EtOH (3 mL) and hydrochloric acid (1 M, 0.5 mL). The crude material from this reaction was then stirred in THF (2 mL) and hydrochloric acid (1 M, 2 mL) and then purified by passing through an SCX-2 cartridge eluting with 1 M NH_3_ in MeOH/CH_2_Cl_2_. The ammoniacal solution was concentrated *in vacuo* to yield the product as pale yellow oil (46.9 mg, 73%); ^1^H NMR (500 MHz, CDCl_3_) 1.68 (qd, *J* = 12.5, 3.8 Hz, 2H), 1.87 (br d, *J* = 12.8 Hz, 2H), 1.99 (br s, 1H), 2.68 (tt, *J* = 12.1, 3.6 Hz, 1H), 2.76 (td, *J* = 12.1, 2.4 Hz, 2H), 3.21 (br d, *J* = 12.1 Hz, 2H), 7.20 (ddd, *J* = 6.7, 4.8, 1.9 Hz, 1H), 7.32–7.35 (m, 2H), 7.69–7.75 (m, 2H), 7.91–7.96 (m, 2H), 8.66–8.69 (m, 1H); LC - MS (method C; ESI, *m/z*) *t*_*R*_ = 0.47 min–239 [(M + H)^+^]; HRMS (method D): found 239.1545; calculated for C_16_H_19_N_2_ (M + H)^+^ 239.1548.

### 5.10 8-(4-(2-(4-(4-(Pyridin-2-yl)phenyl)piperidin-1-yl)ethyl)-1*H*-pyrazol-1-yl)-3-((2-(trimethylsilyl)ethoxy)methyl)pyrido[3,4-*d*]pyrimidin-4(3*H*)-one (**13a**)

According to General Procedure 3, triethylamine (0.05 mL, 0.367 mmol), 2-(1-(4-oxo-3-((2-(trimethylsilyl)ethoxy)methyl)-3,4-dihydropyrido[3,4-*d*]pyrimidin-8-yl)-1*H*-pyrazol-4-yl)- ethyl methanesulfonate (85 mg, 0.183 mmol) and 2-(4-(piperidin-4-yl)phenyl)pyridine (43.7 mg, 0.183 mmol) were reacted together in anhydrous DMF (1 mL). Purification on a KP-Sil snap cartridge (8% [0.2 M NH_3_ in MeOH] in CH_2_Cl_2_) gave the product as a pale yellow oil (65 mg, 58%); ^1^H NMR (500 MHz, CDCl_3_) 0.01 (s, 9H), 0.96–1.01 (m, 2H), 1.86–1.98 (m, 4H), 2.16–2.27 (m, 2H), 2.57–2.66 (m, 1H), 2.70–2.76 (m, 2H), 2.83–2.90 (m, 2H), 3.20 (br d, *J* = 11.1 Hz, 2H), 3.67–3.72 (m, 2H), 5.46 (s, 2H), 7.21 (ddd, *J* = 6.7, 4.8, 1.6 Hz, 1H), 7.63 (br d, *J* = 8.2 Hz, 2H), 7.70–7.76 (m, 2H), 7.82 (s, 1H), 7.94 (d, *J* = 8.2 Hz, 2H), 8.07 (d, *J* = 5.0 Hz, 1H), 8.31 (s, 1H), 8.60 (s, 1H), 8.63 (d, *J* = 5.0 Hz, 1H), 8.67–8.69 (m, 1H); LC - MS (method C; ESI, *m/z*) *t*_*R*_ = 1.17 min–478 [(M−SEM + H)^+^]; HRMS (method D): found 478.2351; calculated for C_28_H_28_N_7_O (M−SEM + H)^+^ 478.2355.

### 5.11 8-(4-(2-(4-(4-(Pyridin-2-yl)phenyl)piperidin-1-yl)ethyl)-1*H*-pyrazol-1-yl)pyrido[3,4-*d*]pyrimidin-4(3*H*)-one (**17a**)

According to general procedure 4, 8-(4-(2-(4-(4-(pyridin-2-yl)phenyl)piperidin-1-yl)ethyl)-1*H*-pyrazol-1-yl)-3-((2-(trimethylsilyl)ethoxy)methyl)pyrido[3,4-*d*]pyrimidin-4(3*H*)-one (25.4 mg, 0.042 mmol) and hydrochloric acid (6 M, 1 mL) were reacted together in THF (1 mL). Purification on a KP-NH snap cartridge (40% EtOH in CH_2_Cl_2_) gave the title product as a white solid (19.2 mg, 96%); ^1^H NMR (500 MHz, DMSO‑*d*_6_) 1.72 (qd, *J* = 12.3, 3.3 Hz, 2H), 1.78–1.84 (m, 2H), 2.07–2.14 (m, 2H), 2.54–2.64 (m, 3H), 2.70–2.75 (m, 2H), 3.10 (br d, *J* = 11.0 Hz, 2H), 7.32 (ddd, *J* = 7.4, 4.8, 1.1 Hz, 1H), 7.38 (br d, *J* = 8.3 Hz, 2H), 7.73 (s, 1H), 7.86 (td, *J* = 7.6, 1.8 Hz, 1H), 7.91–7.94 (m, 1H), 7.98 (d, *J* = 5.1 Hz, 1H), 8.01 (d, *J* = 8.3 Hz, 2H), 8.29 (s, 1H), 8.45 (s, 1H), 8.54 (d, *J* = 5.1 Hz, 1H), 8.63–8.66 (m, 1H), 12.80 (br s, 1H); LC - MS (method C; ESI, *m/z*) *t*_*R*_ = 0.72 min (purity: >98%) – 478 [(M + H)^+^]; HRMS (method D): found 478.2356; calculated for C_28_H_28_N_7_O (M + H)^+^ 478.2355.

### 5.12 8-(4-(2-(2,3-Dihydrospiro[indene-1,4′-piperidin]-1′-yl)ethyl)-1*H*-pyrazol-1-yl)-3-((2-(trimethylsilyl)ethoxy)methyl)pyrido[3,4-*d*]pyrimidin-4(3*H*)-one

According to general procedure 3, triethylamine (0.03 mL, 0.237 mmol), 2-(1-(4-oxo-3-((2-(trimethylsilyl)ethoxy)methyl)-3,4-dihydropyrido[3,4-*d*]pyrimidin-8-yl)-1*H*-pyrazol-4-yl)- ethyl methanesulfonate (80 mg, 0.172 mmol) and 2,3-dihydrospiro[indene-1,4′-piperidine] (38.6 mg, 0.206 mmol) were reacted together in anhydrous DMF (1 mL). Purification on a silica column eluting with 3% [7 M NH_3_ in MeOH] in CH_2_Cl_2_ gave the product as a pale yellow oil (42 mg, 44%); ^1^H NMR (500 MHz, CDCl_3_) 0.02 (s, 9H), 0.96–1.01 (m, 2H), 1.63 (d, *J* = 12.4 Hz, 2H), 1.99–2.03 (m, 4H), 2.34 (br s, 2H), 2.78 (br s, 2H), 2.87–2.95 (m, 4H), 3.09 (br s, 2H), 3.68–3.74 (m, 2H), 5.48 (s, 2H), 7.16–7.26 (m, 4H), 7.82 (s, 1H), 8.07 (d, *J* = 5.1 Hz, 1H), 8.32 (s, 1H), 8.61 (s, 1H), 8.64 (d, *J* = 5.1 Hz, 1H); LC - MS (method C; ESI, *m/z*) *t*_*R*_ = 1.25 min–557 (M + H)^+^.

### 5.13 8-(4-(2-(2,3-Dihydrospiro[indene-1,4′-piperidin]-1′-yl)ethyl)-1*H*-pyrazol-1-yl)pyrido[3,4-*d*]pyrimidin-4(3*H*)-one (**19a**)

According to general procedure 4, 8-(4-(2-(2,3-dihydrospiro[indene-1,4′-piperidin]-1′-yl)ethyl)-1*H*-pyrazol-1-yl)-3-((2-(trimethylsilyl)ethoxy)methyl)pyrido[3,4-*d*]pyrimidin-4(3*H*)-one (42 mg, 0.075 mmol) and hydrochloric acid (6 M, 1 mL) were reacted together in THF (1 mL) for 4 h. Purification was achieved by passing the crude product through an SCX-2 cartridge eluting first with methanol and then 7 N ammonia in methanol. Fractions containing the product were combined, concentrated *in vacuo*, and the residue triturated with Et_2_O. The beige precipitate was obtained by filtration, and dried (31 mg, 96%). ^1^H NMR (500 MHz, DMSO‑*d*_6_): 1.48 (d, *J* = 12.5 Hz, 2H), 1.86 (td, *J* = 13.2, 4.3 Hz, 2H), 1.98 (t, *J* = 7.3 Hz, 2H), 2.31 (br s, 2H), 2.65–2.80 (m, 4H), 2.85 (t, *J* = 7.3 Hz, 2H), 2.95–3.06 (m, 2H), 7.11–7.21 (m, 4H), 7.75 (s, 1H), 7.99 (d, *J* = 5.1 Hz, 1H), 8.29 (s, 1H), 8.44 (s, 1H), 8.56 (d, *J* = 5.1 Hz, 1H), 12.59 (br s, 1H); HRMS (method D): *t*_*R*_ = 1.83 min (purity:100%) – found: 427.2233; calculated for C_25_H_27_N_6_O (M + H)^+^ 427.2246.

### 5.14 1-Ethyl-4-(1*H*-pyrazol-4-yl)piperidine (**32b**)

According to General Procedure 7, 4-(1*H*-pyrazol-4-yl)piperidine (81 mg, 0.536 mmol) and acetaldehyde (0.1 mL, 1.78 mmol) were reacted together in DMF (5 mL) for 10 min. Sodium triacetoxyborohydride (454 mg, 2.14 mmol) was then added. On completion of the reaction, the reaction mixture was concentrated *in vacuo* and the residue redissolved in MeOH/CH_2_Cl_2_. The crude material was passed through an SCX-2 cartridge eluting with 1 M NH_3_ in MeOH/CH_2_Cl_2_. The ammoniacal solution was concentrated *in vacuo* to yield the crude product that was used in the next step without further purification.

### 5.15 8-(4-(1-Ethylpiperidin-4-yl)-1*H*-pyrazol-1-yl)-3-((2-(trimethylsilyl)ethoxy)methyl)pyrido[3,4-*d*]pyrimidin-4(3*H*)-one (**33b**)

According to General Procedure 8, 8-chloro-3-(2-trimethylsilylethoxymethyl)pyrido[3,4-*d*]pyrimidin-4-one (125 mg, 0.401 mmol), 1-ethyl-4-(1*H*-pyrazol-4-yl)piperidine (77.4 mg, 0.432 mmol) and cesium carbonate (211 mg, 0.648 mmol) were reacted together in anhydrous MeCN (3 mL). Purification on a KP-Sil snap cartridge (15% [0.2 M NH_3_ in MeOH] in CH_2_Cl_2_) gave the product as a pale yellow oil (76.8 mg, 39%); ^1^H NMR (500 MHz, CDCl_3_) 0.00 (s, 9H), 0.95–1.00 (m, 2H), 1.19 (t, *J* = 7.4 Hz, 3H), 1.90 (qd, *J* = 12.3, 2.8 Hz, 2H), 2.02–2.10 (m, 2H), 2.20 (t, *J* = 11.3 Hz, 2H), 2.57 (q, *J* = 7.4 Hz, 2H), 2.63–2.71 (m, 1H), 3.14 (br d, *J* = 11.3 Hz, 2H), 3.66–3.71 (m, 2H), 5.46 (s, 2H), 7.79 (s, 1H), 8.06 (d, *J* = 5.0 Hz, 1H), 8.30 (s, 1H), 8.54 (s, 1H), 8.61 (d, *J* = 5.0 Hz, 1H); LC - MS (method C; ESI, *m/z*) *t*_*R*_ = 1.12 min–455 [(M + H)^+^]; HRMS (method D): found 455.2590; calculated for C_23_H_35_N_6_O_2_Si (M + H)^+^ 455.2591.

### 5.16 8-(4-(1-Ethylpiperidin-4-yl)-1*H*-pyrazol-1-yl)pyrido[3,4-*d*]pyrimidin-4(3*H*)-one (**34b**)

According to General Procedure 4, 8-[4-(1-ethyl-4-piperidyl)pyrazol-1-yl]-3-(2-trimethylsilylethoxymethyl)pyrido[3,4-*d*]pyrimidin-4-one (38.2 mg, 0.084 mmol) and hydrochloric acid (6 M, 1 mL) were reacted together in THF (1 mL). Purification on a KP-NH snap cartridge (40% EtOH in CH_2_Cl_2_) gave the title product as a white solid (23.8 mg, 87%); ^1^H NMR (500 MHz, DMSO‑*d*_6_) 1.02 (t, *J* = 7.2 Hz, 3H), 1.58 (qd, *J* = 12.3, 3.4 Hz, 2H), 1.88–1.94 (m, 2H), 1.98 (t, *J* = 11.6 Hz, 2H), 2.35 (q, *J* = 7.2 Hz, 2H), 2.51–2.56 (m, 1H), 2.96 (br d, *J* = 11.6 Hz, 2H), 7.73 (s, 1H), 7.97 (d, *J* = 5.0 Hz, 1H), 8.28 (s, 1H), 8.37 (s, 1H), 8.52 (d, *J* = 5.0 Hz, 1H), 12.70 (br s, 1H); LC - MS (method C; ESI, *m/z*) *t*_*R*_ = 0.57 min (purity:>98%) – 325 [(M + H)^+^]; HRMS (method D): found 325.1787; calculated for C_17_H_21_N_6_O (M + H)^+^ 325.1777.

## Conflicts of interest

The Institute of Cancer Research operates a rewards to inventors scheme applicable to all current and former employees. JB is a former employee of the Institute of Cancer Research, and is a current employee and stock holder of NeoPhore and Azeria Therapeutics.

## Accession codes

Atomic coordinates and structure factors for the crystal structures of KDM4A with compounds **18a**, **16a**, **34a**, **17f, 16m**, **19a**, **34b**, **34g**, **19d**, **17b**, and **17e** can be accessed using PDB codes 6H4O, 6H4P, 6H4Q, 6H4R, 6H4S, 6H4T, 6H4U, 6H4V, 6H4W, 6H4X and 6H4Y, respectively. Atomic coordinates and structure factors for the crystal structures of KDM5B with compounds **16a**, **34a**, **34f** and **34g** can be accessed using PDB codes 6H4Z, 6H50, 6H51 and 6H52. Authors will release the atomic coordinates and experimental data upon article publication.

## Appendix A Supplementary data

The following is the Supplementary data to this article:Experimental procedures for compounds **12b-g, 12i-m, 13b-g, 14b,c, 15b-d, 16b-g, 16i-m, 17b-g, 18b,c, 19b-d, 22e-g, 22j, 22l,m, 25e-g, 25i,j, 25l,m, 23e-g, 26b-g, 24b,c, 27b,c, 29, 30, 32c-h, 33a, 33c-h, 34a, 34c-h, 36, 37, 40, 41,** Figures S1-S7, and summary crystallographic analyses of compounds **16a**, **16m, 17b, 17e, 17f, 18a, 19a, 19d, 34a, 34b, 34f, 34g** (Table S1)
