# Supplementary material for: C8-substituted pyrido[3,4-d]pyrimidin-4(3H)-ones: Studies towards the identification of potent, cell penetrant Jumonji C domain containing histone lysine demethylase 4 subfamily (KDM4) inhibitors, compound profiling in cell-based target engagement assays
Source: Eur J Med Chem. 2019 Sep 1;177:316–37. doi: 10.1016/j.ejmech.2019.05.041 (PMC6580095; doi:10.1016/j.ejmech.2019.05.041)

C8-Substituted pyrido[3,4-*d*]pyrimidin-4(3*H*)-ones: Studies towards the identification of potent, cell penetrant Jumonji C domain containing histone lysine demethylase 4 subfamily (KDM4) inhibitors, compound profiling in cell-based target engagement assays

Yann-Vaï Le Bihan,[1,⊥] Rachel M. Lanigan,[1,⊥] Butrus Atrash,[1] Mark G. McLaughlin,[1] Srikannathasan Velupillai,[2] Andrew G. Malcolm,[1] Katherine S. England,[2,3] Gian-Filippo Ruda,[2] N. Yi Mok,[1] Anthony Tumber,[2,3] Kathy Tomlin,[1] Harry Saville,[1] Erald Shehu,[1] Craig McAndrew,[1] LeAnne Carmichael,[1] James M. Bennett,[2,3] Fiona Jeganathan,[1] Paul Eve,[1] Adam Donovan,[1] Angela Hayes,[1] Francesca Wood,[1] Florence I. Raynaud,[1] Oleg Fedorov,[2,3] Paul E. Brennan,[2,3] Rosemary Burke,[1] Rob L. M. van Montfort,[1] Olivia W. Rossanese,[1] Julian Blagg,*[,1] Vassilios Bavetsias*[,1]

*corresponding author

⊥ authors contributed equally

[1] Cancer Research UK Cancer Therapeutics Unit, The Institute of Cancer Research, London.

[2] Structural Genomics Consortium (SGC), University of Oxford, ORCRB Roosevelt Drive, Oxford OX3 7DQ, UK.

[3] Target Discovery Institute (TDI), Nuffield Department of Medicine, University of Oxford, NDMRB, Roosevelt Drive, Oxford OX3 7FZ, UK.

S2–S81: Experimental procedures for compounds and preparation of intermediates for the synthesis of **12b-g, 12i-m, 13b-g, 14b,c, 15b-d, 16b-g, 16i-m, 17b-g, 18b,c, 19b-d, 22e-g, 22j, 22l,m, 25e-g, 25i,j, 25l,m, 23e-g, 26b-g, 24b,c, 27b,c, 29, 30, 32c-h, 33a, 33c-h, 34a, 34c-h, 36, 37, 40** and **41.**

S82-S87: Figures S1, S2, S3, S4, S5, and S6

S88–S92: Table S1: Crystallographic data collection and refinement statistics for compounds

S93: Figure S7: KDM4A and KDM4B inhibition by compounds **16m** and **34f** (in vitro biochemical assay): 2OG co-substrate competition studies.

S94-S99: ^{1}H-NMR spectra of representative compounds

Total number of pages: 99

E-mail: vassilios.bavetsias@icr.ac.uk; julian.blagg@icr.ac.uk;

8-(4-(2-(4-(3-Fluorophenyl)piperidin-1-yl)ethyl)-1*H*-pyrazol-1-yl)-3-((2-(trimethylsilyl)ethoxy)methyl)pyrido[3,4-*d*]pyrimidin-4(3*H*)-one (12b)

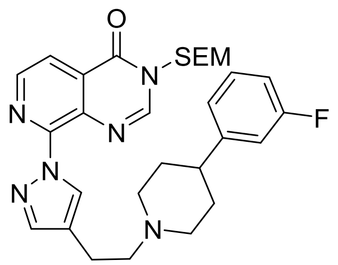

According to General Procedure 3, triethylamine (0.03 mL, 0.215 mmol), 2-(1-(4-oxo-3-((2-(trimethylsilyl)ethoxy)methyl)-3,4-dihydropyrido[3,4-*d*]pyrimidin-8-yl)-1*H*-pyrazol-4-yl)-ethyl methanesulfonate (50.0 mg, 0.107 mmol) and 4-(3-fluorophenyl)piperidine (28.9 mg, 0.161 mmol) were reacted together in anhydrous DMF (1.5 mL). Purification on a KP-Sil

snap cartridge (5% [0.2 M NH_3 in MeOH] in CH_2Cl_2) gave the desired product (32.3 mg, 55%); ^{1}H NMR (500 MHz, $CDCl_3$) 0.01 (s, 9H), 0.96-1.01 (m, 2H), 1.77 - 1.92 (m, 4H), 2.17 (t, *J* = 10.8 Hz, 1H), 2.50-2.59 (m, 1H), 2.67-2.74 (m, 2H), 2.81-2.88 (m, 2H), 3.17 (br d, *J* = 10.8 Hz, 2H), 3.67-3.72 (m, 2H), 5.47 (s, 2H), 6.90 (td, *J* = 8.7, 2.5 Hz, 1H), 6.95 (dt, *J* = 10.2, 1.7 Hz, 1H), 7.02 (d, *J* = 7.6 Hz, 1H), 7.23 - 7.29 (m, 1H), 7.81 (s, 1H), 8.07 (d, *J* = 5.1 Hz, 1H), 8.31 (s, 1H), 8.58 (s, 1H), 8.63 (d, *J* = 5.1 Hz, 1H); LC - MS (method C; ESI, *m/z*) t_R = 1.19 min – 549 [$(M+H)^+$].

8-(4-(2-(4-(3-Fluorophenyl)piperidin-1-yl)ethyl)-1*H*-pyrazol-1-yl)pyrido[3,4-*d*]pyrimidin-4(3*H*)-one (16b)

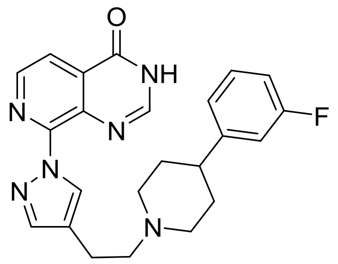

According to General Procedure 4, 8-(4-(2-(4-(3-fluorophenyl)piperidin-1-yl)ethyl)-1*H*-pyrazol-1-yl)-3-((2-(trimethylsilyl)ethoxy)methyl)pyrido[3,4-*d*]pyrimidin-4(3*H*)-one (30.1 mg, 0.055 mmol) and hydrochloric acid (6 M, 1 mL) were reacted together in THF (1 mL). Purification on a KP-NH snap cartridge (40% EtOH in CH_2Cl_2) to give the title product as a white solid (20.1 mg, 88%); ^{1}H NMR (500 MHz, DMSO-d_6) 1.67 (qd, *J* = 12.4, 3.6 Hz, 2H), 1.74-1.81 (m, 2H), 2.07 (td, *J* = 11.8, 1.8 Hz, 2H), 2.52-2.61 (m, 3H), 2.71 (t, *J* = 7.8 Hz, 2H), 3.06 (br d, *J* = 11.3 Hz, 2H), 6.98-7.03 (m, 1H), 7.07-7.13 (m, 2H), 7.30-7.36 (m, 1H), 7.73 (s, 1H), 7.98 (d, *J* = 5.2 Hz, 1H), 8.29 (s, 1H), 8.42 (s, 1H), 8.55 (d, *J* = 5.2 Hz, 1H), 12.77 (br s, 1H); ^{13}C NMR (150 MHz, DMSO-d_6) 21.9, 33.3, 42.1, 54.0, 59.5, 113.2 (d, J_{CF} = 20.8 Hz), 113.9 (d, J_{CF} = 20.4 Hz), 118.3, 121.0, 123.3 (d, J_{CF} = 2.5 Hz), 130.6 (d, J_{CF} = 8.2 Hz), 131.15, 131.5, 136.3, 142.2, 144.5, 148.0, 148.2, 149.8 (d, J_{CF} = 6.5 Hz), 160.2, 162.8

(d, J_{CF} = 242.8 Hz); LC - MS (method C; ESI, *m/z*) t_R = 0.81 min – 419 [(M+H)$^+$]; HRMS (method D): found 419.1993; calculated for $C_{23}H_{24}FN_6O$ (M+H)$^+$ 419.1996.

8-(4-(2-(4-(3-(*tert*-Butyl)phenyl)piperidin-1-yl)ethyl)-1*H*-pyrazol-1-yl)-3-((2-(trimethylsilyl)ethoxy)methyl)pyrido[3,4-*d*]pyrimidin-4(3*H*)-one (12c)

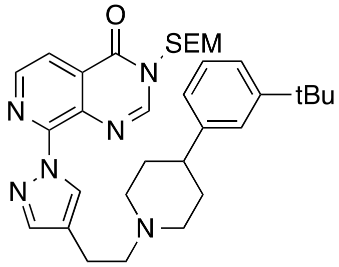

According to General Procedure 5, 2-(1-(4-oxo-3-((2-(trimethylsilyl)ethoxy)methyl)-3,4-dihydropyrido[3,4-*d*]pyrimidin-8-yl)-1*H*-pyrazol-4-yl)acetaldehyde (81 mg, 0.210 mmol), 4-(3-(*tert*-butyl)phenyl)piperidine (46 mg, 0.210 mmol) and sodium triacetoxyborohydride (71 mg, 0.336 mmol) were reacted together in 1,2-dichloroethane (4 mL). Following workup procedure B, the title compound was obtained (30 mg, 24%) as a yellow semi-solid and was used without further purification. LC- MS (method A; ESI, *m/z*)) t_R = 1.51 min - 587.3473 (M+H)$^+$.

8-(4-(2-(4-(3-(*tert*-Butyl)phenyl)piperidin-1-yl)ethyl)-1*H*-pyrazol-1-yl)pyrido[3,4-*d*]pyrimidin-4(3*H*)-one (16c)

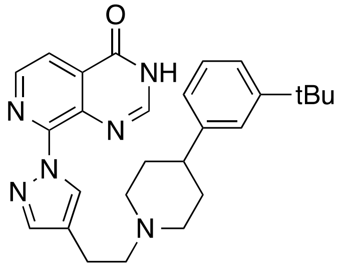

According to General Procedure 6, 8-(4-(2-(4-(3-(*tert*-butyl)phenyl)piperidin-1-yl)ethyl)-1*H*-pyrazol-1-yl)-3-((2-(trimethylsilyl)ethoxy)methyl)pyrido[3,4-*d*]pyrimidin-4(3*H*)-one (30 mg,

0.051 mmol) was reacted with HCl in 1,4-dioxane (4M, 0.128 mL, 0.511 mmol) in 1,4 dioxane/water at 50 °C for 12 h. After workup and flash column chromatography eluting with 0-30% EtOH in CH_2Cl_2, the product was obtained as a white solid (9 mg, 39%); ^{1}H NMR (500 MHz, CD_3OD) 1.32 (s, 9H), 1.84-1.93 (m, 4H), 2.35-2.43 (m, 2H), 2.54-2.69 (m, 1H), 2.81-2.96 (m, 4H), 3.25-3.30 (m, 2H), 7.05-7.33 (m, 4H), 7.83 (br s, 1H), 8.01 (br s, 1H), 8.27 (br s, 1H), 8.55 (br s, 1H), 8.78 (br s, 1H); LC- HRMS (method B; ESI, *m/z*) t_R = 2.38 min - HRMS: found 457.2683; calculated for $C_{27}H_{33}N_6O$ $(M+H)^+$ 457.2710.

8-(4-(2-(4-(3-Methoxyphenyl)piperidin-1-yl)ethyl)-1*H*-pyrazol-1-yl)-3-((2-(trimethylsilyl)ethoxy)methyl)pyrido[3,4-*d*]pyrimidin-4(3*H*)-one (12d)

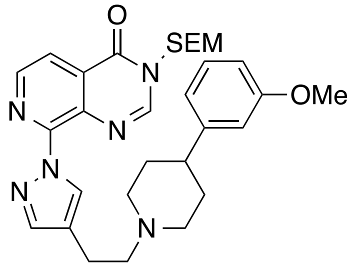

According to General Procedure 3, 2-(1-(4-oxo-3-((2-(trimethylsilyl)ethoxy)methyl)-3,4-dihydropyrido[3,4-*d*]pyrimidin-8-yl)-1*H*-pyrazol-4-yl)ethyl methanesulfonate (98 mg, 0.210 mmol), 4-(3-methoxyphenyl)piperidine (60 mg, 0.316 mmol) and triethylamine (0.073 mL, 0.526 mmol) in DMF was reacted at 50 °C overnight. After workup, the oil obtained was purified by flash column chromatography on a SNAP KP-Sil column eluting with 0-40% EtOH/ CH_2Cl_2 to afford the title compound as a pale yellow oil (21 mg, 18%); ^{1}H NMR (500 MHz, CD_3OD) 0.01 (s, 9H), 0.93-1.01 (m, 2H), 1.76-1.91 (m, 4H), 2.26 (dt, *J* =12.0, 2.9 Hz, 2H), 2.53-2.60 (m, 1H), 2.72-2.77 (m, 2H), 2.85-2.90 (m, 2H), 3.17-3.23 (m, 2H), 3.73-3.77 (m, 2H), 3.78 (s, 3H), 5.49 (s, 2H), 6.75 (ddd, *J = 8.2, 2.5,* 1.0 Hz, 1H), 6.80 (br t, *J* =2.2 Hz, 1H), 6.83 (br d, *J* = 7.6 Hz, 1H), 7.20 (t, *J* = 7.9 Hz, 1H), 7.80 (s, 1H), 8.07 (d, *J* = 5.10 Hz,

1H), 8.45 (s, 1H), 8.58 (d, *J* = 5.0 RHz, 1H), 8.78 (s, 1H); LC- HRMS (Method B; ESI, *m/z*) t_R = 2.77 min - HRMS: found 561.2971; calculated for $C_{30}H_{41}N_6O_3Si$ $(M+H)^+$ 561.3004.

8-(4-(2-(4-(3-Methoxyphenyl)piperidin-1-yl)ethyl)-1*H*-pyrazol-1-yl)pyrido[3,4-*d*]pyrimidin-4(3*H*)-one (16d)

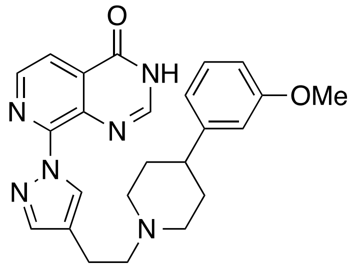

According to General Procedure 6, 8-(4-(2-(4-(3-methoxyphenyl)piperidin-1-yl)ethyl)-1*H*-pyrazol-1-yl)-3-((2-(trimethylsilyl)ethoxy)methyl)pyrido[3,4-*d*]pyrimidin-4(3*H*)-one (21 mg, 0.037 mmol) was reacted with HCl in 1,4-dioxane (4 M, 0.094 mL, 0.374 mmol) in 1,4 dioxane/water at 50 °C for 12 h. After workup and flash column chromatography the product was obtained as a white solid (10 mg, 62%). ^{1}H NMR (500 MHz, CD_3OD) 1.80-1.95 (m, 4H), 2.31-2.40 (m, 2H), 2.56-2.64 (m, 1H), 2.79-2.85 (m, 2H), 2.87.2.94 (m, 2H), 3.23-3.28 (m, 2H), 3.79 (s, 3H), 6.76 (dd, *J* =7.9, 1.9 Hz, 1H), 6.80-6.82 (m, 1H), 6.84 (d, *J* = 7.9 Hz, 1H), 7.21 (t, *J* = 7.9 Hz, 1H), 7.81 (s, 1H), 8.05 (d, *J* = 5.1 Hz, 1H), 8.27 (s, 1H), 8.54 (br s 1H), 8.77 (s, 1H); LC- HRMS (Method D; ESI, *m/z*) t_R = 1.61 min - HRMS: found 431.2191; calculated for $C_{24}H_{27}N_6O_2$ $(M+H)^+$ 431.2195

***Tert*-butyl 4-(3-isopropylphenyl)-5,6-dihydropyridine-1(2*H*)-carboxylate (22e)**

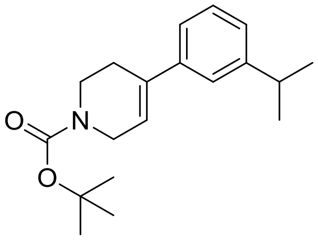

According to General Procedure 1, *tert*-butyl 4-(4,4,5,5-tetramethyl-1,3,2-dioxaborolan-2-yl)-5,6-dihydropyridine-1(2*H*)-carboxylate (340 mg, 1.10 mmol), 1-bromo-3-isopropylbenzene (200 mg, 1.0 mmol) and $Pd(dppf)Cl_2.CH_2Cl_2$ (30 mg, 0.04 mmol) were reacted together in DME (3 mL) and aqueous sodium carbonate (1 M, 2 mL). Purification on a silica column eluting with 3% methanol in CH_2Cl_2 gave the product as a colourless oil (228 mg, 69%). ^{1}H NMR (500 MHz, $CDCl_3$) 1.27 (d, J = 6.9 Hz, 6H), 1.52 (s, 9H), 2.56 (br s, 2H), 2.93 (septet, J = 6.9 Hz, 1H), 3.66 (t, J = 5.7 Hz, 2H), 4.07-4.12 (m, 2H), 6.04 (br s, 1H), 7.25-7.27 (m, 4H); LC - MS (method C; ESI, *m/z*) t_R = 1.66 min – 324 $(M+Na)^+$.

4-(3-Isopropylphenyl)piperidine (25e)

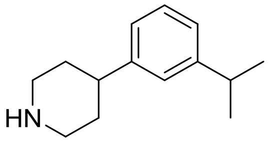

A solution of *tert*-butyl 4-(3-isopropylphenyl)-5,6-dihydropyridine-1(2*H*)-carboxylate (228 mg, 0.756 mmol) in ethanol (10 mL) was stirred in the presence of 10% palladium on carbon under an atmosphere of hydrogen for 2 h. The reaction mixture was filtered through a pad of celite and the crude residue passed through an isolute SCX-2 cartridge to give the reduced product (225 mg, 98%). This material (225 mg, 0.742 mmol) was dissolved in dry dioxane (4 mL) and HCl in dioxane (4 M; 10 mL) and the reaction mixture was stirred at room temperature for 2 h. The solution was then concentrated in vacuo, and the residue filtered through an isolute SCX-2 cartridge to give the title compound as a colourless oil (110 mg, 72% over two steps). ^{1}H NMR (500 MHz, $CDCl_3$) 1.26 (d, J = 6.9 Hz, 6H), 1.66-1.69 (m,

2H), 1.84-1.87 (m, 2H), 2.62 (tt, J = 12.2, 3.7 Hz, 1H), 2.75 (td, J = 12.3, 2.5 Hz, 2H), 2.91 (septet, J = 7.0 Hz, 1H), 3.17-3.22 (m, 2H), 7.06 -7.11 (m, 3H), 7.24-7.27 (m, 1H); LC - MS (method C; ESI, *m/z*) t_R = 0.93 min – 204 $(M+H)^+$.

8-(4-(2-(4-(3-Isopropylphenyl)piperidin-1-yl)ethyl)-1*H*-pyrazol-1-yl)-3-((2-(trimethylsilyl)ethoxy)methyl)pyrido[3,4-*d*]pyrimidin-4(3*H*)-one (12e)

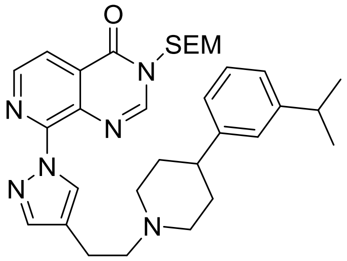

According to General Procedure 5, 2-(1-(4-oxo-3-((2-(trimethylsilyl)ethoxy)methyl)-3,4-dihydropyrido[3,4-*d*]pyrimidin-8-yl)-1*H*-pyrazol-4-yl)acetaldehyde (65 mg, 0.169 mmol), 4-(3-isopropylphenyl)piperidine (55 mg, 0.271 mmol) and sodium triacetoxyborohydride (35.7 mg, 0.169 mmol) were reacted together in dichloromethane (4 mL). Following workup procedure A, the title compound was obtained as a colorless oil (37 mg, 38%). ^{1}H NMR (500 MHz, $CDCl_3$) 0.02 (s, 9H), 0.94-1.04 (m, 2H), 1.26 (d, J = 6.9 Hz, 6H), 1.94 (br s, 2H), 2.29 (s, 2H), 2.57 (s, 1H), 2.81 (s, 2H), 2.91 (dq, J = 13.8, 6.9, 6.5 Hz, 3H), 3.30 (br s, 2H), 3.27 (s, 2H), 3.66-3.75 (m, 2H), 5.48 (s, 2H), 7.10 (ddt, J = 15.3, 7.6, 1.6 Hz, 3H), 7.26 (t, J = 7.6 Hz, 1H), 7.82 (s, 1H), 8.08 (d, J = 5.1 Hz, 1H), 8.32 (s, 1H), 8.60-8.67 (m, 2H); LC - MS (method C; ESI, *m/z*) t_R = 1.32 min – 573 $(M+H)^+$.

8-(4-(2-(4-(3-Isopropylphenyl)piperidin-1-yl)ethyl)-1*H*-pyrazol-1-yl)pyrido[3,4-d]pyrimidin-4(3*H*)-one (16e)

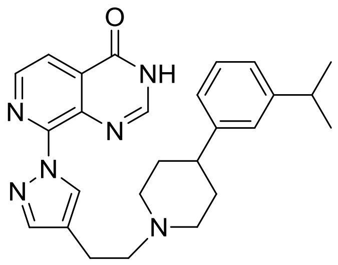

According to General Procedure 4, 8-(4-(2-(4-(3-isopropylphenyl)piperidin-1-yl)ethyl)-1*H*-pyrazol-1-yl)-3-((2-(trimethylsilyl)ethoxy)methyl)pyrido[3,4-*d*]pyrimidin-4(3*H*)-one (37.0 mg, 0.065 mmol) and hydrochloric acid (6 M, 1 mL) were reacted together in THF (1 mL) for 4 h. Purification was achieved by passing the crude product through an SCX cartridge eluting first with methanol and then 7N ammonia in methanol. Fractions containing the product were combined, concentrated *in vacuo*, and the residue triturated with Et_2O. The beige precipitate was obtained by filtration, and dried (10 mg, 35%). ^{1}H NMR (500 MHz, CD_3OD) 1.25 (d, *J* = 6.9 Hz, 6H), 185-1.90 (m, 4H), 2.37 (td, *J* = 11.6, 3.5 Hz, 2H), 2.61 (m, 1H), 2.82-2.93 (m, 4H), 3.27-3.33 (m, 2H), 7.05-7.08 (m, 2H), 7.12 (br s, 1H), 7.21 (t, *J* = 7.6 Hz, 1H), 7.81 (s, 1H), 8.05 (d, *J* = 5.1 Hz, 1H), 8.26 (s, 1H), 8.53 (d, *J* = 5.1 Hz, 1H), 8.77 (s, 1H); HRMS (Method D): t_R = 2.10 min - found 443.2558; calculated for $C_{26}H_{31}N_6O$ $(M+H)^+$ 443.2559.

1-Bromo-3-isobutoxybenzene

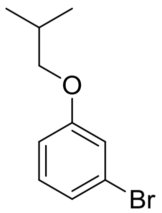

To a stirred solution of 3-bromophenol (346 mg, 2.0 mmol) in dry DMF (10 mL) was added potassium carbonate (552 mg, 4.0 mmol), followed by 1-bromo-2-methylpropane (274 mg, 2.0 mmol). The reaction mixture was heated to 80 °C, stirred at this temperature for 18 h and then allowed to cool to room temperature and diluted with ethyl acetate (50 mL). The

solution was washed with water (2 × 30 mL), dried, and concentrated in vacuo to give the title compound as a colourless oil (328 mg, 72%); ^{1}H NMR (500 MHz, $CDCl_3$) 1.04 (d, *J* = 6.7 Hz, 6H), 2.08-2.11 (m, 1H), 3.71 (d, *J* = 6.5 Hz, 2H), 7.04-7.13 (m, 4H).

tert-Butyl 4-(3-isobutoxyphenyl)-5,6-dihydropyridine-1(2*H*)-carboxylate (22f)

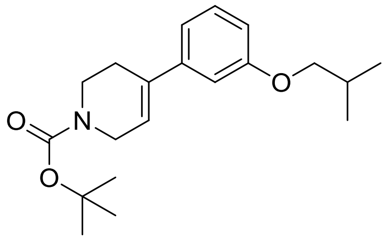

According to General Procedure 1, *tert*-butyl 4-(4,4,5,5-tetramethyl-1,3,2-dioxaborolan-2-yl)-5,6-dihydropyridine-1(2*H*)-carboxylate (170 mg, 0.55 mmol), 1-bromo-3-isobutoxybenzene (115 mg, 0.50 mmol) and $Pd(dppf)Cl_2.CH_2Cl_2$ (30 mg, 0.04 mmol) were reacted together in DME (3 mL) and aqueous sodium carbonate (1 M, 2 mL). Purification on a silica column eluting with 10% ethyl acetate in CH_2Cl_2 gave the product as colourless oil (143 mg, 78%). ^{1}H NMR (500 MHz, $CDCl_3$) 1.04 (d, *J* = 6.7 Hz, 6H), 1.51 (s, 9H), 2.10 (septet, *J* = 6.7 Hz, 1H), 2.54 (d, *J* = 4.9 Hz, 2H), 3.65 (t, *J* = 5.7 Hz, 2H), 3.74 (d, *J* = 6.5 Hz, 2H), 4.09 (br s, 2H), 6.05 (br s, 1H), 6.81 (ddd, *J* = 8.2, 2.5, 0.9 Hz, 1H), 6.90-6.99 (m, 2H), 7.25 (t, *J* = 7.9 Hz, 1H); LC - MS (method C; ESI, *m/z*) t_R = 1.68 min – 354 $(M+Na)^+$.

4-(3-Isobutoxyphenyl)piperidine (25f)

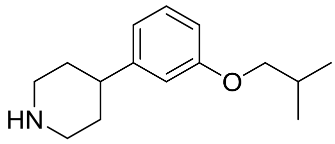

A solution of *tert*-butyl 4-(3-isobutoxyphenyl)-5,6-dihydropyridine-1(2*H*)-carboxylate (143 mg, 0.431 mmol) in ethanol (10 mL) was stirred in the presence of 10% palladium on carbon

under an atmosphere of hydrogen for 2 h. The reaction mixture was filtered through a pad of celite and the crude residue was passed through an isolute SCX-2 cartridge to give the reduced product (140 mg, 98%). This material was dissolved in a solution of HCl in dioxane (4 M; 5 mL) and stirred at room temperature for 2 h. The solution was then concentrated in vacuo, and the residue was passed through an isolute scx cartridge. Fractions containing the product were combined and concentrated in vacuo to afford the title compound as a colourless oil (96 mg, 95%). ^{1}H NMR (500 MHz, $CDCl_3$) 1.03 (d, *J* = 6.7 Hz, 6H), 1.65 (qd, *J* = 12.6, 4.0 Hz, 2H), 1.83-1.86 (m, 2H), 2.09 (septet, *J* = 6.7 Hz, 1H), 2.57 (tt, *J* = 12.1, 3.7 Hz, 1H), 2.74 (td, *J* = 12.3, 2.5 Hz, 2H), 3.16-3.22 (m, 2H), 3.71 (d, *J* = 6.6 Hz, 2H), 6.81 (ddd, *J* = 8.2, 2.6, 1.0 Hz, 1H), 6.79-6.82 (m, 2H), 7.21 (t, *J* = 7.9 Hz, 1H); LC - MS (method C; ESI, *m/z*) t_R = 0.97 min – 234 $(M+H)^+$.

8-(4-(2-(4-(3-Isobutoxyphenyl)piperidin-1-yl)ethyl)-1*H*-pyrazol-1-yl)-3-((2-(trimethylsilyl)ethoxy)methyl)pyrido[3,4-*d*]pyrimidin-4(3*H*)-one (12f)

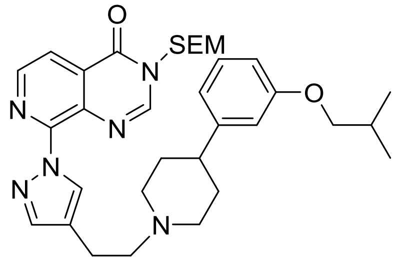

According to General Procedure 5, 2-(1-(4-oxo-3-((2-(trimethylsilyl)ethoxy)methyl)-3,4-dihydropyrido[3,4-*d*]pyrimidin-8-yl)-1*H*-pyrazol-4-yl)acetaldehyde (65 mg, 0.169 mmol), 4-(3-isobutoxyphenyl)piperidine (48 mg, 0.206 mmol) and sodium triacetoxyborohydride (35.7 mg, 0.169 mmol) were reacted together in dichloromethane (4 mL). Following workup procedure A, the title compound was obtained as a colourless oil (34 mg, 33%). ^{1}H NMR (500 MHz, $CDCl_3$) 0.02 (s, 9H), 0.97-1.01 (m, 2H), 1.03 (d, *J* = 6.7 Hz, 6H), 1.92 (br s, 3H), 2.09 (septet, *J* = 6.7 Hz, 1H), 2.25 (br s, 2H), 2.54 (m, 1H), 2.77 (br s, 2H), 2.90 (br s, 2H), 3.24 (br s, 2H), 3.69– 3.72 (m, 4H), 5.47 (s, 2H), 6.75 – 6.77 (m, 1H), 6.81 – 6.84 (m, 2H),

7.22 (t, *J* = 7.80 Hz, 1H), 7.81 (s, 1H), 8.08 (d, *J* = 5.1 Hz, 1H), 8.32 (s, 1H), 8.61 (s, 1H), 8.63 (d, *J* = 5.1 Hz, 1H); LC - MS (method C; ESI, *m/z*) t_R = 1.33 min – 603 $(M+H)^+$.

8-(4-(2-(4-(3-Isobutoxyphenyl)piperidin-1-yl)ethyl)-1*H*-pyrazol-1-yl)pyrido[3,4-*d*]pyrimidin-4(3*H*)-one (16f)

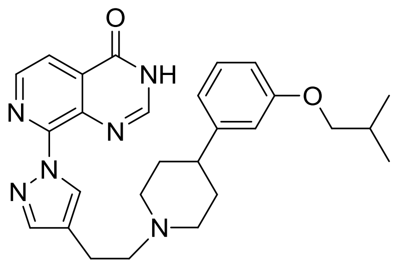

According to General Procedure 4, 8-(4-(2-(4-(3-isobutoxyphenyl)piperidin-1-yl)ethyl)-1*H*-pyrazol-1-yl)-3-((2-(trimethylsilyl)ethoxy)methyl)pyrido[3,4-*d*]pyrimidin-4(3*H*)-one (34.0 mg, 0.056 mmol) and hydrochloric acid (6 M, 1 mL) were reacted together in THF (1 mL) for 4 h. Purification was achieved by passing through an SCX-2 cartridge eluting first with methanol and then 7N ammonia in methanol. Fractions containing the product were combined, concentrated *in vacuo*, and the residue triturated with Et_2O. The beige precipitate was obtained by filtration , and dried (12 mg, 45%). ^{1}H NMR (500 MHz, CD_3OD) 1.04 (d, *J* = 6.7 Hz, 6H), 1.80-1.96 (m, 4H), 2.05-2.07 (m, 1H), 2.39 (td, *J* = 12.0, 2.9 Hz, 2H), 2.61 (tt, *J* = 11.9, 4.1 Hz, 1H), 2.77-2.95 (m, 4H), 3.29 (m, 2H), 3.73 (d, *J* = 6.5 Hz, 2H), 6.74-6.76 (m, 1H), 6.80-6.83 (m, 2H), 7.19 (t, *J* = 7.9 Hz, 1H), 7.82 (s, 1H), 8.05 (d, *J* = 5.1 Hz, 1H), 8.26 (s, 1H), 8.54 (d, *J* = 5.1 Hz, 1H), 8.77 (s, 1H); HRMS (Method D): t_R = 2.22 min - found 473.2675; calculated for $C_{27}H_{33}N_6O_2$ $(M+H)^+$ 473.2665.

***tert*-Butyl 4-(3-(pyridin-2-yl)phenyl)-3,6-dihydropyridine-1(2*H*)-carboxylate (22g)**

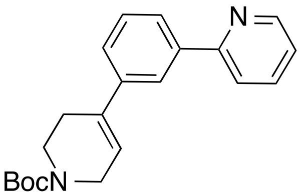

According to General Procedure 1, *tert*-butyl 4-(4,4,5,5-tetramethyl-1,3,2-dioxaborolan-2-yl)-5,6-dihydropyridine-1(2*H*)-carboxylate (300 mg, 0.970 mmol), 2-(3-bromophenyl)pyridine (227 mg, 0.970 mmol) and Pd(dppf)Cl_2.CH_2Cl_2 (79 mg, 0.097 mmol) were reacted together in DME (3 mL) and aqueous sodium carbonate (1 M, 2 mL). Purification on a KP-Sil snap cartridge (5% [0.2 M NH_3 in MeOH] in CH_2Cl_2) gave a pale yellow oil, which was shown to be a mixture of the Boc-protected material and the carbamic acid. This material was taken forward to the next step without further purification.

2-(3-(Piperidin-4-yl)phenyl)pyridine(25g)

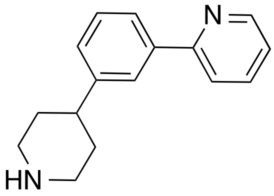

According to General Procedure 2, $Pd(OH)_2$ on carbon (15 mg, 0.96 mmol) and *tert*-butyl 4-(3-(pyridin-2-yl)phenyl)-5,6-dihydropyridine-1(2*H*)-carboxylate (80 mg, 0.238 mmol) were reacted together in EtOH (3 mL) and hydrochloric acid (1 M, 1 mL); at this stage the Boc group was fully removed. The crude material was purified by passing through an SCX-2 cartridge eluting with 1 M NH_3 in MeOH/CH_2Cl_2. The ammoniacal solution was concentrated *in vacuo* to yield the product as a pale yellow oil (31.9 mg, 56% over two steps); ^{1}H NMR (500 MHz, $CDCl_3$) 1.73 (qd, *J* = 12.5, 3.8 Hz, 2H), 1.90 (br d, *J* = 12.5 Hz, 2H), 2.63 (br s, 1H), 2.69-2.81 (m, 3H), 3.21 (br d, *J* = 11.9 Hz, 2H), 7.22 (ddd, *J* = 6.7, 4.8, 1.7 Hz, 1H), 7.29 (br d, *J* = 7.9 Hz, 1H), 7.42 (t, *J* = 7.9 Hz, 1H), 7.70-7.77 (m, 2H), 7.78-

7.81 (m, 1H), 7.89 (br t, *J* = 1.7 Hz, 1H), 8.68-8.71 (m, 1H); LC - MS (method C; ESI, *m/z*) t_R = 0.55 min – 239 [(M+H)$^+$]; HRMS (method D): found 239.1541; calculated for $C_{16}H_{19}N_2$ (M+H)$^+$ 239.1548.

8-(4-(2-(4-(3-(Pyridin-2-yl)phenyl)piperidin-1-yl)ethyl)-1*H*-pyrazol-1-yl)-3-((2-(trimethylsilyl)ethoxy)methyl)pyrido[3,4-*d*]pyrimidin-4(3*H*)-one (12g)

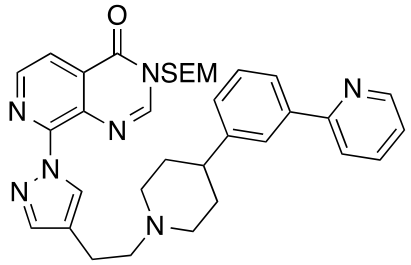

According to General Procedure 3, triethylamine (0.03 mL, 0.237 mmol), 2-(1-(4-oxo-3-((2-(trimethylsilyl)ethoxy)methyl)-3,4-dihydropyrido[3,4-*d*]pyrimidin-8-yl)-1*H*-pyrazol-4-yl)-ethyl methanesulfonate (55.1 mg, 0.118 mmol) and 2-(3-(piperidin-4-yl)phenyl)pyridine (29.9 mg, 0.125 mmol) were reacted together in anhydrous DMF (1 mL). Purification on a KP-Sil snap cartridge (9% [0.2 M NH_3 in MeOH] in CH_2Cl_2) gave the product as a pale yellow oil (34.7 mg, 48%); ^{1}H NMR (500 MHz, $CDCl_3$) 0.01 (s, 9H), 0.96-1.01 (m, 2H), 1.96 (br s, 4H), 2.22 (br s, 2H), 2.67 (quintet, *J* = 7.7 Hz, 1H), 2.71-2.79 (m, 2H), 2.84-2.92 (m, 2H), 3.21 (br d, *J* = 10.1 Hz, 2H), 3.67-3.72 (m, 2H), 5.46 (s, 2H), 7.24 (ddd, *J* = 6.8, 4.9, 1.8 Hz, 1H), 7.32 (br d, *J* = 7.5 Hz, 1H), 7.43 (t, *J* = 7.5 Hz, 1H), 7.72-7.78 (m, 2H), 7.79-7.83 (m, 2H), 7.93 (br t, *J* = 1.8 Hz, 1H), 8.07 (d, *J* = 5.0 Hz, 1H), 8.32 (s, 1H), 8.61 (s, 1H), 8.64 (d, *J* = 5.0 Hz, 1H), 8.68-8.81 (m, 1H); LC - MS (method C; ESI, *m/z*) t_R = 1.19 min – 608 [(M+H)$^+$]; HRMS (method D): found 608.3130; calculated for $C_{34}H_{42}N_7O_2Si$ (M+H)$^+$ 608.3169.

8-(4-(2-(4-(3-(Pyridin-2-yl)phenyl)piperidin-1-yl)ethyl)-1*H*-pyrazol-1-yl)pyrido[3,4-*d*]pyrimidin-4(3*H*)-one (16g)

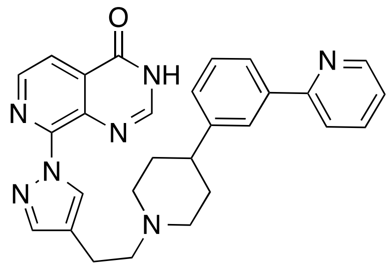

According to General Procedure 4, 8-(4-(2-(4-(3-(pyridin-2-yl)phenyl)piperidin-1-yl)ethyl)-1*H*-pyrazol-1-yl)-3-((2-(trimethylsilyl)ethoxy)methyl)pyrido[3,4-*d*]pyrimidin-4(3*H*)-one (22.9 mg, 0.038 mmol) and hydrochloric acid (6 M, 0.50 mL) were reacted together in THF (0.5 mL). Purification on a KP-NH snap cartridge (40% EtOH in CH_2Cl_2) gave the title compound as a white solid (7.1 mg, 40%); ^{1}H NMR (500 MHz, DMSO-d_6) 1.75 (qd, J = 12.1, 3.6 Hz, 2H), 1.80-1.86 (m, 2H), 2.12 (t, J = 11.3 Hz, 2H), 2.57-2.65 (m, 3H), 2.73 (t, J = 7.4 Hz, 2H), 3.10 (br d, J = 11.3 Hz, 2H), 7.32-7.36 (m, 2H), 7.42 (t, J = 7.6 Hz, 1H), 7.73 (s, 1H), 7.85-7.91 (m, 2H), 7.95-7.99 (m, 3H), 8.29 (s, 1H), 8.45 (s, 1H), 8.53-8.56 (m, 1H), 8.65-8.67 (m, 1H), 12.79 (br s, 1H); LC - MS (method C; ESI, *m/z*) t_R = 0.75 min – 478 [(M+H)$^+$]; HRMS (method D): found 478.2370; calculated for $C_{28}H_{28}N_7O$ (M+H)$^+$ 478.2355.

***tert*-Butyl 4-(3-(pyrimidin-5-yl)phenyl)piperidine-1-carboxylate**

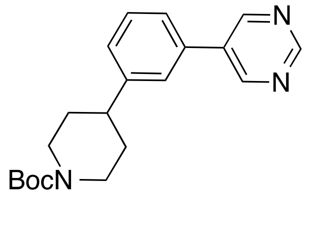

According to General Procedure 1, 5-(4,4,5,5-tetramethyl-1,3,2-dioxaborolan-2-yl)pyrimidine (180 mg, 0.874 mmol), *tert*-butyl 4-(3-bromophenyl)piperidine-1-carboxylate (297 mg, 0.874 mmol) and Pd(dppf)Cl_2.CH_2Cl_2 (71 mg, 0.087 mmol) were reacted together

in DME (3 mL) and aqueous sodium carbonate (1 M, 2 mL). Purification on a KP-Sil snap cartridge (15% EtOAc in cyclohexane) gave the product as a pale yellow oil (296 mg, quant.); ^{1}H NMR (500 MHz, $CDCl_3$) 1.37 (s, 9H), 1.56 (qd, *J* = 12.5, 4.0 Hz, 2H), 1.75 (br d, *J* = 12.5 Hz, 2H), 2.63 (tt, *J* = 12.2, 3.4 Hz, 1H), 2.71 (br s, 2H), 4.17 (br s, 2H), 7.19 (br d, *J* = 7.4 Hz, 1H), 7.27-7.36 (m, 3H), 8.81 (s, 2H), 9.05 (s, 1H); LC - MS (method C; ESI, *m/z*) t_R = 1.41 min – 284 [(M–^{t}Bu+2H)$^+$]; HRMS (method D): found 284.1399; calculated for $C_{16}H_{18}N_3O_2$ (M–^{t}Bu+2H)$^+$ 284.1399.

5-(3-(Piperidin-4-yl)phenyl)pyrimidine (25i)

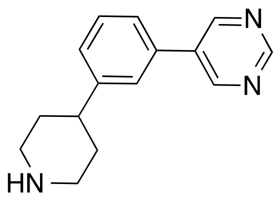

tert-Butyl 4-(3-(pyrimidin-5-yl)phenyl)piperidine-1-carboxylate was dissolved in a mixture of THF (3 mL) and hydrochloric acid (1 M, 3 mL). The reaction mixture was stirred at 50 °C for 2 h and monitored by LCMS. On completion of the reaction, the reaction mixture was concentrated *in vacuo* and the residue redissolved in MeOH/CH_2Cl_2. The crude material was passed through an SCX-2 cartridge eluting with 1 M NH_3 in MeOH/CH_2Cl_2. The ammoniacal solution was concentrated *in vacuo* to yield the product as an off-white solid (114.1 mg, 76%); ^{1}H NMR (500 MHz, $CDCl_3$) 1.63 (qd, *J* = 12.6, 3.7 Hz, 2H), 1.81 (br d, *J* = 12.6 Hz, 2H), 1.87 (br s, 1H), 2.64 (tt, *J* = 12.2, 3.5 Hz, 1H), 2.70 (td, *J* = 12.2, 2.5 Hz, 2H), 3.14 (br d, *J* = 12.2 Hz, 2H), 7.25-7.28 (m, 1H), 7.32-7.40 (m, 3H), 8.87 (s, 2H), 9.12 (s, 1H); LC - MS (method C; ESI, *m/z*) t_R = 0.63 min – 240 [(M+H)$^+$]; HRMS (method D): found 240.1507; calculated for $C_{15}H_{18}N_3$ (M+H)$^+$ 240.1500.

8-(4-(2-(4-(3-(Pyrimidin-5-yl)phenyl)piperidin-1-yl)ethyl)-1*H*-pyrazol-1-yl)-3-((2-(trimethylsilyl)ethoxy)methyl)pyrido[3,4-*d*]pyrimidin-4(3*H*)-one (12i)

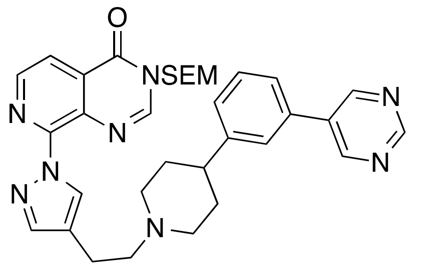

According to General Procedure 3, triethylamine (0.04 mL, 0.309 mmol), 2-(1-(4-oxo-3-((2-(trimethylsilyl)ethoxy)methyl)-3,4-dihydropyrido[3,4-*d*]pyrimidin-8-yl)-1*H*-pyrazol-4-yl)-ethyl methanesulfonate (72 mg, 0.155 mmol) and 5-(3-(piperidin-4-yl)phenyl)pyrimidine (120 mg, 0.501 mmol) were reacted together in anhydrous DMF (1 mL). Purification on a KP-Sil snap cartridge (6% [0.2 M NH_3 in MeOH] in CH_2Cl_2) gave the product as a pale yellow oil (61.5 mg, 65%); ^{1}H NMR (500 MHz, $CDCl_3$) 0.00 (s, 9H), 0.94-1.00 (m, 2H), 1.84-1.97 (m, 4H), 2.20 (td, J = 11.0, 3.0 Hz, 2H), 2.64 (tt, J = 11.1, 4.7 Hz, 1H), 2.68-2.74 (m, 2H), 2.81-2.87 (m, 2H), 3.18 (br d, J = 11.2 Hz, 2H), 3.66-3.71 (m, 2H), 5.46 (s, 2H), 7.36 (br d, J = 7.5 Hz, 1H), 7.40-7.48 (m, 3H), 7.80 (br s, 1H), 7.06 (d, J = 5.1 Hz, 1H), 8.31 (s, 1H), 8.56 (s, 1H), 8.62 (d, J = 5.1 Hz, 1H), 8.94 (s, 2H), 9.20 (s, 1H); LC - MS (method C; ESI, *m/z*) t_R = 1.14 min – 609 [$(M+H)^+$]; HRMS (method D): found 609.3124; calculated for $C_{33}H_{41}N_8O_2Si$ $(M+H)^+$ 609.3121.

8-(4-(2-(4-(3-(Pyrimidin-5-yl)phenyl)piperidin-1-yl)ethyl)-1*H*-pyrazol-1-yl)pyrido[3,4-*d*]pyrimidin-4(3*H*)-one (16i)

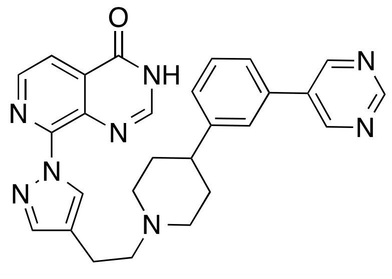

According to General Procedure 4, 8-(4-(2-(4-(3-(pyrimidin-5-yl)phenyl)piperidin-1-yl)ethyl)-1*H*-pyrazol-1-yl)-3-((2-(trimethylsilyl)ethoxy)methyl)pyrido[3,4-*d*]pyrimidin-4(3*H*)-one (59.8 mg, 0.098 mmol) and hydrochloric acid (6 M, 1 mL) were reacted together in THF (1 mL). Purification on a KP-NH snap cartridge (40% EtOH in CH_2Cl_2) gave the title compound as a white solid (38.5 mg, 82%); ^{1}H NMR (500 MHz, DMSO-d_6) 1.73-1.88 (m, 4H), 2.08-2.19 (m, 2H), 2.58-2.68 (m, 3H), 2.74 (t, *J* = 7.2 Hz, 2H), 3.12 (br d, *J* = 10.6 Hz, 2H), 7.38 (br d, *J* = 8.0 Hz, 1H), 7.47 (t, *J* = 7.6 Hz, 1H), 7.62-7.65 (m, 1H), 7.70 (br s, 1H), 7.74 (br s, 1H), 7.99 (d, *J* = 5.1 Hz, 1H), 8.29 (s, 1H), 8.43 (s, 1H), 8.56 (d, *J* = 5.1 Hz, 1H), 9.15 (s, 2H), 9.18 (s, 1H), 12.76 (br s, 1H); LC - MS (method C; ESI, *m/z*) t_R = 0.83 min – 479 [$(M+H)^+$]; HRMS (method D): found 479.2315; calculated for $C_{27}H_{27}N_8O$ $(M+H)^+$ 479.2308.

1-(3-Iodobenzyl)pyrrolidine

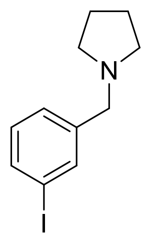

To a stirred solution of 3-iodobenzaldehyde (200 mg, 0.862 mmol) in dry CH_2Cl_2 (10 mL) was added pyrrolidine (61.3 mg, 0.862 mmol). After 10 mins, sodium triacetoxyborohydride

(190 mg, 0.90 mmol) was added and the reaction mixture was stirred at room temperature for 1 h, then it was washed with saturated sodium bicarbonate solution, dried, and concentrated in vacuo. The crude was filtered through an isolute SCX-2 cartridge to give the title compound (205 mg, 83%); ^{1}H NMR (500 MHz, $CDCl_3$) 1.77-1.82 (m, 4H), 2.48-2.52 (m, 4H), 3.56 (s, 2H), 7.05 (t, *J* = 7.7 Hz, 1H), 7.29-7.31 (m, 1H), 7.58 (dt, *J* = 7.9, 1.4 Hz, 1H), 7.71 (t, *J* = 1.7 Hz, 1H); LC - MS (method C; ESI, *m/z*) t_R = 0.64 min – 288 $(M+H)^+$.

Benzyl 4-(3-(pyrrolidin-1-ylmethyl)phenyl)-5,6-dihydropyridine-1(2*H*)-carboxylate (22j)

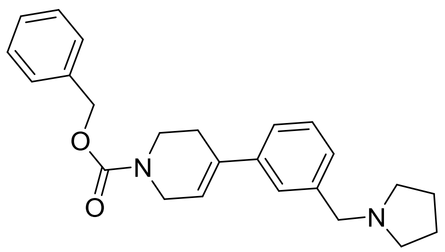

According to general procedure 1, benzyl 4-(4,4,5,5-tetramethyl-1,3,2-dioxaborolan-2-yl)-5,6-dihydropyridine-1(2*H*)-carboxylate (245 mg, 0.714 mmol), 1-(3-iodobenzyl)pyrrolidine (205 mg, 0.714 mmol) and $Pd(dppf)Cl_2.CH_2Cl_2$ (30 mg, 0.04 mmol) were reacted together in DME (3 mL) and aqueous sodium carbonate (1 M, 2 mL). Filtration on an isolute SCX-2 cartridge followed by purification on a silica column eluting with 3% [7 M NH_3 in MeOH] in CH_2Cl_2 gave the product as a brown oil (100 mg, 37%). ^{1}H NMR (500 MHz, $CDCl_3$) 1.85 (br s, 4H), 2.59 (br s, 6H), 3.70-3.74 (m, 4H), 4.17 (s, 2H), 5.20 (s, 2H), 6.05 (br d, *J* = 12.6 Hz, 1H), 7.26-7.41 (m, 9H); LC - MS (method C; ESI, *m/z*) t_R = 1.05 min – 377 $(M+H)^+$.

4-(3-(Pyrrolidin-1-ylmethyl)phenyl)piperidine (25j)

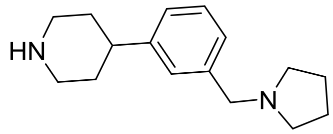

A solution benzyl 4-(3-(pyrrolidin-1-ylmethyl)phenyl)-5,6-dihydropyridine-1(2*H*)-carboxylate (100 mg, 0.266 mmol) in ethanol (10 ml) was stirred in the presence of 10% palladium on charcoal under an atmosphere of hydrogen for 1 h. The solvent was then removed in vacuo, and the residue was redissolved in ethanol (10 mL) and 2 equivalents of acetic acid were added. The reaction mixture was stirred in the presence of 10% palladium on charcoal under an atmosphere of hydrogen for 7 h, then filtered through a pad of celite and the crude residue put through an isolute SCX-2 cartridge followed by silica column chromatography eluting with 10% [7 M NH_3 in MeOH] in CH_2Cl_2 to give the title compound (60 mg, 92%). ^{1}H NMR (500 MHz, $CDCl_3$) 1.65 (dtd, *J* = 13.2, 12.0, 4.0 Hz, 2H), 1.75-1.85 (m, 6H), 2.49 (tdd, J = 7.2, 3.5, 2.2 Hz, 5H), 2.61 (tt, *J* = 12.1, 3.7 Hz, 1H), 2.73 (td, *J* = 12.2, 2.5 Hz, 2H), 3.18 (dt, *J* = 12.4, 3 Hz, 2H), 3.59 (s, 2H), 7.09-7.25 (m, 4H); LC - MS (method C; ESI, *m/z*) t_R = 0.23 min – 245 (M+H).$^+$

8-(4-(2-(4-(3-(Pyrrolidin-1-ylmethyl)phenyl)piperidin-1-yl)ethyl)-1*H*-pyrazol-1-yl)-3-((2-(trimethylsilyl)ethoxy)methyl)pyrido[3,4-*d*]pyrimidin-4(3*H*)-one (12j)

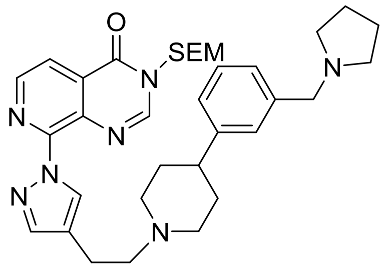

According to General Procedure 5, 2-(1-(4-oxo-3-((2-(trimethylsilyl)ethoxy)methyl)-3,4-dihydropyrido[3,4-*d*]pyrimidin-8-yl)-1*H*-pyrazol-4-yl)acetaldehyde (65 mg, 0.169 mmol), 4-(3-(pyrrolidin-1-ylmethyl)phenyl)piperidine (41.2 mg, 0.169 mmol) and sodium triacetoxyborohydride (35.7 mg, 0.169 mmol) were reacted together in CH_2Cl_2 (4 mL). Following workup procedure A, the title compound was obtained as a colorless oil (45 mg, 44%). ^{1}H NMR (500 MHz, $CDCl_3$) 0.02 (s, 9H), 0.95-0.99 (m, 2H), 1.77-1.80 (m, 4H), 1.82-

1.87 (m, 4H), 2.14-2.16 (m, 2H), 2.51-2.54 (m, 5H), 2.67-2.70 (m, 2H), 2.81-2.84 (m, 2H), 3.14 (dt, *J* = 12.1 3.1 Hz, 2H), 3.62 (br s, 2H), 3.67-3.69 (m, 2H), 5.45 (s, 2H), 7.12-7.17 (m, 2H), 7.22-7.26 (m, 2H), 7.80 (s, 1H), 8.05 (d, *J* = 5.1 Hz, 1H), 8.30 (s, 1H), 8.57 (s, 1H), 8.61 (d, *J* = 5.1 Hz, 1H); LC - MS (method C; ESI, *m/z*) t_R = 0.93 min – 614 $(M+H)^+$.

8-(4-(2-(4-(3-(Pyrrolidin-1-ylmethyl)phenyl)piperidin-1-yl)ethyl)-1*H*-pyrazol-1-yl)pyrido[3,4-*d*]pyrimidin-4(3*H*)-one (16j)

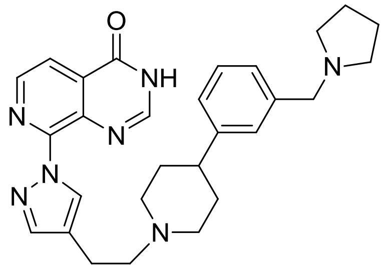

According to General Procedure 4, 8-(4-(2-(4-(3-(pyrrolidin-1-ylmethyl)phenyl)piperidin-1-yl)ethyl)-1*H*-pyrazol-1-yl)-3-((2-(trimethylsilyl)ethoxy)methyl)pyrido[3,4-*d*]pyrimidin-4(3*H*)-one (45 mg, 0.073 mmol) and hydrochloric acid (6 M, 1 mL) were reacted together in THF (1 mL) for 4 h. Purification was achieved by passing the crude product through an SCX cartridge eluting first with methanol and then 7 N ammonia in methanol. Fractions containing the product were combined, concentrated *in vacuo*, and the residue triturated with Et_2O. A beige precipitate was obtained by filtration and dried (26 mg, 73%). ^{1}H NMR (500 MHz, CD_3OD) 1.93-2.08 (m, 6H), 2.68-2.72 (m, 2H), 2.79-2.83 (m, 1H), 2.98-3.05 (m, 2H), 2.99-3.05 (m, 2H), 3.06-3.11 (m, 2H), 3.23 (br s, 4H), 3.47 (br d, 2H), 4.24 (s, 2H), 7.35-7.40 (m, 2H), 7.42-7.45 (m, 2H), 7.85 (s, 1H), 8.08 (d, *J* = 5.2 Hz, 1H), 8.26 (s, 1H), 8.58 (d, *J* = 5.1 Hz, 1H), 8.80 (s, 1H); HRMS (method D) t_R = 2.17 min - found 484.2828; calculated for $C_{28}H_{34}N_7O$ $(M+H)^+$ 484.2819.

8-(4-(2-(4-(3-(Morpholinomethyl)phenyl)piperidin-1-yl)ethyl)-1*H*-pyrazol-1-yl)-3-((2-(trimethylsilyl)ethoxy)methyl)pyrido[3,4-*d*]pyrimidin-4(3*H*)-one (12k)

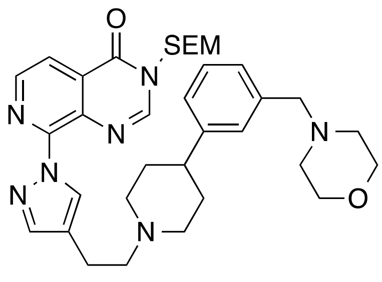

According to General Procedure 3, 2-(1-(4-oxo-3-((2-(trimethylsilyl)ethoxy)methyl)-3,4-dihydropyrido[3,4-*d*]pyrimidin-8-yl)-1*H*-pyrazol-4-yl)ethyl methanesulfonate (50 mg, 0.107 mmol), 4-(3-(piperidin-4-yl)benzyl)morpholine (42 mg, 0.161 mmol) and triethylamine (0.022 mL, 0.161 mmol) in DMF was reacted at 50 °C overnight. After workup the oil obtained was purified by flash column chromatography on a SNAP KP-Sil column eluting with 0-40% EtOH/ CH_2Cl_2 to afford the title compound as a pale yellow oil (53 mg, 78%). ^{1}H NMR (500 MHz, CD_3OD): 0.01 (s, 9H), 0.95-1.00 (m, 2H), 1.79-1.91 (m, 4H), 2.28 (dt, J = 11.3, 2.8 Hz, 2H), 2.45 (br s, 4H), 2.56-2.64 (m, 1H), 2.73-2.78 (m, 2H), 2.85-2.90 (m, 2H), 3.19-3.24 (m, 2H), 3.51 (s, 2H), 3.68 (t, J = 4.8 Hz, 4H), 3.72-3.77 (m, 2H), 5.49 (s, 2H), 7.16-7.19 (m, 2H), 7.23-7.28 (m, 2H), 7.81 (br s, 1H), 8.10 (d, J = 5.1 Hz, 1H), 8.46 (s, 1H), 8.59 (br s, 1H), 8.78 (br s, 1H); LC-MS (method A; ESI, *m/z*) t_R = 1.52 min - 466.1572 $(M+H)^+$

8-(4-(2-(4-(3-(Morpholinomethyl)phenyl)piperidin-1-yl)ethyl)-1*H*-pyrazol-1-yl)pyrido[3,4-*d*]pyrimidin-4(3*H*)-one (16k)

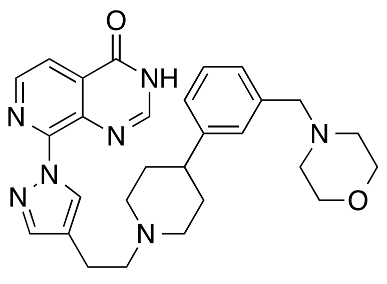

According to General Procedure 6, 8-(4-(2-(4-(3-(morpholinomethyl)phenyl)piperidin-1-yl)ethyl)-1*H*-pyrazol-1-yl)-3-((2-(trimethylsilyl)ethoxy)methyl)pyrido[3,4-*d*]pyrimidin-4(3*H*)-one (36 mg, 0.057 mmol) was reacted with HCl in 1,4-dioxane (4M, 0.143 mL, 0.572 mmol) in 1,4-dioxane/water at 50 °C for 12 h. After workup and flash column chromatography the product was obtained as a pale yellow solid (3 mg, 11%). ^{1}H NMR (500 MHz, DMSO-d_6) 1.66 (dq, *J* = 12.6, 3.5, 2H), 1.72-1.79 (m, 2H), 2.07 (td, *J* = 12.0, 2.2 Hz, 2H), 2.31-2.36 (m, 4H), 2.55-2.63 (m, 3H), 2.68-2.74 (m, 2H), 3.05-3.10 (m, 2H), 3.43 (s, 2H), 3.54-3.58 (m, 4H), 7.13 (br t, *J* = 7.6 Hz, 2H), 7.16 (br s, 1H), 7.23 (t, *J* = 7.6 Hz, 1H), 7.72 (s, 1H), 7.96 (d, *J* = 5.1 Hz, 1H), 8.28 (s, 1H), 8.44 (s, 1H), 8.52 (d, *J* = 5.1 Hz, 1H); LC- HRMS (method B; ESI, *m/z*) t_R = 0.57 min - HRMS: found 500.2759; calculated for $C_{28}H_{34}N_7O_2$ $(M+H)^+$ 500.2768.

2-(3-Bromophenyl)acetaldehyde

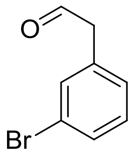

To a solution of 2-(3-bromophenyl)ethanol (400 mg, 1.99 mmol) in dry CH_2Cl_2 (10 mL) was added Dess-Martin periodinane (848mg, 2.00 mmol) and the reaction mixture was stirred at room temperature for 1 h. It was then washed with saturated sodium hydrogen carbonate

solution, dried, and concentrated *in vacuo* to give the product (400 mg, 100%) which was used in the next step without further purification. ^{1}H NMR (500 MHz, $CDCl_3$) 3.72 (s, 2H), 7.15 – 7.32 (m, 4H), 9.71 (s, 1H); LC - MS (method C; ESI, *m/z*) t_R = 0.86 min – 231 $(M+MeOH)^+$.

4-(3-Bromophenethyl)morpholine

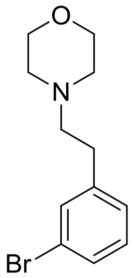

To a stirred solution of 2-(3-bromophenyl)acetaldehyde (400 mg, 2.01 mmol) in dry CH_2Cl_2 (10 mL) was added morpholine (175 mg, 2.01 mmol). After 10 min, sodium triacetoxyborohydride (424 mg, 2.0 mmol) was added and the reaction mixture was stirred at room temperature for 1 h, it was then washed with a saturated bicarbonate solution, dried, and concentrated in vacuo. The crude product was filtered through an isolute SCX-2 cartridge then purified on a silica column eluting with 3% [7 M NH_3 in MeOH] in CH_2Cl_2 to afford the title compound (190 mg, 35%). This material was used in the next step without further purification. LC - MS (method C; ESI, *m/z*) t_R = 0.62 min – 270, 272 [$(M+H)^+$, Br isotopic pattern)].

Benzyl-4-(3-(2-morpholinoethyl)phenyl)-5,6-dihydropyridine-1(2*H*)-carboxylate (22l)

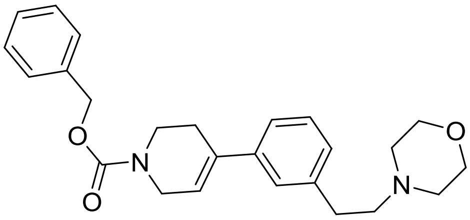

According to General Procedure 1, benzyl 4-(4,4,5,5-tetramethyl-1,3,2-dioxaborolan-2-yl)-5,6-dihydropyridine-1(2*H*)-carboxylate (241 mg, 0.703 mmol), 4-(3-bromophenethyl)morpholine (190 mg, 0.703 mmol) and Pd(dppf)Cl_2.CH_2Cl_2 (30 mg, 0.04 mmol) were reacted together in DME (3 mL) and aqueous sodium carbonate (1 M, 2 mL). Filtration on an isolute SCX-2 cartridge followed by purification on a silica column eluting with 3% [7 M NH_3 in MeOH] in CH_2Cl_2 gave the product as a brown oil (215 mg, 75%). ^{1}H NMR (500 MHz, $CDCl_3$) 2.54-2.63 (m, 8H), 2.81-2.84 (m, 2H), 3.73-3.77 (m, 6H), 4.17 (d, J = 2.4 Hz, 2H), 5.20 (s, 2H), 6.05 (br d, *J* = 12.6 Hz, 1H), 7.12-7.42 (m, 9H); LC - MS (method C; ESI, *m/z*) t_R = 1.08 min – 407 $(M+H)^+$.

4-(3-(Piperidin-4-yl)phenethyl)morpholine (25l)

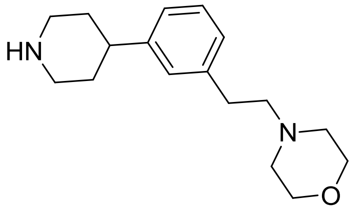

A solution of benzyl 4-(3-(2-morpholinoethyl)phenyl)-5,6-dihydropyridine-1(2*H*)-carboxylate (215 mg, 0.53 mmol) in ethanol (10 mL) was stirred in the presence of 10% palladium on carbon under an atmosphere of hydrogen for 1 h. The solvent was then removed in vacuo, the residue was redissolved in ethanol (10 mL) and 2 equivalents of acetic acid were added. The reaction mixture was stirred in the presence of 10% palladium on charcoal under an atmosphere of hydrogen for 7 h, it was then filtered through a pad of celite, and the crude residue was passed through an isolute SCX-2 cartridge to give the title compound as a brown oil (137 mg, 94%). ^{1}H NMR (500 MHz, $CDCl_3$) 1.65 (qd, *J* = 12.5, 3.7 Hz, 2H), 1.78-1.86 (m, 2H), 2.44-2.67 (m, 8H), 2.70-2.81 (m, 3H), 3.19 (d, *J* = 11.9 Hz, 2H), 3.77 (t, *J* = 4.7 Hz, 4H), 7.04-7.26 (m, 4H); LC - MS (method C; ESI, *m/z*) t_R = 0.25 min – 275 $(M+H)^+$.

8-(4-(2-(4-(3-(2-Morpholinoethyl)phenyl)piperidin-1-yl)ethyl)-1*H*-pyrazol-1-yl)-3-((2-(trimethylsilyl)ethoxy)methyl)pyrido[3,4-*d*]pyrimidin-4(3*H*)-one (12l)

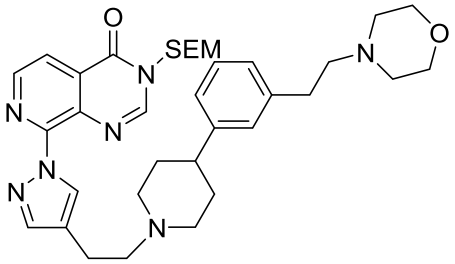

According to General Procedure 5, 2-(1-(4-oxo-3-((2-(trimethylsilyl)ethoxy)methyl)-3,4-dihydropyrido[3,4-*d*]pyrimidin-8-yl)-1*H*-pyrazol-4-yl)acetaldehyde (65 mg, 0.169 mmol), 4-(3-(piperidin-4-yl)phenethyl)morpholine (46.3 mg, 0.169 mmol) and sodium triacetoxyborohydride (35.7 mg, 0.169 mmol) were reacted together in dichloromethane (4 mL) (22 mg, 20%). Following workup procedure A, the title compound was obtained as a colorless oil. ^{1}H NMR (500 MHz, $CDCl_3$) 0.02 (s, 9H), 0.97-0.99 (m, 2H), 1.25-1.28 (m, 2H), 1.85-1.87 (m, 4H), 2.14-2.18 (m, 1H), 2.51-2.55 (m, 4H), 2.58-2.62 (m, 2H), 2.70-2.72 (m, 2H), 2.76-2.89 (m, 4H), 3.17 (br d, J = 12.1 Hz, 2H), 3.68-3.72 (m, 2H), 3.74-3.77 (m, 4H), 5.47 (s, 2H), 7.06-7.11 (m, 3H), 7.23-7.26 (m, 1H), 7.82 (s, 1H), 8.07 (d, J = 5.1 Hz, 1H), 8.32 (s, 1H), 8.58 (s, 1H), 8.63 (d, J = 5.1 Hz, 1H); LC - MS (method C; ESI, *m/z*) t_R = 0.94 min – 644 $(M+H)^+$.

8-(4-(2-(4-(3-(2-Morpholinoethyl)phenyl)piperidin-1-yl)ethyl)-1*H*-pyrazol-1-yl)pyrido[3,4-*d*]pyrimidin-4(3*H*)-one (16l)

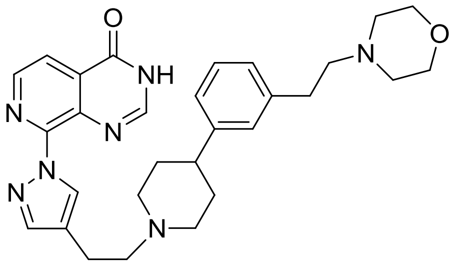

According to General Procedure 4, 8-(4-(2-(4-(3-(2-morpholinoethyl)phenyl)piperidin-1-yl)ethyl)-1*H*-pyrazol-1-yl)pyrido[3,4-*d*]pyrimidin-4(3*H*)-one (22 mg, 0.034 mmol) and hydrochloric acid (6 M, 1 mL) were reacted together in THF (1 mL) for 4 h. Purification was achieved by passing the crude product through an SCX-2 cartridge eluting first with methanol and then 7N ammonia in methanol. Fractions containing the product were combined, concentrated *in vacuo*, and the residue triturated with Et_2O. A beige precipitate was obtained by filtration, and dried (5 mg, 28%). ^{1}H NMR (500 MHz, DMSO-d_6) 1.68 (qd, J = 12.3, 3.7 Hz, 2H), 1.72-1.78 (m, 2H), 2.14 (br s, 2H), 2.42 (br s, 4H), 2.48-2.52 (m, 3H, obscured by DMSO), 2.59-2.67 (m, 2H), 2.67-2.76 (m, 4H), 3.11 (d, J = 10.9 Hz, 2H), 3.57 (t, J = 4.6 Hz, 4H), 7.02-7.08 (m, 2H), 7.10 (br s, 1H), 7.19 (t, J = 7.5 Hz, 1H), 7.74 (s, 1H), 7.99 (d, J = 5.1 Hz, 1H), 8.29 (s, 1H), 8.43 (s, 1H), 8.56 (d, J = 5.1 Hz, 1H); HRMS (Method D): t_R 0.96 min - found 514.2941; calculated for $C_{29}H_{36}N_7O_2$ $(M+H)^+$ 514.2930.

2-(3-Bromophenyl)-*N,N*-dimethylethanamine

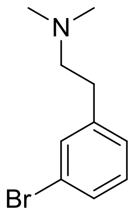

To a stirred solution of 2-(3-bromophenyl)acetaldehyde (200 mg, 1.0 mmol) in dry CH_2Cl_2 (10 mL) was added a solution of dimethylamine in THF (2M, 0.5 mL, 1.0 mmol). After 10 min, sodium triacetoxyborohydride (212 mg, 1.0 mmol) was added, and stirring was continued at room temperature for 1 h. The reaction mixture was then washed with a saturated sodium bicarbonate solution, dried, and concentrated in vacuo. The resulting residue was purified by passing through an isolute SCX-2 cartridge to give the product as a colourless oil (170 mg, 74%). This material was used in the next step without further

purification. LC - MS (method C; ESI, *m/z*) t_R = 0.61 min – 228, 230 [$(M+H)^+$, Br isotopic pattern].

***tert*-Butyl-4-(3-(2-(dimethylamino)ethyl)phenyl)-5,6-dihydropyridine-1(2*H*)-carboxylate (22m)**

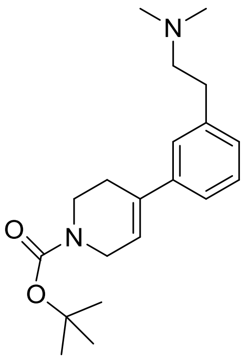

According to General Procedure 1, *tert*-butyl 4-(4,4,5,5-tetramethyl-1,3,2-dioxaborolan-2-yl)-5,6-dihydropyridine-1(2*H*)-carboxylate (230 mg, 0.745 mmol), 2-(3-bromophenyl)-*N*,*N*-dimethylethanamine (170 mg, 0.745 mmol) and Pd(dppf)Cl_2.CH_2Cl_2 (30 mg, 0.04 mmol) were reacted together in DME (3 mL) and aqueous sodium carbonate (1 M, 2 mL). Purification on a silica column eluting with 3% [7 M NH_3 in MeOH] in CH_2Cl_2 gave the product as a colourless oil (168 mg, 68%); ^{1}H NMR (500 MHz, $CDCl_3$) 1.49 (s, 9H), 2.32 (s, 6H), 2.50-2.58 (m, 4H), 2.78-2.81 (m, 2H), 3.62 (t, *J* = 5.5 Hz, 2H), 4.07 (br s, 2H), 6.05 (br s, 1H), 7.10 (dt, *J* = 7.4, 1.5 Hz, 1H), 7.21-7.24 (m, 3H); LC - MS (method C; ESI, *m/z*) t_R = 1.03 min – 331 $(M+H)^+$.

***tert*-Butyl 4-(3-(2-(dimethylamino)ethyl)phenyl)piperidine-1-carboxylate**

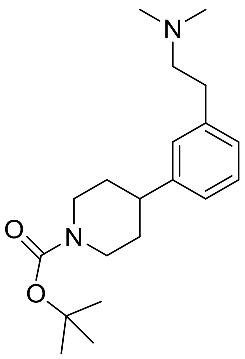

A solution of *tert*-butyl 4-(3-(2-(dimethylamino)ethyl)phenyl)-5,6-dihydropyridine-1(2*H*)-carboxylate (168 mg, 0.508 mmol) in ethanol (10 mL) was stirred in the presence of 10% palladium on carbon under an atmosphere of hydrogen for 2 h. The reaction mixture was filtered through a pad of celite, and the crude residue was passed through an isolute SCX-2 cartridge to give the reduced product (152 mg, 90%). This material was used in the next step without any further purification. LC - MS (method C; ESI, *m/z*) t_R = 1.04 min – 333 $(M+H)^+$.

***N,N*-Dimethyl-2-(3-(piperidin-4-yl)phenyl)ethanamine (25m)**

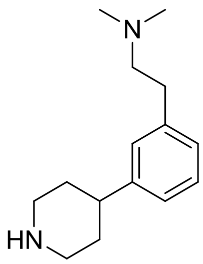

tert-Butyl 4-(3-(2-(dimethylamino)ethyl)phenyl)piperidine-1-carboxylate (152 mg, 0.457 mmol) was dissolved in a solution of HCl in dioxane (4M; 5 mL). The reaction mixture was stirred at room temperature for 2 h, then concentrated in vacuo and the residue was filtered through an isolute SCX-2 cartridge to provide the desired product as a colorless oil (97 mg, 91%); ^{1}H NMR (500 MHz, $CDCl_3$) 1.68 (qd, *J* = 12.3, 3.9 Hz, 2H), 1.79-1.87 (m, 2H), 2.31 (s, 6H), 2.52-2.55 (m, 2H), 2.60 (tt, *J* = 12.1, 3.7 Hz, 1H), 2.72-2.79 (m, 4H), 3.20 (d, *J* = 12

Hz, 2H), 7.04-7.07 (m, 3H), 7.23 (t, J = 7.7 Hz, 1H); LC - MS (method C; ESI, m/z) t_R = 0.22 min – 233 $(M+H)^+$.

8-(4-(2-(4-(3-(2-(Dimethylamino)ethyl)phenyl)piperidin-1-yl)ethyl)-1*H*-pyrazol-1-yl)-3-((2-(trimethylsilyl)ethoxy)methyl)pyrido[3,4-*d*]pyrimidin-4(3*H*)-one (12m)

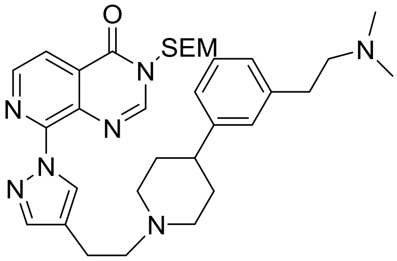

According to General Procedure 5, 2-(1-(4-oxo-3-((2-(trimethylsilyl)ethoxy)methyl)-3,4-dihydropyrido[3,4-*d*]pyrimidin-8-yl)-1*H*-pyrazol-4-yl)acetaldehyde (65 mg, 0.169 mmol), *N*,*N*-dimethyl-2-(3-(piperidin-4-yl)phenyl)ethanamine (39.2 mg, 0.169 mmol) and sodium triacetoxyborohydride (35.7 mg, 0.169 mmol) were reacted together in dichloromethane (4 mL). Following workup procedure A, the title compound was obtained as a colorless oil (44 mg, 43%); ^{1}H NMR (500 MHz, $CDCl_3$) 0.02 (s, 9H), 0.91-1.06 (m, 2H), 1.79-1.90 (m, 4H), 2.09-2.22 (m, 2H), 2.32 (s, 6H), 2.46-2.61 (m, 3H), 2.66-2.73 (m, 2H), 2.74-2.81 (m, 2H), 2.82-2.88 (m, 2H), 3.15 (dt, J = 12.2, 3.1 Hz, 2H), 3.64-3.74 (m, 2H), 5.46 (s, 2H), 7.00-7.13 (m, 3H), 7.22-7.25 (m, 1H), 7.81 (s, 1H), 8.07 (d, J = 5.1 Hz, 1H), 8.31 (s, 1H), 8.57 (s, 1H), 8.62 (d, J = 5.1 Hz, 1H); LC - MS (method C; ESI, m/z) t_R = 0.94 min – 602 $(M+H)^+$.

8-(4-(2-(4-(3-(2-(Dimethylamino)ethyl)phenyl)piperidin-1-yl)ethyl)-1*H*-pyrazol-1-yl)pyrido[3,4-*d*]pyrimidin-4(3*H*)-one (16m)

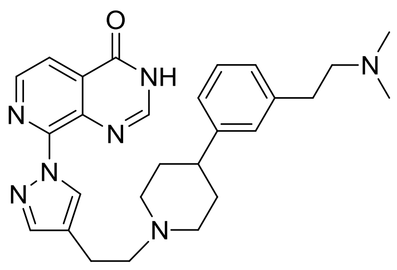

According to General Procedure 4, 8-(4-(2-(4-(3-(2-(dimethylamino)ethyl)phenyl)piperidin-1-yl)ethyl)-1*H*-pyrazol-1-yl)-3-((2-(trimethylsilyl)ethoxy)methyl)pyrido[3,4-*d*]pyrimidin-4(3*H*)-one (44 mg, 0.073 mmol) and hydrochloric acid (6 M, 1 mL) were reacted together in THF (1 mL) for 4 h. Purification was achieved by passing the crude product through an SCX-2 cartridge eluting first with methanol and then 7 N ammonia in methanol. Fractions containing the product were combined, concentrated *in vacuo*, and the residue triturated with Et_2O. The pale brown precipitate was obtained by filtration, and dried (20 mg, 58%). ^1H NMR (500 MHz, CD_3OD) 1.78-1.95 (m, 4H), 2.26-2.37 (m, 2H), 2.40 (s, 6H), 2.55-2.71 (m, 3H), 2.75-2.86 (m, 4H), 2.87-2.94 (m, 2H), 3.24 (dt, J = 12, 3.1 Hz, 2H), 7.08 (dt, J = 7.6, 1.4 Hz, 1H), 7.10-7.15 (m, 2H), 7.24 (t, J = 7.5 Hz, 1H), 7.81 (s, 1H), 8.06 (d, J = 5.2 Hz, 1H), 8.28 (s, 1H), 8.53 (d, J = 5.2 Hz, 1H), 8.77 (s, 1H) ; HRMS (Method D): t_R 0.97 min - found 472.2823; calculated for $C_{27}H_{34}N_7O$ $(M+H)^+$ 472.2825.

***tert*-Butyl 4-(4-(pyridin-3-yl)phenyl)-3,6-dihydropyridine-1(2*H*)-carboxylate (23b)**

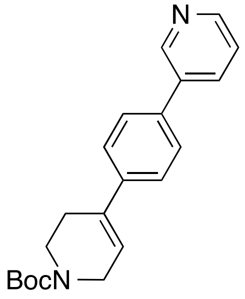

According to General Procedure 1, *tert*-butyl 4-(4,4,5,5-tetramethyl-1,3,2-dioxaborolan-2-yl)-5,6-dihydropyridine-1(2*H*)-carboxylate (300 mg, 0.970 mmol), 3-(4-bromophenyl)pyridine (227 mg, 0.970 mmol) and $Pd(dppf)Cl_2.CH_2Cl_2$ (79 mg, 0.097 mmol) were reacted together in DME (3 mL) and aqueous sodium carbonate (1 M, 2 mL). Purification on a KP-Sil snap cartridge (5% [0.2 M NH_3 in MeOH] in CH_2Cl_2) gave the product as a pale brown solid (301 mg, 92%); ^{1}H NMR (500 MHz, $CDCl_3$) 1.46 (s, 9H), 2.50 (br s, 2H), 3.61 (br t, J = 5.3 Hz, 2H), 4.06 (br s, 2H), 6.06 (br s, 1H), 7.27-7.31 (m, 1H), 7.41-7.44 (m, 2H), 7.48-7.51 (m, 2H), 7.79-7.82 (m, 1H), 8.52 (dd, J = 4.8, 1.6 Hz, 1H), 8.79-8.81 (m, 1H); LC - MS (method C; ESI, *m/z*) t_R = 1.42 min – 337 [$(M+H)^+$]; HRMS (method D): found 337.1915; calculated for $C_{21}H_{25}N_2O_2$ $(M+H)^+$ 337.1916.

3-(4-(Piperidin-4-yl)phenyl)pyridine (26b)

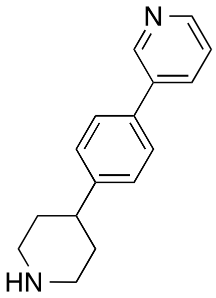

According to General Procedure 2, $Pd(OH)_2$ on carbon (34.1 mg, 0.243 mmol) and *tert*-butyl 4-(4-(pyridin-3-yl)phenyl)-5,6-dihydropyridine-1(2*H*)-carboxylate (204 mg, 0.606 mmol) were reacted together in EtOH (3 mL) and hydrochloric acid (1 M, 0.5 mL). The crude material from this reaction was then stirred in THF (2 mL) and hydrochloric acid (1 M, 2 mL) and purified by passing through an SCX-2 cartridge eluting with 1 M NH_3 in $MeOH/CH_2Cl_2$. The ammoniacal solution was concentrated *in vacuo* to yield the product as a pale brown solid (76.2 mg, 85%); ^{1}H NMR (500 MHz, MeOD) 1.87 (qd, J = 12.9, 3.6 Hz, 2H), 1.96-2.02 (m, 2H), 2.87 (tt, J = 12.1, 3.6 Hz, 1H), 3.00 (td, J = 12.5, 2.6 Hz, 2H), 3.36-3.41 (m, 2H), 7.39-7.42 (m, 2H), 7.50 (ddd, J = 7.0, 4.9, 0.6 Hz, 1H), 7.59-7.63 (m, 2H), 8.06 (ddd, J = 8.0,

2.2, 1.7 Hz, 1H), 8.50 (dd, *J* = 4.9, 1.7 Hz, 1H), 8.77 (d, *J* = 2.2, 1H), NH signal not observed; LC - MS (method C; ESI, *m/z*) t_R = 0.40 min – 239 [$(M+H)^+$]; HRMS (method D): found 239.1547; calculated for $C_{16}H_{19}N_2$ $(M+H)^+$ 239.1548.

8-(4-(2-(4-(4-(Pyridin-3-yl)phenyl)piperidin-1-yl)ethyl)-1*H*-pyrazol-1-yl)-3-((2-(trimethylsilyl)ethoxy)methyl)pyrido[3,4-*d*]pyrimidin-4(3*H*)-one (13b)

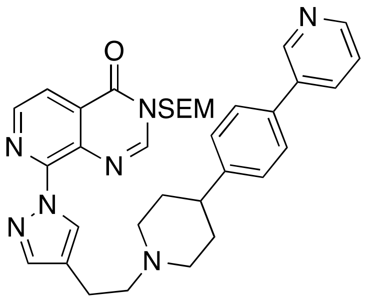

According to General Procedure 3, triethylamine (0.06 mL, 0.425 mmol), 2-(1-(4-oxo-3-((2-(trimethylsilyl)ethoxy)methyl)-3,4-dihydropyrido[3,4-*d*]pyrimidin-8-yl)-1*H*-pyrazol-4-yl)-ethyl methanesulfonate (99 mg, 0.213 mmol) and 3-(4-(piperidin-4-yl)phenyl)pyridine (76 mg, 0.319 mmol) were reacted together in anhydrous DMF (1 mL). Purification on a KP-Sil snap cartridge (15% [0.2 M NH_3 in MeOH] in CH_2Cl_2) gave the product as a pale yellow oil (62.8 mg, 49%); ^{1}H NMR (500 MHz, $CDCl_3$) 0.01 (s, 9H), 0.95-1.01 (m, 2H), 1.83-1.95 (m, 4H), 2.20 (td, *J* = 11.3, 3.3 Hz, 2H), 2.60 (tt, *J* = 11.3, 4.9 Hz, 1H), 2.69-2.75 (m, 2H), 2.82-2.88 (m, 2H), 3.18 (br d, *J* = 11.3 Hz, 2H), 3.66-3.72 (m, 2H), 5.46 (s, 2H), 7.32-7.38 (m, 3H), 7.51-7.55 (m, 2H), 7.81 (s, 1H), 7.86 (ddd, *J* = 7.9, 2.3, 1.7 Hz, 1H), 8.06 (d, *J* = 5.1 Hz, 1H), 8.31 (s, 1H), 8.55-8.58 (m, 2H), 8.63 (d, *J* = 5.1 Hz, 1H), 8.84 (dd, *J* = 2.3, 0.7 Hz, 1H); LC - MS (method C; ESI, *m/z*) t_R = 1.11 min – 478 [$(M-SEM+2H)^+$]; HRMS (method D): found 478.2436; calculated for $C_{28}H_{28}N_7O$ $(M-SEM+2H)^+$ 478.2355.

8-(4-(2-(4-(4-(Pyridin-3-yl)phenyl)piperidin-1-yl)ethyl)-1*H*-pyrazol-1-yl)pyrido[3,4-*d*]pyrimidin-4(3*H*)-one (17b)

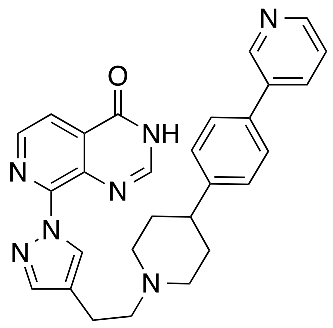

According to General Procedure 4, 8-(4-(2-(4-(4-(pyridin-3-yl)phenyl)piperidin-1-yl)ethyl)-1*H*-pyrazol-1-yl)-3-((2-(trimethylsilyl)ethoxy)methyl)pyrido[3,4-*d*]pyrimidin-4(3*H*)-one (51 mg, 0.084 mmol) and hydrochloric acid (6 M, 1 mL) were reacted together in THF (1 mL). Purification on a KP-NH snap cartridge (40% EtOH in CH_2Cl_2) gave the title product as a white solid (28.3 mg, 71%); ^{1}H NMR (500 MHz, DMSO-d_6) 1.72 (qd, J = 12.4, 3.4 Hz, 2H), 1.78-1.84 (m, 2H), 2.11 (t, J = 11.4 Hz, 2H), 2.54-2.63 (m, 3H), 2.70-2.76 (m, 2H), 3.10 (br d, J = 11.4 Hz, 2H), 7.40 (d, J = 8.3 Hz, 2H), 7.47 (ddd, J = 7.9, 4.7, 0.7 Hz, 1H), 7.64-7.67 (m, 2H), 7.74 (s, 1H), 7.98 (d, J = 5.1 Hz, 1H), 8.05 (ddd, J = 7.9, 2.4, 1.6 Hz, 1H), 8.29 (s, 1H), 8.44 (s, 1H), 8.54-8.56 (m, 2H), 8.87 (dd, J = 2.4, 0.7 Hz, 1H), 12.78 (br s, 1H); LC - MS (method C; ESI, *m/z*) t_R = 0.65 min – 478 [(M+H)$^+$]; HRMS (method D): found 478.2352; calculated for $C_{28}H_{28}N_7O$ (M+H)$^+$ 478.2355.

***tert*-Butyl 4-(4-(pyrimidin-5-yl)phenyl)piperidine-1-carboxylate**

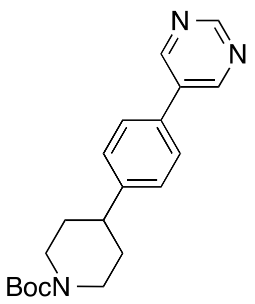

According to General Procedure 1, 5-(4,4,5,5-tetramethyl-1,3,2-dioxaborolan-2-yl)pyrimidine (162 mg, 0.786 mmol), *tert*-butyl 4-(4-bromophenyl)piperidine-1-carboxylate (268 mg, 0.786 mmol) and Pd(dppf)Cl_2.CH_2Cl_2 (64 mg, 0.079 mmol) were reacted together in DME (3 mL) and aqueous sodium carbonate (1 M, 2 mL). Purification on a KP-Sil snap cartridge (15% EtOAc in cyclohexane) gave the product as a pale yellow oil (278 mg, quant.); ^{1}H NMR (500 MHz, $CDCl_3$) 1.41 (s, 9H), 1.58 (qd, *J* = 12.6, 4.0 Hz, 2H), 1.77 (br d, *J* = 12.6 Hz, 2H), 2.65 (tt, *J* = 12.2, 3.4 Hz, 1H), 2.75 (br s, 2H), 4.20 (br s, 2H), 7.26-7.30 (m, 2H), 7.43-7.47 (m, 2H), 8.85 (s, 2H), 9.08 (s, 1H); LC - MS (method C; ESI, *m/z*) t_R = 1.41 min – 340 [$(M+H)^+$]; HRMS (method D): found 340.2014; calculated for $C_{20}H_{26}N_3O_2$ $(M+H)^+$ 340.2025.

5-(4-(Piperidin-4-yl)phenyl)pyrimidine (26c)

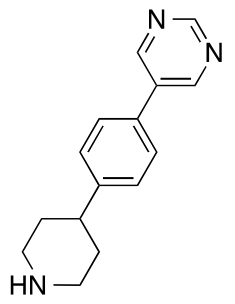

tert-Butyl 4-(4-(pyrimidin-5-yl)phenyl)piperidine-1-carboxylate (177 mg, 0.521 mmol) was dissolved in a mixture of THF (3 mL) and hydrochloric acid (1 M, 3 mL). The reaction mixture was stirred at 50 °C for 2 h and monitored by LCMS. On completion of the reaction the reaction mixture was concentrated *in vacuo* and the residue redissolved in MeOH/CH_2Cl_2. The crude material was passed through an SCX-2 cartridge eluting with 1 M NH_3 in MeOH/CH_2Cl_2. The ammoniacal solution was concentrated *in vacuo* to yield the product as a white solid (90.6 mg, 73%); ^{1}H NMR (500 MHz, $CDCl_3$) 1.62 (qd, *J* = 12.5, 3.7 Hz, 2H), 1.70 (br s, 1H), 1.81 (br d, *J* = 12.5 Hz, 2H), 2.63 (tt, *J* = 12.1, 3.7 Hz, 1H), 2.71 (td, *J* = 12.1, 2.3 Hz, 2H), 3.16 (br d, *J* = 12.1 Hz, 2H), 7.31-7.34 (m, 2H), 7.45-7.50 (m, 2H), 8.88 (s, 2H),

9.12 (s, 1H); LC - MS (method C; ESI, *m/z*) t_R = 0.60 min – 240 [(M+H)$^+$]; HRMS (method D): found 240.1505; calculated for $C_{15}H_{18}N_3$ (M+H)$^+$ 240.1500.

8-(4-(2-(4-(4-(Pyrimidin-5-yl)phenyl)piperidin-1-yl)ethyl)-1*H*-pyrazol-1-yl)-3-((2-(trimethylsilyl)ethoxy)methyl)pyrido[3,4-*d*]pyrimidin-4(3*H*)-one (13c)

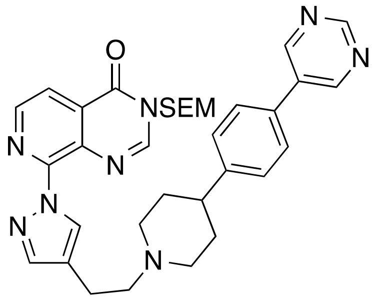

According to General Procedure 3, triethylamine (0.05 mL, 0.332 mmol), 2-(1-(4-oxo-3-((2-(trimethylsilyl)ethoxy)methyl)-3,4-dihydropyrido[3,4-*d*]pyrimidin-8-yl)-1*H*-pyrazol-4-yl)-ethyl methanesulfonate (77 mg, 0.166 mmol) and 5-(4-(piperidin-4-yl)phenyl)pyrimidine (116 mg, 0.484 mmol) were reacted together in anhydrous DMF (1 mL). Purification on a KP-Sil snap cartridge (6% [0.2 M NH_3 in MeOH] in CH_2Cl_2) gave the product as a pale yellow oil (51.2 mg, 51%); ^{1}H NMR (500 MHz, $CDCl_3$) 0.01 (s, 9H), 0.96-1.01 (m, 2H), 1.82-1.98 (m, 4H), 2.21 (td, *J* = 11.2, 2.6 Hz, 2H), 2.63 (tt, *J* = 11.2, 4.6 Hz, 1H), 2.70-2.75 (m, 2H), 2.82-2.89 (m, 2H), 3.19 (br d, *J* = 11.2 Hz, 2H), 3.66-3.72 (m, 2H), 5.47 (s, 2H), 7.41 (d, *J* = 7.9 Hz, 2H), 7.55 (d, *J* = 7.9 Hz, 2H), 7.82 (br s, 1H), 8.07 (d, *J* = 5.1 Hz, 1H), 8.32 (s, 1H), 8.58 (s, 1H), 8.63 (d, *J* = 5.1 Hz, 1H), 8.95 (s, 2H), 9.19 (s, 1H); LC - MS (method C; ESI, *m/z*) t_R = 1.15 min – 609 [(M+H)$^+$]; HRMS (method D): found 609.3102; calculated for $C_{33}H_{41}N_8O_2Si$ (M+H)$^+$ 609.3121.

8-(4-(2-(4-(4-(Pyrimidin-5-yl)phenyl)piperidin-1-yl)ethyl)-1*H*-pyrazol-1-yl)pyrido[3,4-*d*]pyrimidin-4(3*H*)-one (17c)

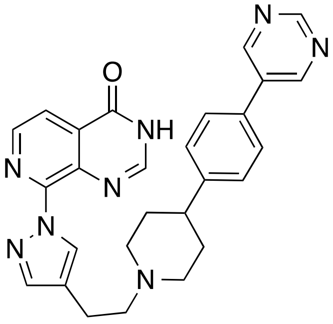

According to General Procedure 4, 8-(4-(2-(4-(4-(pyridin-3-yl)phenyl)piperidin-1-yl)ethyl)-1*H*-pyrazol-1-yl)-3-((2-(trimethylsilyl)ethoxy)methyl)pyrido[3,4-*d*]pyrimidin-4(3*H*)-one (51 mg, 0.084 mmol) and hydrochloric acid (6 M, 1 mL) were reacted together in THF (1 mL). Purification on a KP-NH snap cartridge (40% EtOH in CH_2Cl_2) gave the title compound as a white solid (28.3 mg, 71%); ^{1}H NMR (500 MHz, DMSO-d_6) 1.72 (qd, J = 12.2, 3.4 Hz, 2H), 1.78-1.84 (m, 2H), 2.11 (t, J = 11.5 Hz, 2H), 2.56-2.64 (m, 3H), 2.70-2.76 (m, 2H), 3.10 (br d, J = 11.5 Hz, 2H), 7.44 (d, J = 7.8 Hz, 2H), 7.72-7.76 (m, 3H), 7.98 (d, J = 5.1 Hz, 1H), 8.29 (s, 1H), 8.44 (s, 1H), 8.55 (d, J = 5.1 Hz, 1H), 9.12 (s, 2H), 9.17 (s, 1H), 12.79 (br s, 1H); ^{13}C NMR (150 MHz, DMSO-d_6) 21.9, 33.4, 42.0, 54.0, 59.4, 118.3, 121.0, 127.4, 128.2, 131.2, 131.5, 132.0, 133.6, 142.2, 144.5, 147.7, 147.9, 155.0, 157.5; LC - MS (method C; ESI, *m/z*) t_R = 0.79 min – 479 [(M+H)$^+$]; HRMS (method D): found 479.2312; calculated for $C_{27}H_{27}N_8O$ (M+H)$^+$ 479.2308.

1-(4-(Piperidin-4-yl)phenyl)pyrrolidin-2-one

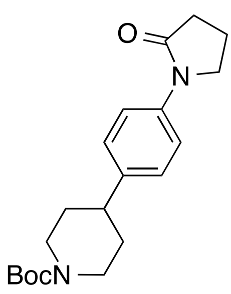

Pd_2dba_3 (16.2 mg, 0.018 mmol), Xantphos (7.7 mg, 0.013 mmol), cesium carbonate (402 mg, 1.23 mmol) and *tert*-butyl 4-(4-bromophenyl)piperidine-1-carboxylate (300 mg, 0.88

mmol) were added to a microwave vial which was sealed, evacuated and placed under N_2. Pyrrolidin-2-one (0.1 mL, 1.32 mmol) and 1,4-dioxane (2 mL) were added into the vial and the reaction mixture was heated thermally at 100 °C for 16 h. The reaction mixture was concentrated onto silica to load onto a KP-Sil snap cartridge for purification (50% EtOAc in cyclohexane) to give the product as a pale orange solid (148.5 mg, 49%); ^{1}H NMR (500 MHz, $CDCl_3$) 1.43 (s, 9H), 1.53 (qd, *J* = 12.8, 4.1 Hz, 2H), 1.73 (br d, *J* = 12.8 Hz, 2H), 2.06 (quintet, *J* = 7.4 Hz, 2H), 2.50 (t, *J* = 8.0 Hz, 2H), 2.57 (tt, *J* = 12.2, 3.5 Hz, 1H), 2.73 (br s, 2H), 3.76 (t, *J* = 7.0 Hz, 2H), 4.17 (br s, 2H), 7.12-7.15 (m, 2H), 7.46-7.50 (m, 2H); LC - MS (method C; ESI, *m/z*) t_R = 1.39 min – 289 [$(M-{}^tBu+2H)^+$]; HRMS (method D): found 289.1559; calculated for $C_{16}H_{21}N_2O_3$ $(M-{}^tBu+2H)^+$ 289.1552.

***tert*-Butyl 4-(4-(2-oxopyrrolidin-1-yl)phenyl)piperidine-1-carboxylate (26d)**

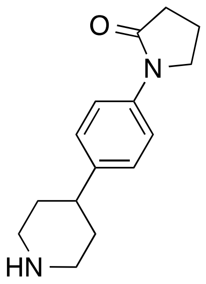

tert-Butyl 4-[4-(2-oxopyrrolidin-1-yl)phenyl]piperidine-1-carboxylate (227 mg, 0.660 mmol) was dissolved in a mixture of THF (3 mL) and hydrochloric acid (6 M, 3 mL). The reaction mixture was stirred at 50 °C for 1 h and monitored by LCMS. On completion of the reaction, the reaction mixture was concentrated *in vacuo* and the residue redissolved in MeOH/CH_2Cl_2. The crude material was passed through an SCX-2 cartridge eluting with 1 M NH_3 in MeOH/CH_2Cl_2. The ammoniacal solution was concentrated *in vacuo* to yield the product as a white solid (130 mg, 79%); ^{1}H NMR (500 MHz, $CDCl_3$) 1.59 (qd, *J* = 12.4, 3.8 Hz, 2H), 1.77 (br d, *J* = 13.2 Hz, 2H), 2.09 (quintet, *J* = 7.5 Hz, 2H), 2.28 (br s, 1H), 2.51-2.60 (m, 3H), 2.69 (td, *J* = 12.4, 2.5 Hz, 2H), 3.14 (br d, *J* = 12.4 Hz, 2H), 3.79 (t, *J* = 7.0 Hz, 2H), 7.16-

7.20 (m, 2H), 7.46-7.50 (m, 2H); LC - MS (method C; ESI, *m/z*) t_R = 0.55 min – 245 [(M+H)$^+$]; HRMS (method D): found 245.1660; calculated for $C_{15}H_{21}N_2O$ (M+H)$^+$ 245.1654.

8-(4-(2-(4-(4-(2-Oxopyrrolidin-1-yl)phenyl)piperidin-1-yl)ethyl)-1*H*-pyrazol-1-yl)-3-((2-(trimethylsilyl)ethoxy)methyl)pyrido[3,4-*d*]pyrimidin-4(3*H*)-one (13d)

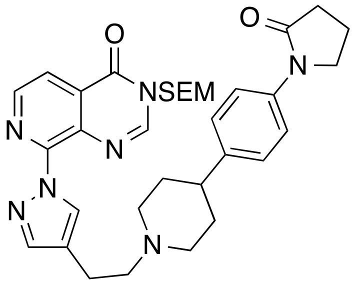

According to General Procedure 3, triethylamine (0.06 mL, 0.458 mmol), 2-(1-(4-oxo-3-((2-(trimethylsilyl)ethoxy)methyl)-3,4-dihydropyrido[3,4-*d*]pyrimidin-8-yl)-1*H*-pyrazol-4-yl)-ethyl methanesulfonate (80 mg, 0.172 mmol) and 1-[4-(4-piperidyl)phenyl]pyrrolidin-2-one (63 mg, 0.258 mmol) were reacted together in anhydrous DMF (1 mL). Purification on a KP-Sil snap cartridge (5% [0.2 M NH_3 in MeOH] in CH_2Cl_2) gave the product as a pale yellow oil (82.4 mg, 78%); ^{1}H NMR (500 MHz, $CDCl_3$) 0.01 (s, 9H), 0.93-1.00 (m, 2H), 1.75-1.89 (m, 4H), 2.09-2.19 (m, 4H), 2.51 (tt, *J* = 11.5, 4.5 Hz, 1H), 2.58 (t, *J* = 8.1 Hz, 2H), 2.66-2.71 (m, 2H), 2.80-2.85 (m, 2H), 3.14 (br d, *J* = 11.5 Hz, 2H), 3.65-3.70 (m, 2H), 3.83 (t, *J* = 7.0 Hz, 2H), 5.45 (s, 2H), 7.21-7.25 (m, 2H), 7.49-7.53 (m, 2H), 7.79 (s, 1H), 8.04 (d, *J* = 5.1 Hz, 1H), 8.29 (s, 1H), 8.57 (s, 1H), 8.61 (d, *J* = 5.1 Hz, 1H); LC - MS (method C; ESI, *m/z*) t_R = 1.19 min – 614 [(M+H)$^+$]; HRMS (method D): found 614.3263; calculated for $C_{33}H_{44}N_7O_3Si$ (M+H)$^+$ 614.3275.

8-(4-(2-(4-(4-(2-Oxopyrrolidin-1-yl)phenyl)piperidin-1-yl)ethyl)-1*H*-pyrazol-1-yl)pyrido[3,4-*d*]pyrimidin-4(3*H*)-one (17d)

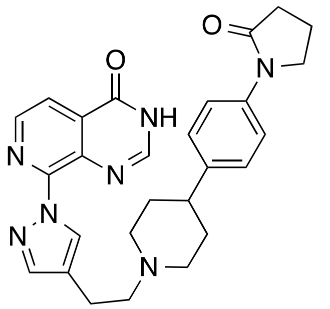

According to General Procedure 4, 8-[4-[2-[4-[4-(2-oxopyrrolidin-1-yl)phenyl]-1-piperidyl]ethyl]pyrazol-1-yl]-3-(2-trimethylsilylethoxymethyl)pyrido[3,4-*d*]pyrimidin-4-one (84.2 mg, 0.137 mmol) and hydrochloric acid (6 M, 1.5 mL) were reacted together in THF (1.5 mL). Purification on a KP-NH snap cartridge (40% EtOH in CH_2Cl_2) gave the title product as a white solid (40.1 mg, 57%); ^1H NMR (500 MHz, DMSO-d_6) 1.65 (qd, J = 12.4, 3.5 Hz, 2H), 1.72-1.78 (m, 2H), 2.01-2.11 (m, 4H), 2.45-2.49 (m, 3H), 2.56-2.60 (m, 2H), 2.68-2.74 (m, 2H), 3.07 (br d, J = 11.5 Hz, 2H), 3.81 (t, J = 7.0 Hz, 2H), 7.25 (d, J = 8.6 Hz, 2H), 7.55 (d, J = 8.6 Hz, 2H), 7.72 (s, 1H), 7.97 (d, J = 5.1 Hz, 1H), 8.28 (s, 1H), 8.43 (s, 1H), 8.54 (d, J = 5.1 Hz, 1H), 12.78 (br s, 1H); ^{13}C NMR (150 MHz, DMSO-d_6) 17.9, 21.9, 32.7, 33.6, 41.7, 48.6, 54.1, 59.4, 112.4, 118.3, 120.0, 121.0, 127.2, 127.5, 131.1, 131.5, 138.1, 142.2, 142.3, 144.5, 147.9, 174.0; LC - MS (method C; ESI, *m/z*) t_R = 0.79 min – 484 $[(M+H)^+]$; HRMS (method D): found 484.2454; calculated for $C_{27}H_{30}N_7O_2$ $(M+H)^+$ 484.2461.

4-(4-Bromophenethyl)morpholine

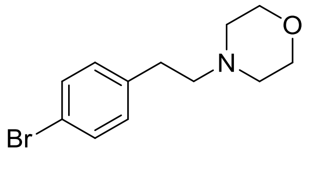

A mixture of morpholine (87 mg, 1.0 mmol) and 2-(4-bromophenyl)acetaldehyde (200 mg, 1.0 mmol) was stirred in dry CH_2Cl_2 (10 mL) for 10 min. Sodium triacetoxyborohydride (211 mg, 1.0 mmol) was then added and the reaction mixture was stirred at room temperature for

1 h, then it was washed with saturated sodium bicarbonate solution (10 mL), dried (Na_2SO_4) and concentrated in vacuo. Purification of the crude product on a silica column eluting with 5% [7 M NH_3 in MeOH] in ethyl acetate afforded the desired product (188 mg, 70%); ^{1}H NMR (500 MHz, CD_3OD) 2.55-2.61 (m, 6H), 2.79 (t, J = 5.5 Hz, 2H), 3.72 (t, J = 4.7 Hz, 4H), 7.16 (d, J = 8.4 Hz, 2H), 7.42 (d, J = 8.4 Hz, 2H). LC - MS (method C; ESI, *m/z*) t_R = 0.65 min – 270, 272 [$(M+H)^+$, Br isotopic pattern].

***Tert*-butyl 4-(4-(2-morpholinoethyl)phenyl)piperidine-1-carboxylate**

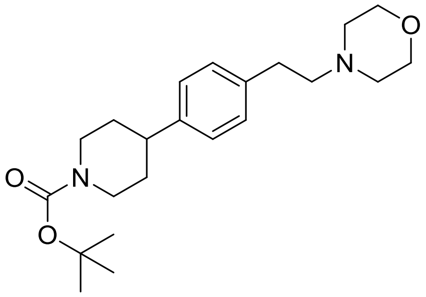

According to General Procedure 1, *tert*-butyl 4-(4,4,5,5-tetramethyl-1,3,2-dioxaborolan-2-yl)-5,6-dihydropyridine-1(2*H*)-carboxylate (215 mg, 0.70 mmol), 4-(4-bromophenethyl)morpholine (188 mg, 0.70 mmol) and Pd(dppf)Cl_2.CH_2Cl_2 (30 mg, 0.04 mmol) were reacted together in DME (3 mL) and aqueous sodium carbonate (1 M, 2 mL). Purification on a silica column eluting with 5% [7 M NH_3 in MeOH] in ethyl acetate gave *tert*-butyl 4-(4-(2-morpholinoethyl)phenyl)-5,6-dihydropyridine-1(2*H*)-carboxylate (**23e**) as a colourless oil (178 mg). LC - MS (method C; ESI, *m/z*) t_R = 1.06 min – 373 $(M+H)^+$. A solution of this material (178 mg, 0.478 mmol) in ethanol (10 mL) was stirred in the presence of 10% palladium on carbon under an atmosphere of hydrogen for 2 h. The reaction mixture was filtered through a pad of celite and the crude residue was passed through an isolute SCX-2 cartridge to give the reduced product (162 mg, 62%); ^{1}H NMR (500 MHz, $CDCl_3$) 1.49 (s, 9H), 1.51-1.63 (m, 2H), 1.80-1.83 (m, 2H), 2.43-2.66 (m, 9H), 2.74-2.88

(m, 4H), 3.76 (t, J = 4.7 Hz, 4H), 7.14-7.16 (m, 4H); LC - MS (method C; ESI, m/z) t_R = 1.03 min – 375 $(M+H)^+$.

4-(4-(Piperidin-4-yl)phenethyl)morpholine (26e)

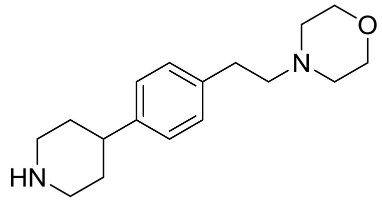

To a solution of *tert*-butyl 4-(4-(2-morpholinoethyl)phenyl)piperidine-1-carboxylate (162 mg, 0.433 mmol) in dry dioxane (4 mL), a solution of HCl in dioxane (4M, 10 mL) was added and the reaction mixture was stirred at room temperature for 2 h. The solution was then concentrated in vacuo and the residue was filtered through an isolute SCX-2 cartridge to give the title compound as a colorless oil (102 mg, 86%); ^{1}H NMR (500 MHz, $CDCl_3$) 1.64 (qd, J = 12.8, 3.9 Hz, 2H), 1.81-1.84 (m, 2H), 2.39 (br s, 1H), 2.53 (br s, 4H), 2.54-2.57 (m, 3H), 2.70-2.84 (m, 4H), 3.18 (d, J = 12.5 Hz, 2H), 3.74 (t, J = 4.6 Hz, 4H), 7.15 (br s, 4H); LC - MS (method C; ESI, m/z) t_R = 1.03 min – 275 $(M+H)^+$.

8-(4-(2-(4-(4-(2-Morpholinoethyl)phenyl)piperidin-1-yl)ethyl)-1*H*-pyrazol-1-yl)-3-((2-(trimethylsilyl)ethoxy)methyl)pyrido[3,4-*d*]pyrimidin-4(3*H*)-one (13a)

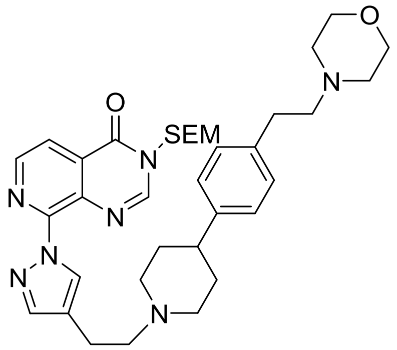

According to General Procedure 5, 2-(1-(4-oxo-3-((2-(trimethylsilyl)ethoxy)methyl)-3,4-dihydropyrido[3,4-*d*]pyrimidin-8-yl)-1*H*-pyrazol-4-yl)acetaldehyde (80 mg, 0.208 mmol), 4-

(4-(piperidin-4-yl)phenethyl)morpholine (102 mg, 0.372 mmol) and sodium triacetoxyborohydride (52.8 mg, 0.25 mmol) were reacted together in dichloromethane (4 mL). Following workup procedure A, the title compound was obtained as a colorless oil (76 mg, 57%). LC - MS (method C; ESI, *m/z*) t_R = 0.94 min – 644 $(M+H)^+$. This material was used in the next step without any further purification.

8-(4-(2-(4-(4-(2-Morpholinoethyl)phenyl)piperidin-1-yl)ethyl)-1*H*-pyrazol-1-yl)pyrido[3,4-*d*]pyrimidin-4(3*H*)-one (17e)

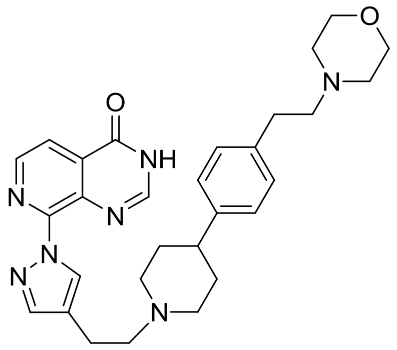

According to General Procedure 4, 8-(4-(2-(4-(4-(2-morpholinoethyl)phenyl)piperidin-1-yl)ethyl)-1*H*-pyrazol-1-yl)-3-((2-(trimethylsilyl)ethoxy)methyl)pyrido[3,4-*d*]pyrimidin-4(3*H*)-one (76 mg, 0.118 mmol) and hydrochloric acid (6 M, 1 mL) were reacted together in THF (1 mL) for 4 h. Purification was achieved by passing the crude product through an SCX-2 cartridge eluting first with methanol and then 7 N ammonia in methanol. Fractions containing the product were combined, concentrated *in vacuo*, and the residue triturated with Et_2O. The beige precipitate was obtained by filtration and dried (9 mg, 15%). ^{1}H NMR (500 MHz, CD_3OD) 1.78-1.94 (m, 4H), 2.36 (td, *J* = 11.9, 3 Hz, 2H), 2.53-2.66 (m, 6H), 2.76-2.94 (m, 5H), 3.23-3.29 (m, 2H), 3.30-3.33 (m, 2H), 3.73 (t, *J* = 4.6 Hz, 4H), 7.16-7.21 (m, 4H), 7.82 (s, 1H), 8.05 (d, *J* = 5.1 Hz, 1H), 8.27 (s, 1H), 8.54 (d, *J* = 5.2 Hz, 1H), 8.77 (s, 1H); HRMS (Method D): t_R 0.92 min - found 514.2954; calculated for $C_{29}H_{36}N_7O_2$ $(M+H)^+$ 514.2930.

2-(4-Bromophenyl)-*N*,*N*-dimethylethanamine

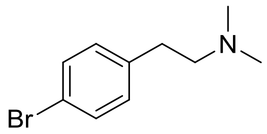

To a stirred solution of 2-(4-bromophenyl)acetaldehyde (200 mg, 1.0 mmol) in dry CH_2Cl_2 (10 mL) was added a solution of dimethylamine in THF (2 M, 0.5 mL, 1.0 mmol). After 10 min, sodium triacetoxyborohydride (212 mg, 1.0 mmol) was added and stirring was continued at room temperature for 1 h. The reaction mixture was then washed with saturated aqueous sodium bicarbonate, dried and concentrated in vacuo. The crude was purified on a silica column eluting with 5% [7 M NH_3 in MeOH] in ethyl acetate to give the product as a brown oil (180 mg, 79%); ^{1}H NMR (500 MHz, CD_3OD) 2.31 (s, 6H), 2.55 (t, *J* = 6.2 Hz, 2H), 2.76 (t, *J* = 6.2 Hz, 2H), 7.14 (d, *J* = 8.4 Hz, 2H), 7.42 (d, *J* = 8.4 Hz, 2H); LC - MS (method C; ESI, *m/z*) t_R = 0.61 min – 228, 230 $[(M+H)^+$, Br isotopic pattern].

***tert*-Butyl 4-(4-(2-(dimethylamino)ethyl)phenyl)piperidine-1-carboxylate**

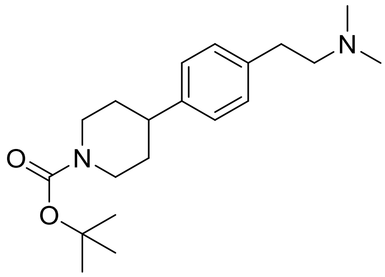

According to General Procedure 1, *tert*-butyl 4-(4,4,5,5-tetramethyl-1,3,2-dioxaborolan-2-yl)-5,6-dihydropyridine-1(2*H*)-carboxylate (244 mg, 0.789 mmol), 2-(4-bromophenyl)acetaldehyde (180 mg, 0.789 mmol) and Pd(dppf)Cl_2.CH_2Cl_2 (30 mg, 0.04 mmol) were reacted together in DME (3 mL) and aqueous sodium carbonate (1 M, 2 mL). Purification on a silica column eluting with 5% [7 M NH_3 in MeOH] in ethyl acetate gave *tert*-butyl 4-(4-(2-(dimethylamino)ethyl)phenyl)-5,6-dihydropyridine-1(2*H*)-carboxylate as a

white solid (159 mg). LC - MS (method C; ESI, *m/z*) t_R = 1.05 min – 331 $(M+H)^+$. This material (159 mg, 0.481 mmol) was dissolved in ethanol (10 mL) and the reaction mixture was stirred in the presence of 10% palladium on charcoal under an atmosphere of hydrogen for 2 h. The reaction mixture was then filtered through a pad of celite and the crude residue was passed through an isolute SCX-2 cartridge to give the reduced product (148 mg, 57%); ^{1}H NMR (500 MHz, $CDCl_3$) 1.49 (s, 9H), 1.58-1.62 (m, 2H), 1.78-1.82 (m, 2H), 2.31 (s, 6H), 2.50-2.56 (m, 2H), 2.58-2.65 (m, 1H), 2.72-2.87 (m, 4H), 4.21 (br s, 2H), 7.12-7.15 (m, 4H); LC - MS (method C; ESI, *m/z*) t_R = 1.05 min – 333 $(M+H)^+$.

***N,N*-Dimethyl-2-(4-(piperidin-4-yl)phenyl)ethanamine (26f)**

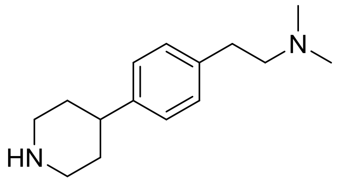

To a solution of *tert*-butyl 4-(4-(2-(dimethylamino)ethyl)phenyl)piperidine-1-carboxylate (148 mg, 0.445 mmol) in dry dioxane (4 mL) was added a solution of HCl in dioxane (4M, 10 mL), and the reaction mixture was stirred at room temperature for 2 h. The solution was then concentrated in vacuo, and the residue filtered through an isolute SCX-2 cartridge to give the product as a colorless oil (96 mg, 93%). ^{1}H NMR (500 MHz, $CDCl_3$) 1.62-1.66 (m, 2H), 1.80-1.83 (m, 2H), 2.31 (s, 6H), 2.52-2.56 (m, 2H), 2.57-2.64 (m, 1H), 2.75-2.78 (m, 4H), 3.20 (d, *J* = 11.9 Hz, 2H), 7.16 (br s, 4H); LC - MS (method C; ESI, *m/z*) t_R = 0.21 min – 233 $(M+H)^+$.

8-(4-(2-(4-(4-(2-(Dimethylamino)ethyl)phenyl)piperidin-1-yl)ethyl)-1*H*-pyrazol-1-yl)-3-((2-(trimethylsilyl)ethoxy)methyl)pyrido[3,4-*d*]pyrimidin-4(3*H*)-one (13f)

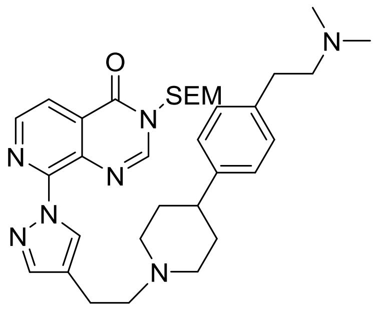

According to General Procedure 5, 2-(1-(4-oxo-3-((2-(trimethylsilyl)ethoxy)methyl)-3,4-dihydropyrido[3,4-*d*]pyrimidin-8-yl)-1*H*-pyrazol-4-yl)acetaldehyde (100 mg, 0.259 mmol), *N*,*N*-dimethyl-2-(4-(piperidin-4-yl)phenyl)ethanamine (96 mg, 0.413 mmol) and sodium triacetoxyborohydride (66 mg, 0.311 mmol) were reacted together in dichloromethane (4 mL). Following workup procedure A, the title compound was obtained as a colorless oil. (70 mg, 45%); LC - MS (method C; ESI, *m/z*) t_R = 0.94 min – 602 $(M+H)^+$. This material was used in the next step without any further purification.

8-(4-(2-(4-(4-(2-(Dimethylamino)ethyl)phenyl)piperidin-1-yl)ethyl)-1*H*-pyrazol-1-yl)pyrido[3,4-*d*]pyrimidin-4(3*H*)-one (17f)

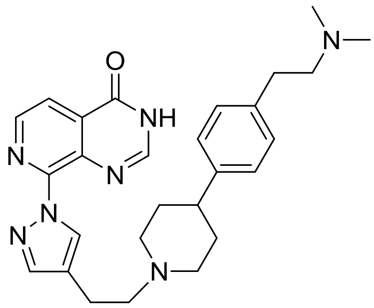

According to General Procedure 4, 8-(4-(2-(4-(4-(2-(dimethylamino)ethyl)phenyl)piperidin-1-yl)ethyl)-1*H*-pyrazol-1-yl)-3-((2-(trimethylsilyl)ethoxy)methyl)pyrido[3,4-*d*]pyrimidin-4(3*H*)-one (70 mg, 0.116 mmol) and hydrochloric acid (6 M, 1 mL) were reacted together in THF (1 mL) for 4 h. Purification was achieved by passing the crude product through an SCX-2 cartridge eluting first with methanol and then 7N ammonia in methanol. Fractions containing the product were combined, concentrated *in vacuo*, and the residue triturated with

Et_2O. The beige precipitate was obtained by filtration and dried (8 mg, 15%). ^{1}H NMR (500 MHz, DMSO-*d6*) 1.60-1.79 (m, 4H), 2.06-2.15 (m, 2H), 2.26 (s, 6H), 2.43-2.48 (m obscured by DMSO peak), 2.51-2.64 (m, 4H), 2.66-2.76 (m, 4H), 3.08 (d, *J* = 11.1 Hz, 2H), 7.09-7.17 (m, 4H), 7.73 (s, 1H), 7.99 (d, *J* = 5.1 Hz, 1H), 8.29 (s, 1H), 8.42 (s, 1H), 8.56 (d, *J* = 5.1 Hz, 1H); HRMS (Method D): t_R 0.93 min - found 472.2827; calculated for $C_{27}H_{34}N_7O$ $(M+H)^+$ 472.2825.

***tert*-Butyl 4-(4-(pyridin-4-yl)phenyl)-3,6-dihydropyridine-1(2*H*)-carboxylate (23g)**

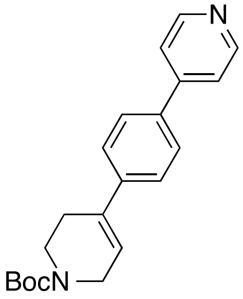

According to General Procedure 1, *tert*-butyl 4-(4,4,5,5-tetramethyl-1,3,2-dioxaborolan-2-yl)-5,6-dihydropyridine-1(2*H*)-carboxylate (250 mg, 0.809 mmol), 4-(4-bromophenyl)pyridine (189 mg, 0.809 mmol) and $Pd(dppf)Cl_2.CH_2Cl_2$ (66 mg, 0.081 mmol) were reacted together in DME (3 mL) and aqueous sodium carbonate (1 M, 2 mL). Purification on a KP-Sil snap cartridge (5% [0.2 M NH_3 in MeOH] in CH_2Cl_2) gave the product as a pale yellow solid (109 mg, 40%); ^{1}H NMR (500 MHz, $CDCl_3$) 1.49 (s, 9H), 2.54 (br s, 2H), 3.65 (br t, *J* = 5.3 Hz, 2H), 4.10 (br s, 2H), 6.12 (br s, 1H), 7.45-7.50 (m, 4H), 7.59-7.62 (m, 2H), 8.62-8.65 (m, 2H); LC - MS (method C; ESI, *m/z*) t_R = 1.24 min – 337 [$(M+H)^+$]; HRMS (method D): found 337.1915; calculated for $C_{21}H_{25}N_2O_2$ $(M+H)^+$ 337.1916.

4-(4-(Piperidin-4-yl)phenyl)pyridine (26g)

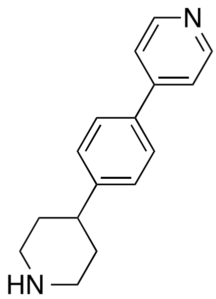

According to General Procedure 2, $Pd(OH)_2$ on carbon (14.2 mg, 0.101 mmol) and *tert*-butyl 4-(4-(pyridin-4-yl)phenyl)-5,6-dihydropyridine-1(2*H*)-carboxylate (85 mg, 0.253 mmol) were reacted together in EtOH (1.5 mL) and hydrochloric acid (1 M, 0.3 mL). The crude material from this reaction was then stirred in THF (2 mL) and hydrochloric acid (1 M, 2 mL) and purified by passing through an SCX-2 cartridge eluting with 1 M NH_3 in $MeOH/CH_2Cl_2$. The ammoniacal solution was concentrated *in vacuo* to yield the crude product as a pale yellow solid that was used in the next step without further purification; ^1H NMR (500 MHz, $CDCl_3$) 1.68 (qd, J = 12.5, 3.9 Hz, 2H), 1.87 (br d, J = 13.0 Hz, 2H), 2.03 (br s, 1H), 2.69 (tt, J = 12.2, 3.5 Hz, 1H), 2.77 (td, J = 12.2, 2.2 Hz, 2H), 3.22 (br d, J = 12.0 Hz, 2H), 7.35 (d, J = 8.2 Hz, 2H), 7.47-7.51 (m, 2H), 7.57-7.61 (m, 2H), 8.62-8.65 (m, 2H).

8-(4-(2-(4-(4-(Pyridin-4-yl)phenyl)piperidin-1-yl)ethyl)-1*H*-pyrazol-1-yl)-3-((2-(trimethylsilyl)ethoxy)methyl)pyrido[3,4-*d*]pyrimidin-4(3*H*)-one (13g)

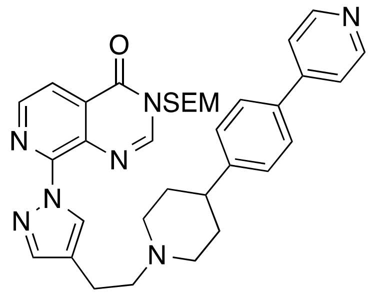

According to General Procedure 3, triethylamine (0.03 mL, 0.190 mmol), 2-(1-(4-oxo-3-((2-(trimethylsilyl)ethoxy)methyl)-3,4-dihydropyrido[3,4-*d*]pyrimidin-8-yl)-1*H*-pyrazol-4-yl)-ethyl methanesulfonate (44.2 mg, 0.095 mmol) and 4-(4-(piperidin-4-yl)phenyl)pyridine (34

mg, 0.143 mmol) were reacted together in anhydrous DMF (1 mL). Purification on a KP-Sil snap cartridge (10% [0.2 M NH_3 in MeOH] in CH_2Cl_2) gave the product as a pale yellow oil (25.4 mg, 44%); ^{1}H NMR (500 MHz, $CDCl_3$) 0.01 (s, 9H), 0.95-1.01 (m, 2H), 1.85-1.97 (m, 4H), 2.21 (td, *J* = 11.0, 3.4 Hz, 2H), 2.62 (tt, *J* = 11.0, 4.4 Hz, 1H), 2.70-2.76 (m, 2H), 2.83-2.89 (m, 2H), 3.20 (br d, *J* = 11.1 Hz, 2H), 3.67-3.73 (m, 2H), 5.47 (s, 2H), 7.36-7.40 (m, 2H), 7.49-7.52 (m, 2H), 7.59-7.62 (m, 2H), 7.82 (s, 1H), 8.07 (d, *J* = 5.1 Hz, 1H), 8.31 (s, 1H), 8.58 (s, 1H), 8.63-8.66 (m, 3H); LC - MS (method C; ESI, *m/z*) t_R = 1.03 min – 478 [(M−SEM+2H)$^+$]; HRMS (method D): found 478.2350; calculated for $C_{28}H_{28}N_7O$ (M−SEM+2H)$^+$ 478.2355.

8-(4-(2-(4-(4-(Pyridin-4-yl)phenyl)piperidin-1-yl)ethyl)-1*H*-pyrazol-1-yl)pyrido[3,4-*d*]pyrimidin-4(3*H*)-one (17g)

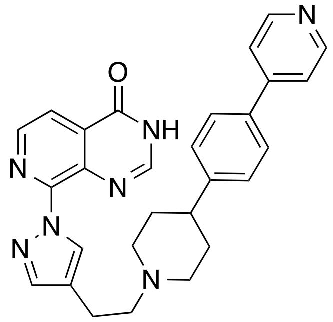

According to General Procedure 4, 8-(4-(2-(4-(4-(pyridin-4-yl)phenyl)piperidin-1-yl)ethyl)-1*H*-pyrazol-1-yl)-3-((2-(trimethylsilyl)ethoxy)methyl)pyrido[3,4-*d*]pyrimidin-4(3*H*)-one (24.7 mg, 0.041 mmol) and hydrochloric acid (6 M, 0.5 mL) were reacted together in THF (0.5 mL). Purification on a KP-NH snap cartridge (40% EtOH in CH_2Cl_2) gave the title compound as a white solid (7.8 mg, 40%); ^{1}H NMR (500 MHz, DMSO-d_6) 1.72 (qd, *J* = 12.3, 3.1 Hz, 2H), 1.77-1.84 (m, 2H), 2.12 (t, *J* = 11.4 Hz, 2H), 2.55-2.65 (m, 3H), 2.69-2.76 (m, 2H), 3.10 (br d, *J* = 11.4 Hz, 2H), 7.41 (d, *J* = 8.2 Hz, 2H), 7.67-7.70 (m, 2H), 7.72-7.76 (m, 3H), 7.99 (d, *J* = 5.1 Hz, 1H), 8.29 (s, 1H), 8.44 (s, 1H), 8.56 (d, *J* = 5.1 Hz, 1H), 8.59-8.63 (m, 2H), 12.74 (br s, 1H); LC - MS (method C; ESI, *m/z*) t_R = 0.60 min – 478 [(M+H)$^+$]; HRMS (method D): found 478.2373; calculated for $C_{28}H_{28}N_7O$ (M+H)$^+$ 478.2355.

1-(3-Bromo-5-(trifluoromethyl)benzyl)pyrrolidine

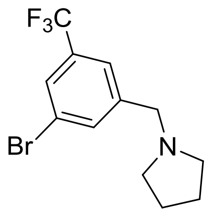

A solution of 3-bromo-5-(trifluoromethyl)benzaldehyde (300 mg, 1.186 mmol) in dry CH_2Cl_2 (10 mL) and pyrrolodine (84 mg, 1.186 mmol) was stirred at room temperature for 10 min, then sodium triacetoxyborohydride (253 mg, 1.20 mmol) was added. Stirring was continued at room temperature for 1 h, the reaction mixture was then washed with saturated aqueous sodium bicarbonate, dried and concentrated in vacuo. The crude product was purified by passing through an isolute SCX-2 cartridge to give a colorless oil (310 mg, 85%). ^{1}H NMR (500 MHz, $CDCl_3$) 1.80-1.84 (m, 4H), 2.46 – 2.58 (m, 4H), 3.65 (s, 2H), 7.54 (br s, 1H), 7.65 (br s, 1H), 7.71 (br s, 1H); LC - MS (method C; ESI, *m/z*) t_R = 0.86 min – 308, 310 [$(M+H)^+$, Br isotopic pattern].

***tert*-Butyl 4-(3-(pyrrolidin-1-ylmethyl)-5-(trifluoromethyl)phenyl)-5,6-dihydropyridine-1(2*H*)-carboxylate (24b)**

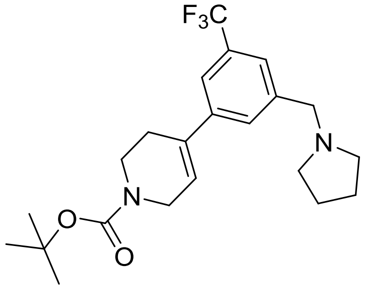

According to General Procedure 1, *tert*-butyl 4-(4,4,5,5-tetramethyl-1,3,2-dioxaborolan-2-yl)-5,6-dihydropyridine-1(2*H*)-carboxylate (371 mg, 1.20 mmol), 1-(3-bromo-5-(trifluoromethyl)benzyl)pyrrolidine (310 mg, 1.01 mmol) and Pd(dppf)Cl_2.CH_2Cl_2 (30 mg, 0.04 mmol) were reacted together in DME (3 mL) and aqueous sodium carbonate (1 M, 2

mL). Purification on a silica column eluting with 3% [7 M NH_3 in MeOH] in CH_2Cl_2 gave the product as a colorless oil (315 mg, 64%). ^{1}H NMR (500 MHz, $CDCl_3$) 1.49 (s, 9H), 1.80-1.82 (m, 4H), 2.20 (br s, 2H), 2.50-2.57 (m, 6H), 3.62-3.71 (m, 2H), 4.08 (br s, 2H), 6.05 (br s, 1H), 7.48 (br s, 2H), 7.56 (br s, 1H); LC - MS (method C; ESI, *m/z*) t_R = 1.17 min – 411 $(M+H)^+$.

4-(3-(Pyrrolidin-1-ylmethyl)-5-(trifluoromethyl)phenyl)piperidine (27b)

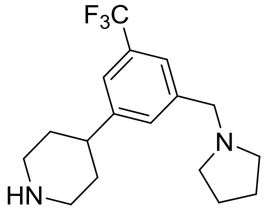

A solution of *tert*-butyl 4-(3-(pyrrolidin-1-ylmethyl)-5-(trifluoromethyl)phenyl)-5,6-dihydropyridine-1(2*H*)-carboxylate (315 mg, 0.76 mmol) in ethanol (10 mL) was stirred in the presence of 10% palladium on charcoal under an atmosphere of hydrogen for 2 h. The reaction mixture was filtered through a pad of celite and the crude residue treated with 4M HCl in dioxane (5 mL) for 2 h. Volatiles were removed and the crude filtered through an isolute SCX-2 cartridge to give the title compound as a colorless oil (122 mg, 51%); ^{1}H NMR (500 MHz, $CDCl_3$) 1.67 (qd, J = 12.5, 4.0 Hz, 2H), 1.74 – 1.92 (m, 6H), 2.48-2.53 (m, 4H), 2.62-2.80 (m, 3H), 3.17-3.24 (m, 2H), 3.64 (s, 2H), 7.35 (br s, 1H), 7.39 (br s, 1H), 7.43 (br s, 1H); LC - MS (method C; ESI, *m/z*) t_R = 0.34 min – 313 $(M+H)^+$.

8-(4-(2-(4-(3-(Pyrrolidin-1-ylmethyl)-5-(trifluoromethyl)phenyl)piperidin-1-yl)ethyl)-1*H*-pyrazol-1-yl)-3-((2-(trimethylsilyl)ethoxy)methyl)pyrido[3,4-*d*]pyrimidin-4(3*H*)-one (14b)

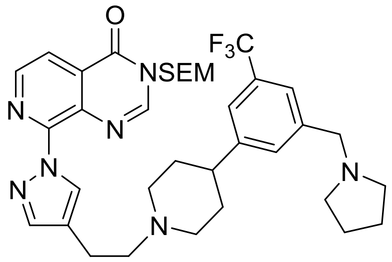

According to General Procedure 5, 2-(1-(4-oxo-3-((2-(trimethylsilyl)ethoxy)methyl)-3,4-dihydropyrido[3,4-*d*]pyrimidin-8-yl)-1*H*-pyrazol-4-yl)acetaldehyde (65 mg, 0.169 mmol), 4-(3-(pyrrolidin-1-ylmethyl)-5-(trifluoromethyl)phenyl)piperidine (35.7 mg, 0.169 mmol) and sodium triacetoxyborohydride (42 mg, 0.20 mmol) were reacted together in dichloromethane (4 mL). Following workup procedure A, the title compound was obtained (33 mg, 29%) as a colorless oil; ^{1}H NMR (500 MHz, $CDCl_3$) 0.02 (s, 9H), 0.94-1.05 (m, 2H), 1.78-1.94 (m, 8H), 2.15-2.20 (m, 2H), 2.51-2.54 (m, 4H), 2.55-2.61 (m, 1H), 2.68-2.74 (m, 2H), 2.80-2.87 (m, 2H), 3.14-3.21 (m, 2H), 3.66 (s, 2H), 3.67-3.73 (m, 2H), 5.47 (s, 2H), 7.38 (s, 1H), 7.43 (br s, 2H), 7.82 (s, 1H), 8.08 (d, J = 5.1 Hz, 1H), 8.32 (s, 1H), 8.57 (s, 1H), 8.65 (d, J = 5.1 Hz, 1H); LC - MS (method C; ESI, *m/z*) t_R = 1.03 min – 552 (M-SEM)$^+$.

8-(4-(2-(4-(3-(Pyrrolidin-1-ylmethyl)-5-(trifluoromethyl)phenyl)piperidin-1-yl)ethyl)-1*H*-pyrazol-1-yl)pyrido[3,4-*d*]pyrimidin-4(3*H*)-one (18b)

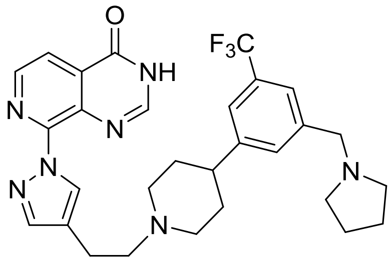

According to General Procedure 4, 8-(4-(2-(4-(3-(pyrrolidin-1-ylmethyl)-5-(trifluoromethyl)phenyl)piperidin-1-yl)ethyl)-1*H*-pyrazol-1-yl)-3-((2-(trimethylsilyl)ethoxy)methyl)pyrido[3,4-*d*]pyrimidin-4(3*H*)-one (33 mg, 0.048 mmol) and hydrochloric acid (6 M, 1 mL) were reacted together in THF (1 mL) for 4 h. Purification was achieved by passing the crude product through an SCX cartridge eluting first with methanol

and then 7N ammonia in methanol. Fractions containing the product were combined, concentrated *in vacuo*, and the residue triturated with Et_2O. The brown precipitate was obtained by filtration and dried (18 mg, 67%). ^{1}H NMR (500 MHz, CD_3OD) 1.81-1.98 (m, 8H), 2.35 (td, *J* = 12.0, 2.7 Hz, 2H), 2.63 (m, 4H), 2.72-2.75 (m, 1H), 2.78-2.83 (m, 2H), 2.88-2.92 (m, 2H), 3.22-3.30 (m, 2H), 3.77 (s, 2H), 7.50 (s, 1H), 7.54 (br s, 1H), 7.55 (br s, 1H), 7.82 (s, 1H), 8.06 (d, *J* = 5.1 Hz, 1H), 8.27 (s, 1H), 8.55 (d, *J* = 5.1 Hz, 1H), 8.77 (s, 1H); HRMS (Method D): t_R 1.16 min - found 552.2703; calculated for $C_{29}H_{33}F_3N_7O$ $(M+H)^+$ 552.2698.

2-(3-Bromo-5-(trifluoromethyl)phenyl)ethan-1-ol

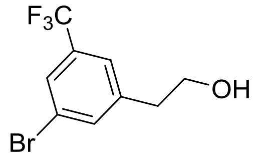

To a solution of 2-(3-bromo-5-(trifluoromethyl)phenyl)acetic acid (1.5 g, 5.3 mmol) in dry THF (5 mL) was added a solution of borane in THF (1 M, 1 M, 11.0 mmol) under nitrogen. The reaction mixture was heated at reflux for 18 h. The reaction was cooled to room temperature and quenched with methanol (2 mL), then concentrated in vacuo and the residue was partitioned between water (20 mL) and ethyl acetate (30 mL). The organic solution was dried and concentrated to give the title compound as colorless oil (1.35 g, 95%); ^{1}H NMR (500 MHz, $CDCl_3$) 2.91 (t, *J* = 6.4 Hz, 2H), 3.90 (t, *J* = 6.4 Hz, 2H), 7.45 (br s, 1H), 7.60 (br s, 1H), 7.65 (br s, 1H).

2-(3-Bromo-5-(trifluoromethyl)phenyl)acetaldehyde

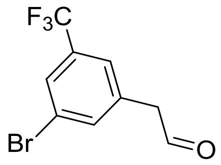

A solution of 2-(3-bromo-5-(trifluoromethyl)phenyl)ethanol (1.35 g, 5 mmol) in dry CH_2Cl_2 (20 mL) was treated with Dess-Martin Periodinane (3.18 g, 7.5 mmol) and the reaction mixture was stirred at room temperature for 1 h, then diluted with dichloromethane (20 mL) and the organic solution was washed with a solution of saturated sodium bicarbonate (10 mL). The organic solution was dried and concentrated in vacuo. The residue was purified on a silica column eluting with 2% ethyl acetate in dichloromethane to give the desired aldehyde as colorless oil (1.05 g, 79%). ^{1}H NMR (500 MHz, $CDCl_3$) 3.79 (d, J = 1.6 Hz, 2H), 7.42 (br s, 1H), 7.57 (br s, 1H), 7.71 (br s, 1H), 9.78 (t, J = 1.6 Hz, 1H).

4-(3-Bromo-5-(trifluoromethyl)phenethyl)morpholine

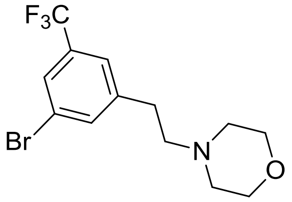

2-(3-Bromo-5-(trifluoromethyl)phenyl)acetaldehyde (1.05 g, 3.93 mmol) was stirred with morpholine (0.342 g, 3.93 mmol) in dry dichloromethane (20 mL) for 5 min. Sodium triacetoxyborohydride (0.84 g, 3.98 mmol) was added and the reaction mixture was stirred for 1 h, then diluted with dichloromethane (20 mL) and the organic solution was washed with saturated sodium hydrogen carbonate solution (10 mL). The organic solution was dried and concentrated in vacuo. The crude product was purified on a silica column eluting with 1% [7N ammonia in methanol] in dichloromethane. The pure fractions afforded the title compound as a pale yellow oil (804 mg, 60%). ^{1}H NMR (500 MHz, $CDCl_3$) 2.52 (br, s, 4H), 2.61 (t, J = 8.3 Hz, 2H), 2.84 (t, J = 8.3 Hz, 2H), 3.74 (t, J = 4.7 Hz, 4H), 7.41(br s, 1H), 7.56 (br s, 1H), 7.62 (br s, 1H); LC - MS (method C; ESI, *m/z*) t_R = 0.93 min – 338, 340 [$(M+H)^+$, Br isotopic pattern].

tert-Butyl-4-(3-(2-morpholinoethyl)-5-(trifluoromethyl)phenyl)-5,6-dihydropyridine-1(2*H*)-carboxylate (24c)

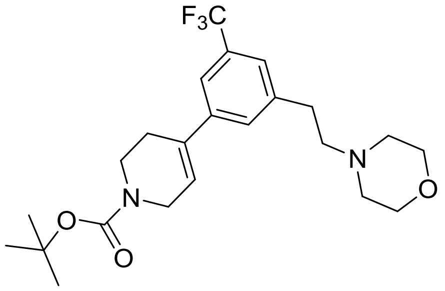

According to General Procedure 1, *tert*-butyl 4-(4,4,5,5-tetramethyl-1,3,2-dioxaborolan-2-yl)-5,6-dihydropyridine-1(2*H*)-carboxylate (450 mg, 1.47 mmol), 4-(3-bromo-5-(trifluoromethyl)phenethyl)morpholine (500 mg, 1.47 mmol) and Pd(dppf)Cl_2.CH_2Cl_2 (120 mg, 0.147 mmol) were reacted together in 1,2-dimethoxyethane (5 mL) and aqueous sodium carbonate (1 M, 3 mL, 3.0 mmol). Purification on a silica column eluting with 3% [7N ammonia in methanol] in dichloromethane gave the product as colorless oil (455 mg, 70%). ^{1}H NMR (500 MHz, $CDCl_3$) 1.50 (s, 9H), 2.52 (br s, 6H), 2.62 (t, J = 8.0 Hz, 2H), 2.88 (t, J = 8.0 Hz, 2H), 3.67 (t, J = 4.8 Hz, 2H), 3.76 (t, J = 4.6 Hz, 4H), 4.10 (br s, 2H), 6.10 (br s, 1H), 7.36 (br s, 1H), 7.39 (br s, 1H), 7.46 (br s, 1H); LC - MS (method_C; ESI, *m/z*) t_R = 1.20 min – 441 $(M+H)^+$.

4-(3-(Piperidin-4-yl)-5-(trifluoromethyl)phenethyl)morpholine (27c)

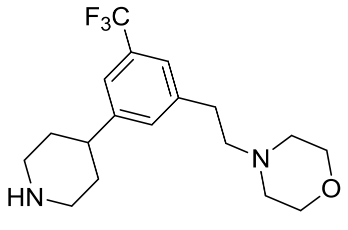

tert-Butyl 4-(3-(2-morpholinoethyl)-5-(trifluoromethyl)phenyl)-5,6-dihydropyridine-1(2*H*)-carboxylate (455 mg, 1.033 mmol) was dissolved in ethanol (20 mL) and stirred in the presence of 10% palladium on carbon under an atmosphere of hydrogen for 2 h. The reaction

mixture was filtered through a pad of celite and the solution was concentrated in vacuo to give the product as colourless oil (430 mg, 94%). This residue was dissolved in dry CH_2Cl_2 (2 mL) and stirred with 4 M HCl in dioxane (10 mL) for 1 h. Volatiles were removed in vacuo, and the residue was passed through an Isolute SCX-2 cartridge followed by purification on silica column chromatography eluting with 10% [7N ammonia in methanol] in dichloromethane to give the title compound as a pale yellow oil (220 mg, 66%). ^{1}H NMR (500 MHz, $CDCl_3$) 1.64 (dq, *J* = 12.5, 4.0 Hz, 2H), 1.82-1.86 (m, 2H), 2.51 (br s, 4H), 2.56-2.62 (m, 2H), 2.67 (tt, *J* = 12.1, 3.7 Hz, 1H), 2.75 (dt, *J* = 12.3, 2.5 Hz, 2H), 2.82-2.87 (m, 2H), 3.21 (dt, *J* = 11.8, 2.7 Hz, 2H), 3.75 (t, *J* = 4.7 Hz, 4H), 7.25 (br s, 1H), 7.31 (br s, 1H), 7.33 (br s, 1H), LC - MS (method C; ESI, *m/z*) t_R = 0.4 min – 343 $(M+H)^+$, HRMS (method D): found 343.2000; calculated for $C_{18}H_{26}F_3N_2O$ $(M+H)^+$ 343.1997.

8-(4-(2-(4-(3-(2-Morpholinoethyl)-5-(trifluoromethyl)phenyl)piperidin-1-yl)ethyl)-1*H*-pyrazol-1-yl)-3-((2-(trimethylsilyl)ethoxy)methyl)pyrido[3,4-*d*]pyrimidin-4(3*H*)-one (14c)

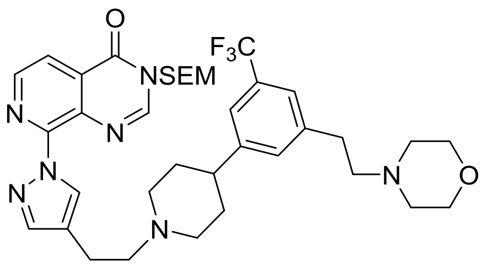

According to General Procedure 5, 2-(1-(4-oxo-3-((2-(trimethylsilyl)ethoxy)methyl)-3,4-dihydropyrido[3,4-*d*]pyrimidin-8-yl)-1*H*-pyrazol-4-yl)acetaldehyde (65 mg, 0.169 mmol), 4-(3-(piperidin-4-yl)-5-(trifluoromethyl)phenethyl)morpholine (57.7 mg, 0.169 mmol) and sodium triacetoxyborohydride (42 mg, 0.20 mmol) were reacted together in dichloromethane (4 mL). Following workup procedure A, the title compound was obtained as a colorless oil (39 mg, 32%). ^{1}H NMR (500 MHz, $CDCl_3$) 0.02 (s, 9H), 0.95-1.00 (m, 2H), 1.88-1.92 (m, 4H), 2.18-2.25 (m, 2H), 2.54 (br s, 4H), 2.58-2.62 (m, 3H), 2.73-2.76 (m, 2H), 2.84-2.89 (m,

4H), 3.21 (d, *J* = 11.7 Hz, 2H), 3.68-3.72 (m, 2H), 3.74-3.82 (m, 4H), 5.47 (s, 2H), 7.31 (br s, 1H), 7.35 (br s, 1H), 7.81 (s, 1H), 8.07 (d, *J* = 5.1 Hz, 1H), 8.32 (s, 1H), 8.58 (s, 1H), 8.63 (d, *J* = 5.1 Hz, 1H); LC - MS (method C; ESI, *m/z*) t_R = 1.02 min – 582 (M-SEM)$^+$.

8-(4-(2-(4-(3-(2-Morpholinoethyl)-5-(trifluoromethyl)phenyl)piperidin-1-yl)ethyl)-1*H*-pyrazol-1-yl)pyrido[3,4-*d*]pyrimidin-4(3*H*)-one (18c)

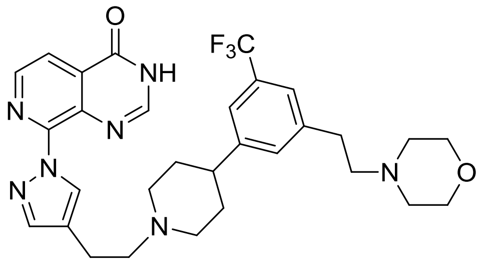

According to General Procedure 4, 8-(4-(2-(4-(3-(2-morpholinoethyl)-5-(trifluoromethyl)phenyl)piperidin-1-yl)ethyl)-1*H*-pyrazol-1-yl)-3-((2-(trimethylsilyl)ethoxy)methyl)pyrido[3,4-*d*]pyrimidin-4(3*H*)-one (39 mg, 0.055 mmol) and hydrochloric acid (6 M, 1 mL) were reacted together in THF (1 mL) for 4 h. Purification was achieved by passing the crude product through an SCX-2 cartridge eluting first with methanol and then 7N ammonia in methanol. Fractions containing the product were combined, concentrated *in vacuo,* and the residue triturated with Et_2O. The brown precipitate was obtained by filtration, and dried (23 mg, 72%). ^{1}H NMR (500 MHz, CD_3OD) 1.82-1.96 (m, 4H), 2.36 (td, *J* = 11.9, 2.8 Hz, 2H), 2.57 (br s, 4H), 2.62-2.67 (m, 2H), 2.72 (tt, *J* = 11.8, 4.0 Hz, 1H), 2.79-2.84 (m, 2H), 2.88-2.94 (m, 4H), 3.25-3.29 (m, 2H), 3.70-3.75 (m, 4H), 7.40 (br s, 2H), 7.43 (br s, 1H), 7.82 (s, 1H), 8.07 (d, *J* = 5.1 Hz, 1H), 8.27 (s, 1H), 8.56 (d, *J* = 5.1 Hz, 1H), 8.78 (s, 1H) ; HRMS (Method D): t_R 1.7 min - found 582.2854; calculated for $C_{30}H_{35}F_3N_7O_2$ (M+H)$^+$ 582.2799. Purity 94%.

***tert*-Butyl 4,7-dimethylspiro[indene-1,4'-piperidine]-1'-carboxylate (29)**

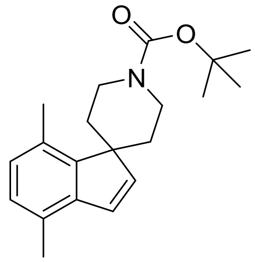

To a stirred, at 0 °C, solution of 4,7-dimethyl-1*H*-indene (250 mg, 1.73 mmol) in dry THF (10 mL), lithium bis(trimethylsilyl)amide (1M solution in THF, 1.7 mL, 1.7 mmol) was added (methodology for the synthesis of spirocyclic indans, see: Chambers M.S. et al. *J. Med. Chem.* **1992**, *35*, 2033-2039). The reaction mixture was allowed to warm to room temperature over a 45 min period, it was then cooled back to 0 °C and was slowly added into a stirred solution of *tert*-butyl bis(2-chloroethyl)carbamate (420 mg, 1.73 mmol) in dry THF at 0 °C. Stirring was continued at this temperature for 2 h, then the mixture was diluted with ethyl acetate (30 mL) and washed with saturated bicarbonate solution, dried, and concentrated in vacuo, The crude product was purified on a silica column eluting with 50% CH_2Cl_2 in hexane to give the product as a colorless oil (276 mg, 51%), ^{1}H NMR (500 MHz, $CDCl_3$) 1.20-1.24 (m, 2H), 1.54 (s, 9H), 2.40 (s, 3H), 2.48 (s, 3H), 2.54 (td, *J* = 13.3, 4.9 Hz, 2H), 3.06 (br s, 2H), 4.28 (br s, 2H), 6.87-6.90 (m, 2H), 6.96-6.98 (m, 2H); LC - MS (method C; ESI, *m/z*) t_R = 1.65 min – 336 $(M+Na)^+$.

***tert*-Butyl 4,7-dimethyl-2,3-dihydrospiro[indene-1,4'-piperidine]-1'-carboxylate**

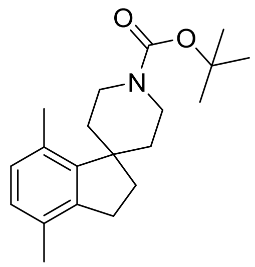

A solution of *tert*-butyl 4,7-dimethylspiro[indene-1,4'-piperidine]-1'-carboxylate (276 mg, 0.881 mmol) in ethanol (10 mL) was stirred in the presence of 10% palladium on charcoal

under an atmosphere of hydrogen for 2 h. The reaction mixture was filtered through a pad of celite, and the filtrate was concentrated in vacuo to give the title compound as a colourless oil (244 mg, 88%), ^{1}H NMR (500 MHz, $CDCl_3$) 1.46-1.50 (m, 2H), 1.51 (s, 9H), 2.11 (t, *J* = 7.5 Hz, 2H), 2.21-2.28 (m, 2H), 2.24 (s, 3H), 2.41 (s, 3H), 2.82 (t, *J* = 7.4 Hz, 2H), 2.95 (td, *J* = 13.2, 2.6 Hz, 2H), 4.10-4.17 (m, 2H), 6.88 (d, *J* = 7.9 Hz, 1H), 6.93 (d, *J* = 7.8 Hz, 1H); LC - MS (method C; ESI, *m/z*) t_R = 1.69 min – 338 $(M+Na)^+$.

4,7-Dimethyl-2,3-dihydrospiro[indene-1,4'-piperidine] (30)

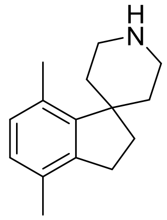

A solution of *tert*-butyl 4,7-dimethyl-2,3-dihydrospiro[indene-1,4'-piperidine]-1'-carboxylate (200 mg, 0.634 mmol) in dry dioxane (3 mL), was treated with 4 M HCl in dioxane (10 mL) for 2 h. It was then evaporated to dryness and the residue was passed through an isolute SCX-2 cartridge to give the product as a colourless oil (130 mg, 95%); ^{1}H NMR (500 MHz, $CDCl_3$) 1.49-1.52 (m, 2H), 2.11 (t, *J* = 7.5 Hz, 2H), 2.24 (s, 3H), 2.20-2.30 (m, 2H), 2.50 (s, 3H), 2.81 (t, *J* = 7.5 Hz, 2H), 2.90 (td, *J* = 1.0, 2.6 Hz, 2H), 3.02-3.12 (m, 2H), 6.89 (d, *J* = 7.8 Hz, 1H), 6.93 (d, *J* = 7.8 Hz, 1H) ; LC - MS (method C; ESI, *m/z*) t_R = 0.88 min – 216 $(M+H)^+$.

8-(4-(2-(4,7-Dimethyl-2,3-dihydrospiro[indene-1,4'-piperidin]-1'-yl)ethyl)-1*H*-pyrazol-1-yl)-3-((2-(trimethylsilyl)ethoxy)methyl)pyrido[3,4-*d*]pyrimidin-4(3*H*)-one

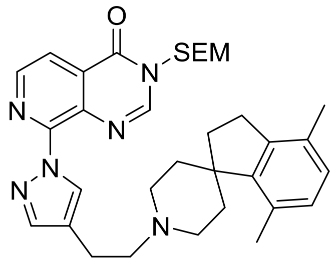

According to General Procedure 3, triethylamine (0.03 mL, 0.237 mmol), 2-(1-(4-oxo-3-((2-(trimethylsilyl)ethoxy)methyl)-3,4-dihydropyrido[3,4-*d*]pyrimidin-8-yl)-1*H*-pyrazol-4-yl)-ethyl methanesulfonate (80 mg, 0.172 mmol) and 4,7-dimethyl-2,3-dihydrospiro[indene-1,4'-piperidine] (44.4 mg, 0.206 mmol) were reacted together in anhydrous DMF (1 mL). Purification on a silica column eluting with 4% [7 M NH_3 in MeOH] in CH_2Cl_2 gave the product as a pale yellow oil (52 mg, 52%); ^{1}H NMR (500 MHz, $CDCl_3$) 0.02 (s, 9H), 0.97-1.01 (m, 2H), 1.55 (d, *J* = 12.8 Hz, 2H), 2.05 (t, *J* = 7.5 Hz, 2H), 2.22 (s, 3H), 2.35–2.45 (m, 2H), 2.49 (s, 3H), 2.52–2.60 (m, 2H), 2.77 (t, *J* = 7.5 Hz, 4H), 2.91 (br s, 2H), 3.10 (br s, 2H), 3.69-3.73 (m, 2H), 5.47 (s, 2H), 6.87 (d, *J* = 7.7 Hz, 1H), 6.91 (d, *J* = 7.7 Hz, 1H), 7.83 (s, 1H), 8.07 (d, *J* = 5.1 Hz, 1H), 8.32 (s, 1H), 8.63 (br s, 2H); LC - MS (method C; ESI, *m/z*) t_R = 1.29 min – 585 $(M+H)^+$.

8-(4-(2-(4,7-Dimethyl-2,3-dihydrospiro[indene-1,4'-piperidin]-1'-yl)ethyl)-1*H*-pyrazol-1-yl)pyrido[3,4-*d*]pyrimidin-4(3*H*)-one (19b)

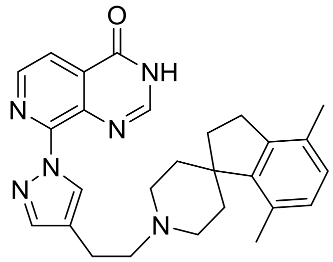

According to General Procedure 4, 8-(4-(2-(4,7-dimethyl-2,3-dihydrospiro[indene-1,4'-piperidin]-1'-yl)ethyl)-1*H*-pyrazol-1-yl)-3-((2-(trimethylsilyl)ethoxy)methyl)pyrido[3,4-*d*]pyrimidin-4(3*H*)-one (52 mg, 0.089 mmol) and hydrochloric acid (6 M, 1 mL) were reacted together in THF (1 mL) for 4 h. Purification was achieved by passing the crude product through an SCX-2 cartridge eluting first with methanol and then 7N ammonia in methanol. Fractions containing the product were combined, concentrated *in vacuo,* and the residue triturated with Et_2O. The white precipitate was obtained by filtration, and dried (36 mg, 89%). ^{1}H NMR (500 MHz, CD_3OD): 1.74-1.83 (m, 2H), 2.18-2.20 (m, 2H), 2.21 (s, 3H),

2.45 (s, 3H), 2.64 (td, *J* = 14.1, 4.2 Hz, 2H), 2.85 (t, *J* =7.4 Hz, 2H), 3.14-3.22 (m, 4H), 3.39-3.44 (m, 2H), 3.60-3.66 (m, 2H), 6.85 (d, *J* = 7.6 Hz, 1H), 6.91 (d, *J* = 7.6 Hz, 1H), 7.89 (s, 1H), 8.09 (d, *J* = 5.1 Hz, 1H), 8.24 (s, 1H), 8.58 (d, *J* = 5.1 Hz, 1H), 8.84 (s, 1H); ^{13}C NMR (150 MHz, DMSO-d_6) 18.9, 19.2, 19.8, 28.3, 31.3, 34.3, 47.3, 49.9, 56.4, 117.9, 118.8, 128.3, 130.3, 131.0, 131.4, 132.1, 136.8, 141.7, 142.7, 144.6, 145.3, 147.8, 148.4, 159.8; HRMS: t_R = 2.04 min - found: 455.2534; calculated for $C_{27}H_{31}N_6O$ $(M+H)^+$ 455.2559.

8-(4-(2-(3*H*-Spiro[isobenzofuran-1,4'-piperidin]-1'-yl)ethyl)-1*H*-pyrazol-1-yl)-3-((2-(trimethylsilyl)ethoxy)methyl)pyrido[3,4-*d*]pyrimidin-4(3*H*)-one

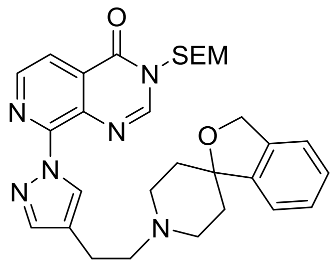

According to General Procedure 3, triethylamine (20.62 mg, 0.206 mmol), 2-(1-(4-oxo-3-((2-(trimethylsilyl)ethoxy)methyl)-3,4-dihydropyrido[3,4-*d*]pyrimidin-8-yl)-1*H*-pyrazol-4-yl)-ethyl methanesulfonate (80 mg, 0.172 mmol) and 3*H*-spiro[isobenzofuran-1,4'-piperidine] (39 mg, 0.206 mmol) were reacted together in anhydrous DMF (1 mL). Purification on a silica column eluting with 3 to 5% [7 M NH_3 in MeOH] in CH_2Cl_2 gave the product as a pale yellow oil (60 mg, 62%); ^{1}H NMR (500 MHz, $CDCl_3$) 0.02 (s, 9H), 0.97-1.01 (m, 2H), 1.80-1.87 (m, 2H), 2.06 (br t, *J* = 7.3 Hz, 2H), 2.54 (br t, *J* = 7.3 Hz, 2H), 2.77-2.82 (m, 2H), 2.85-2.92 (m, 2H), 2.96-3.05 (m, 2H), 3.69-3.72 (m, 2H), 5.10 (s, 2H), 5.48 (s, 2H), 7.17-7.24 (m, 2H), 7.28-7.31 (m, 2H) 7.82 (s, 1H), 8.07 (d, *J* = 5.1 Hz, 1H), 8.33 (s, 1H), 8.58 (s, 1H), 8.64 (d, *J* = 5.1 Hz, 1H); LC - MS (method C; ESI, *m/z*) t_R = 1.23 min – 559 $(M+H)^+$.

8-(4-(2-(3*H*-Spiro[isobenzofuran-1,4'-piperidin]-1'-yl)ethyl)-1*H*-pyrazol-1-yl)pyrido[3,4-*d*]pyrimidin-4(3*H*)-one (19c)

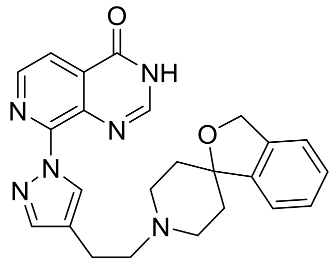

According to General Procedure 4, 8-(4-(2-(3*H*-spiro[isobenzofuran-1,4'-piperidin]-1'-yl)ethyl)-1*H*-pyrazol-1-yl)-3-((2-(trimethylsilyl)ethoxy)methyl)pyrido[3,4-*d*]pyrimidin-4(3*H*)-one (60 mg, 0.107 mmol) and hydrochloric acid (6 M, 3 mL) were reacted together in THF (3 mL) for 4 h. Purification was achieved by passing the crude product through an SCX-2 cartridge eluting first with methanol and then 7 N ammonia in methanol. Fractions containing the product were combined, concentrated *in vacuo*, and the residue triturated with Et_2O. The beige precipitate was obtained by filtration, and dried (39mg, 85%); ^{1}H NMR (500 MHz, DMSO-d_6) 1.61-1.67 (m, 2H), 1.93 (td, J = 13.0, 4.5 Hz, 2H), 2.37-2.41 (m, 2H), 2.61-2.63 (m, 2H), 2.73 (t, J = 7.3 Hz, 2H), 2.88 (d, J = 11.1 Hz, 2H), 4.98 (s, 2H), 7.27 (s, 4H), 7.74 (s, 1H), 7.98 (d, J = 5.1 Hz, 1H), 8.29 (s, 1H), 8.43 (s, 1H), 8.56 (d, J = 5.1 Hz, 1H), 12.57 (br s, 1H); HRMS: t_R = 1.68 min - found: 429.2040; calculated for $C_{24}H_{25}N_6O_2$ $(M+H)^+$ 429.2039.

8-(4-(2-(4-(3-Chlorophenyl)-4-methylpiperidin-1-yl)ethyl)-1*H*-pyrazol-1-yl)-3-((2-(trimethylsilyl)ethoxy)methyl)pyrido[3,4-*d*]pyrimidin-4(3*H*)-one

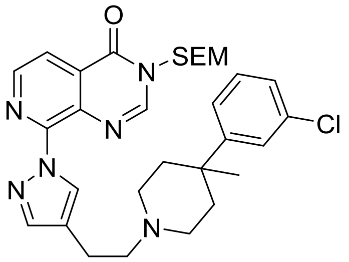

According to General Procedure 5, 2-(1-(4-oxo-3-((2-(trimethylsilyl)ethoxy)methyl)-3,4-dihydropyrido[3,4-*d*]pyrimidin-8-yl)-1*H*-pyrazol-4-yl)acetaldehyde (42 mg, 0.107 mmol), 4-(3-chlorophenyl)-4-methylpiperidine (23 mg, 0.110 mmol) and sodium triacetoxyborohydride (35.7 mg, 0.169 mmol) were reacted together in dichloromethane (4 mL). Following workup procedure A, the title compound was obtained as a colourless oil (35 mg, 56%); ^{1}H NMR (500 MHz, $CDCl_3$) 0.02 (s, 9H), 0.96-1.00 (m, 2H), 1.25 (s, 3H), 1.79-1.88 (m, 2H), 2.12-2.20 (m, 2H), 2.53 (br s, 2H), 2.58-2.69 (m, 4H), 2.80 (t, *J* = 8.5 Hz, 2H), 3.66-3.72 (m, 2H), 5.46 (s, 2H), 7.18 (dt, *J* = 7.4, 1.8 Hz, 1H), 7.22-7.29 (m, 2H), 7.33 (t, *J* = 1.9 Hz, 1H), 7.77 (s, 1H), 8.06 (d, *J* = 5.1 Hz, 1H), 8.28 (s, 1H), 8.56 (s, 1H), 8.62 (d, *J* = 5.1 Hz, 1H); LC - MS (Method C; ESI, *m/z*) t_R = 1.29 min – 579, 581 [$(M+H)^+$] (Cl isotopic pattern).

8-(4-(2-(4-(3-Chlorophenyl)-4-methylpiperidin-1-yl)ethyl)-1*H*-pyrazol-1-yl)pyrido[3,4-*d*]pyrimidin-4(3*H*)-one (19d)

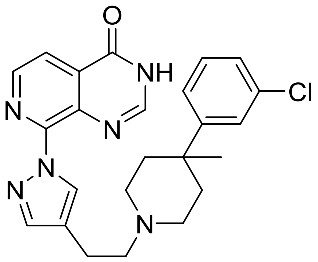

According to General Procedure 4, 8-(4-(2-(4-(3-chlorophenyl)-4-methylpiperidin-1-yl)ethyl)-1*H*-pyrazol-1-yl)-3-((2-(trimethylsilyl)ethoxy)methyl)pyrido[3,4-*d*]pyrimidin-4(3*H*)-one (35 mg, 0.060 mmol) and hydrochloric acid (6 M, 1 mL) were reacted together in THF (1 mL) for 4 h. Purification was achieved by passing the crude product through an SCX-

2 cartridge eluting first with methanol and then 7 N ammonia in methanol. Fractions containing the product were combined, concentrated *in vacuo*, and the residue triturated with Et_2O. The beige precipitate was obtained by filtration, and dried (20 mg, 74%); ^{1}H NMR (500 MHz, CD_3OD) 1.21 (s, 3H), 1.89 (ddd, *J* = 13.4, 7.8, 3.3 Hz, 2H), 2.23 (br s, 2H), 2.63 (br s, 2H), 2.73 – 2.87 (m, 6H), 7.21 (dt, *J* = 7.3, 1.9 Hz, 1H), 7.30-7.37 (m, 2H), 7.41 (t, *J* = 1.9 Hz, 1H), 7.78 (s, 1H), 8.05 (d, *J* = 5.2 Hz, 1H), 8.25 (s, 1H), 8.54 (d, *J* = 5.1 Hz, 1H), 8.73 (s, 1H); HRMS (method D): t_R = 2.10 min - found: 449.1863; calculated for $C_{24}H_{26}ClN_6O$ $(M+H)^+$ 449.1856.

1-Methyl-4-(1*H*-pyrazol-4-yl)-1,2,3,6-tetrahydropyridine (36)

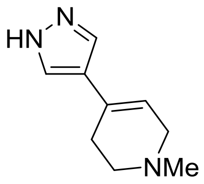

A 2.5 M solution of *n*BuLi (13.1 mL, 32.7 mmol) in hexane was added dropwise to a solution of 4-bromo-1*H*-pyrazole (2.0 g, 13.6 mmol) in tetrahydrofuran (33 mL) at -78 °C. The resulting solution was allowed to warm to room temperature, stirred at room temperature (1.5 h) then cooled to -78 °C. 1-Methylpiperidin-4-one (2.3 mL, 19.1 mmol) was added dropwise and the resulting solution allowed to warm to room temperature and stirred at room temperature (1.5 h). Acetic acid (11.5 mL) was added and the resulting suspension concentrated *in vacuo* to give an orange oil. Trifluoroacetic acid (23 mL) was added and the resulting solution heated at 90 °C for 4 h. The reaction mixture was poured slowly into saturated potassium carbonate solution (100 mL), diluted with water (100 mL) and extracted with dichloromethane (3 × 150 mL). The organic layers were combined, dried over sodium sulfate and concentrated *in vacuo* to give 4 g of an orange oil that was purified by flash column chromatography (gradient from CH_2Cl_2 to 95:5:0.5 CH_2Cl_2:MeOH:NH_4OH) to give

the product as a yellow solid (856 mg, 40%); ^{1}H NMR (400 MHz, $CDCl_3$) 7.58 (2H, s), 5.90–5.97 (1H, m), 3.04–3.11 (2H, m), 2.61–2.69 (2H, m), 2.44–2.52 (2H, m), 2.40 (3H, s); *m/z* (MS, ES^+) 164 ($M+H^+$); LCMS (Method E) t_R 0.41 min (97%, ELSD).

1-Methyl-4-(1*H*-pyrazol-4-yl)piperidine (37)

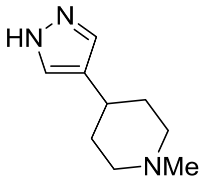

A solution of 1-methyl-4-(1*H*-pyrazol-4-yl)-1,2,3,6-tetrahydropyridine (450 mg, 2.76 mmol) in ethanol (9 mL) was added to 180 mg of Pd (10% on carbon) followed by 5.4 mL of hydrochloric acid (aqueous, 1 M). The resulting mixture was evacuated and refilled with nitrogen (×3) then evacuated and refilled with hydrogen (×3) and stirred under an atmosphere of hydrogen at room temperature for 16 h. The reaction mixture was filtered through Celite® and concentrated *in vacuo*. The residue was taken up in methanol and purified by strong cation exchange chromatography, washing with methanol (100 mL) and extracting with 2 M ammonia in methanol (100 mL) to give the product as a yellow oil (325 mg, 71%); ^{1}H NMR (400 MHz, $CDCl_3$) 7.44 (s, 2H), 2.93-3.01 (m, 2H), 2.48-2.59 (m, 1H), 2.35 (s, 3H), 2.07-2.15 (m, 2H), 1.91-1.98 (m, 2H), 1.67-1.80 (m, 2H); *m/z* (MS, ES^+) 166 $(M+H)^+$; LCMS (Method E) t_R 0.32 min (100%, ELSD).

8-[4-(1-Methylpiperidin-4-yl)-1*H*-pyrazol-1-yl]-3-{[2-(trimethylsilyl)ethoxy]methyl}pyrido[3,4-*d*]pyrimidin-4(3*H*)-one

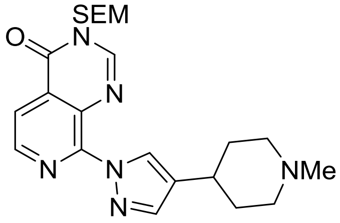

Acetonitrile (1.5 mL) was added to a mixture of 1-methyl-4-(1*H*-pyrazol-4-yl)piperidine (100 mg, 0.60 mmol) and cesium carbonate (394 mg, 1.21 mmol) that had been degassed and refilled with nitrogen × 3 in a sealed vial. The resulting mixture was degassed and refilled with nitrogen × 3 and stirred at room temperature for 1 h. 8-Chloro-3-((2-(trimethylsilyl)ethoxy)methyl)pyrido[3,4-*d*]pyrimidin-4(3*H*)-one (377 mg, 1.21 mmol) was added and the vial resealed and degassed and refilled with nitrogen × 3 then heated at 82 °C for 16 h. The reaction mixture was purified by flash column chromatography (gradient from CH_2Cl_2 to 20% 90:10:1 CH_2Cl_2:MeOH:NH_4OH in CH_2Cl_2) to give the title compound as a yellow gum (97 mg, 36%). Rf 0.70 (90:10:1 CH_2Cl_2:MeOH:NH_4OH); ^{1}H NMR (400 MHz, $CDCl_3$) 8.61 (1H, d, J = 5.0), 8.51 (1H, s), 8.30 (1H, s), 8.05 (1H, d, J = 5.0), 7.78 (1H, s), 5.46 (2H, s), 3.64-3.72 (2H, m), 2.96-3.04 (2H, m), 2.57-2.66 (1H, m), 2.37 (3H, s), 2.11-2.21 (2H, m), 1.99-2.07 (2H, m), 1.79-1.91 (2H, m), 0.93-1.01 (2H, m), 0.00 (9H, s); *m/z* (MS, ES^+) 441 $(M+H)^+$; LCMS (Method E) t_R = 1.41 min (98%, ELSD).

8-[4-(1-Methylpiperidin-4-yl)-1*H*-pyrazol-1-yl]pyrido[3,4-*d*]pyrimidin-4(1*H*)-one (34a)

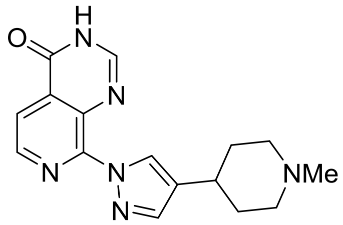

Tetrabutylammonium fluoride (0.34 mL of a 1 M solution in tetrahydrofuran, 0.34 mmol) was added dropwise to a solution of 8-[4-(1-methylpiperidin-4-yl)-1*H*-pyrazol-1-yl]-3-{[2-(trimethylsilyl)ethoxy]methyl}pyrido[3,4-*d*]pyrimidin-4(3*H*)-one (30 mg, 0.068 mmol) in tetrahydrofuran (0.5 mL). The resulting mixture was stirred at room temperature for 72 h then purified by flash column chromatography (gradient from CH_2Cl_2 to 80:20:2 CH_2Cl_2:MeOH:NH_4OH) followed by preparative HPLC to give the product as a white solid (4 mg, 19%); ^{1}H NMR (400 MHz, CD_3OD) 1.85-1.99 (4H, m) 2.19-2.27 (2H, m) 2.72 (3H, s)

2.78-2.95 (3H, m) 7.85 (1H, s) 8.08 (1H, d, J = 5.0) 8.25 (1H, s) 8.57 (1H, d, J = 5.0) 8.74 (1H, s); ^{13}C NMR (100 MHz, CD_3OD) 29.8, 30.8, 43.2, 54.2, 117.8, 126.6, 130.0, 130.9, 139.9, 140.2, 143.8, 146.7, 147.4, 161.0; *m/z* (MS, ES^+) 311 $(M+H)^+$; LCMS (Method E) t_R 0.50 min (94%, ELSD).

1-Isopropyl-4-(1*H*-pyrazol-4-yl)piperidine (32c)

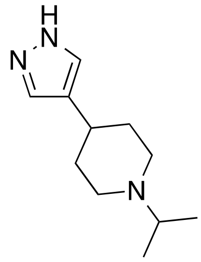

According to General Procedure 7, 4-(1*H*-pyrazol-4-yl)piperidine (81 mg, 0.536 mmol) and acetone (0.1 mL, 1.78 mmol) were reacted together in DMF (5 mL) for 10 min. Sodium triacetoxyborohydride (454.1 mg, 2.14 mmol) was then added. On completion of the reaction, the reaction mixture was concentrated *in vacuo* and the residue redissolved in MeOH/CH_2Cl_2. The crude material was passed through an SCX-2 cartridge eluting with 1 M NH_3 in MeOH/CH_2Cl_2. The ammoniacal solution was concentrated *in vacuo* to yield the crude product that was used in the next step without further purification.

8-(4-(1-Isopropylpiperidin-4-yl)-1*H*-pyrazol-1-yl)-3-((2-(trimethylsilyl)ethoxy)methyl)pyrido[3,4-*d*]pyrimidin-4(3*H*)-one (33c)

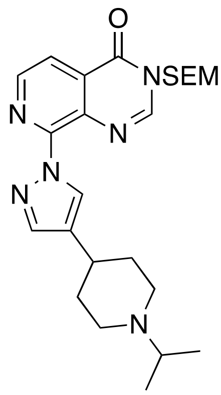

According to General Procedure 8, 8-chloro-3-(2-trimethylsilylethoxymethyl)pyrido[3,4-*d*]pyrimidin-4-one (80 mg, 0.257 mmol), 1-isopropyl-4-(1*H*-pyrazol-4-yl)piperidine (48.3 mg, 0.250 mmol) and cesium carbonate (122.1 mg, 0.375 mmol) were reacted together in anhydrous MeCN (2 mL). Purification on a KP-Sil snap cartridge (15% [0.2 M NH_3 in MeOH] in CH_2Cl_2) gave the product as a brown oil (102.1 mg, 87%); ^{1}H NMR (500 MHz, $CDCl_3$) 0.02 (s, 9H), 0.93-0.98 (m, 2H), 1.10 (d, J = 6.6 Hz, 6H), 1.83 (qd, J = 12.2, 3.3 Hz, 2H), 1.99-2.05 (m, 2H), 2.32 (t, J = 11.5 Hz, 2H), 2.60 (tt, J = 11.5, 3.7 Hz, 1H), 2.82 (septet, J = 6.6 Hz, 1H), 3.01 (br d, J = 11.5 Hz, 2H), 3.64-3.69 (m, 2H), 5.44 (s, 2H), 7.76 (s, 1H), 8.02 (d, J = 5.1 Hz, 1H), 8.27 (s, 1H), 8.51 (s, 1H), 8.59 (d, J = 5.1 Hz, 1H); LC - MS (method C; ESI, *m/z*) t_R = 1.15 min – 469 [(M+H)$^+$]; HRMS (method D): found 469.2736; calculated for $C_{24}H_{37}N_6O_2Si$ (M +H)$^+$ 469.2747.

8-(4-(1-Isopropylpiperidin-4-yl)-1*H*-pyrazol-1-yl)pyrido[3,4-*d*]pyrimidin-4(3*H*)-one (34c)

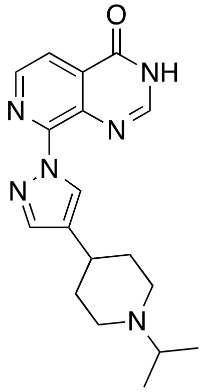

According to General Procedure 4, 8-[4-(1-isopropyl-4-piperidyl)pyrazol-1-yl]-3-(2-trimethylsilylethoxymethyl)pyrido[3,4-*d*]pyrimidin-4-one (76.9 mg, 0.164 mmol) and hydrochloric acid (6 M, 1.5 mL) were reacted together in THF (1.5 mL). Purification on a KP-NH snap cartridge (40% EtOH in CH_2Cl_2) gave the title product as a white solid (43.2 mg, 78%); ^{1}H NMR (500 MHz, DMSO-d_6) 0.99 (d, J = 6.5 Hz, 6H), 1.56 (qd, J = 12.2, 3.3 Hz, 2H), 1.92 (br d, J = 12.6 Hz, 2H), 2.25 (td, J = 11.7, 2.1 Hz, 2H), 2.52 (tt, J = 12.2, 3.9 Hz, 1H), 2.73 (septet, J = 6.5 Hz, 1H), 2.86 (br d, J = 11.4 Hz, 2H), 7.73 (s, 1H), 7.97 (d, J = 5.1 Hz, 1H), 8.29 (s, 1H), 8.37 (s, 1H), 8.53 (d, J = 5.1 Hz, 1H); LC - MS (method C; ESI, *m/z*) t_R = 0.57 min – 339 [(M+H)$^+$]; HRMS (method D): found 339.1923; calculated for $C_{18}H_{23}N_6O$ (M+H)$^+$ 339.1933.

1-Isobutyl-4-(1*H*-pyrazol-4-yl)piperidine (32d)

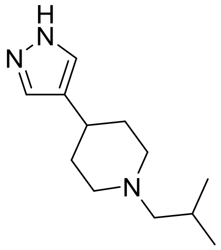

According to General Procedure 7, 4-(1*H*-pyrazol-4-yl)piperidine (80 mg, 0.529 mmol) and isobutyraldehyde (0.19 mL, 2.08 mmol) were reacted together in DMF (5 mL) for 10 min. Sodium triacetoxyborohydride (450 mg, 2.12 mmol) was then added. On completion of the reaction the reaction mixture was concentrated *in vacuo* and the residue redissolved in MeOH/CH_2Cl_2. The crude material was passed through an SCX-2 cartridge eluting with 1 M NH_3 in MeOH/CH_2Cl_2. The ammoniacal solution was concentrated *in vacuo* to yield the product as a white solid (87 mg, 79%); ^{1}H NMR (500 MHz, CD_3OD) 0.94 (d, J = 6.6 Hz, 6H), 1.62-1.73 (m, 2H), 1.82-1.93 (m, 3H), 2.01-2.08 (m,

2H), 2.15 (d, *J* = 7.2 Hz, 2H), 2.49-2.59 (m, 1H), 2.94-2.99 (m, 2H), 7.45 (s, 2H); HRMS (method D): found 208.1821; calculated for $C_{12}H_{22}N_3$ $(M+H)^+$ 208.1810.

8-(4-(1-Isobutylpiperidin-4-yl)-1*H*-pyrazol-1-yl)-3-((2-(trimethylsilyl)ethoxy)methyl)pyrido[3,4-*d*]pyrimidin-4(3*H*)-one (33d)

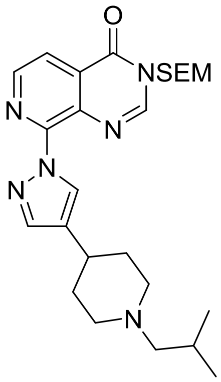

According to General Procedure 8, 8-chloro-3-(2-trimethylsilylethoxymethyl)pyrido[3,4-*d*]pyrimidin-4-one (80 mg, 0.257 mmol), 1-isobutyl-4-(1*H*-pyrazol-4-yl)piperidine (80 mg, 0.386 mmol) and cesium carbonate (125 mg, 0.384 mmol) were reacted together in anhydrous MeCN (1.5 mL). Purification on a KP-Sil snap cartridge (10% [0.2 M NH_3 in MeOH] in CH_2Cl_2) gave the product as a colorless oil (51 mg, 41%); ^{1}H NMR (500 MHz, CD_3OD) -0.01 (s, 9H), 0.90-0.95 (m, 8H), 1.69-1.78 (m, 2H), 1.80-1.88 (m, 1H), 1.94-2.00 (m, 2H), 2.03-2.09 (m, 2H), 2.13-2.16 (m, 2H), 2.54-2.65 (m, 1H), 2.95-3.00 (m, 2H), 3.70-3.75 (m, 2H), 5.45 (s, 2H), 7.75 (s, 1H), 7.99 (d, *J* = 5.1 Hz, 1H), 8.41 (s, 1H), 8.51 (d, *J* = 5.1 Hz, 1H), 8.67 (s, 1H); HRMS (method D): found 483.2886; calculated for $C_{25}H_{39}N_6O_2Si$ $(M +H)^+$ 483.2904.

8-(4-(1-Isobutylpiperidin-4-yl)-1*H*-pyrazol-1-yl)pyrido[3,4-*d*]pyrimidin-4(3*H*)-one (34d)

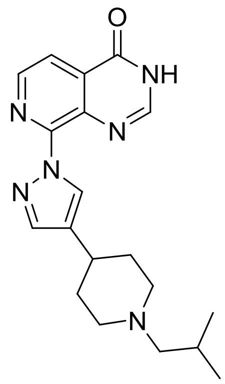

According to General Procedure 4, 8-(4-(1-isobutylpiperidin-4-yl)-1*H*-pyrazol-1-yl)-3-((2-(trimethylsilyl)ethoxy)methyl)pyrido[3,4-*d*]pyrimidin-4(3*H*)-one (51 mg, 0.106 mmol) and hydrochloric acid (6 M, 1.4 mL) were reacted together in THF (1.4 mL). Purification on a KP-NH snap cartridge (40% EtOH in CH_2Cl_2) to give the product as a white solid (18 mg, 48%); ^{1}H NMR (500 MHz, CD_3OD) 0.97 (d, J = 6.6 Hz, 6H), 1.75-1.87 (m, 2H), 1.87-1.98 (m, 1H), 2.05 (d, J = 13.3 Hz, 2H), 2.25 (t, J = 11.7 Hz, 2H), 2.30 (d, J = 7.2 Hz, 2H), 2.66-2.76 (m, 1H), 3.10 (d, J = 11.7 Hz, 2H), 7.79 (s, 1H), 8.04 (d, J = 5.1 Hz, 1H), 8.27 (s, 1H), 8.52 (d, J = 5.1 Hz, 1H), 8.69 (s, 1H); LC - MS (method C; ESI, *m/z*) t_R = 0.67 min – 353 [(M+H)$^+$]; HRMS (method D): found 353.2095; calculated for $C_{19}H_{25}N_6O$ (M+H)$^+$ 353.2090.

1-(Cyclopropylmethyl)-4-(1*H*-pyrazol-4-yl)piperidine (32e)

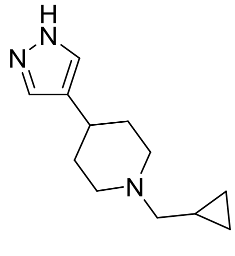

According to General Procedure 7, 4-(1*H*-pyrazol-4-yl)piperidine (80 mg, 0.529 mmol) and cyclopropanecarbaldehyde (0.16 mL, 2.14 mmol) were reacted together in DMF (5 mL) for 10 min. Sodium triacetoxyborohydride (450 mg, 2.12 mmol) was then added. On completion of the reaction, the reaction mixture was concentrated *in vacuo* and the residue redissolved in MeOH/CH_2Cl_2. The crude material was passed through

an SCX-2 cartridge eluting with 1 M NH_3 in MeOH/CH_2Cl_2. The ammoniacal solution was concentrated *in vacuo* to yield the product as a white solid (47 mg, 43%); ^{1}H NMR (500 MHz, CD_3OD) 0.09-0.20 (m, 2H), 0.49-0.60 (m, 2H), 0.85-0.97 (m, 1H), 1.63-1.75 (m, 2H), 1.90-1.99 (m, 2H), 2.10-2.19 (m, 2H), 2.29 (d, *J* = 6.6 Hz, 2H), 2.55 (tt, *J* = 11.7, 3.9 Hz, 1H), 3.10-3.18 (m, 2H), 4.99 (s, 1H), 7.46 (s, 2H); HRMS (method D): found 206.1678; calculated for $C_{12}H_{20}N_3$ $(M+H)^+$ 206.1657.

8-(4-(1-(Cyclopropylmethyl)piperidin-4-yl)-1*H*-pyrazol-1-yl)-3-((2-(trimethylsilyl)ethoxy)methyl)pyrido[3,4-*d*]pyrimidin-4(3*H*)-one (33e)

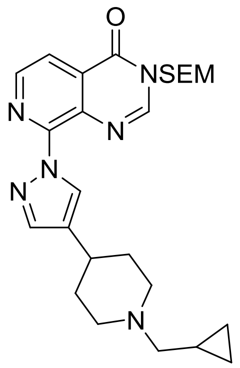

According to General Procedure 8, 8-chloro-3-(2-trimethylsilylethoxymethyl)pyrido[3,4-*d*]pyrimidin-4-one (48 mg, 0.154 mmol), 1-(cyclopropylmethyl)-4-(1*H*-pyrazol-4-yl)piperidine (47 mg, 0.229 mmol) and cesium carbonate (75 mg, 0.230 mmol) were reacted together in anhydrous MeCN (1.5 mL). Purification on a KP-Sil snap cartridge (10% [0.2 M NH_3 in MeOH] in CH_2Cl_2) gave the product as a colorless oil (49 mg, 66%); ^{1}H NMR (500 MHz, CD_3OD) 0.00 (s, 9H), 0.16-0.21 (m, 2H), 0.54-0.60 (m, 2H), 0.92-0.99 (m, 3H), 1.72-1.84 (m, 2H), 1.98-2.10 (m, 2H), 2.21-2.31 (m, 2H), 2.37 (d, *J* = 6.8 Hz, 2H), 2.61-2.72 (m, 1H), 3.22 (d, *J* = 11.5 Hz, 2H), 3.70-3.77 (m, 2H), 5.46 (s, 2H), 7.78 (d, *J* = 0.8 Hz, 1H), 8.02 (d, *J* = 5.1 Hz, 1H), 8.43 (s, 1H), 8.53 (d, *J* = 5.1 Hz, 1H), 8.70 (s, 1H); HRMS (method D): found 481.2739; calculated for $C_{25}H_{37}N_6O_2Si$ $(M+H)^+$ 481.2747.

8-(4-(1-(Cyclopropylmethyl)piperidin-4-yl)-1*H*-pyrazol-1-yl)pyrido[3,4-*d*]pyrimidin-4(3*H*)-one (34e)

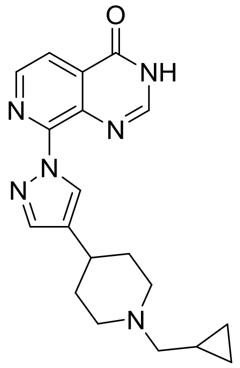

According to General Procedure 4, 8-(4-(1-(cyclopropylmethyl)piperidin-4-yl)-1*H*-pyrazol-1-yl)-3-((2-(trimethylsilyl)ethoxy)methyl)pyrido[3,4-*d*]pyrimidin-4(3*H*)-one (43 mg, 0.090 mmol) and hydrochloric acid (6 M, 1.2 mL) were reacted together in THF (1.2 mL). Purification on a KP-NH snap cartridge (40% EtOH in CH_2Cl_2) to give the product as a white solid (18 mg, 57%); ^{1}H NMR (500 MHz, CD_3OD) 0.20-0.26 (m, 2H), 0.57-0.64 (m, 2H), 0.93-1.02 (m, 1H), 1.78-1.87 (m, 2H), 2.11 (d, J = 13.6 Hz, 2H), 2.36-2.43 (m, 2H), 2.47 (d, J = 6.8 Hz, 2H), 2.72-2.79 (m, 1H), 3.26-3.31 (m, 2H), 7.80 (s, 1H), 8.04 (d, J = 5.2 Hz 1H), 8.27 (s, 1H), 8.51 (d, J = 5.1 Hz, 1H), 8.70 (s, 1H); LC - MS (method C; ESI, *m/z*) t_R = 0.65 min – 351 [(M+H)$^+$]; HRMS (method D): found 351.1941; calculated for $C_{19}H_{23}N_6O$ (M+H)$^+$ 351.1933.

1-(Cyclobutylmethyl)-4-(1*H*-pyrazol-4-yl)piperidine (32f)

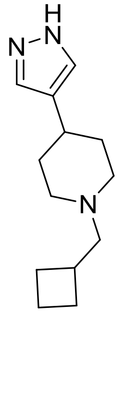

According to General Procedure 7, 4-(1*H*-pyrazol-4-yl)piperidine (108 mg, 0.713 mmol) and cyclobutanecarbaldehyde (60 mg, 0.713 mmol) were reacted together in DMF (3 mL) for 10 min. Sodium triacetoxyborohydride (150 mg, 0.713 mmol) was then added. On completion of the reaction the reaction mixture was passed through an SCX-2 cartridge eluting with 1 M NH_3 in MeOH/CH_2Cl_2. The ammoniacal solution was concentrated *in vacuo* to yield the product (72 mg, 46%); ^{1}H NMR (500 MHz, $CDCl_3$) 1.65-1.72 (m, 4H), 1.75-1.82 (m, 1H), 1.85-1.91 (m, 3H), 2.04-2.12 (m, 4H), 2.46 (d, J = 6.8 Hz, 2H), 2.44-2.53 (m, 1H), 2.55-2.61 (m, 1H), 2.93-2.97 (m, 2H), 7.39 (s, 2H); LC - MS (method C; ESI, *m/z*) t_R = 0.51 min - 220 $(M+H)^+$.

8-(4-(1-(Cyclobutylmethyl)piperidin-4-yl)-1*H*-pyrazol-1-yl)-3-((2-(trimethylsilyl)ethoxy)methyl)pyrido[3,4-*d*]pyrimidin-4(3*H*)-one (33f)

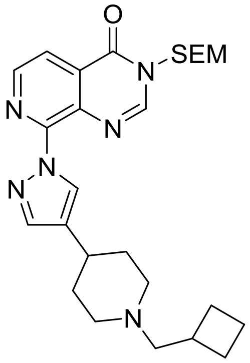

According to General Procedure 8, 8-chloro-3-(2-trimethylsilylethoxymethyl)pyrido[3,4-*d*]pyrimidin-4-one (102 mg, 0.327 mmol), 1-(cyclobutylmethyl)-4-(1*H*-pyrazol-4-yl)piperidine (72 mg, 0.328 mmol) and cesium carbonate (213 mg, 0.656 mmol) were reacted together in anhydrous MeCN (2 mL). Purification on a silica column chromatography eluting with 5% [7 M NH_3 in MeOH] in CH_2Cl_2 gave the product as a brown oil (75 mg, 46%); ^{1}H NMR (500 MHz, $CDCl_3$) 0.02 (s, 9H), 0.94-0.98 (m, 2H), 1.68-1.85 (m, 3H), 1.87-1.99 (m, 3H), 1.99-2.05 (m, 2H), 2.08-2.14 (m, 2H), 2.28 (br t, J = 11.5 Hz, 2H), 2.55-2.72 (m, 4H), 3.09 (br d, J = 11.5 Hz, 2H), 3.67-3.69 (m, 2H), 5.45 (s, 2H), 7.76 (s, 1H), 8.03 (d, J = 5.1

Hz, 1H), 8.29 (s, 1H), 8.53 (s, 1H), 8.59 (d, *J* = 5.1 Hz, 1H); LC - MS (method C; ESI, *m/z*) t_R = 1.15 min – 495 $(M+H)^+$.

8-(4-(1-(Cyclobutylmethyl)piperidin-4-yl)-1*H*-pyrazol-1-yl)pyrido[3,4-*d*]pyrimidin-4(3*H*)-one (34f)

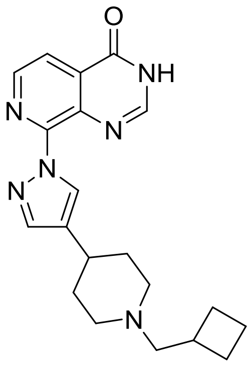

According to General Procedure 4, 8-[4-(1-isopropyl-4-piperidyl)pyrazol-1-yl]-3-(2-trimethylsilylethoxymethyl)pyrido[3,4-*d*]pyrimidin-4-one (75 mg, 0.152 mmol) and hydrochloric acid (6 M, 1.5 mL) were reacted together in THF (1.5 mL). Purification on silica column chromatography eluting with 10% [7 M NH_3 in MeOH] in CH_2Cl_2 gave the product as a white powder (35 mg, 63%); ^{1}H NMR (500 MHz, CD_3OD) 1.76-1.88 (m, 5H), 1.95-2.01 (m, 1H), 2.06-2.12 (m, 2H), 2.14-2.18 (m, 2H), 2.40 (td, *J* = 12.1, 2.7 Hz, 2H), 2.63-2.79 (m, 4H), 3.09-3.15 (m, 2H), 7.80 (s, 1H), 8.05 (d, *J* = 5.1 Hz, 1H), 8.27 (s, 1H), 8.52 (d, *J* = 5.1 Hz, 1H), 8.70 (s, 1H); HRMS (method D): t_R 1.32 min - found 365.2089; calculated for $C_{20}H_{25}N_6O$ $(M+H)^+$ 365.2090.

1-Cyclopentyl-4-(1*H*-pyrazol-4-yl)piperidine (32g)

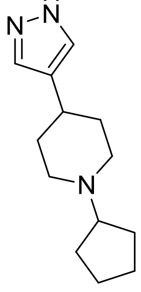

According to General Procedure 7, 4-(1*H*-pyrazol-4-yl)piperidine (55 mg, 0.364 mmol) and cyclopentanone (34 mg, 0.404 mmol) were reacted together in DMF (3 mL) for 10 min. Sodium triacetoxyborohydride (76 mg, 0.364 mmol) was then added. On completion of the reaction the reaction mixture was passed through an isolute SCX-2 cartridge eluting with 1 M NH_3 in MeOH/CH_2Cl_2. The ammoniacal solution was concentrated *in vacuo* to yield the product (70 mg, 88%); ^{1}H NMR (500 MHz, $CDCl_3$) 1.38-1.48 (m, 2H), 1.55-1.78 (m, 6H), 1.91-1.97 (m, 4H), 2.14 (td, J = 11.8, 2.4 Hz, 2H), 2.51 -2.61 (m, 2H), 3.11 (d, J = 11.5 Hz, 2H), 7.46 (s, 2H); LC - MS (method C; ESI, *m/z*) t_R = 0.59 min – 220 $(M+H)^+$.

8-(4-(1-Cyclopentylpiperidin-4-yl)-1*H*-pyrazol-1-yl)-3-((2-(trimethylsilyl)ethoxy)methyl)pyrido[3,4-*d*]pyrimidin-4(3*H*)-one (33g)

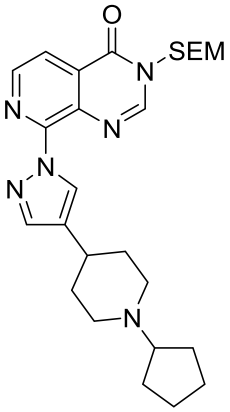

According to General Procedure 8, 8-chloro-3-(2-trimethylsilylethoxymethyl)pyrido[3,4-*d*]pyrimidin-4-one (87 mg, 0.279 mmol) , 1-cyclopentyl-4-(1*H*-pyrazol-4-yl)piperidine (68 mg, 0.310 mmol) and cesium carbonate (202 mg, 0.620 mmol) were reacted together in anhydrous MeCN (2 mL). Purification on a silica column chromatography eluting with 5% [7 M NH_3 in MeOH] in CH_2Cl_2 gave the product as a brown oil (75 mg, 49%); ^{1}H NMR (500 MHz, $CDCl_3$) 0.02 (s, 9H), 0.95-0.98 (m, 2H), 1.50-1.65 (m, 4H), 1.70-1.78 (m, 2H), 1.86-1.98 (m, 4H), 2.01-2.07 (m, 2H), 2.16-2.30 (m, 2H), 2.61-2.71 (m, 2H), 3.23 (br d, J = 11.6

Hz, 2H), 3.68-3.70 (m, 2H), 5.45 (s, 2H), 7.77 (s, 1H), 8.03 (d, *J* = 5.1 Hz, 1H), 8.29 (s, 1H), 8.53 (s, 1H), 8.59 (d, *J* = 5.1 Hz, 1H); LC - MS (method C; ESI, *m/z*) t_R = 1.21 min – 495 $(M+H)^+$.

8-(4-(1-Cyclopentylpiperidin-4-yl)-1*H*-pyrazol-1-yl)pyrido[3,4-*d*]pyrimidin-4(3*H*)-one (34g)

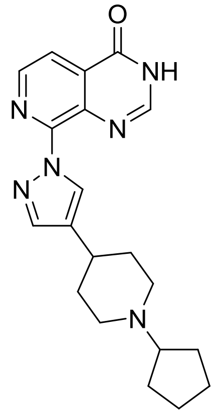

According to General Procedure 4, 8-(4-(1-cyclopentylpiperidin-4-yl)-1*H*-pyrazol-1-yl)-3-((2-(trimethylsilyl)ethoxy)methyl)pyrido[3,4-*d*]pyrimidin-4(3*H*)-one (75 mg, 0.152 mmol) and hydrochloric acid (6 M, 1.5 mL) were reacted together in THF (1.5 mL). Purification on silica column chromatography eluting with 10% [7 M NH_3 in MeOH] in CH_2Cl_2 followed by trituration with ether gave the product as a white powder (33 mg, 60%); ^{1}H NMR (500 MHz, CD_3OD) 1.50-1.58 (m, 2H), 1.60-1.70 (m, 2H), 1.72-1.87 (m, 5H), 2.00-2.07 (m, 3H), 2.09-2.18 (m, 2H), 2.45-2.51 (m, 2H), 2.75-2.92 (m, 2H), 7.81 (s, 1H), 8.05 (d, *J* = 5.1 Hz, 1H), 8.27 (s, 1H), 8.52 (d, *J* = 5.1 Hz, 1H), 8.70 (s, 1H); HRMS (method D): t_R 1.52 min - found 365.1861; calculated for $C_{20}H_{25}N_6O$ $(M+H)^+$ 365.2090.

1-((1-Methyl-1*H*-pyrazol-4-yl)methyl)-4-(1*H*-pyrazol-4-yl)piperidine (32h)

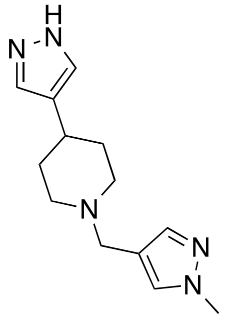

According to General Procedure 7, 4-(1*H*-pyrazol-4-yl)piperidine (74 mg, 0.489 mmol) and 1-methylpyrazole-4-carbaldehyde (53.9 mg, 0.489 mmol) were reacted together in NMP (5 mL) for 6 h. Sodium triacetoxyborohydride (207.4 mg, 0.979 mmol) was then added. On completion of the reaction, the reaction mixture was concentrated *in vacuo* and the residue redissolved in MeOH/CH_2Cl_2. The crude material was passed through an SCX-2 cartridge eluting with 1 M NH_3 in MeOH/CH_2Cl_2. The ammoniacal solution was concentrated *in vacuo* to yield the crude product that was used in the next step without further purification.

8-(4-(1-((1-Methyl-1*H*-pyrazol-4-yl)methyl)piperidin-4-yl)-1*H*-pyrazol-1-yl)-3-((2-(trimethylsilyl)ethoxy)methyl)pyrido[3,4-*d*]pyrimidin-4(3*H*)-one (33h)

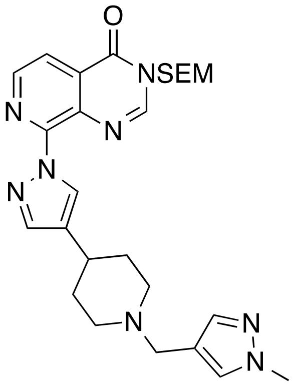

According to General Procedure 8, 8-chloro-3-(2-trimethylsilylethoxymethyl)pyrido[3,4-*d*]pyrimidin-4-one (63.7 mg, 0.204 mmol), 1-[(1-methylpyrazol-4-yl)methyl]-4-(1*H*-pyrazol-4-yl)piperidine (81.9 mg, 0.170 mmol) and cesium carbonate (83.2 mg, 0.255 mmol) were reacted together in anhydrous MeCN (1.5 mL). Purification on a KP-Sil snap cartridge (15%

[0.2 M NH_3 in MeOH] in CH_2Cl_2) gave the product as a pale yellow oil (73 mg, 82%); ^{1}H NMR (500 MHz, $CDCl_3$) −0.02 (s, 9H), 0.93-0.98 (m, 2H), 1.79 (qd, *J* = 12.2, 3.1 Hz, 2H), 1.94-2.02 (m, 2H), 2.12 (br t, *J* = 11.6 Hz, 2H), 2.54-2.64 (m, 1H), 2.98-3.07 (m, 2H), 3.49 (s, 2H), 3.64-3.69 (m, 2H), 3.86 (s, 3H), 5.44 (s, 2H), 7.36 (s, 1H), 7.40 (s, 1H), 7.75 (s, 1H), 8.03 (d, *J* = 5.1 Hz, 1H), 8.27 (s, 1H), 8.49 (s, 1H), 8.58 (d, *J* = 5.1 Hz, 1H); LC - MS (method C; ESI, *m/z*) t_R = 1.07 min – 521 [(M+H)$^+$]; HRMS (method D): found 521.2817; calculated for $C_{26}H_{37}N_8O_2Si$ (M +H)$^+$ 521.2809.

8-(4-(1-((1-Methyl-1*H*-pyrazol-4-yl)methyl)piperidin-4-yl)-1*H*-pyrazol-1-yl)pyrido[3,4-*d*]pyrimidin-4(3*H*)-one (34h)

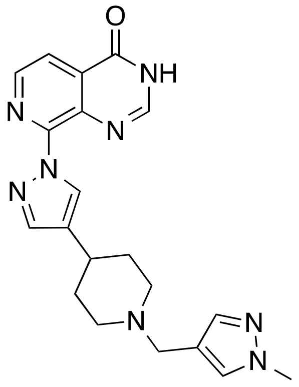

According to General Procedure 4, 8-[4-[1-[(1-methylpyrazol-4-yl)methyl]-4-piperidyl]pyrazol-1-yl]-3-(2-trimethylsilylethoxymethyl)pyrido[3,4-*d*]pyrimidin-4-one (59 mg, 0.113 mmol) and hydrochloric acid (6 M, 1.5 mL) were reacted together in THF (1.5 mL). Purification on a KP-NH snap cartridge (40% EtOH in CH_2Cl_2) gave the product as a white solid (36 mg, 81%); ^{1}H NMR (500 MHz, DMSO-d_6) 1.57 (qd, *J* = 11.7, 4.4 Hz, 2H), 1.86-1.93 (m, 2H), 1.98 (td, *J* = 11.4, 1.9 Hz, 2H), 2.87 (br d, *J* = 11.8 Hz, 2H), 3.78 (s, 3H), 7.29 (s, 1H), 7.56 (s, 1H), 7.71 (s, 1H), 7.95 (d, *J* = 5.3 Hz, 1H), 8.27 (s, 1H), 8.37 (s, 1H), 8.50 (d, *J* = 5.1 Hz, 1H), 12.71 (br s, 1H); LC - MS (method C; ESI, *m/z*) t_R = 0.57 min – 391 [(M+H)$^+$]; HRMS (method D): found 391.1992; calculated for $C_{20}H_{23}N_8O$ (M+H)$^+$ 391.1995.

8-(4-(2-(2,3-Dihydrospiro[indene-1,4'-piperidin]-1'-yl)ethyl)-4,5-dihydro-1*H*-pyrazol-1-yl)-3-((2-(trimethylsilyl)ethoxy)methyl)quinazolin-4(3*H*)-one (40)

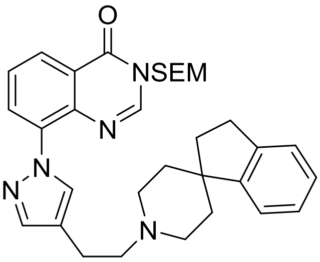

Triethylamine (13 mg, 0.13 mmol) was added to a solution of mesylate **38** (Hatch, S. B. et al, *Epigenetics & Chromatin* **2017**, 10:9, DOI 10.1186/s13072-017-0116-6; 60 mg, 0.13 mmol, freshly made from alcohol precursor) and 2,3-dihydrospiro[indene-1,4'-piperidine] (29 mg, 0.155 mmol) in anhydrous DMF (1 mL, 0.1 M) under N2. The reaction mixture was heated at 50 °C for 15 h and monitored by LCMS. When the reaction had gone to completion, the reaction mixture was diluted in H_2O (5 mL) and extracted three times with EtOAc (3 × 5 mL). The combined organic layers were washed with saturated sodium bicarbonate solution, dried over MgSO4, and concentrated *in vacuo* to give the crude material which was purified by flash silica chromatography eluting with 3% 7 N ammonia in methanol in CH_2Cl_2 yielding the title compound as a yellow oil (33 mg, 45.7%). ^{1}H NMR (500 MHz, $CDCl_3$) 0.02 (s, 9H), 0.97-1.01 (m, 2H), 1.60-1.63 (m, 3H), 2.00-2.07 (m, 3H), 2.27 (t, *J* = 11.7 Hz, 2H), 2.73-2.75 (m, 2H), 2.85-2.94 (m, 4H), 3.02 (br d, *J* = 11.7 Hz, 2H), 3.69-3.73 (m, 2H), 5.48 (s, 2H), 7.18-7.24 (m, 4H), 7.61 (t, *J* = 7.9 Hz, 1H), 7.69 (s, 1H), 8.22 (s, 1H), 8.26 (dd, *J* = 7.9, 1.5 Hz, 1H), 8.30 (dd, *J* = 7.9, 1.5 Hz, 1H), 8.43 (s, 1H); LCMS (method C ; ESI, *m/z*) t_r = 1.29 min – 556 [$(M+H)^+$].

8-(4-(2-(2,3-Dihydrospiro[indene-1,4'-piperidin]-1'-yl)ethyl)-4,5-dihydro-1*H*-pyrazol-1-yl)quinazolin-4(3*H*)-one (41)

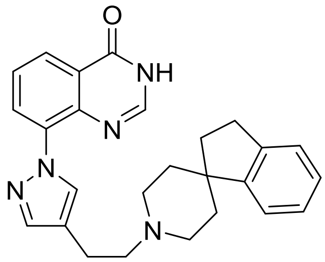

Hydrochloric acid (6 M, 3 mL) was added to a solution of 8-(4-(2-(2,3-dihydrospiro[indene-1,4'-piperidin]-1'-yl)ethyl)-4,5-dihydro-1*H*-pyrazol-1-yl)-3-((2-(trimethylsilyl)ethoxy)methyl)quinazolin-4(3*H*)-one (33 mg, 0.06 mmol) in THF (3 mL). The reaction mixture was stirred at 60 °C for 3 h and monitored by LCMS. Following completion of the reaction, the reaction mixture was concentrated *in vacuo* and filtered through SCX followed by silica gel chromatography eluting with 5% 7N ammonia in methanol in CH_2Cl_2. The pure fractions afforded the title compound as a white powder (7 mg, 28%); ^{1}H NMR (500 MHz, CD_3OD) 1.62 (br d, *J* = 12.1 Hz, 2H), 2.03 (td, *J* = 12.2, 3.4 Hz, 2H), 2.10 (t, *J* = 8.6 Hz, 2H), 2.51(br s, 2H), 2.84-2.94 (m, 6H), 3.16 (br d, *J* = 11.6 Hz, 2H), 7.13 - 7.22 (m, 4H), 7.66 (t, *J* = 7.9 Hz, 1H), 7.72 (s, 1H), 8.12 (dd, *J* = 7.8, 1.3 Hz, 2H), 8.14 (dd, *J* = 8.0, 1.5 Hz, 1H), 8.27 (dd, *J* = 7.9, 1.5 Hz, 1H), 8.43 (s, 1H); HRMS (method D): found 426.2276; calculated for $C_{26}H_{28}N_5O$ $(M+H)^+$ 426.2294.

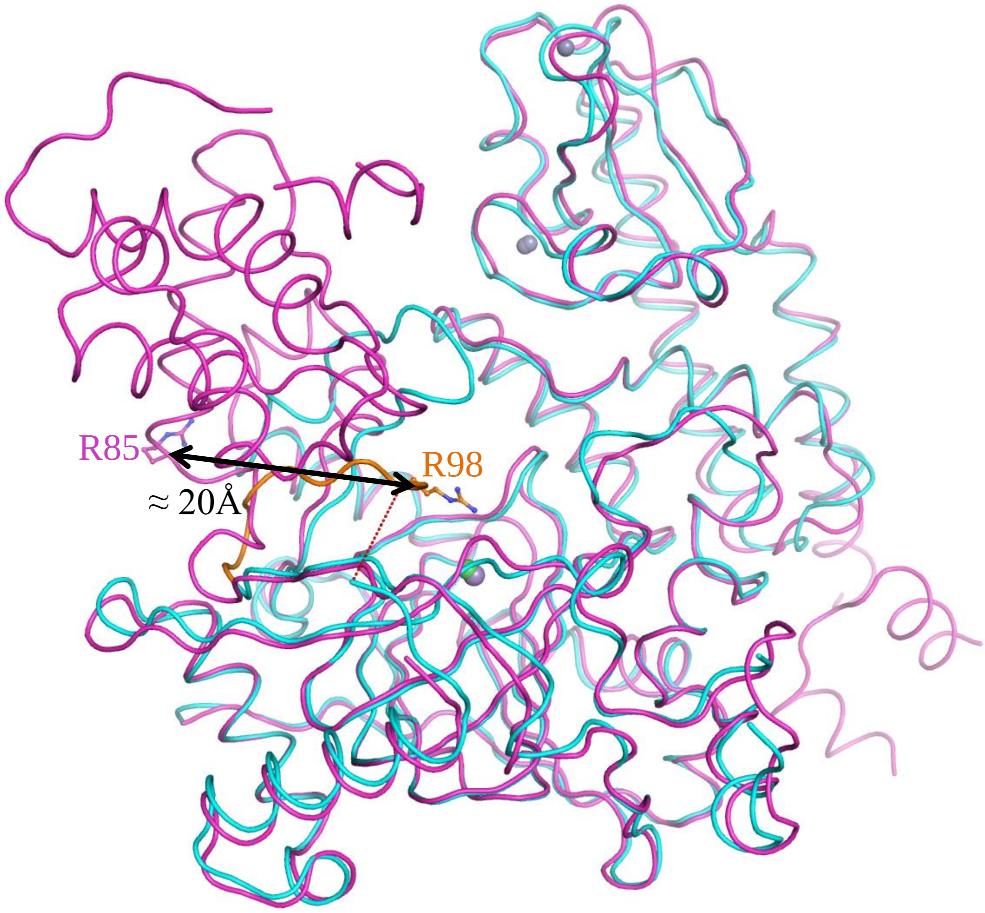

Figure S1: Overlay of structures of KDM5A 12-797 without internal deletion (PDB 5CEH, magenta) and KDM5B 26-772 with internal deletion of residues 102-373 (PDB 5A3T, cyan). We believe that the position of loop 91-100 of KDM5B (depicted in orange) is artefactual due to the deletion of residues 102-373 and their replacement by 4 glycines in this particular KDM5B construct; notably, the corresponding region in KDM5A, which is part of a helix of the ARID domain, adopts a different position with Cα of R85 in KDM5A located about 20 Å away from Cα of the corresponding residue R98 in KDM5B.

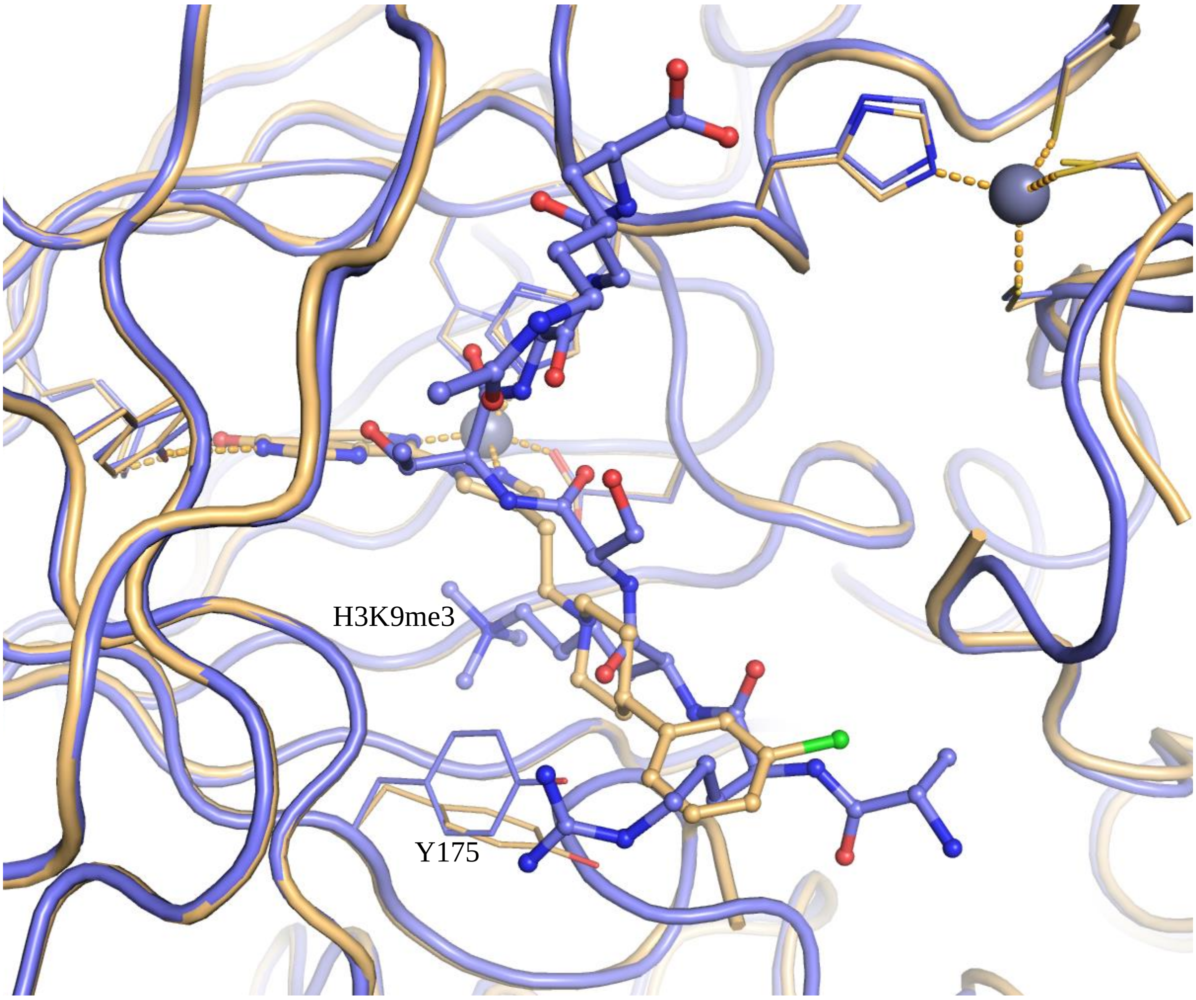

Figure S2: Overlay of crystal structures of **16a** (beige) and H3K9Me3 peptide (PDB 2OQ6, blue) bound to KDM4A. Zn(II) atoms are shown as spheres. Proteins backbone chains are represented as a cartoon tubes, key residues are displayed in line representation. Compound **16a** and H3K9Me3 peptide are shown in ball and stick representation.

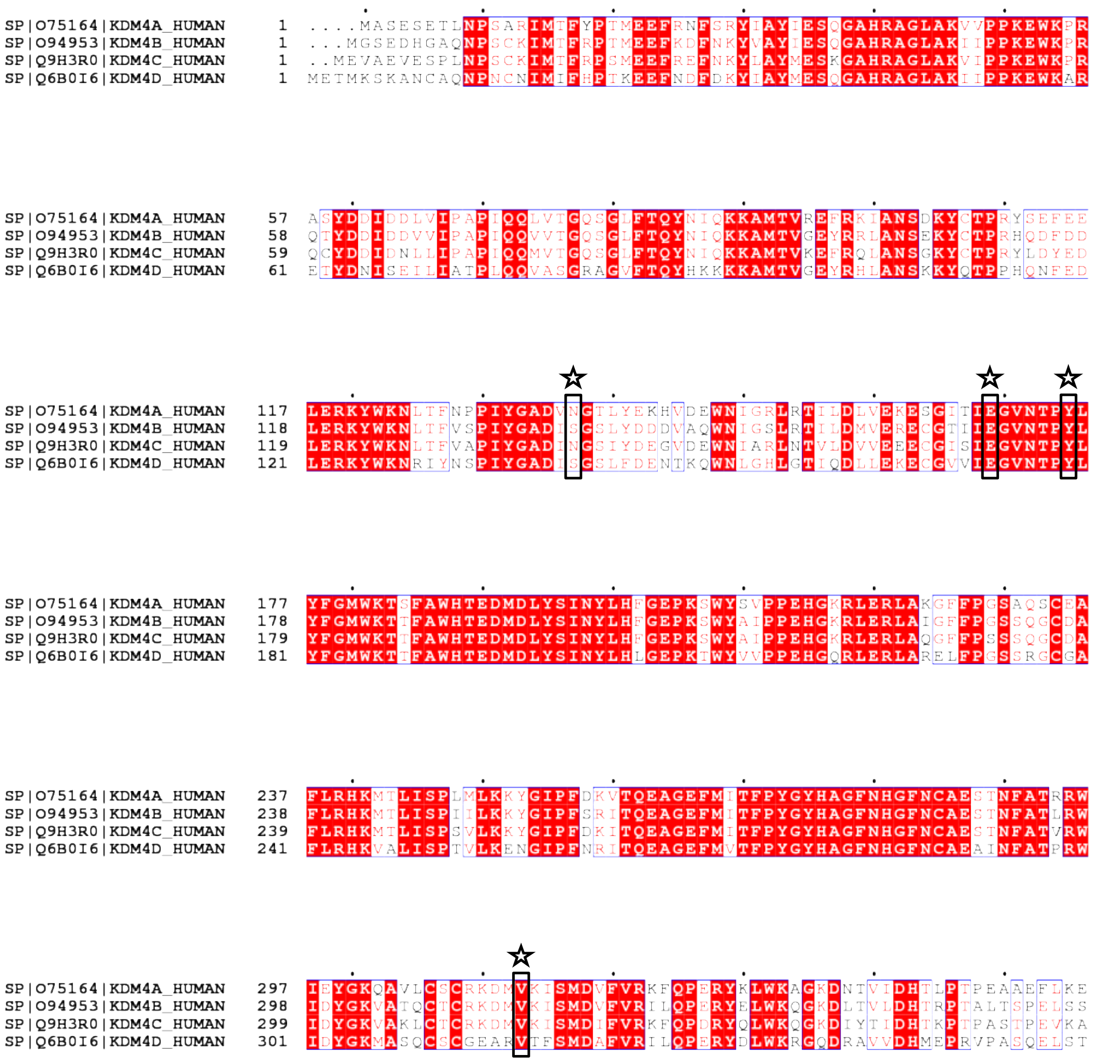

Figure S3: Sequence alignment of KDM4A, B, C and D. Residues targeted to gain potency and/or selectivity are highlighted with a black star and a rectangle. Alignments have been generated with Clustal Omega (Sievers *et al.*, 2011), and the corresponding figure generated with ESPript 3.0 (Robert *et al.*, 2014).

Sievers, F.; Wilm, A.; Dineen, D.G.; Gibson, T.J.; Karplus, K.; Li, W.; Lopez, R.; McWilliam, H.; Remmert, M.; Söding, J.; Thompson, J.D.; Higgins, D.G. Fast, scalable generation of high-quality protein multiple sequence alignments using Clustal Omega. *Mol. Syst. Biol.* **2011**, *7*, 539.

Robert, X.; Gouet, P. Deciphering key features in protein structures with the new ENDscript server. *Nucleic Acids Res.* **2014**, *42*, W320−W324.

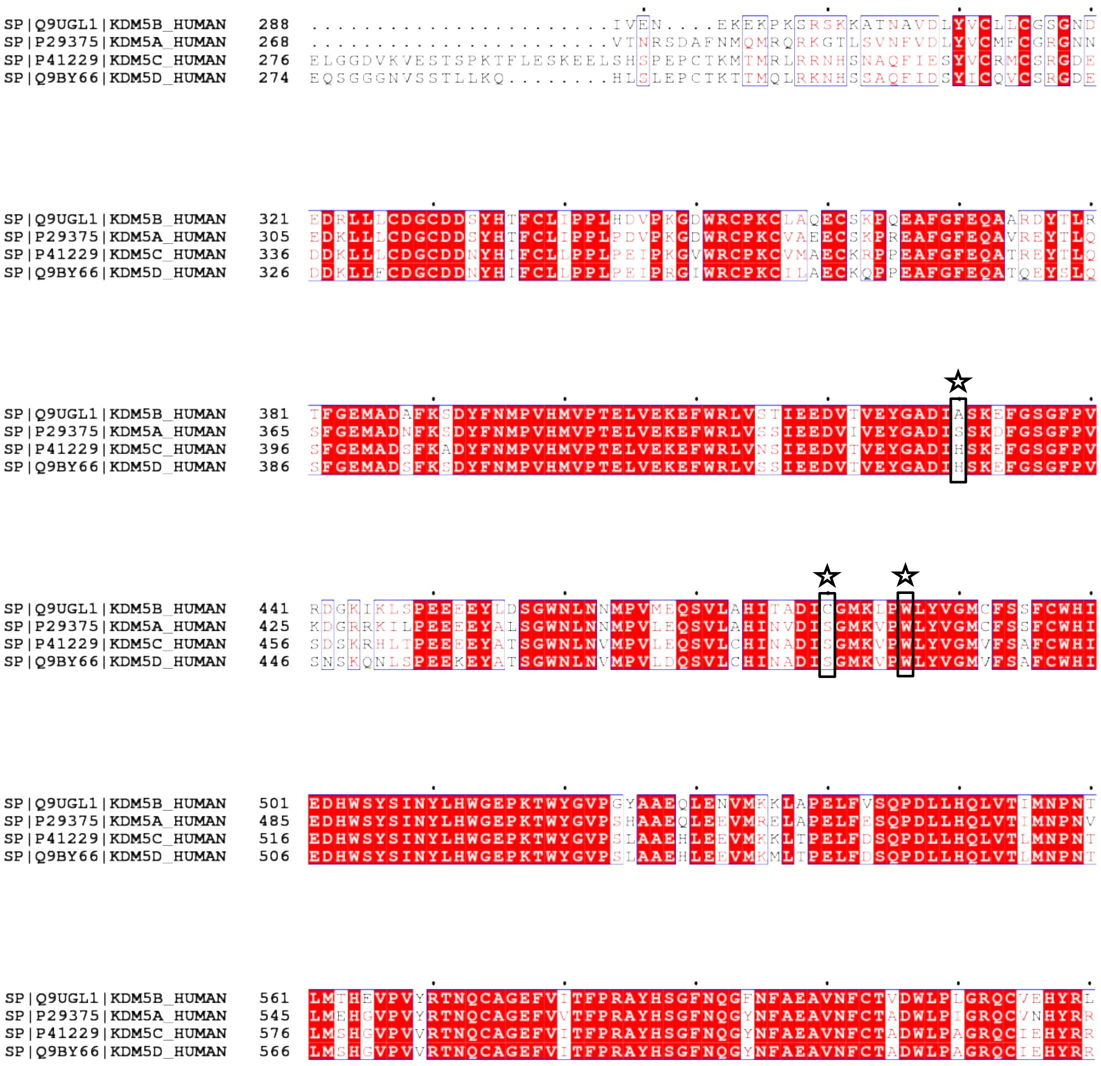

Figure S4: Sequence alignment of KDM5A, B, C and D. Residues targeted to gain potency and/or selectivity are highlighted with a black star and a rectangle. Alignments have been generated with Clustal Omega (Sievers *et al.*, 2011), and the corresponding figure generated with ESPript 3.0 (Robert *et al.*, 2014).

Sievers, F.; Wilm, A.; Dineen, D.G.; Gibson, T.J.; Karplus, K.; Li, W.; Lopez, R.; McWilliam, H.; Remmert, M.; Söding, J.; Thompson, J.D.; Higgins, D.G. Fast, scalable generation of high-quality protein multiple sequence alignments using Clustal Omega. *Mol. Syst. Biol.* **2011**, *7*, 539.

Robert, X.; Gouet, P. Deciphering key features in protein structures with the new ENDscript server. *Nucleic Acids Res.* **2014**, *42*, W320−W324.

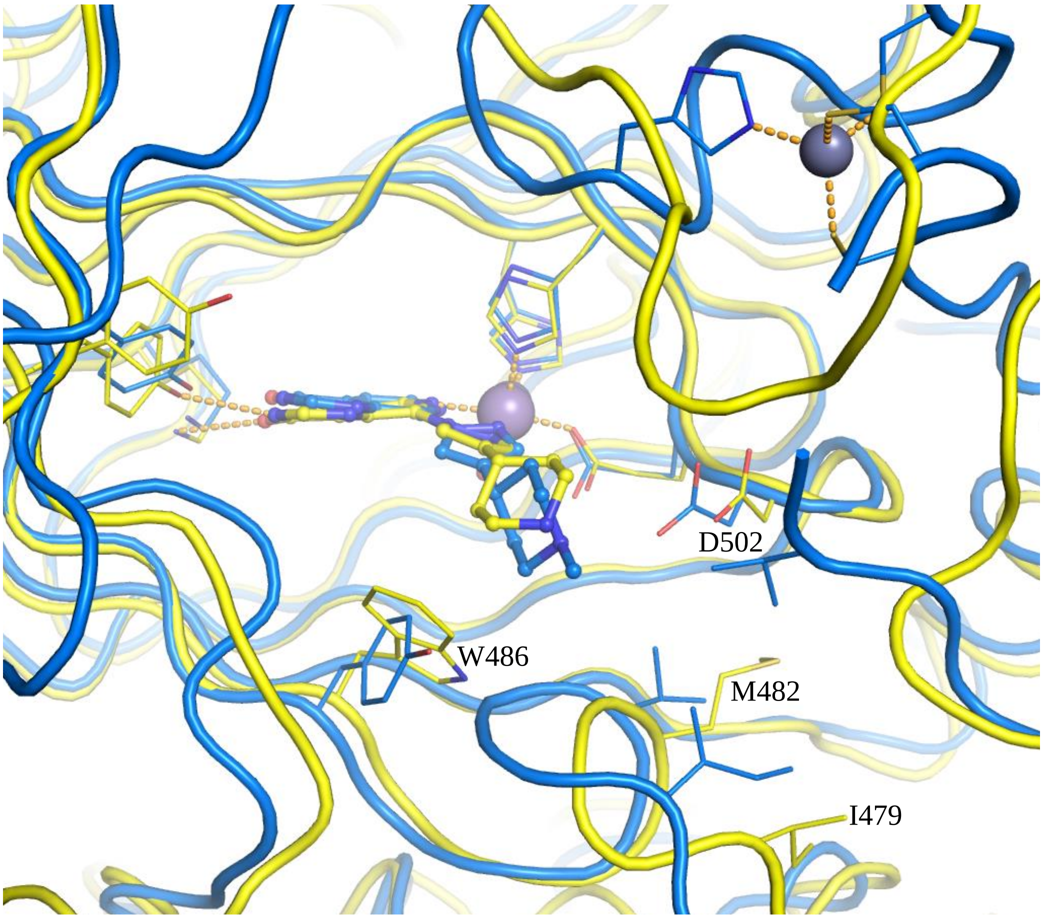

Figure S5: Overlay of crystal structures of **34a** bound to KDM4A (blue) or KDM5B (yellow). Zn(II) or Mn(II) atoms are shown as spheres. Proteins are represented as a cartoon tubes, plus lines for some key residues. Compound **16a** is shown in ball and sticks representation. Labels correspond to KDM5B residue numbers. KDM5B loop 91-100 has been removed due to its suspected construct-linked artefactual position.

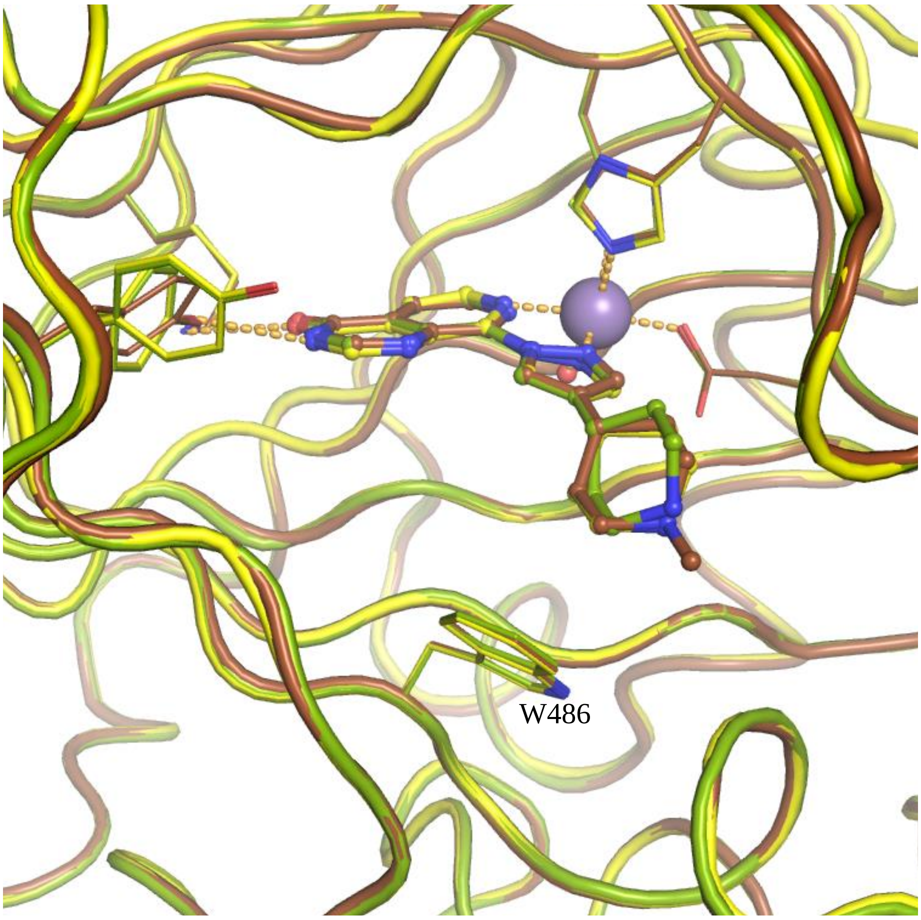

Figure S6: Overlay of crystal structures of **34a** (yellow), **34f** (brown) and **34g** (green) bound to KDM5B. Mn(II) atom is shown as a sphere. Proteins are represented as a cartoon tubes, plus lines for some key residues. Compounds are shown in ball and sticks representation. KDM5B loop 91-100 has been removed due to its suspected construct-linked artefactual position.

Table S1: Crystallographic data collection and refinement statistics.

Protein construct	KDM4A 1-359	KDM4A 1-359	KDM4A 1-359
Ligand	**16a**	**16m**	**17b**
PDB code	**6H4P**	**6H4S**	**6H4X**
Crystal			
Space group	P 1 2_1 1	P 1 2_1 1	P 1 2_1 1
Unit cell dimensions (a/b/c in Å)	58.24/102.06/143.32	57.69/101.37/142.93	57.83/101.40/142.54
Unit cell angles (α/β/γ in °)	90/99.24/90	90/99.25/90	90/99.52/90
Data collection and processing			
Beamline	DLS I03	In-house Rigaku	DLS I03
Wavelength (Å)	0.9762	1.5419	0.9762
Integration program	XDS	XDS	XDS
Reduction program	AIMLESS	AIMLESS	AIMLESS
Resolution range	102.09 – 2.19	49.64 – 2.45	49.71 – 2.34
Number of unique reflections [a]	84582 (6271)	59858 (4700)	68376 (4581)
Completeness [a]	99.9 (100)	100 (100)	99.8 (99.9)
Redundancy [a]	6.7 (6.6)	7.5 (7.2)	6.7 (6.9)
R_{merge} (%) [a]	10 (123.8)	30 (208.5)	11.1 (152.1)
I/σ(I) [a]	9.2 (1.3)	6.2 (1.1)	9.6 (1.1)
$CC_{1/2}$ [a, b]	0.997 (0.519)	0.985 (0.325)	0.997 (0.37)
Refinement			
Program	BUSTER	BUSTER	BUSTER
R_{work} (%)	16.98	17.31	16.54
R_{free} (%)	20.70	21.78	20.71
Number of residues	1372	1352	1358
Number of water molecules	647	638	480
Average B-factor ($Å^2$)	61.35	47.65	61.52
Ramachandran favoured (%)	98.08	97.81	98.28
Ramachandran outliers (%)	0	0	0
RMSD bonds (Å)	0.010	0.010	0.010
RMSD angles (°)	1.71	1.74	1.71

[a] Values in parentheses are for the highest resolution shell.

[b] Half-dataset correlation coefficient, see: Karplus, P. A.; Diederichs, K. Linking crystallographic model and data quality. *Science* **2012**, *336*, 1030−1033.

Protein construct	KDM4A 1-359	KDM4A 1-359	KDM4A 1-359
Ligand	**17e**	**17f**	**18a**
PDB code	**6H4Y**	**6H4R**	**6H4O**
Crystal			
Space group	$P\ 1\ 2_1\ 1$	$P\ 1\ 2_1\ 1$	$P\ 1\ 2_1\ 1$
Unit cell dimensions (a/b/c in Å)	58.05/100.76/143.18	58.11/101.47/142.12	57.52/101.37/142.54
Unit cell angles (α/β/γ in °)	90/99.56/90	90/99.41/90	90/99.50/90
Data collection and processing			
Beamline	DLS I03	DLS I03	DLS I02
Wavelength (Å)	0.9762	0.9762	0.9795
Integration program	XDS	XDS	XDS
Reduction program	AIMLESS	AIMLESS	AIMLESS
Resolution range	100.76 – 2.38	49.29 – 2.14	48.95 – 2.25
Number of unique reflections [a]	65181 (4834)	89600 (4579)	76575 (4568)
Completeness [a]	99.8 (100)	99.8 (99.7)	99.9 (100)
Redundancy [a]	6.5 (6.9)	6.7 (7)	6.6 (6.6)
R_{merge} (%) [a]	9.9 (124.9)	12.6 (204.7)	12.8 (187.4)
I/σ(I) [a]	9.2 (1.3)	9.2 (1.2)	10.0 (1.4)
$CC_{1/2}$ [a, b]	0.996 (0.511)	0.996 (0.397)	0.996 (0.357)
Refinement			
Program	BUSTER	BUSTER	BUSTER
R_{work} (%)	17.39	17.95	16.90
R_{free} (%)	20.87	21.90	20.26
Number of residues	1356	1356	1358
Number of water molecules	495	741	467
Average B-factor ($Å^2$)	70.10	55.24	59.64
Ramachandran favoured (%)	98.5	97.60	98.05
Ramachandran outliers (%)	0	0	0
RMSD bonds (Å)	0.010	0.010	0.010
RMSD angles (°)	1.74	1.73	1.71

[a] Values in parentheses are for the highest resolution shell.

[b] Half-dataset correlation coefficient, see: Karplus, P. A.; Diederichs, K. Linking crystallographic model and data quality. *Science* **2012**, *336*, 1030−1033.

Protein construct	KDM4A 1-359	KDM4A 1-359	KDM4A 1-359
Ligand	**19a**	**19d**	**34a**
PDB code	**6H4T**	**6H4W**	**6H4Q**
Crystal			
Space group	P 1 2_1 1	P 1 2_1 1	P 1 2_1 1
Unit cell dimensions (a/b/c in Å)	57.86/101.81/142.68	57.56/101.49/142.31	57.42/101.80/142.11
Unit cell angles (α/β/γ in °)	90/99.20/90	90/99.07/90	90/99.55/90
Data collection and processing			
Beamline	In-house Rigaku	In-house Rigaku	In-house Rigaku
Wavelength (Å)	1.5419	1.5419	1.5419
Integration program	XDS	XDS	XDS
Reduction program	AIMLESS	AIMLESS	AIMLESS
Resolution range	47.87 – 2.38	48.92 – 2.81	49.71 – 2.31
Number of unique reflections [a]	65581 (4581)	39555 (4429)	70808 (4568)
Completeness [a]	100 (100)	100 (100)	100 (100)
Redundancy [a]	14.7 (11.9)	8.5 (7.6)	14.2 (10.3)
R_{merge} (%) [a]	26.2 (366)	38.4 (196)	96.9 (428.3)
I/σ(I) [a]	9.5 (0.8)	7.5 (0.9)	3.5 (1)
$CC_{1/2}$ [a, b]	0.995 (0.320)	0.942 (0.381)	0.924 (0.359)
Refinement			
Program	BUSTER	BUSTER	BUSTER
R_{work} (%)	17.35	21.80	17.29
R_{free} (%)	21.59	26.62	21.29
Number of residues	1360	1352	1377
Number of water molecules	749	176	981
Average B-factor (Å^2)	50.53	53.96	30.69
Ramachandran favoured (%)	98.06	96.83	98.16
Ramachandran outliers (%)	0	0	0
RMSD bonds (Å)	0.010	0.010	0.010
RMSD angles (°)	1.76	1.77	1.75

[a] Values in parentheses are for the highest resolution shell.

[b] Half-dataset correlation coefficient, see: Karplus, P. A.; Diederichs, K. Linking crystallographic model and data quality. *Science* **2012**, *336*, 1030−1033.

Protein construct	KDM4A 1-359	KDM4A 1-359	KDM5B 26-771Δ101−374
Ligand	**34b**	**34g**	**16a**
PDB code	**6H4U**	**6H4V**	**6H4Z**
Crystal			
Space group	P 1 2_1 1	P 1 2_1 1	P 6_5 2 2
Unit cell dimensions (a/b/c in Å)	58.97/104.22/145.04	58.83/103.78/144.03	144.48/144.48/154.35
Unit cell angles (α/β/γ in °)	90/99.57/90	90/99.45/90	90/90/120
Data collection and processing			
Beamline	DLS I04-1	DLS I04-1	DLS I04-1
Wavelength (Å)	0.9282	0.9282	0.9282
Integration program	XDS	XDS	XDS
Reduction program	AIMLESS	AIMLESS	AIMLESS
Resolution range	49.30 – 2.21	49.07 – 2.15	77.18 – 2.30
Number of unique reflections [a]	86644 (4589)	92826 (4578)	42633 (2975)
Completeness [a]	100 (100)	100 (100)	99.7 (96.5)
Redundancy [a]	6.9 (7)	6.9 (7.1)	19.6 (19.6)
R_{merge} (%) [a]	8.8 (255.5)	7.4 (195.6)	11.3 (173.8)
I/σ(I) [a]	11.7 (0.9)	12.2 (0.9)	21.0 (1.8)
$CC_{1/2}$ [a, b]	0.997 (0.306)	0.998 (0.302)	0.999 (0.656)
Refinement			
Program	BUSTER	BUSTER	BUSTER
R_{work} (%)	17.59	17.57	18.62
R_{free} (%)	20.79	21.08	21.14
Number of residues	1380	1367	458
Number of water molecules	711	678	304
Average B-factor ($Å^2$)	61.40	61.31	60.51
Ramachandran favoured (%)	98.31	98.44	98.45
Ramachandran outliers (%)	0	0	0
RMSD bonds (Å)	0.010	0.010	0.010
RMSD angles (°)	1.68	1.67	1.70

[a] Values in parentheses are for the highest resolution shell.

[b] Half-dataset correlation coefficient, see: Karplus, P. A.; Diederichs, K. Linking crystallographic model and data quality. *Science* **2012**, *336*, 1030−1033.

Protein construct	KDM5B 26-771Δ101−374	KDM5B 26-771Δ101−374	KDM5B 26-771Δ101−374
Ligand	**34a**	**34f**	**34g**
PDB code	**6H50**	**6H51**	**6H52**
Crystal			
Space group	P 6_5 2 2	P 6_5 2 2	P 6_5 2 2
Unit cell dimensions (a/b/c in Å)	142.36/142.36/152.78	142.00/142.00/152.41	142.12/142.12/152.40
Unit cell angles (α/β/γ in °)	90/90/120	90/90/120	90/90/120
Data collection and processing			
Beamline	DLS I03	DLS I03	DLS I03
Wavelength (Å)	0.9763	0.9763	0.9763
Integration program	XDS	XDS	XDS
Reduction program	AIMLESS	AIMLESS	AIMLESS
Resolution range	47.97 – 2.19	51.95 – 2.21	51.97 – 2.14
Number of unique reflections [a]	47457 (3450)	45895 (3334)	50252 (3516)
Completeness [a]	99.9 (99.9)	99.9 (100)	99.5 (96.5)
Redundancy [a]	16.8 (8.3)	17.1 (8.9)	16.1 (7.2)
R_{merge} (%) [a]	11.4 (128.4)	9.4 (149.1)	8.0 (145.5)
I/σ(I) [a]	18.3 (1.4)	18.7 (1.3)	20.4 (1.1)
$CC_{1/2}$ [a, b]	0.999 (0.494)	0.999 (0.460)	0.999 (0.436)
Refinement			
Program	BUSTER	BUSTER	BUSTER
R_{work} (%)	18.21	18.19	18.47
R_{free} (%)	20.58	19.72	20.28
Number of residues	456	454	458
Number of water molecules	364	282	321
Average B-factor (Å^2)	54.77	61.54	58.98
Ramachandran favoured (%)	97.56	97.75	97.56
Ramachandran outliers (%)	0	0	0
RMSD bonds (Å)	0.010	0.010	0.010
RMSD angles (°)	1.73	1.70	1.69

[a] Values in parentheses are for the highest resolution shell.

[b] Half-dataset correlation coefficient, see: Karplus, P. A.; Diederichs, K. Linking crystallographic model and data quality. *Science* **2012**, *336*, 1030−1033.

Compound **16m**

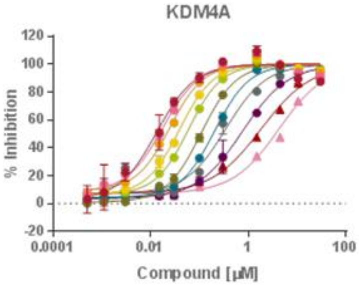

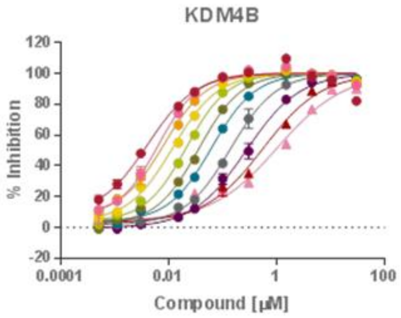

Compound 16m	KDM4B	KDM4A
**2OG (µM)**	**IC_{50} (µM)**	**IC_{50} (µM)**
**0.25**	0.004	0.013
**0.5**	0.006	0.017
**1**	0.008	0.025
**2**	0.012	0.039
**4**	0.021	0.060
**8**	0.036	0.108
**16**	0.062	0.204
**50**	0.138	0.346
**100**	0.270	0.758
**300**	0.568	1.842
**1000**	1.044	4.286
**Fold change**	261	330

Compound **34f**

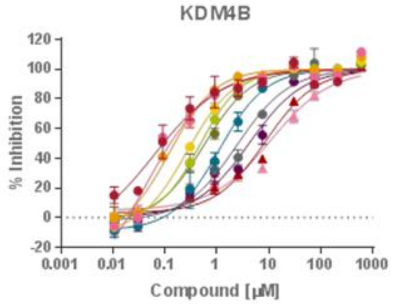

Compound 34f	KDM4B
**2OG (µM)**	**IC_{50} (µM)**
**0.25**	0.066
**0.5**	0.040
**1**	0.121
**2**	0.265
**4**	0.477
**8**	0.564
**16**	1.106
**50**	2.437
**100**	4.209
**300**	8.680
**1000**	11.060
**Fold change**	168

Figure S7: KDM4A and KDM4B inhibition by compounds **16m** and **34f** (in vitro biochemical assay): 2OG co-substrate competition studies.

^{1}H-NMR spectra of representative compounds

Compound **16g**

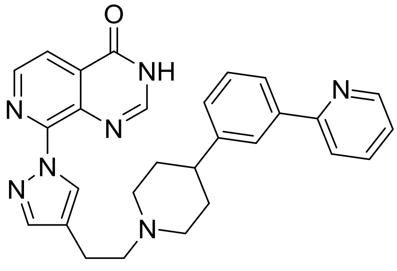

16g

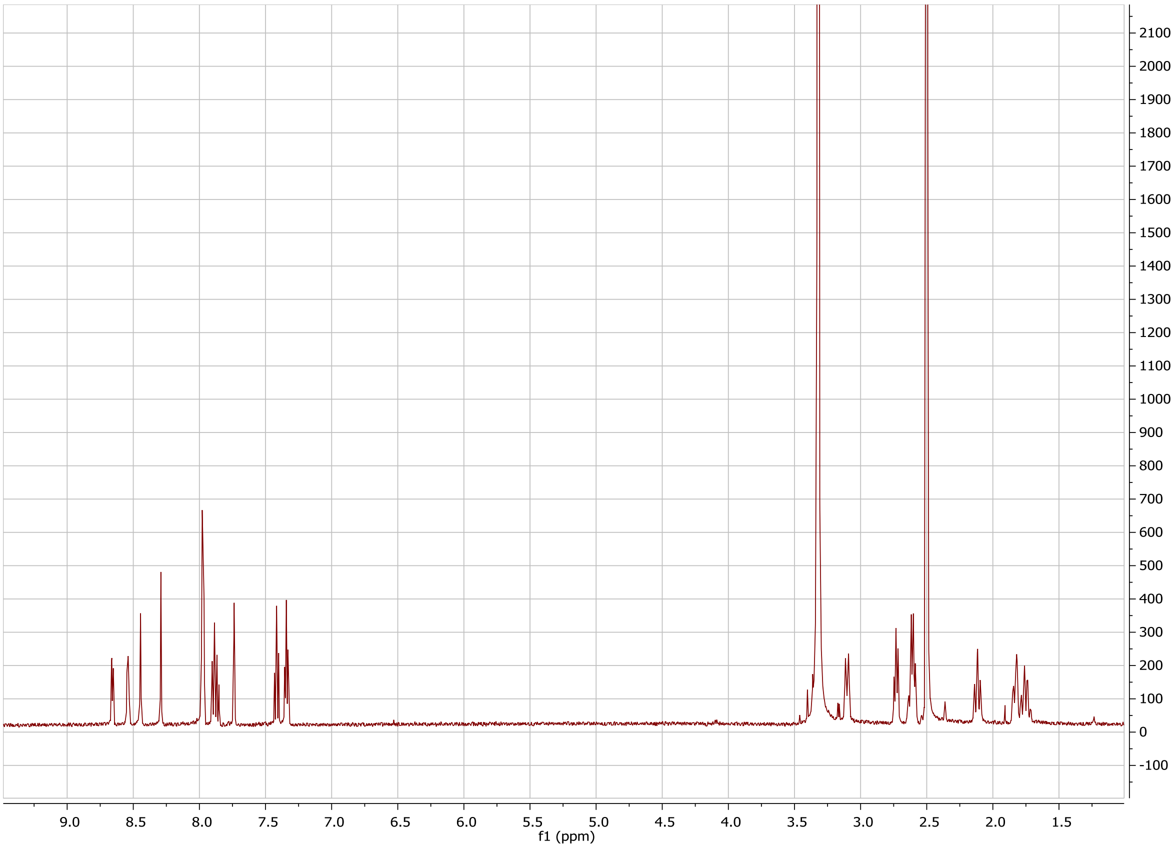

Compound **16i**

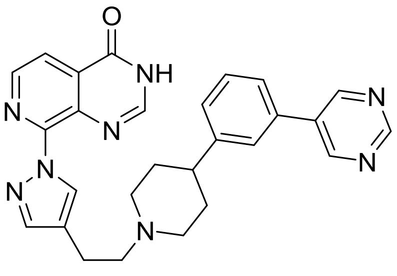

16i

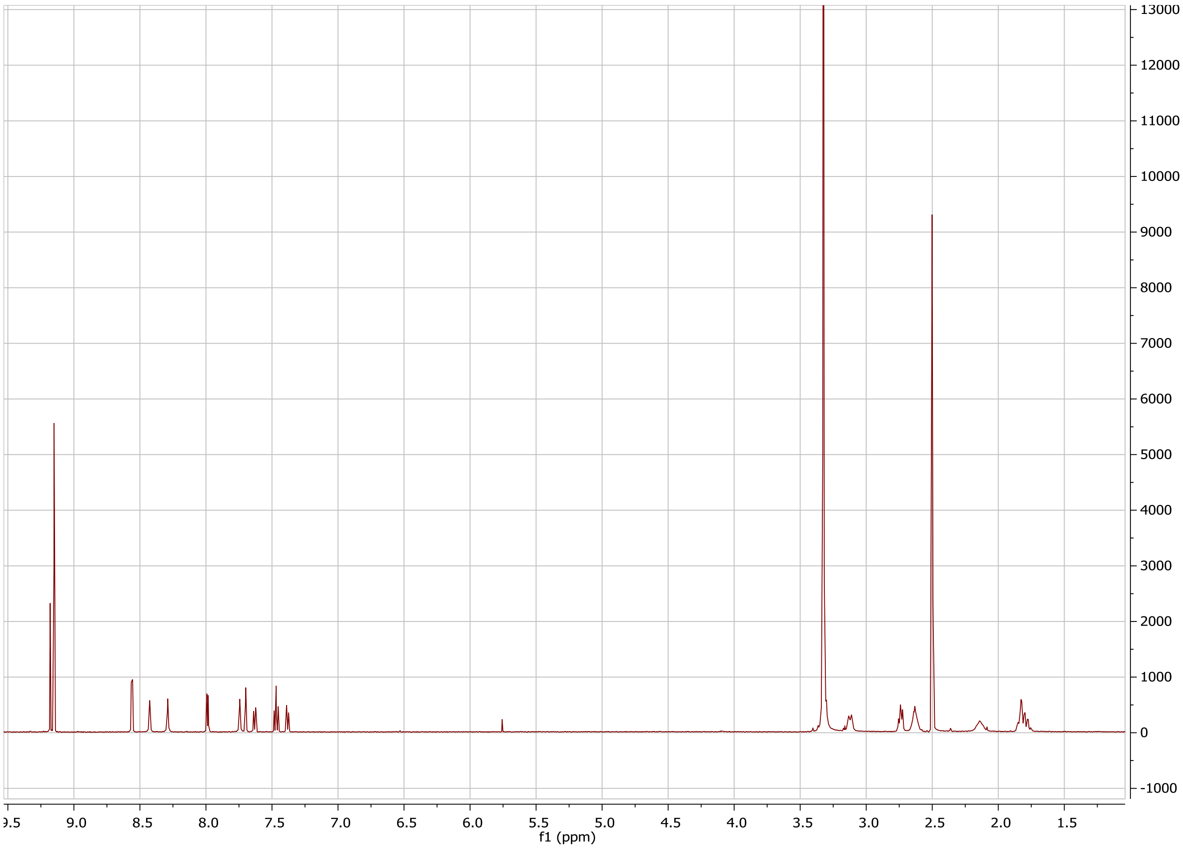

Compound **17c**

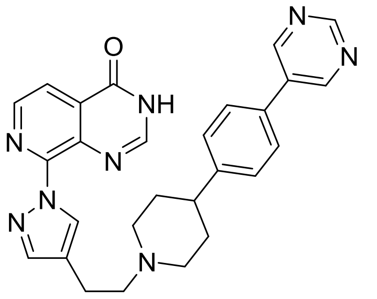

17c

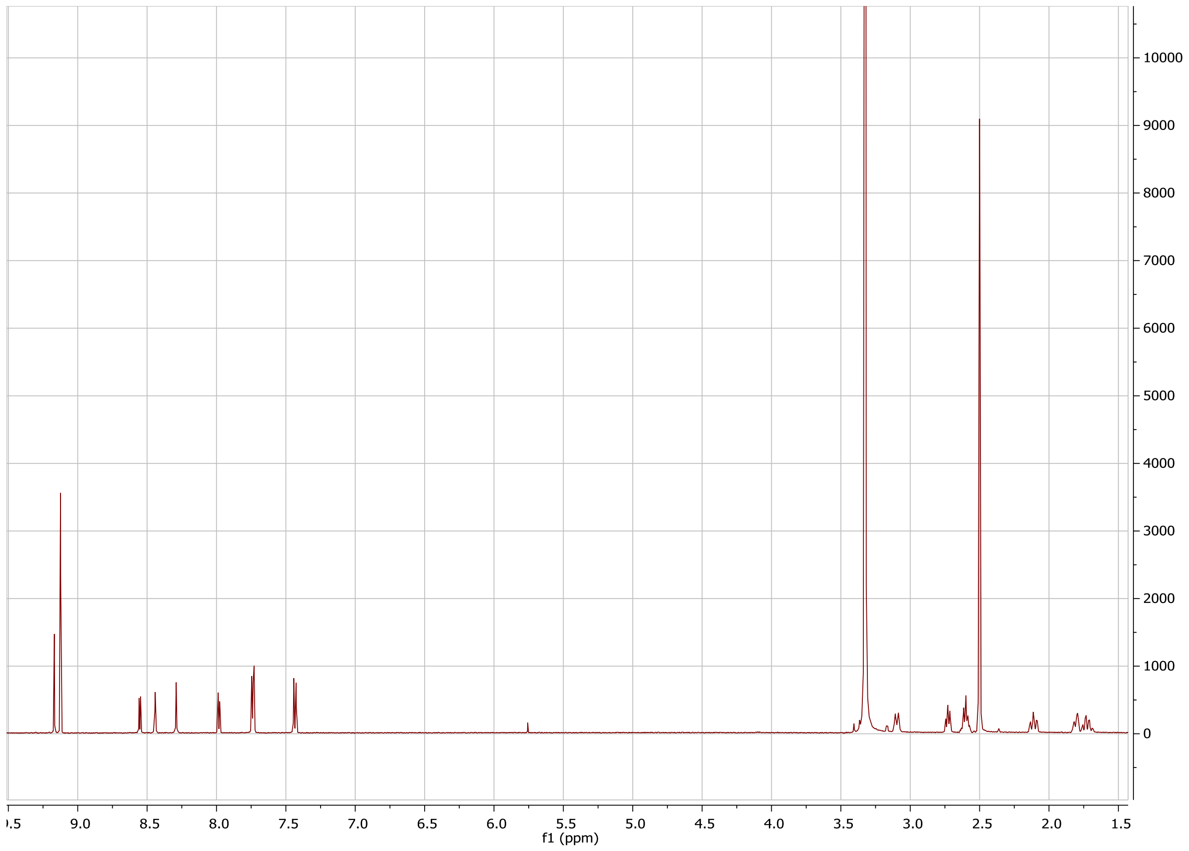

Compound **17g**

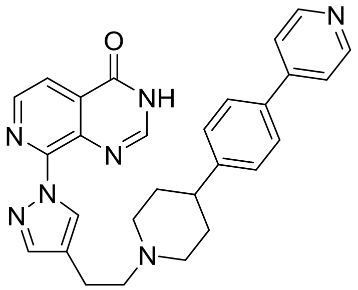

17g

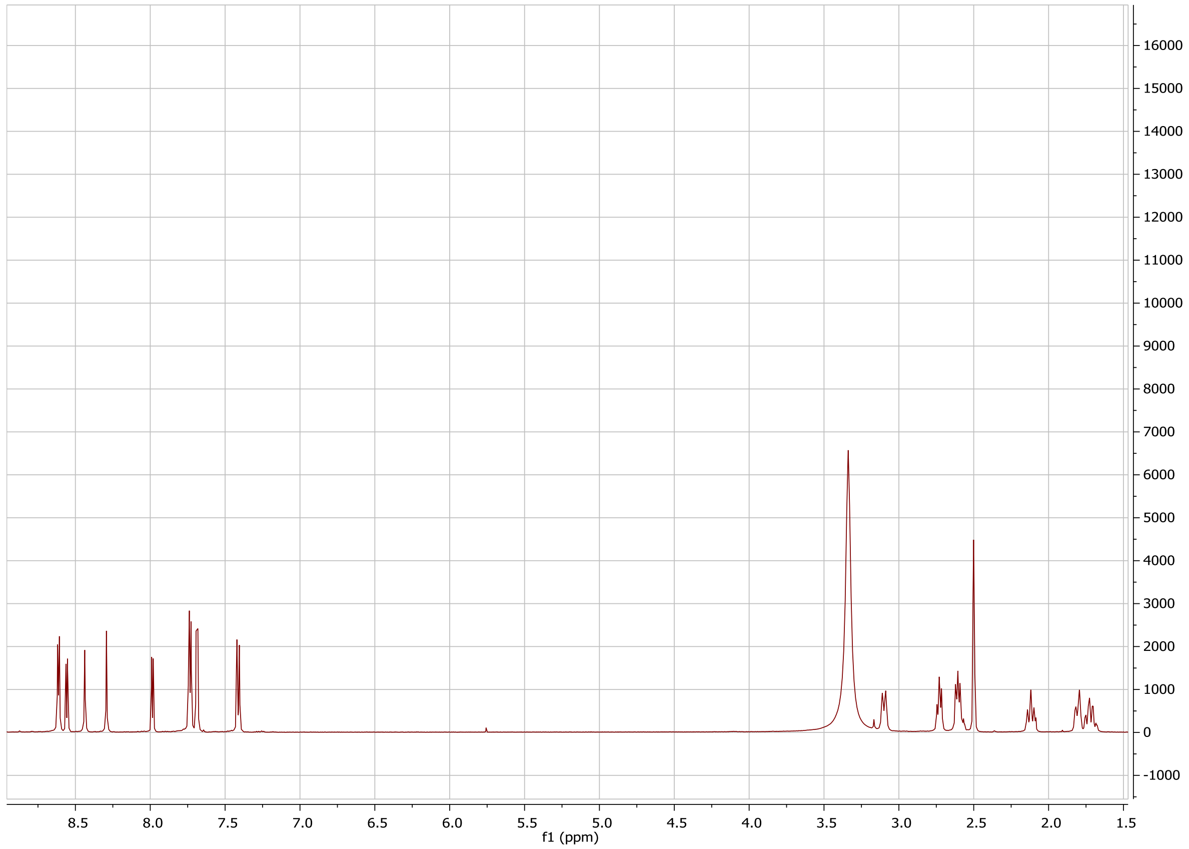

Compound **19d**

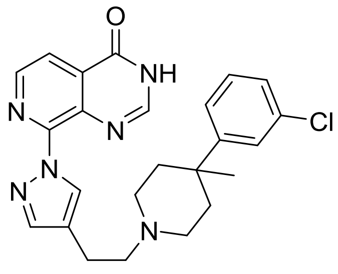

19d

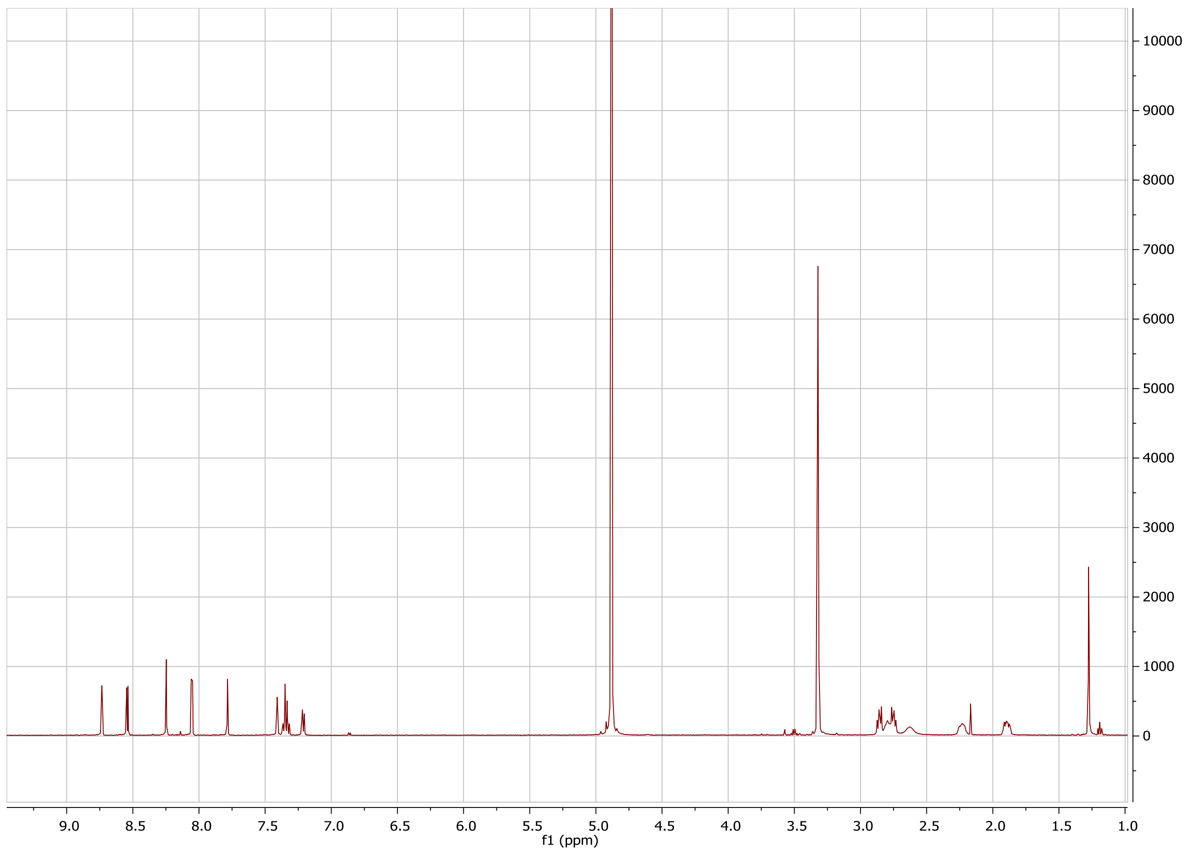

Compound **34c**

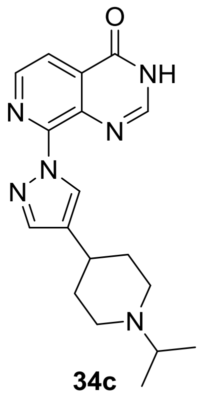

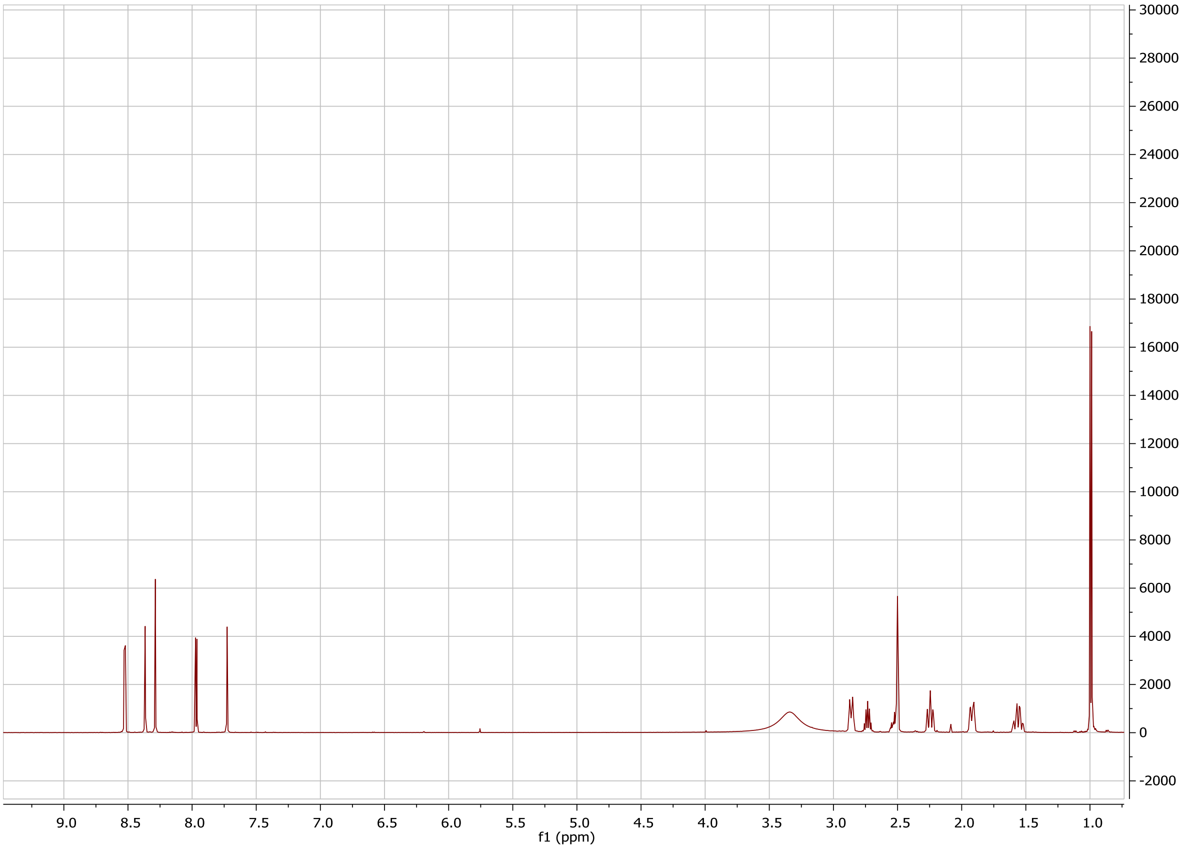

Supplement: Multimedia component 1 [file mmc1.pdf]
